# Protein Cargo of Extracellular Vesicles From Bovine Follicular Fluid and Analysis of Their Origin From Different Ovarian Cells

**DOI:** 10.3389/fvets.2020.584948

**Published:** 2020-11-04

**Authors:** Svetlana Uzbekova, Carmen Almiñana, Valerie Labas, Ana-Paula Teixeira-Gomes, Lucie Combes-Soia, Guillaume Tsikis, Anais Vitorino Carvalho, Rustem Uzbekov, Galina Singina

**Affiliations:** ^1^CNRS, IFCE, INRAE, Université de Tours, PRC, Nouzilly, France; ^2^Functional Genomics, Vetsuisse Faculty Zurich, Institute of Veterinary Anatomy, University of Zurich, Zurich, Switzerland; ^3^CHU de Tours, INRAE, Université de Tours, PRC, CIRE, Tours, France; ^4^INRAE, Université de Tours, ISP, Nouzilly, France; ^5^Faculty of Medecine, University of Tours, Tours, France; ^6^Faculty of Bioengineering and Bioinformatics, Moscow State University, Moscow, Russia; ^7^L. K. Ernst Federal Science Center for Animal Husbandry, Podolsk, Russia

**Keywords:** extracellular vesicles, exosomes, follicular fluid, follicular cells, oocyte, bovine, proteome, transcriptome

## Abstract

Follicular fluid (FF) fills the interior portion of the ovarian antral follicle and provides a suitable microenvironment for the growth of the enclosed oocyte through molecular factors that originate from plasma and the secretions of follicular cells. FF contains extracellular nanovesicles (ffEVs), including 30–100-nm membrane-coated exosomes, which carry different types of RNA, proteins, and lipids and directly influence oocyte competence to develop embryo. In the present study, we aimed to characterize the protein cargo of EVs from the FF of 3–6-mm follicles and uncover the origins of ffEVs by assessing expression levels of corresponding mRNAs in bovine follicular cells and oocyte and cell proteomes. Isolated exosome-like ffEVs were 53.6 + 23.3 nm in size and could be internalized by cumulus-oocyte complex. Proteomes of ffEVs and granulosa cells (GC) were assessed using nanoflow liquid chromatography coupled with high-resolution tandem mass spectrometry after the gel fractionation of total proteins. In total, 460 protein isoforms corresponding to 322 unique proteins were identified in ffEVs; among them, 190 were also identified via GC. Gene Ontology terms related to the ribosome, protein and RNA folding, molecular transport, endocytosis, signal transduction, complement and coagulation cascades, apoptosis, and developmental biology pathways, including PI3K-Akt signaling, were significantly enriched features of ffEV proteins. FfEVs contain numerous ribosome and RNA-binding proteins, which may serve to compact different RNAs to regulate gene expression and RNA degradation, and might transfer ribosomal constituents to the oocyte. Majority of genes encoding ffEV proteins expressed at different levels in follicular cells and oocyte, corroborating with numerous proteins, which were reported in bovine oocyte and cumulus cells in other studies thus indicating possible origin of ffEV proteins. The limited abundance of several mRNAs within follicular cells indicated that corresponding ffEV proteins likely originated from circulating exosomes released by other tissues. Analysis of bovine ffEV transcriptome revealed that mRNAs present in ffEV accounted for only 18.3% of detected ffEV proteins. In conclusion, our study revealed numerous proteins within ffEVs, which originated from follicular and other cells. These proteins are likely involved in the maintenance of follicular homeostasis and may affect oocyte competence.

## Introduction

In mammals, the antral ovarian follicle is filled with follicular fluid (FF), which provides an optimal environment for oocyte growth, meiotic maturation, and the acquisition of oocyte competence for embryonic development (1, 2). The FF is derived from both blood plasma and the secretions of different types of somatic follicular cells (cumulus, granulosa, and thecal layers). The active exchange of molecular factors occurs between follicular cells and the oocyte via different mechanisms of cell-to-cell communication including extracellular vesicles (EVs) (3–6). EVs are enclosed within membranes and can be classified into three principal groups according to their biogenesis and size, as follows: exosomes (30–150 nm), macrovesicles (100–1,000 nm), and apoptotic bodies (7). Exosomes are released to the extracellular space from intracellular multivesicular bodies on fusion with the plasma membrane. They contain different molecular cargo, which includes proteins, lipids, and different types of RNAs and DNA. These factors can be transferred to target cells to facilitate cell-to-cell communication and signal transmission in different physiological systems, including reproductive organs (4, 7, 8).

Recently, EVs have been identified in most types of maternal reproductive fluids (follicular, oviductal, and uterine fluids) (4, 9–13). It has been suggested that exosomes, like extracellular vesicles identified in the follicular fluid (ffEVs), mediate molecular signaling between follicle cells and may modulate the functions of target cells [reviewed by Tesfaye et al. (6)]. It has been shown that bovine ffEVs are capable of stimulating granulosa cell proliferation (14) and cumulus expansion *in vitro* (15). These effects were associated with the sizes of follicles from which ffEVs originated and revealed that ffEVs from small follicles (3–6 mm) were preferentially taken up by follicular cells vs. larger follicles (14). Further, research revealed that these follicles affected oocyte competence to a greater degree than large follicles (16), and displayed increased blastocyst rates (16) and modulated gene expression in cumulus cells (CC) (17). However, the addition of ffEVs during *in vitro* maturation (IVM) did not affect oocyte maturation rates (17). In contrast, when ffEV supplementation was assessed throughout the IVM of heat-stressed oocytes, increases in the rates of cell cleavage and embryo development were observed (18). Interestingly, rates were similar to those associated FF supplementation, which improved the quality of blastocysts produced *in vitro* (19). Taken together, recent studies have shown that ffEVs play important roles in the process of oocyte maturation by affecting functional mechanisms of meiotic division, cytoplasm maturation, and stress protection via different pathways. Moreover, molecules that are released from surrounding follicular cells into FF via ffEVs could serve as non-invasive molecular markers of oocyte competence (6).

To date, most studies of the molecular cargo of ffEVs have focused on ffEV miRNA and their roles in the regulation of target cell gene expression after EV uptake by granulosa cells (GG) and cumulus-oocyte complex (COC) (11, 17, 20–23). A number of miRNAs have been determined to be up- or downregulated in small subordinate follicle ffEVs compared with larger ones. In addition, some miRNAs of ffEV have been associated with cellular proliferation and inflammatory response pathways (23). Differences have been also observed between ffEV miRNAs of hyperstimulated and unstimulated heifers. Differentially expressed genes identified were associated with genes involved in oocyte meiosis and MAPK and TGF-beta signaling pathways (22). Furthermore, different cargo of ffEV miRNAs have been identified in follicles with low and high progesterone levels, and some activate numerous signaling pathways in CC or COCs after 9 h IVM (17). In post-calving cows, miRNA expression patterns of ffEVs of cows with different degrees of energy balance differed. Further, differential expression of miRNA-targeting genes involved in TGF-beta, mTOR, and PI3K signaling, apoptosis, the cell cycle, adherent junction, and other biological pathways important for reproductive functions was observed (24).

Data regarding the molecular components of EVs and their release from different cell types that were determined *in vivo, in vitro*, and from the assessment of variety of biological fluids from different species have been deposited in the public online database, Vesiclepedia (25). In 2019, this database contained 10,500 miRNA entries, 27,600 mRNA entries, and 350,000 protein entries (26). Compared with other species, proteomic data derived from bovine EVs are scarce. In fact, only 1,562 protein IDs from bovine EVs have been reported up to date, compared with 12,800 protein IDs that have been identified from human EVs. In cows, EV proteins have previously been identified from plasma (27), urine, saliva, milk (28), and oviduct fluid (10). However, no comprehensive analysis of the bovine ffEV protein cargo has yet been performed. In contrast, the protein cargo of ffEVs has been assessed in equine and human ffEVs, and 73 and 662 proteins were identified, respectively (20, 29). Moreover, a comparison of the abundance of ffEV proteins in control and polycystic ovary syndrome ovaries could potentially identify potential markers of oocyte quality (29).

FfEVs and their cargo have the potential to affect oocyte competence acquisition, which is necessary for ensuring optimal embryo development, and they are potential markers of oocyte quality. Despite its potential utility, minimal data regarding protein cargo of bovine ffEVs are available. Therefore, the present study aimed to characterize the protein cargo of bovine ffEVs, particularly within exosomes. Moreover, because follicles contain different types of follicular cells (granulosa, theca, and CC), and because enclosed oocytes are within FF, we aimed to reveal the origin of ffEVs by analyzing ffEV proteins and corresponding mRNA expression levels in follicular cells.

## Materials and Methods

### Ethics

No experiments involving living animals were performed.

### Chemicals

All chemicals were purchased from Sigma-Aldrich (Saint-Quentin Fallavier, France), unless otherwise specified.

### The Isolation of Extracellular Vesicles From Follicular Fluid

Follicular fluid (FF) contained in follicles of 3–6 mm was obtained from a batch of 20–40 bovine ovaries from three independent experiments. In total, three ffEV samples from three pools of FF aspirates and the corresponding three GC samples were obtained. Then, ffEV samples were isolated from the three independent experiments by serial centrifugation to clarify the FF preparations followed by ultracentrifugation based on the protocol used by Almiñana et al. (10, 30, 31) for isolation and characterization of oviductal EVs in bovine and porcine. The protocol was based on the original protocol for isolating EVs from fluids reported by Thery et al. (32) with a few modifications. The protocol used is similar to da Silveira et al. protocol to isolate EVs from FF preparations from equine and bovine (16, 33). First, FF samples were clarified via centrifugation at 300 × *g* for 15 min to remove remaining cells, followed by a 15-min centrifugation step at 2,000 × *g* to eliminate cell debris and apoptotic bodies. Then, clarified FF (5-ml samples) was centrifuged 30 min at 12,000 × *g* at 4°C to remove microvesicles. Supernatants were transferred to clear tubes and centrifuged at 100,000 × *g* 90 min at 4°C (Beckman centrifuge L8-M, SW41T1 rotor). Pellets were washed in phosphate-buffered saline (PBS) and centrifuged at 100,000 × *g* 90 min. Final ffEV pellets were diluted with 25 μl PBS and stored at −80°C.

### Transmission Electron Microscopic Analysis of Extracellular Vesicles

Transmission electron microscopy (TEM) was performed to assess all the ffEV pools using the protocol previously employed (10, 30, 34). Briefly, for TEM analyses, 2.5-μl ffEV samples were fixed in 5 μl of 2% glutaraldehyde solution in PBS. Then, 3 μl of each fixed ffEV suspension was placed on a Formvar/Carbon coated grid and incubated for 5 min at room temperature (20–22°C). Samples were washed with distilled water three times, stained with 2% uranyl acetate, and air dried at room temperature. Micrographs were obtained using a Hitachi HT 7700 Elexience TEM at 80 kV (with an AMT charge-coupled device camera) and a JEM 1011 TEM (JEOL, Japan) equipped with a Gatan digital camera that required Digital Micrograph software (Gatan, Pleasanton, United States) at 100 kV. EV size measurements were performed using ImageJ software (NIH, Bethesda, United States).

### Uptake of ffEV by COCs

To examine the uptake of ffEV by COCs, ffEV samples were labeled with PKH67, a lipophilic green fluorescent dye using the Green Fluorescent Cell Linker Kit (MINI67) in accordance with the manufacturers' recommendations, with minor modifications. Briefly, 30 μl of the ffEV suspension (environ 100 μg of EV), or 30 μl of PBS (negative control), was added to 125 μl of diluent C. Second, 1 μl PKH67 (4 μM) was added to 125 μl of the same diluent. Then, both solutions were mixed. Tubes containing EV-PKH67 and PBS-PH67 mixtures were incubated for 5 min at room temperature in the dark. To stop the reactions, 250 μL of 16% BSA solution in PBS was added. After incubating for 1 min, 3.5 ml sterile, filtered PBS was added to each sample. Tubes with labeled vesicles (PKH67-EV) and controls (PKH67-PBS) were ultracentrifuged at 100,000 × *g* for 30 min at 4°C to pellet EVs. Liquid was completely removed from each tube and both EV and PBS pellets were resuspended in 100 μl culture medium (TCM199 without phenol red, M3769). Both green-labeled ffEVs and control samples were stored at 4°C for 1 day before being used for COC uptake studies.

IVM was performed using selected bovine COCs in 500 ml TCM199 without phenol red supplemented with 100 ng/ml epidermal growth factor (EGF) as earlier described (35). Then, 25 μl of either labeled EVs (PKH67-EV) or control preparations (PBS-PHK67) were added to COCs for 24 h at 38.8°C, 5% CO_2_, and 20% O_2_. After IVM, COCs were rinsed two times for 10 min in phenol-red-free TCM199 and twice with warm PBS/0.1% BSA. To observe the nuclei of cells, COCs were fixed in 4% paraformaldehyde in PBS for 10 min, washed with PBS/0.1% BSA, and stained 15 min using Hoechst 33342 solution (2 μg/ml). After washing two times for 5 min in PBS, COCs were mounted onto slides containing Moveol solution. Finally, COCs were observed under a Zeiss LSM700 confocal microscope (Carl Zeiss Microscopy GmbH, Munich, Germany) using the ×20 and ×40 oil immersion objectives with appropriate filters. Images were taken using Zen 2012 version 8.0 software (Carl Zeiss Microscopy).

### Proteomic Analysis

Total protein concentrations within ffEV and GC preparations were measured using a bicinchoninic acid assay (BCA; Interchim, Montluçon, France) according to the manufacturer's instructions. Samples were stored at −80°C for subsequent proteomic analyses.

The general workflow of proteomic analyses has been reported in supplementary data (Supplementary Figure 1). For proteome analysis, 80 μg total proteins from GC (three independent GC samples were pooled) and ffEVs (three independent ffEV preparations were pooled) obtained from 3–6-mm follicles were solubilized in SDS-PAGE sample buffer containing 4% SDS, 5% beta-mercaptoethanol, and 1.2 M Tris–HCl (pH 6.8) and boiled for 5 min at 95°C. Denatured samples were centrifuged at 10,000 × *g* for 5 min and fractionated on an 8–16% polyacrylamide gel (SDS-PAGE minigel 8.3 × 6 cm × 1.5 mm) at 120 V for 90 min. The SDS-PAGE gel stained with Coomassie blue solution and gel bands containing EV and GC samples were sectioned into 10 slices.

Each gel slice was washed in water and twice in 100% methanol/50 mM NH_4_HCO_3_ (1:1) for 1 min and rinsed with 100% acetonitrile/50 mM NH_4_HCO_3_ (1:1) for 5 min. Then, samples were dehydrated in 100% acetonitrile for 30 s and dried using a SPD1010 speedvac system (Thermosavant; Thermo Fisher Scientific, Bremen, Germany). Cystein reduction and alkylation were performed via successive incubations in 25 mM dithiothreitol/50 mM NH_4_HCO_3_ for 20 min at 56°C and 55 mM iodoacetamide/50 mM NH_4_HCO_3_ for 20 min in the dark at room temperature. Gel slices were washed in water and successively incubated in 50 mM NH_4_HCO_3_/acetonitrile (1:1) for 5 min and acetonitrile for 30 s, and dried. Proteins were digested in 25 mM ammonium hydrocarbonate that contained 12.5 ng/μl trypsin and incubated overnight. Peptide extractions were performed as previously described (36).

#### Nano LC-MS/MS Analysis

Nanoflow liquid chromatography–tandem mass spectrometry (nanoLC-MS/MS) analysis was performed using an LTQ Orbitrap Velos mass spectrometer (Thermo Fisher Scientific) coupled to an Ultimate 3000 RSLC Chromatographer (Dionex, Amsterdam, The Netherlands). Peptide extracts were injected on an Acclaim PepMap 100, LC Packings trap C18 column (75 μm inner diameter, 2 cm long, 3 μm particles, 100 Å pores) and separated using a Acclaim PepMap C18 nano-column (75 μm inner diameter, 50 cm long, 3 μm particles, 100 Å pores). Mobile phases consisted of (A) 98% water/2% acetonitrile in the presence of 0.1% formic acid and (B) 16% water/84% acetonitrile in the presence of 0.1% formic acid. The gradient consisted of 4–60% B for 90 min at a 300 nl/min flow rate. The instrument was operated in positive data-dependent mode with a resolution set to 60,000. In the scan range of *m*/*z* 300–1,800, the 20 most intense peptide ions with charge states ≥2 were sequentially isolated and fragmented using collision-induced dissociation. A lock mass was enabled for accurate mass measurements. Polydimethylcyclosiloxane [*m*/*z*, 445.1200025; (Si(CH_3_)_2_O)_6_] ions were used for the internal recalibration of the mass spectra.

#### Protein Identification and Validation

Protein data identification was performed using the Mascot search engine, version 2.3.2 (Matrix Science, London, United Kingdom), in combination with Proteome Discoverer 2.1 software (Thermo Fisher Scientific), against the mammal section of the non-redundant NCBI database (downloaded July 2018). Search parameters included trypsin as a protease with two missed cleavage sites allowed and with carbamidomethylcysteine addition, methionine oxidation, and acetylation of the N-terminal protein included as variable modifications. The tolerance of the ions was set to 5 ppm for the parent and 0.8 Da for fragment ion matches. Identified proteins were subjected to Scaffold v4.8.4 software (Proteome Software, Portland, United States) for validation. The identified identities of peptides and proteins were accepted if they could be established at a confidence score of >95% probability threshold, using the Prophet algorithm (37, 38). The correspondent percent decoy false discovery rate (FDR) was 0.4% regarding the number of proteins/clusters, and FDR was 0.02% for the number of spectra, which was in accordance with FDR values acceptable by Mass Spectrometry Data Interpretation Guidelines (<1%) (39). Proteins sharing similar peptide sequences were grouped into clusters. For each protein identified to be non-bovine, a manual blastp analysis against *Bos taurus* protein databases was performed to obtain a species-specific identification of the protein.

### Western Blot

Concentrated 4× Laemmli buffer was directly added to either ffEV or GC preparations, incubated on ice for 15 min, and heated for 8 min at 99°C. Protein lysates were centrifuged 5 min at 12,000 × *g*. Approximately 8 μg of each preparation was subjected to electrophoresis on a 4–12% acrylamide gel (Life Technologies, Saint-Aubin, France) and transferred to a 0.45-μm nitrocellulose membrane (Pall Corporation; VWR International, France). To verify the transfer, membranes were stained with Red Ponceau solution for 1 min, rinsed, and scanned. After washing with a TBS−0.1%/Tween-20 (TBST) solution, blocking was performed with 5% non-fat dry milk in TBST for 1 h at room temperature. Incubation with primary rabbit antibodies to human HSPA8 (1/1,000 dilution), RPS17 (1/1,000 dilution), RPS6 (1/500 dilution), or mouse antibodies to human vinculin (VCL) (1/2,000 dilution) was performed at 4°C overnight. Membranes were then washed using TBST and incubated with secondary horseradish peroxidase (HRP)-conjugated secondary antibodies (final dilution of 1:10,000) in TBST for 2 h at room temperature. After washing, the specific luminescence was revealed by chemiluminescent reagent ECL-Plus (Thermo-Fisher Scientific, Courtaboeuf, France). Signals were captured using a GeneGnome camera (Syngene, Cambridge, United Kingdom) and Genesys 1.5.4 software (Syngene).

### Gene Expression Analysis

To analyze bovine gene expression within theca cells (TH), GC, CC, and oocytes, we used transcriptome data (public repository: Gene Expression Omnibus, accession number GSE149151) previously obtained with a customized 60K Agilent bovine microarray, which covered 97% of Ensembl *Bos taurus* transcripts. These microarray expression data were validated via the real-time PCR of 10 different genes in a previous study (40). For the present study, we retrieved normalized expression values for genes coding proteins identified in ffEVs. For each gene, the median expression value of all spots was considered (at least two spots were present per gene in the microarray). The analysis of differential gene expression in follicular cells (TH, GC, CC, and oocytes) and a hierarchical classification were performed using normalized expression values of four biological replicates using XLSTAT software (Addinsoft, Paris, France). For differential analysis, ANOVA with Benjamini–Hochberg (BH)-corrected *p*-values was applied. Tukey test was used for multiple pair analyses. Differences at corrected values of *p* < 0.05 were considered significant.

RNA sequencing data obtained from the RNA content of bovine follicular fluid extracellular vesicles (16) were used to compare lists of the proteins identified in ffEVs and their coding mRNAs. Normalized RNAseq data were retrieved from GEO (accession number GSE96832), and transcripts with more than zero fragments per kilobase per million mapped reads (FPKM) were considered to be present in ffEVs (from a minimum of 0.186762 FPKM to a maximum of 2.53391 FPKM per gene loci).

### Functional Annotation of the ffEV Proteins

Lists of proteins identified in bovine ffEVs and GC in the study were compared with lists of proteins retrieved from the Vesiclepedia database, version 4.1 (http://microvesicles.org/index.html), which contained information regarding the molecular composition of EVs in different species (25, 26). The list of official symbols of ffEV proteins was compared with lists produced from either bovine or human EV-proteomes. In addition, the list of proteins identified here in bovine ffEVs was compared with lists of proteins recently reported in bovine oocyte and cumulus cells (41, 42).

Gene Ontology (GO) analyses were performed with FunRich software, version 3.1.3. (43), DAVID functional annotation bioinformatics microarray analysis (https://david.ncifcrf.gov/), and the STRING functional protein association network (https://string-db.org/). GO enrichment analysis was performed using a BH-corrected value of *p* < 0.05.

## Results

### Characterization of Extracellular Vesicles From Bovine Follicular Fluid and an Analysis of Their Uptake of by the COC

**Figure 1:**
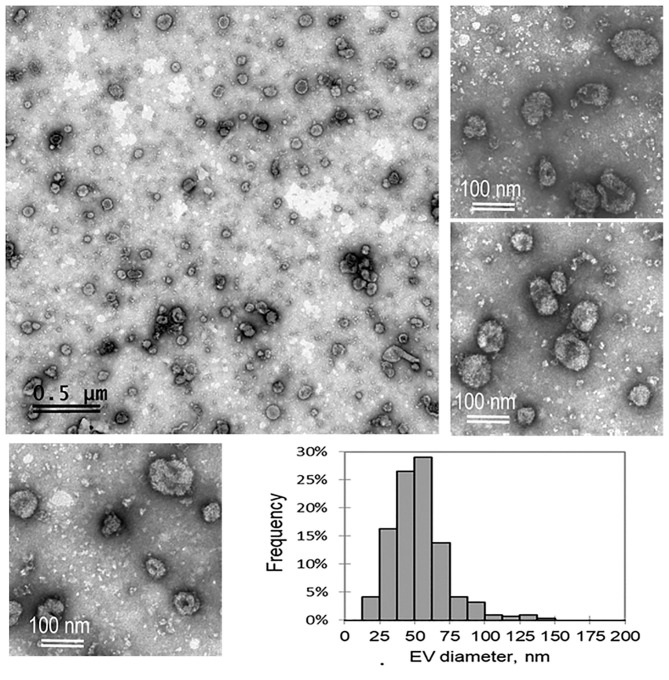
A representative transmission electron microscopy images of bovine exosome-like follicular fluid extracellular vesicles (ffEVs) derived from 3–6-mm ovarian bovine follicles and a histogram of the size distribution of ffEVs are shown.

Morphological characterization of ffEVs via TEM was performed to confirm the presence of exosomes obtained from FF after serial ultracentrifugations (Figure 1). All three ffEV preparations, taken for analysis, showed a population of small nanovesicles that resembled exosomes, mainly ranged in 40–100 nm size. The histogram shown in Figure 1 showed the vesicle size distribution of ffEV preparations. A mean diameter of 53.6 ± 23.3 nm from 313 measured vesicles was obtained. The percentage of ffEVs within a 100–150-nm range was 2.9% and the percentage within a 150–200-nm range was 0.3%.

Mass spectrometry proteomic data revealed the presence of several exosome markers within ffEV preparations, identified with 97–100% probability: tetraspanins CD81, CD9, and also cytosolic protein markers: annexins ANXA2 and ANXA5, and heat shock proteins HSPA6, HSP90-alpha, and HSPA8 (Supplementary Table 1).

**Figure 2:**
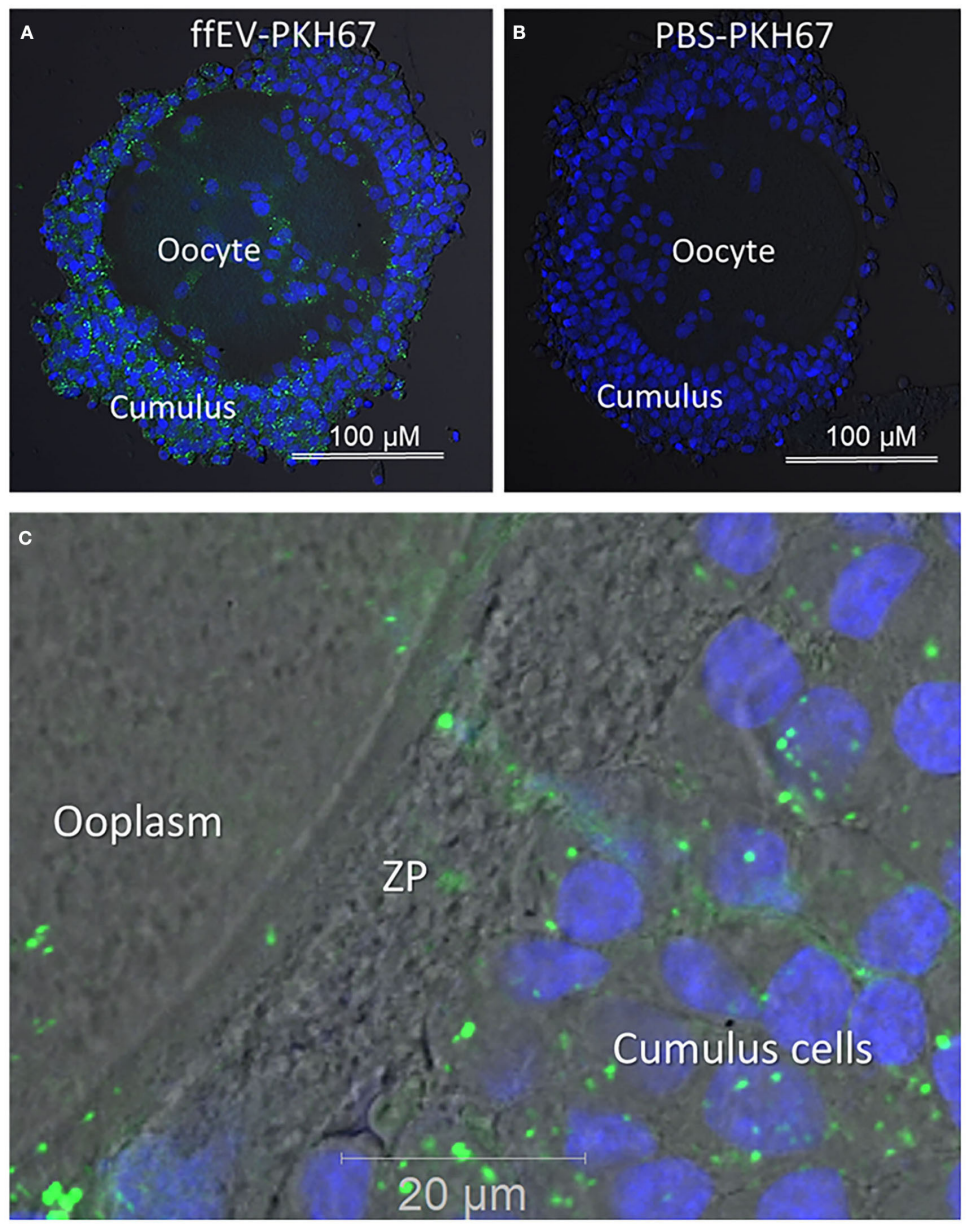
Uptake of follicular fluid extracellular vesicles (ffEVs) by cumulus–oocyte complexes during *in vitro* maturation is shown. **(A)** Representative confocal microscopy images of COCs after 24 h IVM in the presence of 50 μg/ml of labeled exosomes, ffEVs-PKH67, or **(B)** a similar volume of a control PBS-PKH67 preparation. **(C)** Fluorescent ffEVs were observed in the cytoplasm of cumulus cells, the zona pellucida (ZP), and ooplasm.

To demonstrate that COCs were able to internalize ffEVs during oocyte IVM, ffEVs were labeled with green fluorescent PKH67 dye and incubated with bovine COCs for 24 h. Confocal microscopy was used to confirm that ffEVs were internalized by COCs by detecting green fluorescent spots in the cytoplasm of CC, around the nucleus (Figure 2A). No green fluorescent EVs were observed in the negative controls, when COCs were incubated with control PKH67–PBS preparation (Figure 2B). Furthermore, we observed that green fluorescent ffEVs were located into zona pellucida and the periphery of the ooplasm (Figure 2C).

### Proteomic Analysis of Bovine Follicular Fluid Exosome-Like EVs and Comparison of ffEV Protein Cargo With Follicular Cell Proteins

**Figure 3:**
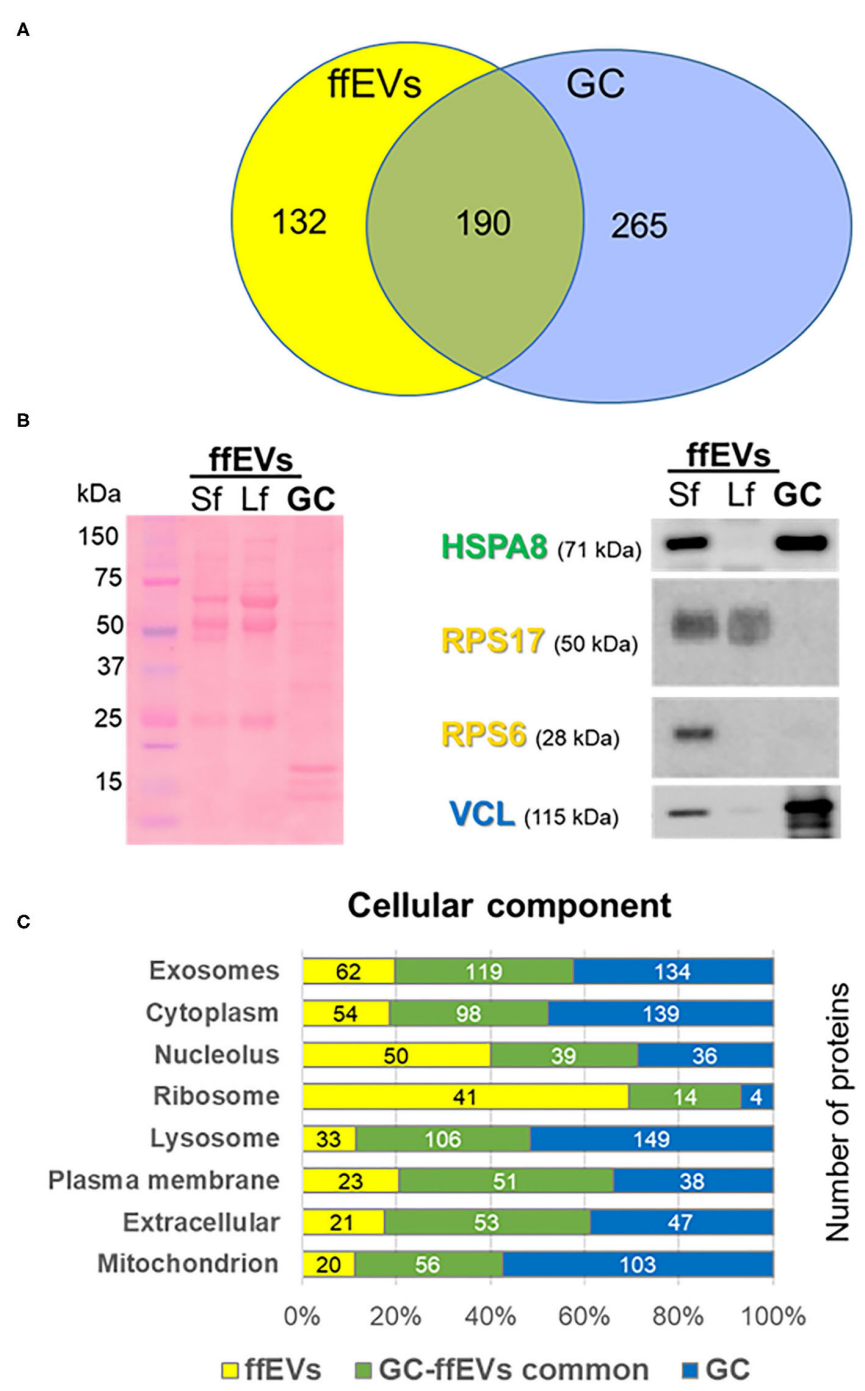
Comparative proteomic analysis of exosome-like extracellular vesicles from follicular fluid (ffEVs) and granulosa cells (GC). **(A)** Venn diagram was used to compare lists of proteins identified in bovine ffEVs and GC. A number of detected proteins and unique protein identifiers are shown. **(B)** Western blot analysis of proteins identified in ffEV and/or GC. Total levels of proteins within ffEVs of small follicles (Sf, 3–6 mm) and large follicles (Lf, >10 mm) as well as GC were tested via immunoblot using anti-HSPA8 (identified in EV/GC), anti-VCL (identified in GC), anti-RPS6, and anti-RPS17 (identified in ffEVs) antibodies. On the left, a representative picture of a red Ponceau-stained membrane after the electrotransfer of ffEV and GC proteins resolved by SDS-PAGE is shown. **(C)** Comparative analysis of cellular component GO terms enriched in proteins uniquely identified from ffEVs (ffEVs, yellow color bars), unique to GC (GC, blue color bars), or common to both ffEVs and GC (common ffEV-GC, green color bars). **(C)** Comparative analysis of biological pathways GO terms associated with proteins of ffEVs, EVs and GC, and GC alone.

Using nanoLC-MS/MS proteomics, 460 and 593 protein isoforms were identified in ffEVs and GC, respectively (Supplementary Table 1). All data are available in the PRoteomics IDEntification database (https://www.ebi.ac.uk/pride/) with an accession number of PXD020508. These isoforms represent 322 and 466 protein clusters, which include 322 proteins that were uniquely identified (protein ID) and 455 proteins in ffEVs and GC, respectively (Table 1). A comparison between the lists of the proteins identified in ffEVs and GC revealed 286 isoforms (190 proteins) of common proteins identified in ffEVs and GC, while 174 isoforms (132 proteins) and 307 isoforms (265 proteins) were identified only in ffEVs (EV-unique proteins) or GC (GC-unique proteins), respectively (Figure 3A). The complete lists of all the protein IDs identified in bovine ffEVs and GC (ffEV-unique, ffEV-GC-common, and GC-unique lists) are included in Supplementary Table 2.

**Table 1:** The number of proteins identified in bovine follicular fluid extracellular vesicles (ffEVs) and granulosa cells (GCs).

**Identified proteins**	**Protein isoforms**	**Protein clusters**	**Proteins (ID)**
ffEVs	460	322	322
GC	593	466	455
Common ffEV–GC	286	197	190

We compared the list of 322 protein IDs identified in ffEVs with the lists of proteins identified in bovine oocytes (1073 IDs) and CC (1547 IDs) after IVM reported by Marei et al. (41). Protein cargo of ffEVs shared 116 and 182 protein IDs with oocyte and CC, respectively.

More recent study by Gegenfurtner et al. (42) reported the identification of 1960 proteins in bovine oocyte (42). By comparing ffEV and oocyte lists of proteins, we found that 217 out of 322 ffEV proteins were also present in bovine oocytes.

In attempt to reveal whether ffEV proteins were found in other EV types, and to identify new potential cargo from other studies in bovine, we compared the lists of ffEV and GC proteins here identified with the lists of proteins identified in bovine and human EVs, which we retrieved from their respective Vesiclepedia databases (Table 2). In total, 100 out of 322 ffEV proteins identified here (30.2%) had previously been mentioned in the Vesiclepedia *Bos taurus* database. Moreover, 294 out of 322 ffEV protein IDs (91.4%) were present in *Homo sapiens* exosome-proteome data. In addition, of reported GC-unique proteins, 30.6 and 91.7% were identified in bovine and human EVs, respectively (Table 2).

**Table 2:** Presence of follicular fluid extracellular vesicle (ffEV) and granulosa cell (GC) proteins in Vesiclepedia databases.

**Identified proteins**		**Present in vesiclepedia databases**	
	**Proteins (ID)**	***Bos taurus***	***Homo sapiens***
	***n***	***n*** **(%)**	***n*** **(%)**
ffEV-unique	132	34 (25.8)	121 (91.7)
Common ffEV/GC	190	66 (34.7)	173 (91.1)
GC-unique	265	81 (30.6)	243 (91.7)

Our study identified 27 proteins in bovine ffEVs, which were not present in current bovine or human EV-proteome databases. These proteins included ACTL7, APMAP, CGN1, COLGALT1, FUSIP1, H2AZ1, H2BC19, H2BC21, HSD17B1, HSD3B2, IGHD, IGL, RACK1, SEPT2, SFRS9, TERT, TMEM35A, Vl1b, and several locus-positioned proteins, which mainly code for different IgGs.

To validate ffEV MS proteomic data, a Western blot analysis of selected proteins from bovine ffEVs isolated from FF from small follicles (Sf) 3–6 mm in diameter and large follicles (Lf) more than 10 mm in diameter, and on GC was performed (Figure 3B). HSPA8 was detected in both ffEV samples and within GC. Ribosomal proteins RPPS6 and RPS17 were detected exclusively in ffEVs. VCL was much more abundant in GC compared with ffEV samples. Interestingly, differences in the protein abundance of VCL, HSPA98, and RPS6 appeared between the ffEV preparations from Sf and Lf.

### GO Analysis of Proteins Identified in ffEVs and GC

First, the GO analysis of proteins identified in ffEVs and GC was performed using FunRich software. As shown in Figure 3C, the cellular component term “exosomes” was associated with 52.1 and 70.8% of ffEV and ffEV-GC proteins, respectively, and to 55.6% of GC-unique proteins. GC-unique proteins were more tightly associated with the term “lysosome” (61.8% of GC proteins, 63.1% of ffEV-GC, and 27.7% of EV-specific proteins). Similar proportions of GC-unique, ffEV-unique, and ffEV-GC groups were observed for the term “mitochondrion.” The term “ribosome” was mainly associated with proteins identified in ffEVs (41/132 and 14/190 identified in ffEVs and ffEV-GC protein lists, respectively), whereas only 4/265 proteins were identified exclusively from GC were associated with the ribosome. GO terms “cytoplasm,” “nucleus,” “plasma membrane,” and “extracellular” were relatively similarly associated with proteins derived exclusively from ffEVs or GC, and common to both ffEVs and GC. Protein-associated biological pathways including “Developmental biology,” “Metabolism of proteins,” and “Translation” were mainly identified in ffEVs compared with GC and both ffEVs and GC (Supplementary Figure 2). Similarly, ffEV-unique proteins were mainly involved in RNA metabolism, integrin interactions, estrogen receptor signaling, IFN-gamma pathway, and insulin pathways. Proteins, involved in TNF-related apoptosis inducing ligand (TRAIL), and vascular endothelial growth factor (VEGF) signaling pathways were more frequently identified in ffEVs than the GC. Molecular Function GO terms including “structural constituent of ribosome,” “RNA binding,” “extracellular matrix structure,” “transporter activity,” “receptor activity,” and “GTPase activity” were more highly represented in ffEV proteins (ffEV and ffEV-GC lists), whereas the GO term “catalytic activity” was more frequently observed in GC-unique proteins. Biological processes including “protein metabolism,” “transport,” “signal transduction,” “cell communication,” and the “immune response” were more frequently associated with ffEV-unique proteins than GC-unique ones, whereas “energy pathways” and “metabolism” were more tightly associated with GC-unique proteins than those of ffEVs (Supplementary Table 3).

**Figure 4:**
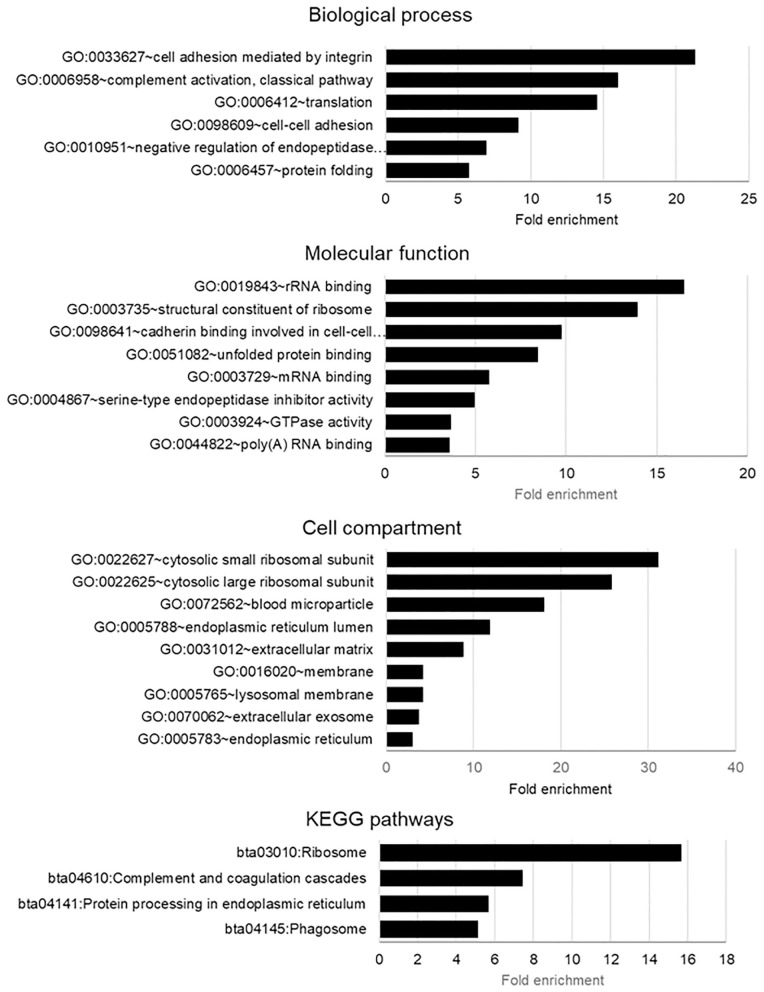
Gene Ontology (GO) enrichment biological processes, molecular functions, and biological pathway terms in genes encoding the proteins identified from exosome-like extracellular vesicles derived from bovine follicular fluid (ffEVs). The analysis was performed using the DAVID 6.8 application (https://david.ncifcrf.gov/home.jsp). Only significantly enriched GO terms are shown (Benjamini–Hochberg corrected values of *p* < 0.05 were considered significant).

To obtain more meaningful understanding of proteins identified in bovine ffEVs (ffEV and ffEV-GC lists), DAVID functional analysis was performed using a *Bos taurus* gene list as the background (Figure 4, Supplementary Table 4). This analysis showed that in ffEV proteins, biological process GO terms including “cell adhesion mediated by integrin” (21.3-fold), “complement activation” (16.0-fold), “translation” (14.6-fold), “cell-to-cell adhesion” (9.2-fold), “regulation of endopeptidase activity” (6.7-fold), and “protein-folding” (5.7-fold) were significantly enriched (*p* < 0.05). Regarding molecular function GO terms, “rRNA binding” (16.5-fold), “structural constituent of ribosome” (13.9-fold), “cadherin binding involved in cell-to cell communication” (9.7-fold), and “unfolded protein binding” (8.4-fold) were significantly enriched. In addition, “RNA-binding,” “endopeptidase activity,” and “GTPase activity” functions were significantly enriched (*p* < 0.05). Regarding Cell Compartment GO terms, those related to small and large ribosome subunits (31.2- and 25.8-fold, respectively), were most significantly enriched. Other enriched GO terms included “blood microparticles” (18.1-fold enrichment), “ER lumen” (11.9-fold), “extracellular matrix” (8.9-fold), “membrane” (4.2-fold), “lysosomal membrane” (4.2-fold), “extracellular exosome membrane” (3.8-fold), and endo (3.1-fold).

Different biological pathways associated with 322 proteins identified in ffEVs were analyzed using STRING software, and results of these analyses are shown in Table 3. The most significantly enriched KEGG pathway was related to term “Ribosome” and includes 46 ribosomal proteins (15.7-fold enrichment). Moreover, 26 ffEV proteins are related to “Protein processing in endoplasmic reticulum pathways” (5.7-fold enrichment), a list that includes seven heat shock proteins. Twenty-three proteins of the phagosome pathway (5.1-fold enrichment), 17 proteins involved in “Complement and coagulation cascades pathways” (7.5-fold enrichment), and 13 ffEV proteins related to the PI3K-Akt signaling pathway (COL6A1, HSP90AA1, HSP90AB1, HSP90B1, IGHM, ITGA2, ITGA6, ITGAV, ITGB1, ITGB3, RAC1, VTN, YWHAQ) were also identified. Other significantly enriched pathways included “Cholesterol metabolism,” “Carbon metabolism,” “Synaptic vesicle cycle,” “Spliceosome,” “Proteasome,” “Lysosome,” “Thyroid hormone synthesis,” “Antigen processing and presentation,” “Regulation of actin cytoskeleton,” “Endocytosis,” “Ferroptosis,” and “Necroptosis (Table 3).

**Table 3:** Biological pathway enrichment in bovine ffEV proteins via the analysis of functional protein association networks.

**# Term ID**	**KEGG pathways description**	**N proteins (ffEVs)**	**N proteins (background)**	**FDR**
bta03010	Ribosome	46	138	3.65e−41
MRPL3,RPL10,RPL10L,RPL11,RPL13,RPL14,RPL15,RPL18,RPL18A,RPL19,RPL21,RPL23,RPL23A,RPL26RPL27,RPL27A,RPL30,RPL32,RPL35,RPL35A,RPL38, RPL4,RPL5,RPL6,RPL7,RPL7A,RPL8,RPLP0,RPS10,RPS11,RPS13,RPS14,RPS17,RPS18,RPS2,RPS23,RPS24,RPS27A,RPS3, RPS3A,RPS4Y1,RPS5,RPS7,RPS8, RPS9,RPSA				
bta04141	Protein processing in endoplasmic reticulum	26	160	4.12e−16
CALR,CANX,CKAP4,DAD1,DDOST,ERP29,GANAB,HSP90AA1,HSP90AB1,HSP90B1,HSPA5,HSPA6,HSPA8,LMAN1,LMAN2,PDIA3,PDIA4,PDIA6,PRKCSH, RPN1,RPN2,SEC24C,SEC61A1,SSR4,UGGT1,VCP				
bta04145	Phagosome	23	155	1.43e−13
ATP6V0D1,ATP6V1E1,C3,CALR,CANX,CGN1,DYNC1I2,IGHM,ITGA2,ITGAV,ITGB1,ITGB3,LAMP2,M6PR,MRC2,RAB7A,RAC1,SEC22B,SEC61A1,STX7, TFRC,TUBA1C,TUBB2A				
bta04610	Complement and coagulation cascades	17	82	4.38e−12
A2M,C1QB,C3,C4A,C5,C8G,C9,CFH,CFI,CLU,F2,FGA,FGB,FGG,PLG,SERPIND1,VTN				
bta04979	Cholesterol metabolism	8	46	3.86e−05
APOA1,APOA4,APOE,LRPAP1,SORT1,VDAC1,VDAC2,VDAC3				
bta04216	Ferroptosis	7	40	0.00013
CP,FTL,PCBP1,TF,TFRC,VDAC2,VDAC3				
bta04918	Thyroid hormone synthesis	8	70	0.00043
ALB,ATP1A1,CANX,GPX8,HSP90B1,HSPA5,PDIA4,TTR				
bta04721	Synaptic vesicle cycle	7	59	0.0010
AP2A1,AP2B1,ATP6V0D1,ATP6V1E1,CLTA,CLTC,NAPA				
bta03040	Spliceosome	9	125	0.0028
HNRNPA3,HNRNPU,HSPA6,HSPA8,PCBP1,SFRS13A,SRSF7,SRSF9,TRA2B				
bta04612	Antigen processing and presentation	7	76	0.0032
CALR,CANX,HSP90AA1,HSP90AB1,HSPA6,HSPA8,PDIA3				
bta03050	Proteasome	5	45	0.0090
PSMA1,PSMA5,PSMA6,PSMC6,PSMD2				
bta04810	Regulation of actin cytoskeleton	10	192	0.0090
F2,GSN,ITGA2,ITGA6,ITGAV,ITGB1,ITGB3,MYL12A,PFN2,RAC1				
bta01200	Carbon metabolism	7	108	0.0142
ADPGK,GAPDH,GOT2,MDH2,PGD,PGK1,PRPS1L1				
bta04142	Lysosome	7	116	0.0183
ATP6V0D1,CLTA,CLTC,CTSD,LAMP2,M6PR,SORT1				
bta04144	Endocytosis	10	231	0.0208
AP2A1,AP2B1,ARF1,CLTA,CLTC,HSPA6,HSPA8,RAB11A,RAB7A,TFRC				
bta04151	PI3K-Akt signaling pathway	13	352	0.0208
COL6A1,HSP90AA1,HSP90AB1,HSP90B1,IGHM,ITGA2,ITGA6,ITGAV,ITGB1,ITGB3,RAC1,VTN,YWHAQ				
bta04217	Necroptosis	7	152	0.0498
FTL,H2AFV,HSP90AA1,HSP90AB1,VDAC1,VDAC2,VDAC3				

### Comparison of Protein and Correspondent mRNA Content in Bovine ffEVs

*In silico* analysis of RNA sequencing data obtained on bovine FF exosomes (GEO accession GSE96832) (16) allowed us to map 3,966 genes coding for mRNAs detected in ffEVs. A comparison of expressed mRNAs with the list of 322 proteins detected in ffEVs revealed 59 commonly identified proteins and transcripts (Table 4), indicating that ffEVs contain mRNAs that account for 18.3% of the proteins detected within ffEVs.

**Table 4:** Transcripts encoding ffEV proteins that were detected in bovine ffEVs.

**Gene**	**Description**	**FPKM**
ACTL7A	*Bos taurus* actin-like 7A	1.36222
ADPGK	*Bos taurus* ADP-dependent glucokinase	0.238912
C1QB	*Bos taurus* complement component 1, q subcomponent, B chain	1.09207
CFI	*Bos taurus* complement factor I	0.493228
CPD	Carboxypeptidase D	0.186762
EEF1A2	*Bos taurus* eukaryotic translation elongation factor 1 alpha 2	0.584246
EEF1G	*Bos taurus* eukaryotic translation elongation factor 1 gamma	0.874273
EFCAB3	EF-hand calcium binding domain 3	0.369745
ERP29	*Bos taurus* endoplasmic reticulum protein 29	0.50338
FGG	*Bos taurus* fibrinogen gamma chain	0.601315
FKBP11	*Bos taurus* FK506 binding protein 11	1.16161
HIST1H1D	*Bos taurus* histone cluster 1	0.169719
HRG	*Bos taurus* histidine-rich glycoprotein	0.626686
HSP90AB1	*Bos taurus* heat shock protein 90 kDa alpha	0.572244
HSPA6	Heat shock 70 kDa protein 6	0.964551
IER3IP1	*Bos taurus* immediate early response 3 interacting protein 1	0.447852
IGHM	Immunoglobulin heavy constant mu	0.465297
IGSF8	*Bos taurus* immunoglobulin superfamily, member 8 (IGSF8)	0.66334
M6PR	*Bos taurus* mannose-6-phosphate receptor (cation dependent)	0.513152
MIF	*Bos taurus* macrophage migration inhibitory factor	2.53391
MYL6	*Bos taurus* myosin, light chain 6	5.03815
PCBP1	*Bos taurus* poly(rC) binding protein 1	0.421419
PFN2	*Bos taurus* profilin 2	0.306297
PGRMC2	*Bos taurus* progesterone receptor membrane component 2	0.565112
PRPS1L1	*Bos taurus* phosphoribosyl pyrophosphate synthetase 1-like 1	0.921892
PRPSAP1	*Bos taurus* phosphoribosyl pyrophosphate synthetase-associated protein 1	0.944507
PSMA1	*Bos taurus* proteasome (prosome, macropain) subunit, alpha type, 1	0.53781
RAB1A	*Bos taurus* RAB1A, member RAS oncogene family	0.835257
RAB7A	RAB7A, member RAS oncogene family	0.657846
RAC1	*Bos taurus* ribonuclease, RNase A family, 1	1.21942
RPL10	*Bos taurus* ribosomal protein L10	0.963056
RPL15	*Bos taurus* ribosomal protein L15	0.943742
RPL17	*Bos taurus* ribosomal protein L17	1.65646
RPL19	*Bos taurus* ribosomal protein L19	2.20665
RPL23A	*Bos taurus* ribosomal protein L23a	2.21847
RPL27A	*Bos taurus* ribosomal protein L27a	3.17647
RPL35	*Bos taurus* ribosomal protein L35	1.94657
RPL35A	*Bos taurus* ribosomal protein L35a	1.39589
RPL36	60S ribosomal protein L36	2.63049
RPL38	Ribosomal protein L38	2.9163
RPL4	*Bos taurus* ribosomal protein L4	0.745345
RPS10	*Bos taurus* ribosomal protein S10	4.49435
RPS15A	*Bos taurus* ribosomal protein S15A	2.70663
RPS16	Ribosomal protein S16	2.19883
RPS26	Ribosomal protein S26	1.8005
RPS27L	*Bos taurus* ribosomal protein S27-like	1.31883
RPS6	*Bos taurus* ribosomal protein S6	1.11923
RPSA	*Bos taurus* ribosomal protein SA	0.62304
SCAMP2	*Bos taurus* secretory carrier membrane protein 2	0.417959
SERPINH1	*Bos taurus* serpin peptidase inhibitor, clade H	0.309656
SFRS9	*Bos taurus* serine/arginine-rich splicing factor 9	1.63843
SRGN	Serglycin	0.531369
STX6	*Bos taurus* syntaxin 6	1.24533
TMED10	Transmembrane emp24-like trafficking protein 10	0.78312
TMED4	*Bos taurus* transmembrane emp24 protein transport domain containing 4	0.640647
TRA2B	*Bos taurus* transformer 2 beta homolog	0.441172
VDAC1	*Bos taurus* voltage-dependent anion channel 1	1.3177
VDAC2	*Bos taurus* voltage-dependent anion channel 2	1.12455
VTN	*Bos taurus* vitronectin	0.38606

**Figure 5:**
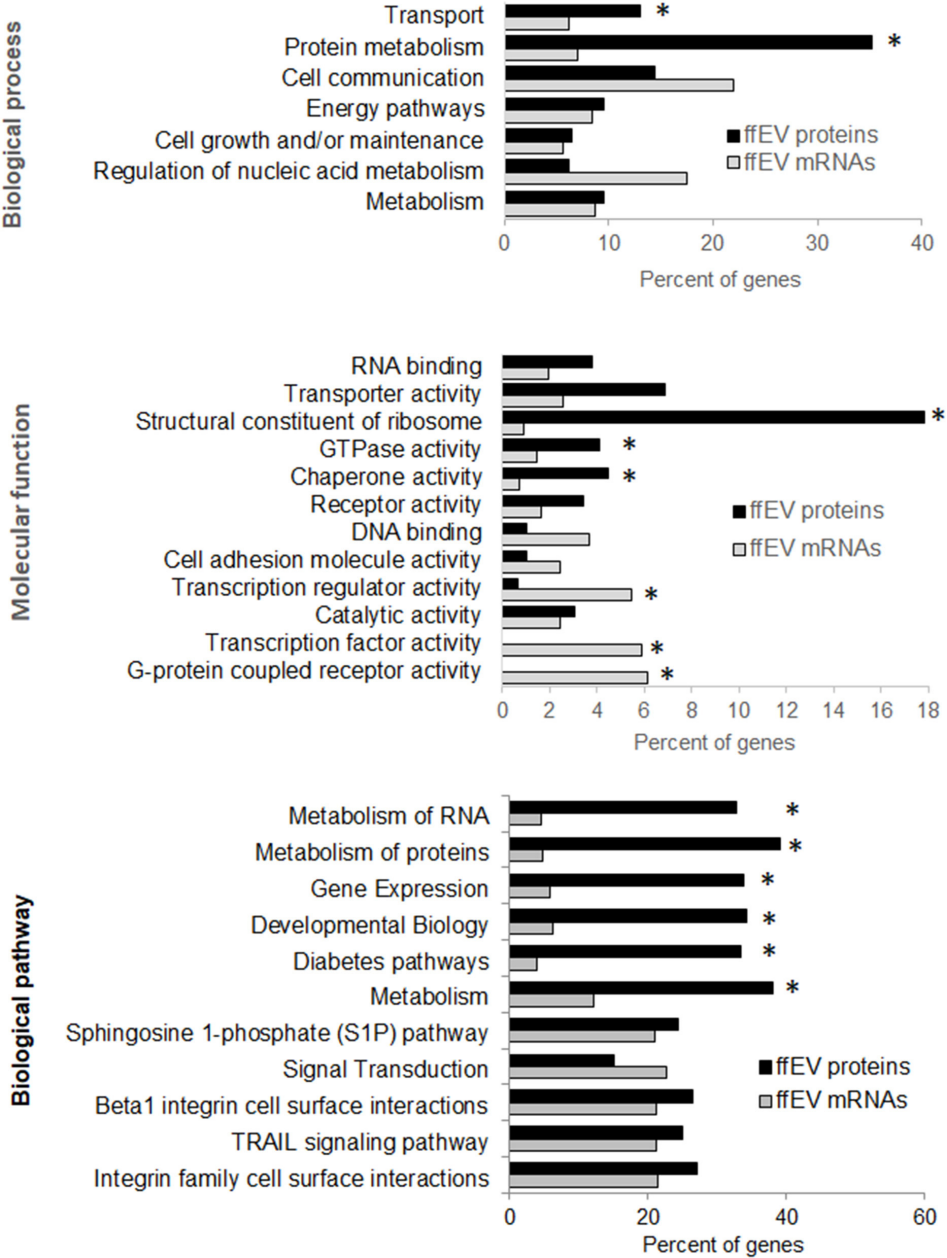
Comparative analysis of Gene Ontology (GO) enrichment terms identified from lists of 322 proteins and 3,466 mRNAs that were identified from bovine ffEVs. Asterisks mark significant differences in GO term enrichment (*p* < 0.05).

Analyses of the most representative biological process, molecular function, and biological pathway GO terms were performed using 3,466 mRNAs and 322 bovine ffEV proteins, which revealed differences and similarities (Figure 5). Biological process terms including “transport” and “protein metabolism” and pathway terms including “structural constituent of ribosome,” “GTPase activity” and “chaperon activity” molecular functions, and “RNA metabolism,” “protein metabolism,” “gene expression,” “developmental biology,” and “diabetes” were significantly enriched in ffEV proteins vs. mRNAs. Other GO terms were either similarly associated with proteins and mRNAs or, like “transcription factor activity” and “G-coupled receptor activity” molecular functions, were more enriched in the ffEV-mRNA list.

### Gene Expression Analysis of ffEV Proteins Identified in Bovine Ovarian Follicular Cells

**Figure 6:**
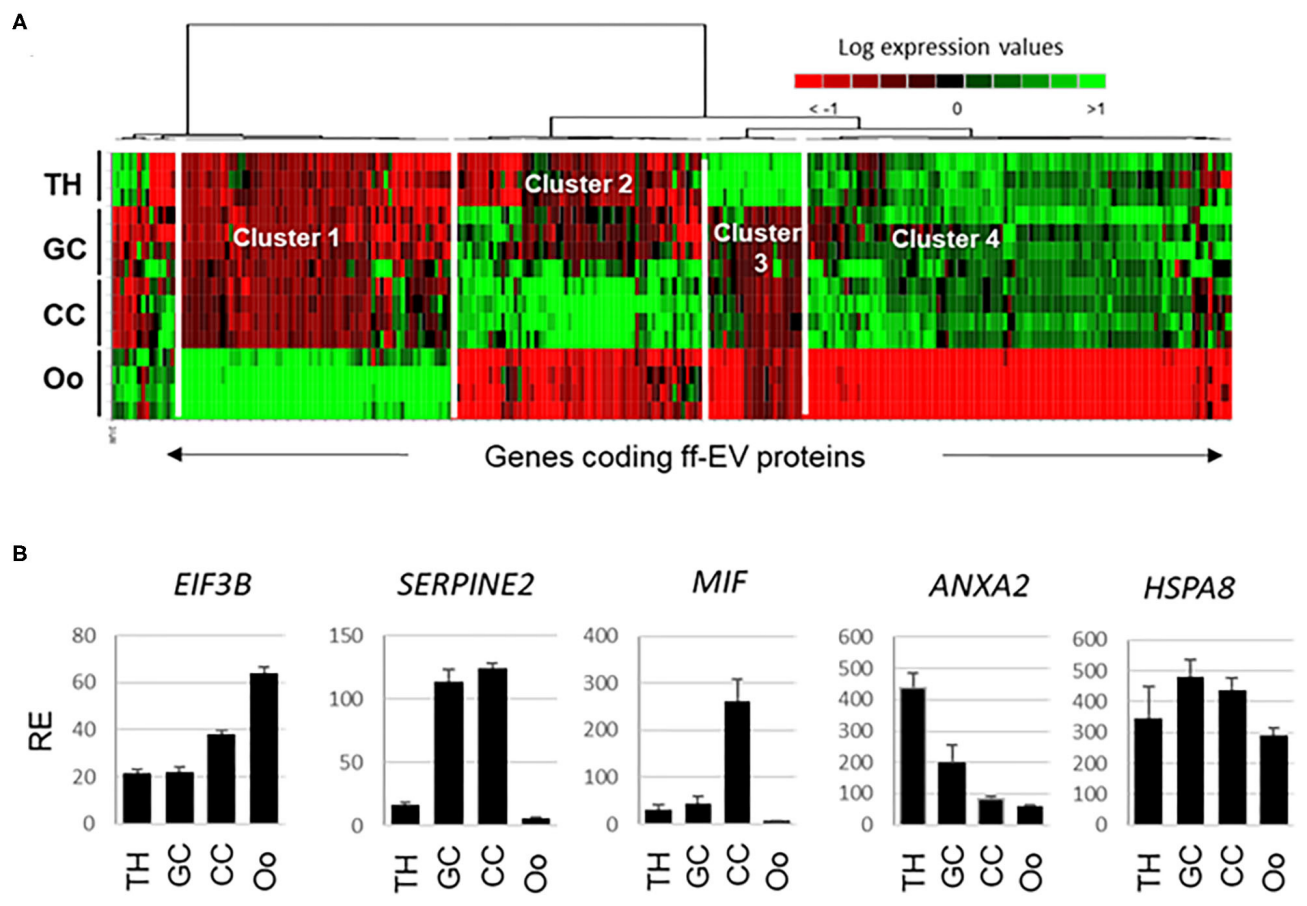
Integrative transcriptomic analysis of genes encoding proteins identified from ffEVs that were determined to be differentially expressed in different bovine follicular cell types including theca cells (THs), granulosa cells (GC), cumulus cells (CC), and oocytes (OOs). **(A)** Heat map representation of genes encoding proteins identified in ffEVs. **(B)** Examples of genes from each cluster of the heatmap are shown.

In an attempt to uncover the origin of ffEV proteins, the expression of corresponding coding genes was evaluated using previously obtained bovine follicular cell transcriptome data, which included the TH, GC, CC, and oocytes (GEO accession GSE149151) (40). Gene expression values for genes coding 267 ffEV-identified proteins could be retrieved. Statistical analysis revealed that 239 genes were differently expressed in at least one compartment relative to others (ANOVA, BH-corrected *p* < 0.05). All the data have been included in Supplementary Table 5. Several genes displayed similar gene expression values in all cells (*HSPA8, ITGB1, HSP90B1, EIF3A, AP2B1*, etc.). Normalized gene expression values were submitted to hierarchical clustering and presented as a heatmap (Figure 6A).

The heatmap clearly shows four different clusters of gene expression patterns. Cluster 1 represents the genes that were overexpressed in the oocyte, including *EIF3B* (shown in Figure 6B), *SCAMP2, SCAMP3, RBP4, GNA11, MLEC, MGP*, and others. Cluster 2 includes genes overexpressed in GC and CC, including as *SERPINE2* (Figure 6). *GPX1, ITGA6, BLVRA, IFSF8, ITGA2*, and others were overexpressed in CC in a manner that was similar to *MIF* (Figure 6B). Cluster 3 contains genes overexpressed in the TH layer, for example, *ANXA2* (Figure 6B), *FN1, C1QB, APOA1, CD9*, and *IGLL1*. Cluster 4 is the largest cluster and contains genes that were more highly expressed in all somatic cells (TH, GC, and CC) than the oocyte. For example, all the 57 ribosomal protein genes were included in this cluster, as well as *VIM, CAD, AP2A1, SERPINH1, GSTA1*, and others.

### Ribosomal Proteins in ffEVs and Expression of Corresponding Genes

**Figure 7:**
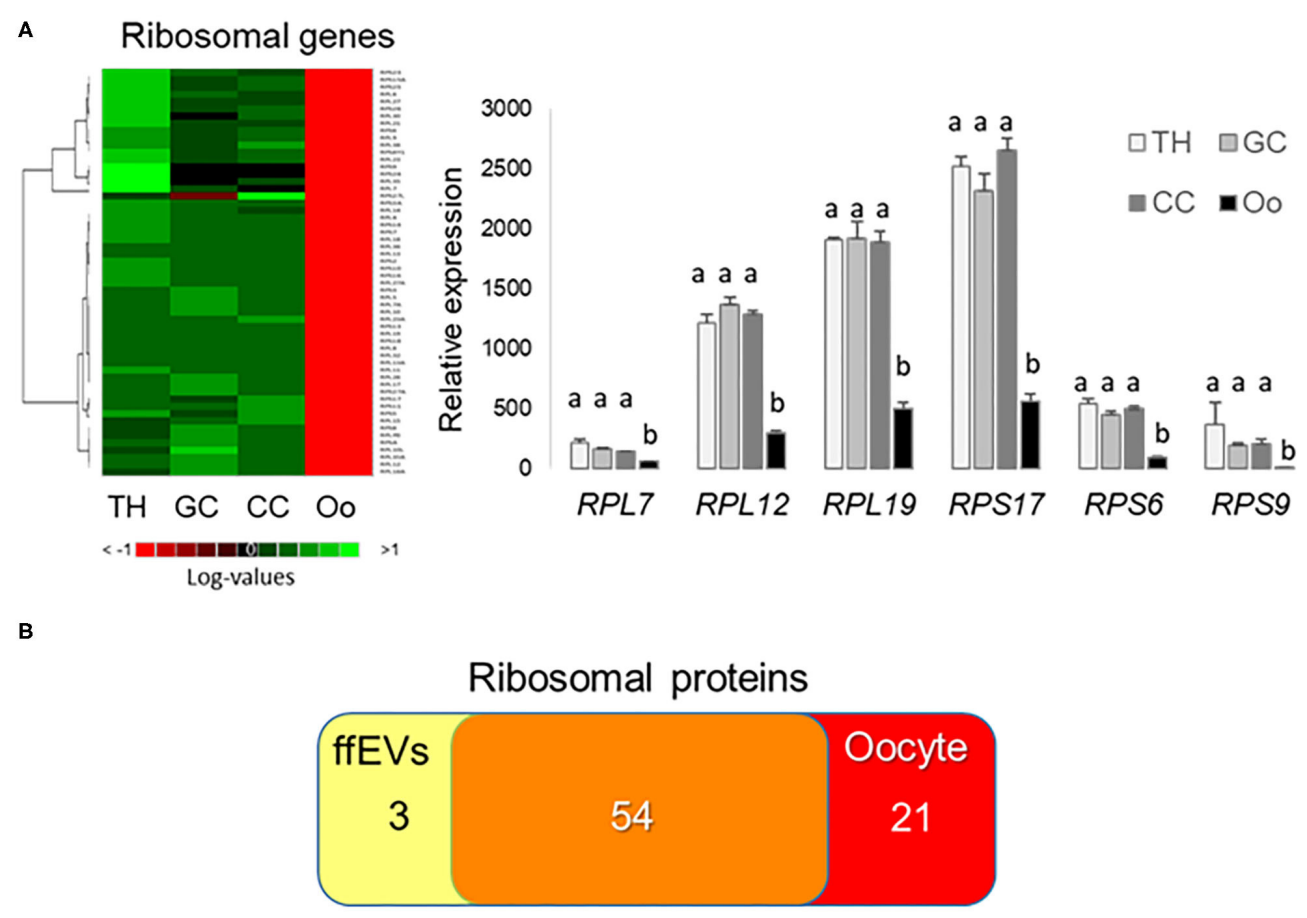
Ribosomal proteins in follicular fluid exosome-like extracellular vesicles (ffEVs) and expression of corresponding genes. **(A)** Gene expression levels of small and large subunits ribosomal proteins in follicular theca cells (TH), granulosa cells (GC), cumulus cells (CC), and oocytes (OO). The heatmap represents log expression values of small and large subunit ribosome genes from different follicular cell types that were also identified in ffEVs. The graph includes mean ± SEM expression values for selected ribosomal proteins from four independent replicates. Different letters indicate differences at a significance level of *p* < 0.05. **(B)** Venn diagram representing a number of ribosomal proteins identified here in ffEVs and in bovine oocytes (42).

Relative expression values for genes encoding small and large subunit ribosomal proteins in TH, GC, CC, and the oocyte are presented as a heatmap and histograms (Figure 7A). Interestingly, marked differences in gene expression values were observed between the oocyte and follicular cells for all the ribosomal proteins detected in ffEVs, and all ribosomal genes showed the lowest mRNA abundance in oocyte (Figure 7A, Supplementary Table 5). At protein level, 57 small and large ribosome subunit proteins were identified in ffEVs, and 54 of them were common to list of 75 ribosomal proteins identified in bovine oocytes (42) as shown by Venn diagram (Figure 7B).

## Discussion

Our study includes the first extensive characterization of protein cargo of bovine ffEVs isolated from small follicles (3–6 mm), which have been shown to improve oocyte developmental competence *in vitro*. Additional analysis of the follicular GC proteome revealed that it shared 41% protein identity with the ffEV protein cargo. Further, an integrative analysis of RNA expression profiles of genes encoding ffEV proteins in different types of follicular cells as well as the mRNA content of ffEVs have provided new clues regarding the diverse origin of EVs present in bovine FF.

To characterize ffEV preparations morphologically, molecularly, and functionally, TEM observations, mass spectrometry analysis, and ffEV uptake assays were performed, respectively. TEM observations revealed that the majority of ffEVs in our preparations could be considered exosome-like EVs because their size was similar to that which had previously been described for bovine FF exosomes (14–16, 23). In addition, ffEVs possessed exosome protein markers, which were formerly identified in ffEV preparations by mass spectrometry. Taken together, these observations confirmed the exosome-like nature of our preparations. The percentage of ffEVs larger than 100 nm was very low (3%), suggesting that only a very small proportion of EVs in ffFF preparations could be associated to the presence of microvesicles.

Functionally, an analysis of ffEV uptake by COCs *in vitro* demonstrated that prepared ffEVs were internalized by these cells. Although the mechanisms of molecular exosome-directed exchange between the oocyte and CC have not been well-studied, the possible involvement of nanovesicles in the rapid transport of large molecules from CC to the oocyte via trans-zonal projections was previously demonstrated in bovine COCs (3). Indeed, in our study, we have observed labeled ffEVs in the cytoplasm of CC, some projections in the zona pellucida, and faint labeling of the periphery of the ooplasm. These findings are similar to those reported by da Silveira et al. (16), which revealed the presence of labeled exosomes that could be observed in CC and zona projections but were not observable 9 h post-IVM. The localization of ffEVs may suggest that once they are transferred to the ooplasm, ffEVs that reached their targets may be desorbed *in situ*, thus reducing their levels of fluorescence. Another hypothesis is that ffEVs released their cargo within CC, which assure further transport of the molecules to enclosed oocyte through gap junctions.

In summary, results here were in accordance with previous studies because they showed that exosome-like EVs from bovine FF may be internalized by COCs during IVM and, therefore, functionally affect follicular cells and the oocyte [(8, 15, 16, 18).

### Protein Characterization of Bovine ffEVs

The findings of the present study enhance our knowledge of bovine ffEV protein cargo. Indeed, until now, EV-proteomic studies in bovine have been limited to exosomes derived from milk (44) plasma, saliva, urine (27, 28), oviduct fluid (10), and spent medium containing ovarian cell secretions (45). Here, we identified 322 proteins in ffEVs. Among these, only 30% had previously been detected in bovine EVs but more than 91% of ffEV proteins overlapped with annotations in the Vesiclepedia human EV-proteome database (26). Furthermore, according to our data, 59% of the proteins identified in ffEVs were also included in the GC proteome, and more than half of the GC proteins identified were also associated with the GO term “Exosome”. Moreover, more than 91% of GC-unique proteins also overlapped with human EV-proteomes. Taking in account published oocyte and CC proteomic analysis (41, 42) and our data, it may be suggested that a significant part of ffEVs likely originated from GC and also from CC and oocyte. Hence, ffEVs may have diverse origins because around 40% of the proteins identified in ffEVs were neither detected in GC nor in CC (41).

Proteomic analyses of ffEVs in equine (20), porcine (46), and human (29) identified 73, 249, and 662 proteins, respectively. Comparisons of the protein cargo of bovine ffEVs (322 protein IDs) with other species revealed similarities. Of the 42 proteins detected in mare FF (47), 17 proteins including A2M, ALB, ANXA2, ANXA4, ANXA5, APOA1, APOA4, CLU, CP, FGA, FGB, FGG, FN1, GAPDH, HRG, TTR, and VTN were also identified in this study. Notably, da Silveira et al. (20) identified several apolipoproteins, immunoglobulins, fibrinogens, and complement components from ffEVs isolated from the follicles of young mares that we also identified in preparations of bovine ffEVs. In human ffEVs, 86 proteins were differentially expressed in patients with polycystic ovary syndrome relative to controls (29), some of which were also identified in our study (APMAP, PRDX6, isocitrate and malate dehydrogenases, etc). However, the average size of human ffEVs was determined to be larger than that of our preparations (100 nm and 53 nm for human and bovine ffEVs, respectively) (29).

Very recently, the proteome of porcine ffEVs from sexually matured gilts was analyzed, and 249 proteins involved in binding functions (41.4%), catalytic activity (37.1%), transporter activity (6.9%), translational and transcriptional regulation, the extracellular matrix, and the cytoskeleton were identified (46). However, when we compared the reported molecular functions of porcine ffEV proteins with those of our study, both similarities and differences were observed. Because the average size of porcine ffEVs reported by the authors was 122.6 nm for 3–7-mm follicles, the ffEV preparations used in the study likely contained a greater proportion of large vesicles (microvesicles, >100 nm in size) than our bovine ffEV preparations. These differences may have resulted from different protocols that were employed for the isolation of ffEVs, the follicular origin of FF, and species differences, among the other factors.

### Functional Significance of ffEV Proteins

Proteins identified from bovine ffEVs are known to be involved in numerous processes, which have been associated with follicular homeostasis, the development of the enclosed oocyte, and signaling pathways. GO analysis of biological processes, molecular functions, and pathways associated with ffEV proteins revealed numerous commonly enriched GO terms. In fact, protein and RNA metabolism, response to stimulus, transport, extracellular matrix, focal adhesion, and protein secreting/folding GO terms were enriched in milk, oviduct, follicular fluid, and different human cell line exosomes (34, 44, 46, 48). Pathways related to developmental biology processes, including PI3K-Akt, insulin, interferon gamma, and EGFR signaling and immune response were associated with ffEV and not CG proteins. Further, these pathways were previously associated with the miRNA cargo of ffEVs (21, 23, 24).

Numerous bovine ffEV proteins were associated with protein processing in the endoplasmic reticulum, which is a basic process needed for cell survival in which the synthesis, folding, post-translational modification, transport, and sorting of proteins and some lipids occur. Endoplasmic reticulum–stressed epithelial ovarian cancer cells released exosomes that delivered miRNA to macrophages, which in turn promoted tumor cell proliferation and migration (49). In humans, FF cells are 5–15% macrophages (50) and are likely ffEV target cells capable of regulating follicular cell functions including GC proliferation (14).

The enrichment of proteins associated with endocytosis, ferroptosis, phagosomes, and lysosome pathways indicated that ffEVs might participate in the processes of removing dying and infected cells from inside the follicle, and thus the maintenance of follicular homeostasis vs. atresia, as was previously shown in the *Drosophila* ovary (51). In addition, several proteasome subunit proteins (PSMA1, PSMA5, PSMA6, PSMC6, PSMD2) were also identified in bovine ffEVs. An abundance of 20S and 26S proteasome proteins was also detected in exosomes secreted by tumor-associated macrophages (52), and it was shown that the 20S proteasome was associated with mesenchymal stem cell exosomes (53). Proteasomes are involved in protein degradation and adaptive immunity, which are processes that are essential for the regulation of the cell cycle, gene expression, and the oxidative stress response (54). All these processes are highly important for follicles entering the dominant phase needed to prepare for ovulation (55). Moreover, ubiquitin-mediated proteolysis was the most enriched pathway associated with genes predicted to be targeted by miRNAs, which were highly abundant in FF exosomes (21). However, more studies will be needed to determine whether ffEVs contain functional proteasomes.

In addition, according to our analysis, the mRNA cargo of bovine ffEVs includes transcripts of <20% of ffEV proteins. A comparison of functional terms revealed that mRNAs and proteins in ffEVs were associated with both similar and different pathways. These data prompted us to question whether different roles may exist for mRNAs and proteins present in ffEVs. Whether mRNAs that were detected in ffEVs have regulatory functions similar to miRNA (23, 24) or if they are destined to be degraded/translated requires further investigation.

### Ribosome ffEV Constituents

Enrichment analysis of all identified ffEV proteins revealed that GO terms related to RNA binding, translation, and structural constituents of ribosome were significantly overrepresented. Indeed, in our study, 57 ribosomal proteins and several other RBPs were identified in bovine ffEVs. Interestingly, most of these proteins were unique to ffEVs and were not here detected in GC, which means significant enrichment of ribosomal proteins in ffEVs. However, 54 of ffEV ribosomal proteins were identified in bovine oocytes (42). Previous studies demonstrated that EVs released from different cells contain a substantial amount of coding and non-coding RNAs. This finding was consistent with the high quantity of RBPs that were found in EVs, which were often enriched compared with other protein classes (48). Some RNA types arrived to recipient cells in intact and functional forms and had the potential to modulate gene expression and function; however, other transcripts may be non-functional degradation products [reviewed in O'Brien et al. (56)]. RNA packaging in exosomes can occur through different mechanisms including RNA recognition via a number of RBPs, which can account for ~25% of EV protein content (48, 57). This is in accordance with proteomic analyses of EVs from human embryonic kidney cells and mouse myoblasts, which revealed enrichments in ribosome and translation-related proteins (48), findings that are similar that which was determined for the ffEV protein cargo. In reproductive fluids, Almiñana et al. showed that mRNAs encoding ribosomal proteins of the large (RPL) and small subunit (RPS) were the most abundant protein-coding RNAs within oviductal EVs (34). These results are in line with studies in EVs from other body fluids including saliva (58) and urine (59) It is possible that the numerous ribosomal proteins identified in our ffEV preparations, and those identified by others, may form RNA–ribonucleoprotein complexes that function to package RNAs into EVs and, thus, participate in RNA maintenance (57) or RNA degradation in exosomes. The presence of numerous RBPs and ribosomal proteins in ffEVs may suggest a possible role for the proteins in RNA sorting, which was also reported in EVs of different origins (60). On the other hand, an analysis of different types of EVs in biological fluids demonstrated that many RBPs were not associated with classical exosomes displaying CD9, CD81, and CD63 markers, and were instead linked to other EV subtypes (61). This finding suggested the presence of different subpopulations of exosome-like ffEVs. In addition, the presence of ribosomal subunits within EVs was also observed in liquid biopsies of a variety of cancer cells (62). Despite numerous studies that have shown that ribosomal proteins represent a large percentage of EV cargo (34, 48, 52), their functional roles remain unclear.

Interestingly, among transcripts encoding ffEV proteins that were detected in FF exosomes via RNA-seq data (16), 18 mRNAs encoded ribosomal proteins. Several of these mRNAs were among 30 of the most abundant transcripts identified from porcine ffEVs (63). In the study, it was demonstrated that porcine ffEVs contain full-length mRNAs, which may be translated into ribosomal proteins and modulate biological processes in recipient cells (63). Therefore, the presence of both ribosomal subunit transcripts and proteins in ffEVs suggests that the transfer of the constituents of ribosomes plays an important role in cell-to-cell communication within the follicle. Whether these ribosome-related factors are designated to the oocyte, which displayed limited expression of ribosomal genes and may differ from somatic cells ribosomal RNAs (64), but contained 75 ribosomal RPS and RPL proteins including 54 common proteins to ffEVs (42), requires further investigation.

### Different Origins of Bovine ffEVs

All cell types are capable of releasing EVs, but their cargo may be cell specific, and all cells present in a follicle (mainly GC, but also TH, CC, and also the oocyte) may release EVs into the FF. Therefore, we hypothesized that ffEVs may be composed of a mixture of EVs of different origins. Analyzing the origin of ffEVs will provide valuable information that may clarify the potential role of ffEVs in the follicle and their functional impact on the oocyte. In an attempt to investigate the cell origin of the ffEVs, we considered two approaches: (1) by trying to correlate gene expression in the follicular cells with protein cargo of the ffEVs; (2) by comparing the proteins in follicular cells and ffEVs. However, both approaches have limitations. Thus, gene expression does not always correlate with protein expression. Regarding proteome analyses, the proteins identified in the ffEVs are secreted ones, which usually are enriched in EVs compared with the cells, so it is possible that they are not sufficiently abundant in the cells to be detected by mass spectrometry or Western blot. Previous data from our laboratory has shown that in somatic follicular cells as CC, expression patterns of mRNAs and encoded total proteins (without post-translational modifications) are globally correlated (65–68). Thus, we considered that relative abundance of mRNAs may reflect protein relative expression level and performed a comparative analysis of the expression levels of genes encoding proteins identified from bovine ffEVs in GC, TH, CC, and the oocyte using previously obtained transcriptomic data (40).

Our transcriptomic analysis revealed that 83% of the genes encoding ffEV proteins expressed in different follicular cells types (TH, GC, CC, and also the oocyte). Interestingly, numerous genes were more highly expressed in oocytes compared with the other follicular cell types (cluster 1). Because mRNA and correspondent protein abundances did not correlate in the oocyte (2, 69–71), overexpression in the oocyte suggests that these transcripts may be stored as maternal RNAs for translation during oocyte maturation and/or the first rounds of embryonic cleavage (72). An increased abundance of these transcripts in oocyte and presence of their corresponding proteins in ffEVs are of particular importance to oocyte maturation and further embryonic development. In fact, recent analysis of oocyte proteome (42) revealed that 67.4% of ffEV proteins were present in bovine oocytes. To the best of our knowledge, the secretion/uptake of EVs by the oocyte has not previously been reported; however, the occurrence of rapid molecular exchange between the oocyte and surrounding CC was observed in multiple studies [reviewed in Collado et al. (5)]. Taken together, our results showed that oocytes contain an important number of transcripts encoding ffEV proteins and this corroborates with detection of a number of corresponding proteins in the oocytes (41, 42). Taken together, this indicates that the release of EVs by the oocyte should not be excluded.

On the other hand, coding genes overexpressed in CC compared with other follicular cells were also found in the list of proteins from ffEVs. These proteins are known to be involved in oocyte maturation and include proteins related to steroidogenesis (HSDA7B1 and PTFRGN), oocyte maturation, and sperm capacitation (calreticulin, integrin 2, clustrin). More than half of ffEV proteins were also identified in CC surrounding mature bovine oocytes (41). Taken together, these findings indicate that CC may be also important sources of ffEVs and play important roles in the acquisition of oocyte competence.

Furthermore, a number of proteins identified in ffEVs, which were related to complement and coagulation cascades, were previously determined to be overexpressed at the transcriptional level in CC (C3, CLU, F2), oocytes (C5, PLG, FGB), and TH cells (A2M, C1QB, CFH, CFI). Others were similarly expressed in the different cell types (C8G, FGA, SERPIND1, and VTN). A large number of these proteins were also identified in a recent study by Walter et al. (45), which showed that proteins related to complement, coagulation cascades, and steroid biosynthesis were enriched in CC surrounding the oocytes that matured successfully under *in vivo* conditions compared with IVM. For example, an increase of C3 secretion by COCs during IVM was reported, although C3 concentrations in FF were 100-fold higher than in spent IVM medium *in vivo* (45). In contrast, the relatively low levels of expression of C3, C5, and C9 genes in follicular cells of our study suggest that correspondent complement and coagulation cascade proteins in ffEVs may originate from follicular cells, but may also be derived from plasma.

The integrative proteomic and transcriptome data provided in this study suggest that a mixture of ffEVs derived from GC, CC, TH, and the oocyte may be present in FF. Moreover, we cannot exclude the possibility that some ffEVs may be derived from the plasma. However, the differentiation of EV subpopulations in a complex mixture such as FF remains a major challenge for researchers in the field (73). Advances in immunoaffinity purification methods for EV isolation will allow us to detect how representative a subset of EVs may be of the entire ffEV population. Therefore, knowledge of the molecular components of ffEVs and an understanding of their potential origins at different levels will be needed to serve as a strong molecular basis for the development of improved detection methods. Our current data provide a basis for this type of study.

Our current findings and those of other researchers (6) suggest that proteins and other components identified in ffEVs have the potential to serve as non-invasive molecular markers of oocyte competence. This characteristic of ffEVs makes them a valuable source of novel diagnostic and therapeutic strategies in reproductive medicine (74). Although their utility is theoretical currently, data have shown that levels of cumulus proteins were different in mature and non-mature COCs (45). These differential proteins were detected in ffEVs, and therefore, ffEVs may be involved in the acquisition of competence by the enclosed oocyte. Further, ffEVs used as supplements during IVM produced a pronounced effect on oocyte competence, which was reflected in increased blastocyst formation rates (16). This reflects the potential of ffEVs for improving outcomes of a variety of assisted reproductive technologies.

In conclusion, our study provides the first extensive characterization of the protein cargo of ffEVs isolated from small bovine follicles (3–6 mm), which are known to be capable of improving oocyte competence *in vitro*. The proteins identified here may be associated to oocyte maturation and embryo development competence. In addition, they may be associated with a variety of signaling processes that occur between the oocyte and follicular cells, and likely determine whether further follicular growth or death occurs. Furthermore, our integrative proteomic and transcriptome analysis strongly suggested that the FF may be composed of a mixture of EVs that are derived from ovarian follicular somatic cells, the oocyte, and circulating blood. Our findings provide a strong basis for the further characterization of EV subpopulations in FF. Taken together, the findings of this study highlight the potential of ffEVs as sources of non-invasive molecular markers of oocyte competence and enhance our capacity to use ffEVs to improve outcomes in a variety of assisted reproductive technologies.

## Data Availability Statement

The mass spectrometry proteomics data have been deposited to the ProteomeXchange Consortium via the PRIDE partner repository with the dataset identifier PXD020508.

## Ethics Statement

Ethical review and approval was not required for the animal study because No experiments with living animals were performed.

## Author Contributions

SU designed the study, performed the experiments, analyzed the data, and wrote the paper. CA performed the experiments and wrote the paper. VL, A-PT-G, LC-S, and GT prepared protein samples, performed mass spectrometry analyses, protein identification, and analyzed proteomic data. RU performed the electron microscopy analysis, and AC performed the transcriptomic analysis. GS conceived the study, and participated in data analysis and the wrote the paper. All authors contributed to the article and approved the submitted version.

## Conflict of Interest

The authors declare that the research was conducted in the absence of any commercial or financial relationships that could be construed as a potential conflict of interest.

## Supplementary Material

Supplementary Figure 1 Proteomic analyses workflow.

Supplementary Figure 2 Gene Ontology analysis of proteins identified in ffEV, GC, and both ffEV and GC.

Supplementary Table 1 Data of MS analysis of bovine ffEVs and GC.

Supplementary Table 2 List of proteins identified in bovine ffEVs and GC.

Supplementary Table 3 Gene Ontology terms in ffEV-unique, common ffEV-GC, and GC-unique protein lists.

Supplementary Table 4 Enrichment Analysis of GO terms of ffEV proteins using DAVID.

Supplementary Table 5 Gene expression analysis of ffEV proteins in follicular cells.
