# Supplementary figures and images for: Protein Cargo of Extracellular Vesicles From Bovine Follicular Fluid and Analysis of Their Origin From Different Ovarian Cells
Source: Front Vet Sci. 2020 Nov 4;7:584948. doi: 10.3389/fvets.2020.584948 (PMC7672127; doi:10.3389/fvets.2020.584948)

# Proteomic analysis workflow

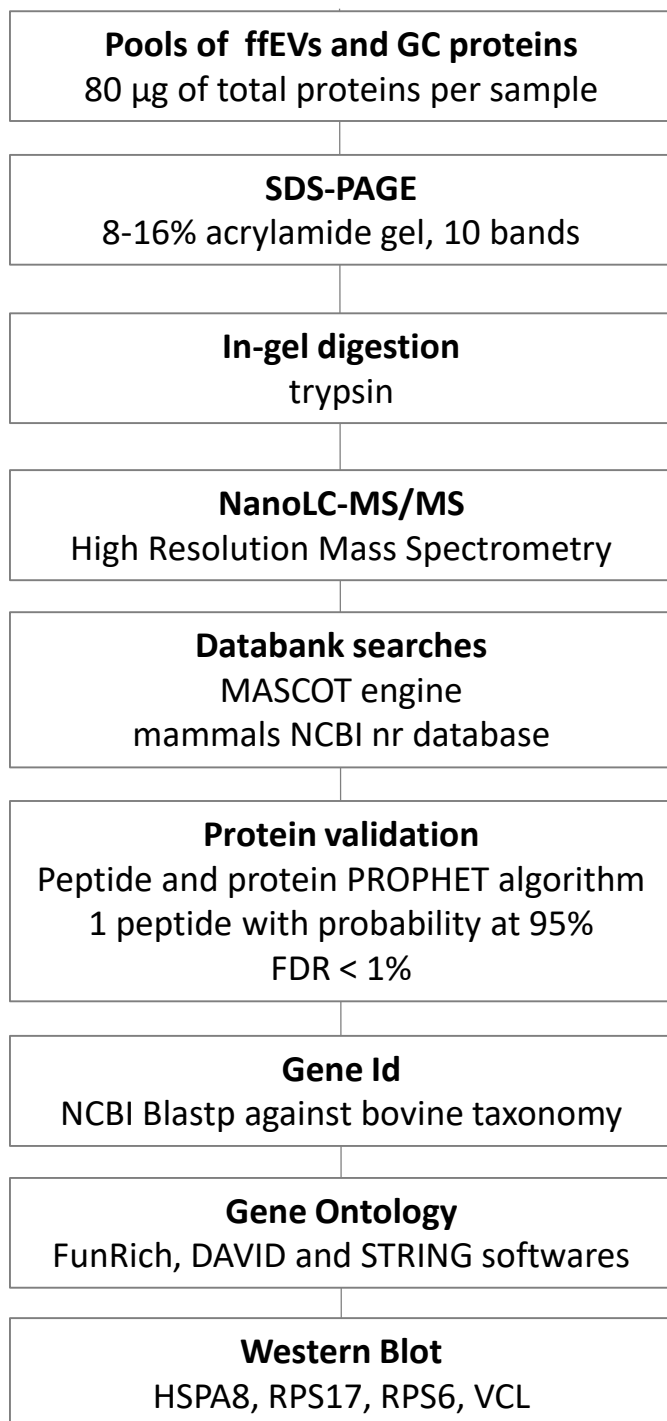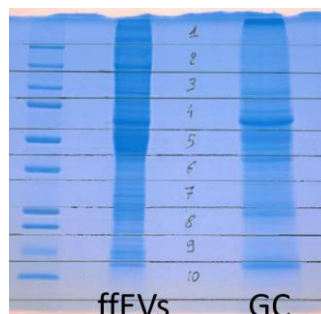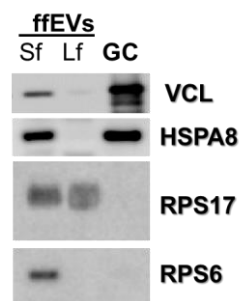

Supplement: Supplementary Figure 1 — Proteomic analyses workflow. [file Data_Sheet_1.PDF]

(A)

## Biological Pathways

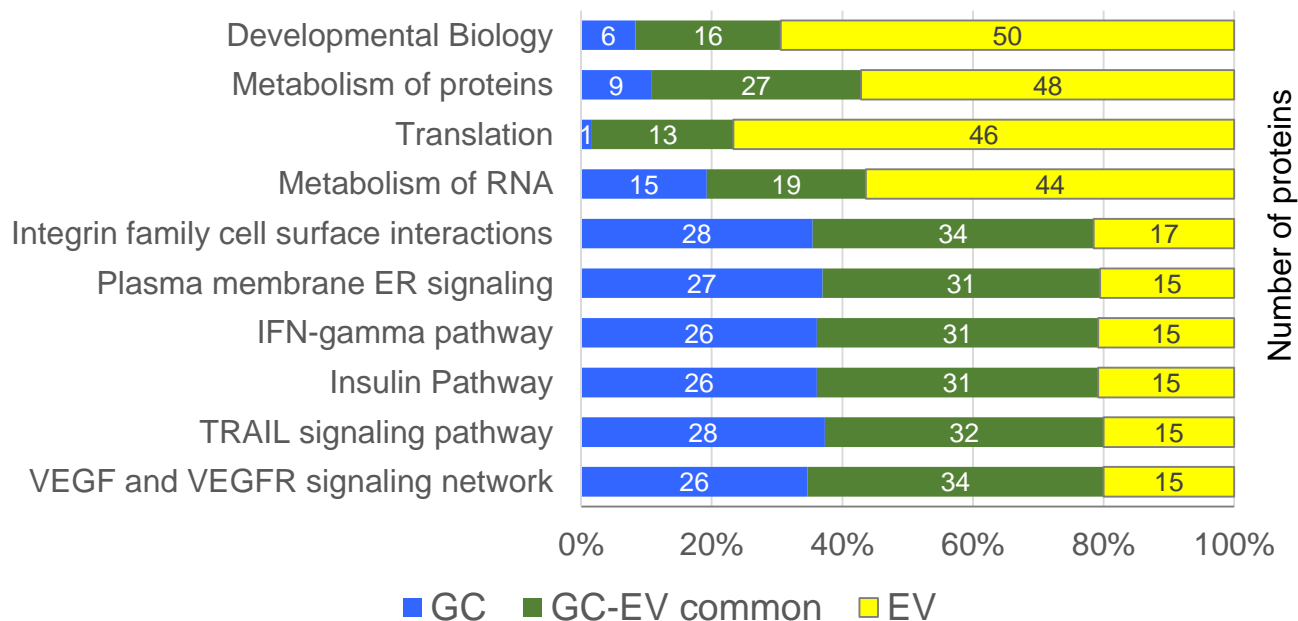

(B)

## Molecular function

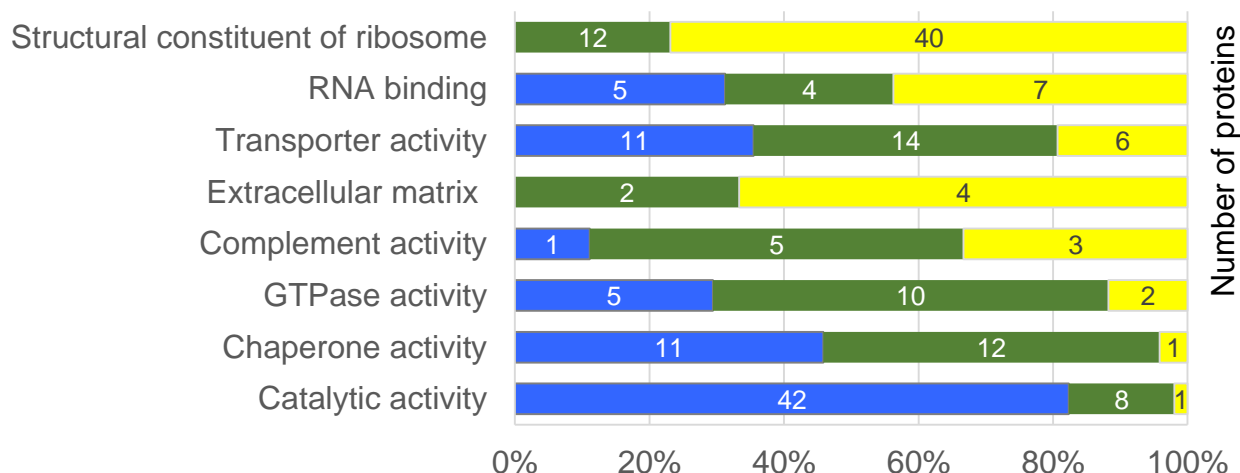

(C)

## Biological processes

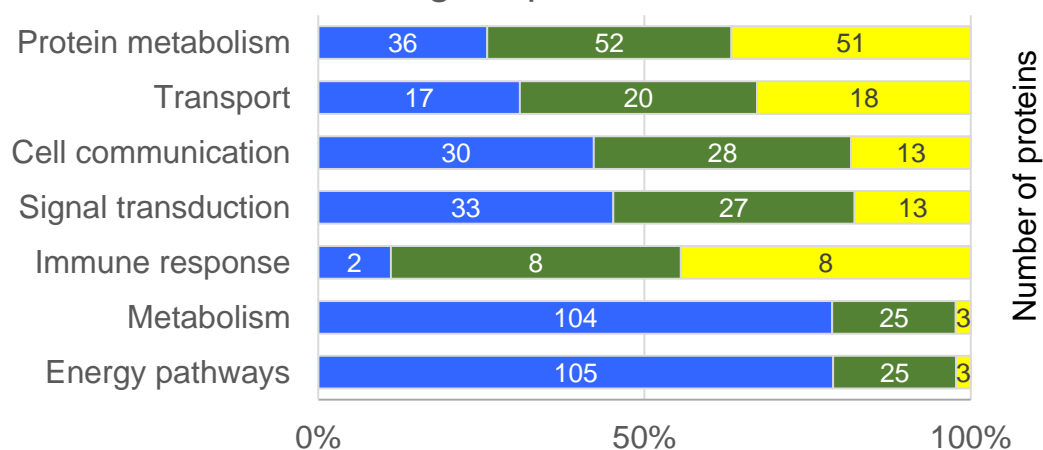

Supplement: Supplementary Figure 2 — Gene Ontology analysis of proteins identified in ffEV, GC, and both ffEV and GC. [file Data_Sheet_2.PDF]
