# Supplementary material for: Protein Cargo of Extracellular Vesicles From Bovine Follicular Fluid and Analysis of Their Origin From Different Ovarian Cells
Source: Front Vet Sci. 2020 Nov 4;7:584948. doi: 10.3389/fvets.2020.584948 (PMC7672127; doi:10.3389/fvets.2020.584948)
Supplement: Supplementary Table 1 — Data of MS analysis of bovine ffEVs and GC. [file Table_1.pdf]

Supplementary data Table S1.

List of proteins identified by nanoLC-MS/MS:

767 proteins identified in 601 clusters
validated with Scaffold software using Peptide an Protein Prophet

Proteins Informations				Normalized Weighted Spectra		Normalized emPAI		Total Unique Peptide Count		Total Unique Spectrum Count		Percent Coverage		Protein Identification Probability	
#	Identified Proteins	Accession Number	Molecular Weight	EXO	Granulosa	EXO	Granulosa	EXO	Granulosa	EXO	Granulosa	EXO	Granulosa	EXO	Granulosa
1	Cluster of cationic trypsin precursor [Bos taurus] (NP_001107199.1)	NP_001107199.1	26 kDa	3 455,10	3 196,90	10,455	8,9249	12	11	20	19	71,50%	65,00%	100%	100%
1,1	cationic trypsin precursor [Bos taurus]	NP_001107199.1	26 kDa	3 092,10	2 835,90	10,455	8,9249	12	11	20	19	71,50%	65,00%	100%	100%
2	Cluster of bP47 protein, partial [Bos taurus] (CAA72406.1)	CAA72406.1 [5]	46 kDa	3 023,70	136,09	40,042	1,457	32	11	56	13	59,70%	33,70%	100%	100%
2,1	bP47 protein, partial [Bos taurus]	CAA72406.1 (+3)	46 kDa	2 491,80	115,85	49,168	1,457	32	11	56	13	59,70%	33,70%	100%	100%
2,2	MFGE8 protein [Bos taurus]	AAI02355.1	48 kDa	328,82	16,523	30,916	1,203	30	10	52	12	54,80%	26,90%	99%	80%
3	Cluster of ALB protein [Bos taurus] (AAI02743.1)	AAI02743.1 [4]	69 kDa	940,2	2 900,90	2,1904	5,6354	30	45	43	59	33,60%	56,30%	100%	100%
3,1	ALB protein [Bos taurus]	AAI02743.1	69 kDa	293,1	881,86	4,325	9,4254	24	38	36	51	33,60%	56,30%	100%	100%
3,2	PREDICTED: serum albumin [Bubalus bubalis]	XP_006047011.1	69 kDa	416,15	1 406,50	3,0245	5,8631	22	32	30	42	30,50%	46,80%	100%	100%
3,3	PREDICTED: serum albumin [Pantholops hodgsonii]	XP_005983856.1	69 kDa	62,856	204,25	1,2072	1,6177	11	15	17	21	15,70%	21,40%	98%	100%
3,4	serum albumin [Carlito syrichta]	XP_021565587.1	69 kDa	94,859	176,53	0,20467	0,14631	3	2	4	3	5,06%	2,45%	100%	91%
4	Cluster of histone H1.3 [Bos taurus] (NP_001094536.1)	NP_001094536.1 [43]	22 kDa	1 697,30	0	12,981	0	67	0	98	0	67,50%	0,00%	100%	83%
4,1	histone H2B type 2-E-like [Myotis lucifugus]	XP_006105894.1	14 kDa	317,72	0	25,979	0	8	0	15	0	67,50%	0,00%	100%	6%
4,2	histone H2A type 1-like [Mus pahari]	XP_021071623.1	14 kDa	219,11	0	6,038	0	6	0	9	0	40,80%	0,00%	100%	14%
4,3	PREDICTED: histone H1.5-like [Bubalus bubalis]	XP_006045703.1	34 kDa	154,93	0	8,4377	0	17	0	24	0	40,80%	0	100%	0
4,4	hypothetical protein M91_16887, partial [Bos mutus]	ELR45782.1 (+1)	14 kDa	62,464	0	59,906	0	12	0	19	0	67,20%	0	100%	0
4,5	PREDICTED: histone H1.2 [Camelus bactrianus]	XP_010972491.1	21 kDa	54,536	0	3,3301	0	8	0	10	0	33,30%	0	100%	0
4,6	PREDICTED: histone H1t-like [Panthera pardus]	XP_019280705.1	22 kDa	25,108	0	0,76487	0	3	0	4	0	12,80%	0	100%	0
4,7	PREDICTED: histone H2A type 1-like [Bos indicus]	XP_019841862.1	14 kDa	19,444	0	9,8398	0	7	0	11	0	40,80%	0	100%	0
4,8	histone H1.2 [Bos taurus]	NP_001076894.1 (+19)	21 kDa	63,8	0	3,3301	0	8	0	10	0	32,90%	0	100%	0
4,9	PREDICTED: histone H4-like [Bos indicus]	XP_019822814.1	11 kDa	298,19	0	68,787	0	10	0	16	0	57,30%	0	100%	0
4,1	histone H1.3 [Bos taurus]	NP_001094536.1 (+7)	22 kDa	47,611	0	3,1216	0	8	0	10	0	31,70%	0	100%	0
4,11	PREDICTED: histone H2B type 1-like [Bos indicus]	XP_019817686.1	14 kDa	2,6051	0	0,93024	0	2	0	3	0	23,80%	0	100%	0
4,12	PREDICTED: histone H1.4 isoform X1 [Oryctolagus cuniculus]	XP_008260689.2	38 kDa	89,997	0	8,82	0	19	0	27	0	43,20%	0	100%	0
4,13	histone H3.3-like [Piliocolobus tephrosceles]	XP_023052122.1	15 kDa	13,201	0	1,7504	0	4	0	5	0	24,30%	0	100%	0
4,14	PREDICTED: late histone H2B.L4-like [Bos indicus]	XP_019822502.1	14 kDa	9,1964	0	2,752	0	5	0	6	0	24,20%	0	97%	0
4,15	histone H1.1 [Bos taurus]	XP_010816821.1	22 kDa	14,134	0	1,0334	0	4	0	5	0	20,60%	0	100%	0
4,16	hypothetical protein Celaphus_00014537 [Cervus elaphus hippelaphus]	OWK13060.1	28 kDa	80,444	0	2,8709	0	9	0	12	0	37,20%	0	100%	0
5	Cluster of clathrin heavy chain 1 [Bos taurus] (NP_776448.1)	NP_776448.1 [79]	192 kDa	1 502,90	30,378	0,64162	0,061761	57	4	73	4	35,50%	2,88%	100%	100%
5,1	clathrin heavy chain 1 [Bos taurus]	NP_776448.1 (+66)	192 kDa	1 471,70	24,562	2,273	0,051124	53	3	69	3	35,50%	2,03%	100%	100%
5,2	clathrin heavy chain 2 [Pteropus alecto]	XP_015442279.1	190 kDa	7,9051	0	0,20755	0	10	0	11	0	6,10%	0	100%	0
5,3	PREDICTED: LOW QUALITY PROTEIN: clathrin heavy chain 2 [Callithrix jacchus]	XP_009004827.2	195 kDa	4,7755	0	0,20201	0	9	0	11	0	4,36%	0	98%	0
5,4	clathrin heavy chain 2 isoform X1 [Phascolarctos cinereus]	XP_020852006.1 (+2)	190 kDa	3,5223	0	0,27084	0	9	0	11	0	6,64%	0	96%	0
5,5	PREDICTED: clathrin heavy chain 2 isoform X1 [Saimiri boliviensis boliviensis]	XP_010328781.1 (+5)	187 kDa	4,6828	0	0,25474	0	9	0	12	0	5,60%	0	98%	0
5,6	PREDICTED: tricarboxylate transport protein, mitochondrial isoform X2 [Cricetulus griseus]	XP_007616356.1	45 kDa	0	2,5458	0	0,072397	0	1	0	1	0	2,88%	0	95%
6	Cluster of complement component 3 [Bos taurus] (AKE14287.1)	AKE14287.1 [5]	187 kDa	524,07	210,26	0,39548	0,72592	36	27	43	33	29,10%	23,20%	100%	100%
6,1	complement component 3 [Bos taurus]	AKE14287.1 (+2)	187 kDa	472,63	199,35	1,0031	0,72592	33	26	40	32	29,10%	23,20%	100%	100%
6,2	complement C3 [Equus caballus]	XP_001915589.1	186 kDa	24,013	3,7063	0,091671	0,034859	4	2	5	2	3,55%	1,87%	98%	69%
6,3	complement C3 [Mesocricetus auratus]	XP_021089376.1	186 kDa	9,3538	1,6713	0,091671	0,034859	3	1	5	2	2,53%	0,84%	95%	53%
7	Cluster of PREDICTED: LOW QUALITY PROTEIN: actin-like [Rhinopithecus bieti] (XP_017741679.1)	XP_017741679.1 [8]	34 kDa	121,49	548,17	0,16542	7,4914	9	35	14	54	18,20%	64,20%	100%	100%
7,1	PREDICTED: LOW QUALITY PROTEIN: actin-like [Rhinopithecus bieti]	XP_017741679.1	34 kDa	11,202	26,317	0,20932	0,74831	1	4	2	6	5,96%	19,50%	56%	100%
7,2	PREDICTED: beta-actin-like protein 2 isoform X1 [Nannospalax galili]	XP_008830814.1 (+1)	42 kDa	12,325	64,771	0,16542	0,96427	2	6	2	8	7,18%	26,30%	99%	100%
7,3	hypothetical protein A6R68_03900 [Neotoma lepida]	OBS67565.1	27 kDa	0	21,887	0	2,2097	0	8	0	10	0,00%	30,70%	27%	100%
7,4	actin beta, partial [Chaerephon plicatus]	AFJ05200.1	23 kDa	5,7382	60,585	0,14908	16,701	1	14	1	20	7,84%	64,20%	34%	100%
7,5	LOW QUALITY PROTEIN: actin, clone 302-like [Dasypus novemcinctus]	XP_012373603.1	42 kDa	24,712	101,62	0,84224	14,074	5	21	8	34	18,20%	59,10%	86%	100%
7,6	RecName: Full=Actin, cytoplasmic 1; AltName: Full=Beta-actin; Contains: RecName: Full=Actin, cytoplasmic	Q0PGG4.1	42 kDa	15,197	58,947	0,58364	4,6694	4	16	6	23	13,30%	43,70%	71%	99%
7,7	actin, cytoplasmic 1 [Mus pahari]	XP_021042765.1	42 kDa	10,307	43,435	0,84516	13,073	5	22	8	33	18,10%	63,50%	53%	96%
8	Cluster of IgG1 heavy chain constant region, partial [Bos taurus] (AAB37381.2)	AAB37381.2 [3]	36 kDa	140	132,33	1,3656	1,9256	11	16	16	20	38,00%	52,90%	100%	100%
8,1	IgG1 heavy chain constant region, partial [Bos taurus]	AAB37381.2	36 kDa	82,721	53,572	2,1663	2,1142	9	11	13	13	38,00%	52,90%	100%	100%
8,2	immunoglobulin gamma heavy chain [Bos taurus]	AQT27060.1	50 kDa	41,715	62,199	0,56485	1,2629	5	11	7	13	18,60%	36,40%	100%	100%
8,3	immunoglobulin gamma 1 heavy chain constant region, partial [Bos taurus]	ABE68619.1	36 kDa	15,564	16,563	2,1663	2,3996	9	12	13	14	32,50%	52,90%	92%	95%
9	Cluster of endoplasmin precursor [Bos taurus] (NP_777125.1)	NP_777125.1 [3]	92 kDa	116,44	619,03	0,50347	1,2665	12	24	13	26	17,70%	32,80%	100%	100%
9,1	endoplasmin precursor [Bos taurus]	NP_777125.1 (+1)	92 kDa	61,324	361,23	0,52271	1,2837	11	22	12	24	17,70%	32,80%	100%	100%
9,2	heat shock protein gp96 precursor, partial [Homo sapiens]	AAK74072.1	90 kDa	51,776	240,88	0,48423	1,2494	10	21	11	23	15,60%	28,10%	100%	100%
10	heat shock protein beta-1 [Bos taurus]	NP_001020740.1 (+1)	23 kDa	428,19	149,46	5,0265	4,1491	9	11	13	12	75,00%	68,10%	100%	100%
11	Cluster of serpin H1 precursor [Bos taurus] (NP_001039528.1)	NP_001039528.1 [6]	47 kDa	293,11	392,49	1,7833	5,0295	15	27	19	35	38,30%	56,70%	100%	100%
11,1	serpin H1 precursor [Bos taurus]	NP_001039528.1 (+2)	47 kDa	251,34	326,96	2,9504	7,2041	14	23	18	31	38,30%	56,70%	100%	100%
11,2	PREDICTED: serpin H1 [Chrysochloris asiatica]	XP_006875485.1	47 kDa	13,012	27,768	0,61618	0,8326	6	8	7	9	17,90%	18,40%	100%	91%
11,3	serpin H1 isoform X1 [Pteropus vampyrus]	XP_011366708.1 (+1)	47 kDa	20,52	25,538	1,1245	2,8549	9	15	11	20	21,50%	31,50%	85%	99%
12	Cluster of vitronectin precursor [Bos taurus] (NP_001030222.1)	NP_001030222.1 [4]	54 kDa	487,66	14,581	1,1504	0,12477	14	2	20	2	31,50%	5,67%	100%	100%
12,1	vitronectin precursor [Bos taurus]	NP_001030222.1 (+2)	54 kDa	458,38	13,51	1,94	0,12477	13	2	18	2	31,50%	5,67%	100%	100%
12,2	PREDICTED: vitronectin [Callithrix jacchus]	XP_002748402.1	52 kDa	19,986	0	0,36072	0	3	0	5	0	5,24%	0	100%	0
13	immunoglobulin kappa light chain constant region, partial [Bos taurus]	AEM45004.1 (+3)	12 kDa	411,2	54,681	88,131	8,2831	12	8	18	9	86,10%	84,30%	100%	100%
14	Cluster of PREDICTED: hemoglobin subunit beta [Bos indicus] (XP_019831403.1)	XP_019831403.1 [5]	16 kDa	51,825	584,48	1,6257	5,7055	6	12	6	16	46,20%	62,80%	100%	100%
14,1	PREDICTED: hemoglobin subunit beta [Bos indicus]	XP_019831403.1	16 kDa	27,382	317,09	1,6257	10,931	5	9	5	13	45,50%	62,80%	100%	100%
14,2	RecName: Full=Hemoglobin subunit beta-A; AltName: Full=Beta-A-globin; AltName: Full=Hemoglobin beta-/	P04346.1	16 kDa	17,716	175,25	1,6257	5,721	5	7	5	8	46,20%	53,80%	100%	100%
14,3	RecName: Full=Hemoglobin subunit beta; AltName: Full=Beta-globin; AltName: Full=Hemoglobin beta chain	P02053.2 (+2)	16 kDa	2,1127	57,377	0,21529	0,4649	1	2	1	2	6,80%	15,60%	5%	100%
15	Cluster of immunoglobulin lambda-1 light chain-like [Bos taurus] (XP_024844915.1)	XP_024844915.1 [2]	25 kDa	347,4	188,23	1,9639	4,876	10	11	10	16	44,10%	48,70%	100%	100%
15,1	immunoglobulin lambda-1 light chain-like [Bos taurus]	XP_024844915.1	25 kDa	151,64	80,425	1,7757	4,1373	7	8	7	11	44,10%	48,70%	100%	100%
15,2	immunoglobulin lambda light chain [Bos taurus]	AAK84156.1	25 kDa	98,586	56,952	2,1521	5,6147	8	8	8	13	42,60%	34,00%	100%	100%
16	Cluster of prostaglandin F2 receptor negative regulator [Bos taurus] (XP_010801437.1)	XP_010801437.1 [3]	99 kDa	197,95	1,2151	1,0582	0,032662	16	1	22	1	21,80%	1,14%	100%	99%

16,1	prostaglandin F2 receptor negative regulator [Bos taurus]	XP_010801437.1 (+2)	99 kDa	194,44	1,2151	1,0582	0,032662	16	1	22	1	21,80%	1,14%	100%	99%
17	Cluster of PREDICTED: glutathione S-transferase A1 [Bison bison bison] (XP_010843548.1)	XP_010843548.1 [3]	26 kDa	66,287	255,26	0,56755	5,4364	5	14	7	22	19,80%	66,20%	100%	100%
17,1	PREDICTED: glutathione S-transferase A1 [Bison bison bison]	XP_010843548.1	26 kDa	36,693	207,59	0,85437	10,434	4	13	5	20	19,80%	66,20%	100%	100%
17,2	glutathione S-transferase A1-like isoform X1 [Microcebus murinus]	XP_012616307.1 (+1)	26 kDa	26,337	39,669	0,28074	0,43828	1	2	2	3	5,38%	11,20%	99%	100%
18	endoplasmic reticulum chaperone BiP precursor [Bos taurus]	NP_001068616.1 (+1)	72 kDa	71,366	247,89	0,5619	1,1048	8	13	10	17	17,40%	26,00%	100%	100%
19	Cluster of serotransferrin precursor [Bos taurus] (NP_803450.2)	NP_803450.2	78 kDa	94,304	153,11	0,78891	0,92077	12	16	14	17	17,20%	20,60%	100%	100%
19,1	serotransferrin precursor [Bos taurus]	NP_803450.2	78 kDa	90,698	143,83	0,78891	0,92077	12	15	14	16	17,20%	20,60%	100%	100%
20	Cluster of Fibrinogen alpha chain [Bos taurus] (AAI42073.1)	AAI42073.1 [4]	67 kDa	207,3	58,326	1,0578	0,26605	11	5	15	5	24,40%	10,70%	100%	100%
20,1	Fibrinogen alpha chain [Bos taurus]	AAI42073.1 (+3)	67 kDa	207,26	58,319	1,0578	0,26605	11	5	15	5	24,40%	10,70%	100%	100%
21	Cluster of hemoglobin subunit alpha [Bos taurus] (NP_001070890.2)	NP_001070890.2 [44]	15 kDa	29,736	430,15	0,27156	2,7826	6	14	7	19	10,60%	71,80%	100%	100%
21,1	hemoglobin subunit alpha [Bos taurus]	NP_001070890.2 (+1)	15 kDa	11,894	189,89	0,50028	12,451	1	8	2	13	10,60%	71,80%	100%	100%
21,2	hemoglobin alpha subunit 2 [Peromyscus maniculatus]	ABN71051.1 (+21)	15 kDa	5,0975	65,799	0,22859	0,83176	1	3	1	3	10,60%	36,60%	99%	100%
21,3	hemoglobin alpha-1 globin chain [Homo sapiens]	AAK37554.1 (+12)	15 kDa	4,2479	59,793	0,22341	1,2057	1	3	1	4	10,60%	28,20%	99%	100%
21,4	RecName: Full=Hemoglobin subunit alpha	B3EWE3.1 (+1)	15 kDa	2,5488	43,829	0,22683	0,49236	1	2	1	2	10,60%	17,00%	98%	99%
21,5	RecName: Full=Hemoglobin subunit alpha; AltName: Full=Alpha-globin; AltName: Full=Hemoglobin alpha ch	P01973.1 (+3)	15 kDa	2,5488	45,189	0,22683	1,2301	1	3	1	4	10,60%	28,40%	99%	100%
21,6	hemoglobin subunit alpha-like [Physeter catodon]	XP_007130440.2	15 kDa	3,3984	25,609	0,22341	0,48419	1	2	1	2	10,60%	16,90%	98%	100%
22	Cluster of PREDICTED: elongation factor 1-alpha 1 [Bison bison bison] (XP_010849567.1)	XP_010849567.1 [22]	50 kDa	101,1	213,86	0,39133	0,42353	10	11	12	13	21,20%	31,00%	100%	100%
22,1	PREDICTED: LOW QUALITY PROTEIN: elongation factor 1-alpha 1-like [Galeopterus variegatus]	XP_008572262.1	49 kDa	3,0949	4,2661	0,21545	0,13563	3	2	3	2	6,86%	4,87%	99%	96%
22,2	Elongation factor 1-alpha 1 [Heterocephalus glaber]	EHB15382.1	24 kDa	1,1749	3,6454	0,30688	0,13985	2	1	2	1	11,10%	6,45%	99%	97%
22,3	PREDICTED: elongation factor 1-alpha 1 [Bison bison bison]	XP_010849567.1 (+18)	50 kDa	62,593	122,82	0,77786	0,99511	7	9	9	11	21,20%	31,00%	100%	100%
22,4	PREDICTED: elongation factor 1-alpha 1-like [Leptonychotes weddellii]	XP_006745012.1	55 kDa	15,741	52,39	0,26515	0,33343	4	4	4	5	11,30%	11,90%	99%	84%
23	Cluster of apolipoprotein A-I preproprotein [Bos taurus] (NP_776667.2)	NP_776667.2 [2]	30 kDa	216,03	88,499	4,3047	4,2012	12	14	16	16	49,10%	54,00%	100%	100%
23,1	apolipoprotein A-I preproprotein [Bos taurus]	NP_776667.2 (+1)	30 kDa	215,56	88,356	4,3047	4,2012	12	14	16	16	49,10%	54,00%	100%	100%
24	Cluster of alpha-2-macroglobulin precursor [Bos taurus] (NP_001103265.1)	NP_001103265.1 [6]	168 kDa	258,27	57,111	0,26519	0,099611	20	6	20	6	15,60%	4,44%	100%	100%
24,1	alpha-2-macroglobulin precursor [Bos taurus]	NP_001103265.1 (+1)	168 kDa	180,1	41,675	0,391	0,099611	17	5	17	5	15,60%	4,44%	100%	100%
24,2	PREDICTED: alpha-2-macroglobulin isoform X1 [Bos indicus]	XP_019817158.1 (+1)	168 kDa	71,036	13,568	0,3643	0,078916	16	4	16	4	14,80%	3,64%	100%	80%
24,3	alpha-2-macroglobulin isoform X1 [Sarcophilus harrisii]	XP_012406159.1 (+1)	165 kDa	4,5548	1,7664	0,040276	0,019462	2	1	2	1	1,56%	0,81%	98%	8%
25	Cluster of PREDICTED: LOW QUALITY PROTEIN: heat shock cognate 71 kDa protein [Bos mutus] (XP_01433378	XP_014333782.1 [13]	72 kDa	40,78	130,02	0,3213	0,54337	9	13	14	16	14,90%	25,30%	100%	100%
25,1	Heat shock cognate 71 kDa protein [Heterocephalus glaber]	EHB18685.1	58 kDa	4,6843	24,029	0,25058	0,31462	2	3	4	5	6,33%	8,64%	100%	100%
25,2	PREDICTED: LOW QUALITY PROTEIN: heat shock cognate 71 kDa protein [Bos mutus]	XP_014333782.1 (+9)	72 kDa	27,014	92,486	0,56614	0,77213	7	11	10	13	14,90%	25,30%	100%	100%
25,3	PREDICTED: heat shock 70 kDa protein 6-like [Monodelphis domestica]	XP_001362950.1 (+1)	71 kDa	1,8391	1,4006	0,14718	0,045731	2	1	3	1	4,04%	2,02%	99%	87%
26	Cluster of fibrinogen gamma-B chain isoform X1 [Bos taurus] (XP_005217490.1)	XP_005217490.1	49 kDa	92,605	97,21	1,0481	1,1571	7	9	11	12	22,80%	33,60%	100%	100%
26,1	fibrinogen gamma-B chain isoform X1 [Bos taurus]	XP_005217490.1	49 kDa	89,919	95,643	1,0481	1,1571	7	9	11	12	22,80%	33,60%	100%	100%
27	Cluster of PREDICTED: neutral alpha-glucosidase AB isoform X3 [Bos indicus] (XP_019810339.1)	XP_019810339.1 [5]	107 kDa	20,39	134,88	0,088258	0,4258	5	23	6	24	4,03%	28,60%	100%	100%
27,1	PREDICTED: neutral alpha-glucosidase AB isoform X3 [Bos indicus]	XP_019810339.1	107 kDa	10,511	115,66	0,12913	0,70778	3	17	4	18	4,03%	28,60%	100%	100%
27,2	hypothetical protein PANDA_014926, partial [Ailuropoda melanoleuca]	EFB28162.1 (+2)	108 kDa	0,4371	6,3441	0,030683	0,34377	1	10	1	10	1,26%	15,50%	13%	98%
27,3	Neutral alpha-glucosidase AB, partial [Tupaia chinensis]	ELW52032.1	141 kDa	9,1941	11,299	0,047391	0,22586	2	9	2	9	1,68%	10,60%	100%	100%
28	Cluster of PREDICTED: tubulin beta-2A chain-like [Ochotona princeps] (XP_012785615.1)	XP_012785615.1 [9]	44 kDa	59,471	210,22	0,72446	1,785	10	25	10	29	28,60%	46,50%	100%	100%
28,1	tubulin, beta 6 isoform 3-like protein [Camelus ferus]	EPY79353.1 (+1)	44 kDa	1,3401	17,463	0,33469	1,3396	4	11	4	12	10,80%	30,20%	76%	100%
28,2	PREDICTED: tubulin beta chain-like [Chrysochloris asiatica]	XP_006861626.1	46 kDa	6,1481	22,626	0,41737	0,98261	5	9	5	10	14,90%	22,50%	89%	99%
28,3	LOW QUALITY PROTEIN: tubulin beta chain [Canis lupus familiaris]	XP_005615473.1	23 kDa	4,7333	14,889	0,51932	1,6084	3	6	3	7	16,20%	33,00%	75%	100%
28,4	PREDICTED: tubulin beta-8 chain [Callithrix jacchus]	XP_002758113.1	50 kDa	0,27355	9,6586	0,13768	0,65738	2	7	2	8	4,26%	15,20%	56%	99%
28,5	PREDICTED: tubulin beta chain isoform X1 [Jaculus jaculus]	XP_012807536.1	50 kDa	12,055	28,617	0,77345	2,9686	9	18	9	22	28,60%	45,70%	98%	100%
28,6	tubulin beta-4A chain [Phascolarctos cinereus]	XP_020827102.1	50 kDa	15,696	46,928	0,67546	2,5581	8	17	8	20	25,60%	46,50%	98%	100%
28,7	PREDICTED: tubulin beta-4B chain-like isoform X1 [Rhinopithecus roxellana]	XP_010385160.1	50 kDa	10,429	32,645	0,37911	1,9257	5	14	5	17	17,50%	38,00%	88%	99%
28,8	PREDICTED: LOW QUALITY PROTEIN: tubulin beta-2B chain isoform X1 [Cricetulus griseus]	XP_016821521.1	48 kDa	6,2868	21,678	0,59191	2,2398	7	15	7	18	24,40%	39,90%	82%	95%
29	Cluster of protein disulfide-isomerase A6 precursor [Bos taurus] (NP_001193274.1)	NP_001193274.1 [13]	48 kDa	119,79	341,45	0,75774	2,4444	10	27	12	37	25,00%	47,00%	100%	100%
29,1	protein disulfide-isomerase A6 [Myotis lucifugus]	XP_023599500.1	56 kDa	35,185	79,079	0,58372	1,6117	6	13	8	17	15,20%	27,00%	100%	100%
29,2	protein disulfide-isomerase A6 precursor [Bos taurus]	NP_001193274.1 (+5)	48 kDa	34,08	142,45	0,94766	5,7241	8	20	10	29	25,00%	47,00%	100%	100%
29,3	protein disulfide isomerase-related protein 5, partial [Homo sapiens]	AAB50217.1	46 kDa	15,39	34,812	0,74182	2,6576	7	13	8	19	20,40%	26,40%	100%	100%
29,4	protein disulfide-isomerase A6 [Dasypus novemcinctus]	XP_004459385.1	48 kDa	10,365	20,181	0,70506	2,2571	6	12	8	18	18,00%	28,20%	61%	100%
29,5	protein disulfide-isomerase A6 [Otolemur garnettii]	XP_003787439.1	48 kDa	11,672	24,884	0,59358	2,7045	6	14	7	20	15,50%	28,20%	59%	98%
29,6	PREDICTED: protein disulfide-isomerase A6 [Erinaceus europaeus]	XP_007529904.1	48 kDa	3,001	9,5554	0,22192	1,0555	3	8	3	11	8,41%	18,40%	38%	100%
29,7	protein disulfide-isomerase A6 [Carlito syrichta]	XP_021571942.1	28 kDa	2,2956	7,0149	0,25102	1,6891	2	6	2	9	10,80%	21,60%	13%	97%
29,8	protein disulfide-isomerase A6 [Delphinapterus leucas]	XP_022409837.1	48 kDa	4,988	14,113	0,39627	1,8554	4	12	5	16	14,10%	32,50%	46%	99%
30	PREDICTED: LOW QUALITY PROTEIN: uncharacterized protein LOC109570963 [Bos indicus]	XP_019832649.1	35 kDa	145,78	65,625	0,57027	1,0315	4	3	5	8	10,00%	11,50%	100%	100%
31	Cluster of TPA: annexin A5 [Bos taurus] (DAA28951.1)	DAA28951.1 [7]	36 kDa	61,171	222,37	0,56149	5,1893	5	20	6	29	25,20%	74,10%	100%	100%
31,1	PREDICTED: annexin A5 isoform X1 [Bos indicus]	XP_019817755.1	36 kDa	49,005	112,54	0,69883	9,498	5	18	6	27	25,20%	72,30%	100%	100%
31,2	TPA: annexin A5 [Bos taurus]	DAA28951.1 (+2)	36 kDa	4,9391	79,595	0,42415	7,8148	3	17	3	23	16,50%	74,10%	100%	100%
31,3	Annexin A5 [Tupaia chinensis]	ELW62626.1 (+1)	46 kDa	2,8376	15,991	0,1499	1,6094	2	11	2	14	4,08%	34,10%	69%	100%
31,4	PREDICTED: annexin A5 [Echinops telfairi]	XP_004716543.1	36 kDa	2,6374	8,8916	0,30382	1,8349	2	7	2	10	10,30%	29,30%	54%	99%
32	Cluster of 60S ribosomal protein L4 [Ictidomys tridecemlineatus] (XP_005316777.1)	XP_005316777.1 [4]	47 kDa	140,18	0	1,0596	0	10	0	12	0	31,40%	0	100%	0
32,1	60S ribosomal protein L4 [Ictidomys tridecemlineatus]	XP_005316777.1 (+1)	47 kDa	90,942	0	1,2491	0	10	0	12	0	31,40%	0	100%	0
32,2	60S ribosomal protein L4 [Otolemur garnettii]	XP_012657259.1	38 kDa	26,881	0	1,1429	0	7	0	9	0	24,30%	0	100%	0
32,3	PREDICTED: LOW QUALITY PROTEIN: 60S ribosomal protein L4-like [Chlorocebus sabaeus]	XP_007980180.1	50 kDa	22,33	0	0,78683	0	7	0	9	0	18,90%	0	99%	0
33	fibrinogen beta chain precursor [Bos taurus]	NP_001136389.1	56 kDa	124,04	36,454	1,2195	0,74978	9	10	14	10	21,00%	22,80%	100%	100%
34	Cluster of 60S ribosomal protein L7a [Bos taurus] (NP_001035610.1)	NP_001035610.1 [2]	30 kDa	167,37	0	2,9406	0	13	0	16	0	41,70%	0	100%	0
34,1	60S ribosomal protein L7a [Bos taurus]	NP_001035610.1 (+1)	30 kDa	166,02	0	2,9406	0	11	0	13	0	41,70%	0	100%	0
35	Cluster of PREDICTED: LOW QUALITY PROTEIN: tubulin alpha-1C chain [Capra hircus] (XP_017903331.1)	XP_017903331.1 [8]	50 kDa	7,6463	256,39	0	2,0684	6	26	6	34	19,40%	49,80%	100%	100%
35,1	tubulin alpha-3 chain-like [Otolemur garnettii]	XP_003801738.2	46 kDa	1,1806	16,025	0,23176	1,1159	3	8	3	11	12,30%	20,50%	94%	100%
35,2	PREDICTED: LOW QUALITY PROTEIN: tubulin alpha-1C chain-like [Callithrix jacchus]	XP_009001973.1	50 kDa	1,7266	61,872	0,38008	3,5754	5	17	5	24	19,40%	49,40%	75%	100%
35,3	PREDICTED: tubulin alpha chain-like 3 [Chrysochloris asiatica]	XP_006862850.1	56 kDa	0	2,638	0	0,11812	0	2	0	2	0,00%	6,50%	54%	97%
35,4	PREDICTED: tubulin alpha-1C chain-like [Vicugna pacos]	XP_006213088.1	50 kDa	0,37154	14,229	0,21338	1,0042	3	9	3	11	11,10%	22,50%	42%	99%
35,5	PREDICTED: tubulin alpha-1A chain-like [Cebus capucinus imitator]	XP_017366013.1	46 kDa	0,52554	23,389	0,23116	2,1808	3	14	3	17	11,10%	38,90%	92%	99%
35,6	PREDICTED: LOW QUALITY PROTEIN: tubulin alpha-1B chain-like [Hipposideros armiger]	XP_019481534.1	46 kDa	0,74741	52,491	0,31939	3,4764	4	18	4	22	15,90%	49,80%	87%	100%
35,7	PREDICTED: LOW QUALITY PROTEIN: tubulin alpha-1C chain [Capra hircus]	XP_017903331.1	50 kDa	0,75024	29,917	0,29408	2,5482	4	16	4	19	14,70%	45,40%	65%	100%
35,8	RecName: Full=Tubulin alpha-1A chain; AltName: Full=Alpha-tubulin 1; AltName: Full=Tubulin alpha-1 chain	P02550.1	50 kDa	1,1375	31,561	0,29263	2,5286	4	15	4	20	15,70%	38,40%	55%	99%
36	Cluster of 40S ribosomal protein S8, partial [Bos mutus] (ELR57442.1)	ELR57442.1 [23]	24 kDa	176,71	0	3,3257	0	10	0	14	0	46,90%	0	100%	0
36,1	40S ribosomal protein S8, partial [Bos mutus]	ELR57442.1 (+21)	24 kDa	148,42	0	4,4348	0	9	0	13	0	46,90%	0	100%	0

36,2	40S ribosomal protein S8 [Octodon degus]	XP_004639262.1	24 kDa	28,263	0	2,2166	0	6	0	9	0	34,10%	0	100%	0
37	Cluster of calreticulin [cattle, brain, Peptide, 400 aa] (AAB30209.1)	AAB30209.1 [3]	46 kDa	14,443	59,541	0,31854	0,84389	4	11	4	12	17,70%	34,30%	100%	100%
37,1	calreticulin [cattle, brain, Peptide, 400 aa]	AAB30209.1	46 kDa	12,086	47,821	0,31854	1,1085	4	10	4	11	17,70%	34,30%	100%	100%
37,2	calreticulin [Physeter catodon]	XP_007103674.1 (+1)	48 kDa	1,8547	9,5498	0,22136	0,5793	3	6	3	7	12,90%	23,00%	42%	100%
38	Cluster of glia-derived nexin precursor [Bos taurus] (NP_777094.1)	NP_777094.1 [11]	44 kDa	166,52	6,0756	1,23	0,1537	9	2	11	2	23,90%	6,55%	100%	100%
38,1	glia-derived nexin precursor [Bos taurus]	NP_777094.1 (+10)	44 kDa	166,25	6,0606	1,23	0,1537	9	2	11	2	23,90%	6,55%	100%	100%
39	Cluster of protein disulfide-isomerase A3 precursor [Bos taurus] (NP_776758.2)	NP_776758.2 [4]	57 kDa	39,081	210,22	0,25934	1,9787	6	20	8	26	13,30%	49,50%	100%	100%
39,1	protein disulfide-isomerase A3 precursor [Bos taurus]	NP_776758.2	57 kDa	30,057	182,6	0,40364	3,0112	4	19	6	25	13,30%	49,50%	100%	100%
39,2	PDIA3 isoform 4, partial [Pan troglodytes]	PNI75197.1 (+1)	14 kDa	7,6463	13,888	0,25408	0,94623	1	3	1	3	11,40%	28,50%	100%	100%
39,3	protein disulfide-isomerase A3 [Ictidomys tridecemlineatus]	XP_005316550.1	57 kDa	1,0142	6,5372	0,1203	0,56015	2	8	2	8	7,33%	19,80%	99%	63%
40	Cluster of malate dehydrogenase, mitochondrial isoform X1 [Bos taurus] (XP_005225065.1)	XP_005225065.1 [6]	36 kDa	0,84959	324,44	0	7,5591	1	28	1	37	6,51%	62,70%	98%	100%
40,1	malate dehydrogenase, mitochondrial isoform X1 [Bos taurus]	XP_005225065.1 (+1)	36 kDa	0,43311	182,59	0,093729	19,069	1	25	1	34	6,51%	62,70%	52%	100%
40,2	PREDICTED: malate dehydrogenase, mitochondrial [Sorex araneus]	XP_004620941.1	35 kDa	0	57,246	0	3,2137	0	12	0	16	0	38,20%	0	100%
40,3	malate dehydrogenase, mitochondrial [Dasypus novemcinctus]	XP_004448330.1	36 kDa	0,26095	50,825	0,094028	3,1039	1	10	1	16	6,51%	36,10%	51%	99%
40,4	PREDICTED: malate dehydrogenase, mitochondrial [Oryctolagus cuniculus]	XP_002711952.1 (+1)	35 kDa	0,1524	30,851	0,094028	4,8495	1	15	1	20	6,51%	46,40%	8%	98%
41	Cluster of 60S ribosomal protein L6 [Bos taurus] (NP_001026926.1)	NP_001026926.1 [4]	33 kDa	107,9	0	2,209	0	8	0	12	0	35,90%	0	100%	0
41,1	60S ribosomal protein L6 [Bos taurus]	NP_001026926.1 (+3)	33 kDa	107,75	0	2,209	0	8	0	12	0	35,90%	0	100%	0
42	Cluster of alpha enolase [Bos taurus] (AAD33073.1)	AAD33073.1 [2]	47 kDa	0	247,88	0	4,4963	0	30	0	40	0	64,70%	0	100%
42,1	alpha enolase [Bos taurus]	AAD33073.1	47 kDa	0	134,31	0	5,9347	0	21	0	29	0	64,70%	0	100%
42,2	alpha-enolase [Ictidomys tridecemlineatus]	XP_005334801.1	47 kDa	0	96,869	0	3,058	0	15	0	21	0	44,00%	0	100%
43	Cluster of 60S ribosomal protein L7 [Bos taurus] (NP_001014928.1)	NP_001014928.1 [22]	29 kDa	81,547	0	1,0311	0	6	0	7	0	26,60%	0,00%	100%	72%
43,1	60S ribosomal protein L7 [Bos taurus]	NP_001014928.1 (+20)	29 kDa	64,973	0	1,136	0	6	0	7	0	26,60%	0,00%	100%	38%
43,2	similar to 60S ribosomal protein L7; similar to P18124 (PID:d133021) [Homo sapiens]	AAD08846.1	29 kDa	15,507	0	0,9263	0	4	0	5	0	23,10%	0,00%	97%	8%
44	Cluster of ATP synthase subunit beta, mitochondrial precursor [Bos taurus] (NP_786990.1)	NP_786990.1 [4]	56 kDa	0	331,73	0	3,5638	0	21	0	26	0	57,60%	0	100%
44,1	ATP synthase subunit beta, mitochondrial precursor [Bos taurus]	NP_786990.1 (+3)	56 kDa	0	327,58	0	3,5638	0	20	0	25	0	57,60%	0	100%
45	Cluster of 60S ribosomal protein L8 [Octodon degus] (XP_004639702.1)	XP_004639702.1	28 kDa	49,276	0	1,1988	0	4	0	7	0	18,30%	0	100%	0
45,1	60S ribosomal protein L8 [Octodon degus]	XP_004639702.1	28 kDa	45,73	0	1,1988	0	4	0	7	0	18,30%	0	100%	0
46	Cluster of LOW QUALITY PROTEIN: 40S ribosomal protein S3-like [Octodon degus] (XP_023571358.1)	XP_023571358.1	27 kDa	90,057	37,669	3,1083	1,2527	11	7	12	7	54,30%	38,70%	100%	100%
46,1	LOW QUALITY PROTEIN: 40S ribosomal protein S3-like [Octodon degus]	XP_023571358.1	27 kDa	89,926	37,64	3,1083	1,2527	11	7	12	7	54,30%	38,70%	100%	100%
47	Cluster of integrin beta 1 [Bos taurus] (ABH07895.1)	ABH07895.1 [30]	88 kDa	115,51	23,057	0,29693	0,074567	8	2	9	2	11,40%	2,63%	100%	100%
47,1	integrin beta 1 [Bos taurus]	ABH07895.1 (+28)	88 kDa	75,515	11,23	0,39217	0,074567	8	2	9	2	11,40%	2,63%	100%	95%
47,2	integrin beta-1 isoform X2 [Phascolarctos cinereus]	XP_020846113.1	88 kDa	23,919	9,3474	0,20168	0,074471	5	2	5	2	7,88%	2,63%	99%	85%
48	Cluster of hypothetical protein A6R68_13763, partial [Neotoma lepida] (OBS71660.1)	OBS71660.1 [8]	34 kDa	82,778	222,65	0,47824	0,9372	7	17	10	21	19,90%	51,80%	100%	100%
48,1	hypothetical protein A6R68_13763, partial [Neotoma lepida]	OBS71660.1	34 kDa	2,5488	6,7281	0,097764	0,20004	1	2	1	2	5,36%	8,83%	99%	100%
48,2	PREDICTED: glyceraldehyde-3-phosphate dehydrogenase-like [Ovis aries musimon]	XP_012021852.1	36 kDa	32,892	147,23	0,85872	2,1035	4	9	7	13	19,50%	39,00%	100%	100%
48,3	Glyceraldehyde-3-phosphate dehydrogenase, partial [Fukomys damarensis]	KFO20252.1 (+1)	37 kDa	12,255	19,583	0,41674	1,3513	4	10	4	10	14,40%	51,80%	60%	99%
48,4	glyceraldehyde-3-phosphate dehydrogenase-like [Dasypus novemcinctus]	XP_012373380.2	17 kDa	9,7468	10,765	0,71212	1,4125	3	5	3	5	19,90%	34,20%	54%	100%
48,5	hypothetical protein A6R68_10850 [Neotoma lepida]	OBS58035.1	30 kDa	1,8298	2,4302	0,23508	0,10873	1	1	2	1	3,60%	2,88%	8%	95%
48,6	glyceraldehyde-3-phosphate dehydrogenase-like [Octodon degus]	XP_012368145.1	36 kDa	11,794	15,816	0,70426	1,1936	5	9	6	9	17,70%	32,10%	67%	96%
48,7	PREDICTED: glyceraldehyde-3-phosphate dehydrogenase-like [Odobenus rosmarus divergens]	XP_012416906.1	36 kDa	0,61648	2,7442	0,093433	0,19079	1	2	1	2	4,22%	15,70%	5%	95%
49	Cluster of complement C4-like isoform X2 [Bos taurus] (XP_005223750.2)	XP_005223750.2 [6]	196 kDa	84,109	6,0756	0,12117	0	12	1	14	1	8,21%	3,48%	100%	100%
49,1	complement C4-like isoform X2 [Bos taurus]	XP_005223750.2 (+2)	196 kDa	24,378	3,036	0,16102	0,016337	8	1	9	1	8,21%	1,80%	100%	84%
49,2	RecName: Full=Complement C4; Contains: RecName: Full=Complement C4 alpha chain; Contains: RecName:	P01030.2	102 kDa	46,308	3,0396	0,17271	0,031622	4	1	5	1	8,15%	3,48%	100%	68%
49,3	complement C4-A [Felis catus]	XP_006931645.1	192 kDa	1,1837	0	0,034567	0	2	0	2	0	1,84%	0	99%	0
49,4	PREDICTED: LOW QUALITY PROTEIN: complement C4-A isoform X1 [Ovis aries]	XP_014958087.1	178 kDa	9,2316	0	0,11637	0	6	0	6	0	5,70%	0,00%	100%	39%
50	Cluster of ATP synthase subunit alpha, mitochondrial precursor [Bos taurus] (NP_777109.1)	NP_777109.1 [5]	60 kDa	0	199,28	0	2,5683	0	25	0	30	0	43,90%	0	100%
50,1	ATP synthase, H+ transporting, mitochondrial F1 complex, alpha subunit 1, cardiac muscle [Bos taurus]	AAI16060.1 (+3)	60 kDa	0	97,886	0	2,5683	0	20	0	24	0	43,90%	0	100%
50,2	ATP synthase subunit alpha, mitochondrial precursor [Bos taurus]	NP_777109.1	60 kDa	0	94,201	0	2,5683	0	20	0	24	0	43,90%	0	100%
51	Cluster of immunoglobulin heavy chain constant region, partial [Bos taurus] (AAP55674.1)	AAP55674.1 [4]	50 kDa	83,26	0	0,5965	0	8	0	10	0	20,70%	0,00%	100%	77%
51,1	immunoglobulin heavy chain constant region, partial [Bos taurus]	AAP55674.1	50 kDa	58,351	0	0,78232	0	7	0	9	0	20,70%	0,00%	100%	38%
51,2	immunoglobulin M heavy chain secretory form [Bos taurus]	AAN60017.1 (+2)	66 kDa	24,909	0	0,41068	0	5	0	7	0	10,70%	0,00%	100%	8%
52	Cluster of sodium/potassium-transporting ATPase subunit alpha-1 [Bos taurus] (NP_001070266.1)	NP_001070266.1 [26]	113 kDa	66,268	68,047	0,10778	0,10907	7	6	7	6	6,86%	6,86%	100%	100%
52,1	PREDICTED: potassium-transporting ATPase alpha chain 2-like [Monodelphis domestica]	XP_007495241.1	116 kDa	3,7232	6,6903	0,028518	0,056448	1	2	1	2	1,44%	2,21%	97%	100%
52,2	sodium/potassium-transporting ATPase subunit alpha-4 isoform X1 [Papio anubis]	XP_003892961.1 (+11)	114 kDa	6,251	4,2361	0,058585	0,028186	2	1	2	1	2,24%	1,46%	100%	49%
52,3	sodium/potassium-transporting ATPase subunit alpha-1 [Bos taurus]	NP_001070266.1 (+11)	113 kDa	26,897	27,564	0,1889	0,1514	6	5	6	5	6,86%	6,86%	100%	99%
52,4	ATP1A1 [Bos indicus]	AGY54951.1	113 kDa	21,143	21,289	0,15513	0,11937	5	4	5	4	5,39%	5,39%	97%	99%
53	Cluster of 60S ribosomal protein L13a [Bos taurus] (NP_001070466.1)	NP_001070466.1 [6]	24 kDa	33,134	0	1,907	0	6	0	8	0	32,00%	0	100%	0
53,1	60S ribosomal protein L13a [Bos taurus]	NP_001070466.1 (+5)	24 kDa	33,088	0	1,907	0	6	0	8	0	32,00%	0	100%	0
54	Cluster of ADP/ATP translocase 3 [Bos taurus] (NP_777085.1)	NP_777085.1 [2]	33 kDa	0	122,73	0	1,3928	0	11	0	14	0	37,50%	0	100%
54,1	ADP/ATP translocase 3 [Bos taurus]	NP_777085.1	33 kDa	0	54,61	0	1,3525	0	7	0	8	0	35,20%	0	100%
54,2	LOW QUALITY PROTEIN: ADP/ATP translocase 2 [Dasypus novemcinctus]	XP_023441614.1	28 kDa	0	64,306	0	1,433	0	6	0	8	0	37,50%	0	100%
55	Cluster of dolichyl-diphosphooligosaccharide--protein glycosyltransferase subunit 1 precursor [Bos taurus] (NI	NP_001076074.1 [6]	68 kDa	50,126	40,099	0,33347	0,12479	8	4	9	4	16,30%	8,87%	100%	100%
55,1	dolichyl-diphosphooligosaccharide--protein glycosyltransferase subunit 1 precursor [Bos taurus]	NP_001076074.1	68 kDa	43,207	28,863	0,4594	0,20326	7	4	8	4	16,30%	8,87%	100%	100%
55,2	PREDICTED: dolichyl-diphosphooligosaccharide--protein glycosyltransferase subunit 1 [Orcinus orca]	XP_004284389.1 (+2)	70 kDa	0,78424	4,4866	0,097146	0,046319	2	1	2	1	3,72%	1,94%	91%	98%
55,3	PREDICTED: dolichyl-diphosphooligosaccharide--protein glycosyltransferase subunit 1 isoform X1 [Odobenu	XP_004395756.1 (+1)	69 kDa	4,1629	4,3489	0,20754	0,096557	4	2	4	2	10,70%	4,45%	99%	55%
56	Cluster of PREDICTED: major vault protein [Bison bison bison] (XP_010835612.1)	XP_010835612.1 [2]	99 kDa	53,524	0	0,37944	0	14	0	14	0	23,50%	0	100%	0
56,1	PREDICTED: major vault protein [Bison bison bison]	XP_010835612.1	99 kDa	51,832	0	0,58139	0	14	0	14	0	23,50%	0	100%	0
56,2	major vault protein [Felis catus]	NP_001036027.1	99 kDa	1,1401	0	0,17748	0	5	0	5	0	6,85%	0	96%	0
57	Cluster of immunoglobulin gamma heavy chain [Bos taurus] (AQT27056.1)	AQT27056.1 [2]	49 kDa	121,99	86,326	0,9618	1,6546	8	14	12	19	26,70%	40,20%	100%	100%
57,1	immunoglobulin gamma heavy chain [Bos taurus]	AQT27056.1	49 kDa	69,245	47,994	1,0416	1,6047	7	11	11	15	21,40%	40,20%	100%	100%
57,2	immunoglobulin gamma heavy chain [Bos taurus]	AQT27057.1	51 kDa	52,749	38,332	0,88198	1,7045	7	11	10	16	26,70%	39,40%	100%	100%
58	KRT9 protein, partial [Homo sapiens]	AAI21171.1 (+3)	48 kDa	54,374	55,896	0,30621	0,38674	4	5	4	5	33,80%	39,20%	100%	100%
59	elongation factor 2 [Bos taurus]	NP_001068589.1 (+2)	95 kDa	1,6992	132,45	0,034605	0,3947	1	9	1	10	1,52%	14,70%	97%	100%
60	Cluster of transferrin receptor protein 1 [Bos taurus] (NP_001193506.1)	NP_001193506.1 [7]	85 kDa	123,19	0	0,30392	0	7	0	7	0	11,60%	0	100%	0
60,1	transferrin receptor protein 1 [Bos taurus]	NP_001193506.1 (+6)	85 kDa	118,74	0	0,30392	0	7	0	7	0	11,60%	0	100%	0
61	Cluster of gap junction alpha-1 protein [Bos taurus] (NP_776493.1)	NP_776493.1	43 kDa	73,065	17,012	1,4299	0,15619	9	1	12	2	27,40%	3,92%	100%	100%
61,1	gap junction alpha-1 protein [Bos taurus]	NP_776493.1	43 kDa	70,752	17,012	1,4299	0,15619	9	1	12	2	27,40%	3,92%	100%	100%
62	Cluster of P4HB protein [Bos taurus] (AAI46272.1)	AAI46272.1 [4]	57 kDa	0	154,32	0	1,5811	0	15	0	21	0	44,70%	0	100%
62,1	P4HB protein [Bos taurus]	AAI46272.1 (+1)	57 kDa	0	106,24	0	2,1952	0	15	0	21	0	44,70%	0	100%

62,2	PREDICTED: protein disulfide-isomerase [Orcinus orca]	XP_004275625.1	57 kDa	0	21,276	0	1,7104	0	13	0	18	0	34,30%	0	100%
62,3	unnamed protein product [Homo sapiens]	CAA28775.1	57 kDa	0	12,2	0	0,83765	0	8	0	11	0	16,10%	0	99%
63	Cluster of L-lactate dehydrogenase B chain isoform LDHB [Bos taurus] (NP_776525.2)	NP_776525.2 [7]	37 kDa	0	139,74	0	0,89093	0	15	0	20	0	35,50%	0	100%
63,1	PREDICTED: L-lactate dehydrogenase A chain-like isoform X1 [Pantholops hodgsonii]	XP_005983389.1	37 kDa	0	31,181	0	2,3261	0	10	0	14	0	35,50%	0	100%
63,2	L-lactate dehydrogenase B chain isoform LDHB [Bos taurus]	NP_776525.2 (+2)	37 kDa	0	50,568	0	0,53033	0	4	0	5	0	15,00%	0	100%
63,3	PREDICTED: L-lactate dehydrogenase C chain isoform X1 [Condylura cristata]	XP_004683052.1	36 kDa	0	4,8653	0	0,18708	0	2	0	2	0	6,33%	0	99%
63,4	hypothetical protein PANDA_015812, partial [Ailuropoda melanoleuca]	EFB16717.1	37 kDa	0	9,2685	0	0,67165	0	5	0	6	0	15,10%	0	100%
63,5	PREDICTED: L-lactate dehydrogenase A chain isoform X1 [Jaculus jaculus]	XP_004650857.1	40 kDa	0	40,272	0	0,73947	0	5	0	7	0	15,00%	0	100%
64	Cluster of integrin alpha-6 precursor [Bos taurus] (NP_001103451.1)	NP_001103451.1 [4]	119 kDa	107,9	0	0,23207	0	10	0	11	0	15,20%	0,00%	100%	98%
64,1	integrin alpha-6 precursor [Bos taurus]	NP_001103451.1 (+2)	119 kDa	105,26	0	0,34909	0	10	0	11	0	15,20%	0,00%	100%	93%
64,2	integrin alpha-6 isoform X2 [Dasypus novemcinctus]	XP_004450095.1	119 kDa	2,6405	0	0,11504	0	4	0	4	0	5,69%	0	97%	0
65	Cluster of PREDICTED: 40S ribosomal protein S11 [Ochotona princeps] (XP_004597224.1)	XP_004597224.1	18 kDa	23,789	0	0,97252	0	3	0	4	0	26,10%	0	100%	0
65,1	PREDICTED: 40S ribosomal protein S11 [Ochotona princeps]	XP_004597224.1	18 kDa	22,84	0	0,97252	0	3	0	4	0	26,10%	0	100%	0
66	receptor for retinol uptake STRA6 [Bos taurus]	NP_001069198.1 (+7)	74 kDa	87,508	24,302	0,30082	0,13726	5	3	6	3	8,83%	5,24%	100%	100%
67	Cluster of Annexin A2 [Bos taurus] (AAI02517.1)	AAI02517.1 [7]	39 kDa	8,4959	162,83	0,18057	2,7914	2	19	2	22	7,96%	64,00%	100%	100%
67,1	Annexin A2 [Bos taurus]	AAI02517.1 (+5)	39 kDa	8,4274	144,19	0,18057	4,5483	2	17	2	20	7,96%	64,00%	100%	100%
67,2	PREDICTED: LOW QUALITY PROTEIN: annexin A2-like [Echinops telfairi]	XP_012862251.1	40 kDa	0	18,218	0	1,0345	0	8	0	9	0	30,50%	0	100%
68	Cluster of estradiol 17-beta-dehydrogenase 1 [Bos taurus] (NP_001095835.1)	NP_001095835.1	34 kDa	16,142	104,5	0,2064	3,7953	1	14	2	17	4,73%	67,50%	100%	100%
68,1	estradiol 17-beta-dehydrogenase 1 [Bos taurus]	NP_001095835.1	34 kDa	16,142	104,48	0,2064	3,7953	1	14	2	17	4,73%	67,50%	100%	100%
69	Cluster of 60S ribosomal protein L14 [Bos taurus] (NP_001029846.1)	NP_001029846.1 [10]	23 kDa	88,357	0	0,52313	0	5	0	5	0	23,80%	0	100%	0
69,1	60S ribosomal protein L14 [Bos taurus]	NP_001029846.1 (+1)	23 kDa	40,194	0	0,71094	0	4	0	4	0	23,80%	0	100%	0
69,2	PREDICTED: 60S ribosomal protein L14-like isoform X1 [Pantholops hodgsonii]	XP_005970482.1 (+1)	24 kDa	16,504	0	0,1433	0	1	0	1	0	5,63%	0	95%	0
69,3	60S ribosomal protein L14, partial [Bos mutus]	ELR59746.1 (+5)	23 kDa	31,659	0	0,71515	0	4	0	4	0	23,60%	0	100%	0
70	Cluster of PREDICTED: 60S ribosomal protein L3 [Ursus maritimus] (XP_008703769.1)	XP_008703769.1 [3]	50 kDa	90,056	0	0,71634	0	6	0	8	0	18,10%	0,00%	100%	17%
70,1	PREDICTED: 60S ribosomal protein L3 [Ursus maritimus]	XP_008703769.1	50 kDa	82,677	0	0,56333	0	5	0	7	0	10,70%	0,00%	100%	17%
70,2	PREDICTED: 60S ribosomal protein L3 isoform X1 [Orycteropus afer afer]	XP_007939869.1 (+1)	46 kDa	7,3795	0	0,86935	0	6	0	8	0	18,10%	0	100%	0
71	Cluster of integrin alpha-2 precursor [Bos taurus] (NP_001159971.1)	NP_001159971.1 [6]	130 kDa	85,809	0	0,2527	0	9	0	9	0	10,80%	0,00%	100%	97%
71,1	integrin alpha-2 precursor [Bos taurus]	NP_001159971.1 (+5)	130 kDa	85,805	0	0,2527	0	9	0	9	0	10,80%	0,00%	100%	97%
72	Cluster of ribosomal protein L18, partial [Bos taurus] (ABD77172.1)	ABD77172.1 [39]	19 kDa	152,08	0	2,1695	0	6	0	7	0	34,30%	0	100%	0
72,1	ribosomal protein L18, partial [Bos taurus]	ABD77172.1 (+38)	19 kDa	147,98	0	2,1695	0	6	0	7	0	34,30%	0	100%	0
73	Cluster of adipocyte plasma membrane-associated protein isoform X1 [Bos taurus] (XP_024856500.1)	XP_024856500.1 [4]	43 kDa	57,772	41,314	0,42493	0,50053	6	10	8	11	12,30%	28,30%	100%	100%
73,1	APMAP [Cervus elaphus hippelaphus]	OWK02950.1 (+1)	46 kDa	18,789	6,1765	0,51362	0,60669	5	6	6	7	12,30%	21,90%	99%	100%
73,2	PREDICTED: adipocyte plasma membrane-associated protein [Ceratotherium simum simum]	XP_004441986.1	46 kDa	22,417	8,4703	0,41275	0,22501	4	3	5	3	9,64%	8,43%	100%	100%
73,3	adipocyte plasma membrane-associated protein isoform X1 [Bos taurus]	XP_024856500.1	43 kDa	16,517	26,644	0,34843	0,66989	4	7	4	7	9,71%	28,30%	100%	100%
74	Cluster of triosephosphate isomerase [Bos taurus] (NP_001013607.1)	NP_001013607.1 [10]	27 kDa	0	145,81	0	8,1427	0	21	0	27	0	74,30%	0	100%
74,1	triosephosphate isomerase [Bos taurus]	NP_001013607.1 (+7)	27 kDa	0	94,55	0	15,568	0	18	0	24	0	74,30%	0	100%
74,2	PREDICTED: triosephosphate isomerase [Tupaia chinensis]	XP_006170851.1	30 kDa	0	35,573	0	5,3546	0	14	0	18	0	58,90%	0	100%
74,3	LOW QUALITY PROTEIN: triosephosphate isomerase-like [Myotis lucifugus]	XP_014309162.1	27 kDa	0	9,6066	0	3,5053	0	10	0	13	0	50,00%	0	98%
75	Cluster of heterogeneous nuclear ribonucleoprotein U [Bos taurus] (NP_001070388.2)	NP_001070388.2 [27]	90 kDa	62,87	0	0,2406	0	6	0	7	0	12,60%	0	100%	0
75,1	heterogeneous nuclear ribonucleoprotein U [Bos taurus]	NP_001070388.2 (+24)	90 kDa	37,283	0	0,28514	0	6	0	7	0	12,60%	0	100%	0
75,2	PREDICTED: heterogeneous nuclear ribonucleoprotein U isoform X1 [Hipposideros armiger]	XP_019479393.1 (+1)	91 kDa	15,312	0	0,19607	0	4	0	5	0	7,76%	0	98%	0
76	clusterin preproprotein [Bos taurus]	NP_776327.1	51 kDa	63,719	0	0,45847	0	5	0	6	0	12,80%	0	100%	0
77	Cluster of heat shock protein HSP 90-alpha [Pan troglodytes] (BAK63243.1)	BAK63243.1	85 kDa	0,25812	119,05	0	0,50932	1	13	1	13	1,91%	15,00%	94%	100%
77,1	heat shock protein HSP 90-alpha [Pan troglodytes]	BAK63243.1	85 kDa	0,23921	105,88	0,038994	0,50932	1	11	1	11	1,91%	15,00%	86%	100%
78	Cluster of ribosomal protein S4 Y-linked 1, partial [Macaca mulatta] (ACL51673.1)	ACL51673.1 [4]	29 kDa	66,252	0	0,75569	0	8	0	8	0	20,10%	0,00%	100%	100%
78,1	ribosomal protein S4 Y-linked 1, partial [Macaca mulatta]	ACL51673.1 (+1)	29 kDa	9,4489	0	0,91703	0	6	0	6	0	17,40%	0	100%	0
78,2	40S ribosomal protein S4, X isoform-like [Microcebus murinus]	XP_012597174.1	30 kDa	36,205	0	1,0957	0	7	0	7	0	20,10%	0,00%	100%	60%
78,3	Actin-related protein 3B [Myotis brandtii]	EPQ16302.1	71 kDa	20,593	0	0,25436	0	5	0	5	0	6,70%	0,00%	99%	98%
79	Cluster of annexin A4 [Bos taurus] (NP_001001440.2)	NP_001001440.2 [2]	36 kDa	61,17	59,532	0,70426	1,3943	6	10	6	10	20,40%	38,60%	100%	100%
79,1	annexin A4 [Bos taurus]	NP_001001440.2 (+1)	36 kDa	60,664	55,621	0,70426	1,3943	6	10	6	10	20,40%	38,60%	100%	100%
80	Cluster of ribosomal protein L5 [Bos taurus] (AAX46329.1)	AAX46329.1 [8]	33 kDa	57,772	12,151	0,61715	0,45789	4	4	5	4	15,30%	21,50%	100%	100%
80,1	ribosomal protein L5 [Bos taurus]	AAX46329.1 (+7)	33 kDa	57,638	12,094	0,61715	0,45789	4	4	5	4	15,30%	21,50%	100%	100%
81	TPA: 40S ribosomal protein S6, partial [Bos taurus]	DAA26959.1 (+31)	24 kDa	88,357	0	1,1738	0	4	0	6	0	22,60%	0	100%	0
82	Cluster of phosphoglycerate kinase 1 [Bos taurus] (NP_001029471.1)	NP_001029471.1 [4]	45 kDa	2,5488	109,36	0	1,463	1	15	1	17	3,60%	44,40%	100%	100%
82,1	phosphoglycerate kinase 1 [Bos taurus]	NP_001029471.1 (+1)	45 kDa	1,7285	76,808	0,074745	1,8882	1	13	1	15	3,60%	44,40%	91%	100%
82,2	RecName: Full=Phosphoglycerate kinase 1	P00559.2	45 kDa	0,38814	13,875	0,074556	1,3299	1	10	1	12	3,60%	41,00%	32%	100%
82,3	PREDICTED: phosphoglycerate kinase 1 [Rhinopithecus roxellana]	XP_010387813.1	45 kDa	0,38666	15,232	0,074556	1,1709	1	9	1	11	3,60%	32,40%	32%	100%
83	Cluster of heat shock protein HSP 90-beta [Bos taurus] (NP_001073105.1)	NP_001073105.1 [9]	83 kDa	3,9419	152,18	0	0,70439	3	14	3	15	3,87%	22,10%	87%	100%
83,1	heat shock protein HSP 90-beta [Bos taurus]	NP_001073105.1 (+8)	83 kDa	3,9308	151,66	0,12375	0,70439	3	13	3	14	3,87%	22,10%	86%	100%
84	Cluster of immunoglobulin lambda-1 light chain-like isoform X2 [Bos taurus] (XP_024833611.1)	XP_024833611.1 [4]	26 kDa	95,302	49,681	1,0782	0	5	3	5	6	25,30%	13,50%	100%	100%
84,1	immunoglobulin lambda-1 light chain-like isoform X2 [Bos taurus]	XP_024833611.1 (+3)	26 kDa	68,485	34,628	1,0782	1,6102	5	3	5	6	25,30%	13,50%	100%	92%
85	Cluster of PREDICTED: 40S ribosomal protein S2 isoform X1 [Bos mutus] (XP_005886871.2)	XP_005886871.2	33 kDa	23,789	0	0,61463	0	5	0	7	0	12,20%	0,00%	100%	94%
85,1	PREDICTED: 40S ribosomal protein S2 isoform X1 [Bos mutus]	XP_005886871.2	33 kDa	22,865	0	0,61463	0	4	0	5	0	12,20%	0,00%	100%	80%
86	Cluster of cytoskeleton-associated protein 4 [Bos taurus] (XP_002687704.3)	XP_002687704.3 [2]	65 kDa	32,284	63,186	0,22152	0,53201	4	12	4	14	10,20%	28,50%	100%	100%
86,1	cytoskeleton-associated protein 4 [Bos taurus]	XP_002687704.3	65 kDa	32,284	56,717	0,22152	0,71452	4	11	4	11	10,20%	28,50%	100%	100%
86,2	LOW QUALITY PROTEIN: cytoskeleton-associated protein 4, partial [Loxodonta africana]	XP_010599848.2	53 kDa	0	6,4242	0	0,3495	0	3	0	5	0	14,20%	0	100%
87	Cluster of PREDICTED: ceruloplasmin isoform X3 [Bos indicus] (XP_019816622.1)	XP_019816622.1 [2]	99 kDa	27,187	12,151	0,17748	0,10055	5	3	5	3	5,98%	4,94%	100%	100%
87,1	PREDICTED: ceruloplasmin isoform X3 [Bos indicus]	XP_019816622.1 (+1)	99 kDa	27,184	12,151	0,17748	0,10055	5	3	5	3	5,98%	4,94%	100%	100%
88	PREDICTED: serum albumin [Erinaceus europaeus]	XP_007523612.1	69 kDa	8,4959	42,529	0,048315	0,047153	1	1	1	1	1,97%	1,97%	99%	100%
89	RecName: Full=Conglutinin; Flags: Precursor	P23805.2	38 kDa	40,78	10,936	0,52254	0,17896	5	2	5	2	17,50%	7,82%	100%	100%
90	PREDICTED: 40S ribosomal protein S16-like [Eptesicus fuscus]	XP_008140569.1	17 kDa	28,036	0	1,964	0	5	0	6	0	31,40%	0,00%	100%	74%
91	Cluster of PREDICTED: transmembrane emp24 domain-containing protein 10 [Mustela putorius furo] (XP_004	XP_004739292.1	25 kDa	17,841	10,936	0,66401	1,1173	4	5	4	6	16,40%	24,70%	100%	100%
91,1	PREDICTED: transmembrane emp24 domain-containing protein 10 [Mustela putorius furo]	XP_004739292.1	25 kDa	17,831	10,931	0,66401	1,1173	4	5	4	6	16,40%	24,70%	100%	100%
92	transitional endoplasmic reticulum ATPase [Bos taurus]	NP_001029466.1 (+21)	89 kDa	38,232	51,035	0,075332	0,19419	2	5	2	5	2,36%	7,44%	100%	100%
93	Cluster of ribosomal protein S13 [Ailuropoda melanoleuca] (ADT82659.1)	ADT82659.1 [6]	17 kDa	33,984	0	1,4607	0	4	0	5	0	21,20%	0	100%	0
93,1	ribosomal protein S13 [Ailuropoda melanoleuca]	ADT82659.1 (+5)	17 kDa	33,267	0	1,4607	0	4	0	5	0	21,20%	0	100%	0
94	ribosomal protein S9 [Homo sapiens]	AAA85659.1 (+14)	23 kDa	28,036	0	0,74603	0	3	0	4	0	9,28%	0	100%	0
95	TPA: peptidyl-prolyl cis-trans isomerase B [Bos taurus]	DAA25310.1 (+9)	23 kDa	37,189	19,304	0,73694	0,50104	4	3	4	3	22,60%	20,70%	100%	100%
96	Cluster of PREDICTED: annexin A6 isoform X3 [Bos indicus] (XP_019820826.1)	XP_019820826.1 [3]	75 kDa	16,992	17,012	0,19155	0,13428	7	3	7	3	15,10%	7,05%	100%	100%

96,1	PREDICTED: annexin A6 isoform X3 [Bos indicus]	XP_019820826.1	75 kDa	15,82	16,321	0,29387	0,13428	6	3	6	3	15,10%	7,05%	100%	100%
96,2	PREDICTED: annexin A6 isoform X1 [Equus asinus]	XP_014689892.1 (+1)	76 kDa	1,1011	0,53955	0,08922	0,042618	2	1	2	1	4,16%	1,78%	98%	65%
97	Cluster of PREDICTED: membrane-associated progesterone receptor component 1 isoform X1 [Bos mutus] (XP	XP_005887269.1 [3]	22 kDa	67,118	23,087	1,0619	1,0364	5	4	5	5	29,40%	19,10%	100%	100%
97,1	PREDICTED: membrane-associated progesterone receptor component 1 isoform X1 [Bos mutus]	XP_005887269.1 (+2)	22 kDa	67,065	23,069	1,0619	1,0364	5	4	5	5	29,40%	19,10%	100%	100%
98	Cluster of 60S ribosomal protein L10 [Bos taurus] (NP_777185.1)	NP_777185.1 [6]	25 kDa	6,7967	0	0,6714	0	4	0	4	0	22,00%	0	100%	0
98,1	60S ribosomal protein L10 [Bos taurus]	NP_777185.1 (+5)	25 kDa	5,2077	0	0,6714	0	4	0	4	0	22,00%	0	100%	0
99	Cluster of pyruvate kinase PKM [Bos taurus] (NP_001192656.1)	NP_001192656.1 [5]	58 kDa	0	54,681	0	0,5555	0	10	0	11	0	25,80%	0	100%
99,1	pyruvate kinase PKM [Bos taurus]	NP_001192656.1 (+3)	58 kDa	0	39,121	0	0,72406	0	9	0	10	0	25,80%	0	100%
99,2	pyruvate kinase PKM isoform X1 [Dasypus novemcinctus]	XP_004463783.1	58 kDa	0	14,803	0	0,38694	0	6	0	6	0	13,90%	0	100%
100	Cluster of clathrin light chain A isoform X3 [Bos taurus] (XP_005210119.1)	XP_005210119.1 [12]	23 kDa	70,516	0	0,49911	0	3	0	3	0	17,40%	0	100%	0
100,1	clathrin light chain A isoform X3 [Bos taurus]	XP_005210119.1 (+11)	23 kDa	70,193	0	0,49911	0	3	0	3	0	17,40%	0	100%	0
101	plasminogen precursor [Bos taurus]	NP_776376.1 (+1)	91 kDa	12,744	25,518	0,11259	0,07196	2	2	3	2	3,08%	3,08%	100%	100%
102	Cluster of ribosomal protein S26 [Mus musculus] (AAB07729.1)	AAB07729.1 [44]	13 kDa	19,541	0	1,5573	0	4	0	4	0	44,30%	0	100%	0
102,1	ribosomal protein S26 [Mus musculus]	AAB07729.1 (+43)	13 kDa	19,26	0	1,5573	0	4	0	4	0	44,30%	0	100%	0
103	Cluster of PREDICTED: NADH-cytochrome b5 reductase 3 isoform X1 [Ovis aries] (XP_012031122.1)	XP_012031122.1 [2]	32 kDa	14,443	61,971	0,22271	1,6822	2	8	2	10	4,68%	32,70%	100%	100%
103,1	PREDICTED: NADH-cytochrome b5 reductase 3 isoform X1 [Ovis aries]	XP_012031122.1 (+1)	32 kDa	14,298	61,653	0,22271	1,6822	2	8	2	10	4,68%	32,70%	100%	100%
104	Cluster of TPA: adaptor-related protein complex 2, alpha 1 subunit [Bos taurus] (DAA19706.1)	DAA19706.1 [60]	108 kDa	45,878	0	0,12773	0	4	0	4	0	5,00%	0	100%	0
104,1	TPA: adaptor-related protein complex 2, alpha 1 subunit [Bos taurus]	DAA19706.1 (+59)	108 kDa	45,702	0	0,12773	0	4	0	4	0	5,00%	0	100%	0
105	Cluster of aldose reductase [Bos taurus] (NP_001012537.1)	NP_001012537.1 [3]	36 kDa	14,443	67,314	0,092846	1,8438	2	12	2	13	3,49%	50,20%	100%	100%
105,1	RecName: Full=Aldose reductase; Short=AR; AltName: Full=20-alpha-hydroxysteroid dehydrogenase; Short=	P16116.1	36 kDa	7,6463	36,615	0,093138	1,8528	1	11	1	12	3,49%	50,20%	100%	100%
105,2	aldose reductase [Bos taurus]	NP_001012537.1 (+1)	36 kDa	6,7967	30,577	0,092554	1,8349	1	11	1	12	3,48%	50,00%	100%	100%
106	Cluster of 60S ribosomal protein L13 [Bos taurus] (NP_001015543.1)	NP_001015543.1 [10]	24 kDa	22,939	0	0,68088	0	5	0	5	0	26,50%	0	100%	0
106,1	60S ribosomal protein L13 [Bos taurus]	NP_001015543.1 (+6)	24 kDa	15,612	0	0,67896	0	4	0	4	0	26,50%	0	100%	0
106,2	PREDICTED: 60S ribosomal protein L13-like isoform X1 [Elephantulus edwardii]	XP_006879341.1 (+2)	24 kDa	7,3229	0	0,6828	0	4	0	4	0	26,50%	0	100%	0
107	Cluster of dolichyl-diphosphooligosaccharide--protein glycosyltransferase subunit 2 precursor [Bos taurus] (NI	NP_001029776.1 [14]	69 kDa	6,7967	71,692	0,097952	0,20044	2	4	2	4	7,92%	12,00%	100%	100%
107,1	dolichyl-diphosphooligosaccharide--protein glycosyltransferase subunit 2 precursor [Bos taurus]	NP_001029776.1 (+13)	69 kDa	6,7967	71,668	0,097952	0,20044	2	4	2	4	7,92%	12,00%	100%	100%
108	Cluster of 60S ribosomal protein L18a-like [Papio anubis] (XP_021793737.1)	XP_021793737.1 [2]	21 kDa	21,24	0	1,1379	0	6	0	6	0	29,70%	0	100%	0
108,1	60S ribosomal protein L18a-like [Papio anubis]	XP_021793737.1	21 kDa	8,1057	0	1,1238	0	5	0	5	0	28,40%	0	100%	0
108,2	60S ribosomal protein L18a [Heterocephalus glaber]	XP_004873387.2	25 kDa	13,134	0	1,1519	0	5	0	5	0	29,70%	0	100%	0
109	Cluster of PREDICTED: voltage-dependent anion-selective channel protein 1 isoform X1 [Camelus ferus] (XP_0	XP_014410440.1 [3]	32 kDa	0	97,607	0	4,1604	0	13	0	17	0,00%	58,70%	92%	100%
109,1	PREDICTED: voltage-dependent anion-selective channel protein 1 isoform X1 [Camelus ferus]	XP_014410440.1 (+2)	32 kDa	0	78,431	0	4,1604	0	13	0	17	0,00%	58,70%	79%	100%
110	Cluster of 60 kDa heat shock protein, mitochondrial [Bos taurus] (NP_001160080.1)	NP_001160080.1 [4]	61 kDa	0	99,64	0	1,5454	0	16	0	19	0	37,90%	0	100%
110,1	60 kDa heat shock protein, mitochondrial [Bos taurus]	NP_001160080.1 (+3)	61 kDa	0	88,593	0	1,5454	0	15	0	18	0	37,90%	0	100%
111	solute carrier family 29 (nucleoside transporters), member 1, partial [Bos taurus]	ABG67048.1 (+3)	49 kDa	20,39	14,581	0,21651	0,065935	2	1	3	1	5,11%	3,78%	100%	100%
112	Cluster of ornithine aminotransferase, mitochondrial precursor [Bos taurus] (NP_001029412.1)	NP_001029412.1 [5]	48 kDa	0	85,059	0	1,5035	0	12	0	14	0	43,10%	0	100%
112,1	ornithine aminotransferase, mitochondrial precursor [Bos taurus]	NP_001029412.1 (+4)	48 kDa	0	83,435	0	1,5035	0	12	0	14	0	43,10%	0	100%
113	prohibitin-2 [Bos taurus]	NP_001039663.1 (+1)	33 kDa	0	80,198	0	4,4337	0	17	0	18	0	66,90%	0	100%
114	Cluster of LOW QUALITY PROTEIN: GTP-binding nuclear protein Ran [Neophocaena asiaeorientalis asiaeorient	XP_024614458.1	24 kDa	10,195	13,366	0,47279	0,65892	2	3	3	4	11,60%	16,70%	100%	100%
114,1	LOW QUALITY PROTEIN: GTP-binding nuclear protein Ran [Neophocaena asiaeorientalis asiaeorientalis]	XP_024614458.1	24 kDa	10,143	13,254	0,47279	0,65892	2	3	3	4	11,60%	16,70%	100%	100%
115	14-3-3 protein epsilon [Cervus nippon]	AVX48934.1 (+4)	29 kDa	0	75,906	0	3,9663	0	12	0	15	0	47,50%	0	100%
116	calnexin precursor [Bos taurus]	NP_001099082.1 (+1)	68 kDa	7,6463	14,581	0,21012	0,15012	4	3	4	3	6,07%	8,09%	100%	100%
117	similar to ribosomal protein L30, partial [Bos taurus]	BAC56497.1 (+29)	18 kDa	53,524	0	1,0127	0	4	0	4	0	23,60%	0	100%	0
118	immunoglobulin lambda light chain variable region, partial [Bos taurus]	AAA83135.1	11 kDa	40,259	15,579	1,3505	1,318	3	3	3	3	49,00%	49,00%	100%	100%
119	Cluster of filamin-A [Bos taurus] (NP_001193443.1)	NP_001193443.1 [7]	281 kDa	0	48,605	0	0,058411	0	5	0	5	0	2,68%	0	100%
119,1	filamin-A [Bos taurus]	NP_001193443.1 (+6)	281 kDa	0	48,084	0	0,058411	0	5	0	5	0	2,68%	0	100%
120	PREDICTED: histone H2A.V [Bos mutus]	XP_014332511.1 (+62)	15 kDa	90,803	0	1,3274	0	2	0	4	0	16,90%	0,00%	100%	12%
121	Cluster of LOW QUALITY PROTEIN: voltage-dependent anion-selective channel protein 2-like [Neophocaena as	XP_024611830.1 [3]	32 kDa	2,5488	75,338	0,10544	1,0889	1	10	1	11	3,40%	44,20%	100%	100%
121,1	LOW QUALITY PROTEIN: voltage-dependent anion-selective channel protein 2-like [Neophocaena asiaeorie	XP_024611830.1	32 kDa	2,5488	17,317	0,10544	0,21654	1	2	1	2	3,38%	7,77%	99%	100%
121,2	PREDICTED: voltage-dependent anion-selective channel protein 2 isoform X2 [Orcinus orca]	XP_004273119.1 (+1)	32 kDa	0	44,788	0,10582	1,9613	0	9	0	10	3,40%	44,20%	17%	100%
122	peroxiredoxin-2 [Bos taurus]	NP_777188.1	22 kDa	0	99,64	0	3,691	0	9	0	11	0	41,70%	0	100%
123	Cluster of aspartate aminotransferase, mitochondrial [Bos taurus] (NP_777231.1)	NP_777231.1 [5]	48 kDa	0	75,338	0	1,7041	0	16	0	16	0,00%	49,10%	41%	100%
123,1	aspartate aminotransferase, mitochondrial [Bos taurus]	NP_777231.1 (+4)	48 kDa	0	72,513	0	1,7041	0	15	0	15	0	49,10%	0	100%
124	ribosomal protein S5 [Homo sapiens]	AAA85658.1 (+8)	23 kDa	51,825	25,518	0,3184	0,71922	2	3	2	4	13,70%	15,20%	100%	100%
125	Cluster of AP-2 complex subunit beta [Carlito syrichta] (XP_021562796.1)	XP_021562796.1	106 kDa	23,789	0	0,20247	0	4	0	6	0	5,89%	0	100%	0
125,1	AP-2 complex subunit beta [Carlito syrichta]	XP_021562796.1	106 kDa	21,317	0	0,20247	0	4	0	6	0	5,89%	0	100%	0
126	TPA: CD81 antigen, partial [Bos taurus]	DAA13541.1 (+9)	23 kDa	45,028	0	0,96208	0	4	0	5	0	29,00%	0	100%	0
127	Cluster of fructose-bisphosphate aldolase A [Bos taurus] (NP_001095385.1)	NP_001095385.1 [5]	39 kDa	0	59,093	0	1,3689	0	15	0	16	0	60,20%	0	100%
127,1	fructose-bisphosphate aldolase A [Bos taurus]	NP_001095385.1 (+2)	39 kDa	0	42,721	0	2,051	0	13	0	14	0	60,20%	0	100%
127,2	LOW QUALITY PROTEIN: fructose-bisphosphate aldolase A [Castor canadensis]	XP_020014532.1	45 kDa	0	7,6026	0	1,0091	0	10	0	10	0	39,50%	0	100%
127,3	PREDICTED: LOW QUALITY PROTEIN: fructose-bisphosphate aldolase A-like [Macaca fascicularis]	XP_015299582.1	39 kDa	0	7,5308	0	1,0465	0	9	0	9	0	42,30%	0	100%
128	unnamed protein product [Homo sapiens]	BAG34735.1 (+17)	16 kDa	12,744	0	0,21686	0	1	0	1	0	5,88%	0	100%	0
129	Apolipoprotein E [Bos taurus]	AAI34730.1 (+10)	36 kDa	55,223	1,2151	0,19357	0,090328	2	1	2	1	7,91%	4,43%	100%	99%
130	similar to ribosomal protein L17, partial [Bos taurus]	BAC56477.1 (+57)	21 kDa	16,992	0	0,57222	0	3	0	3	0	24,00%	0	100%	0
131	Cluster of 3-hydroxyacyl-CoA dehydrogenase type-2 [Bos taurus] (NP_776759.1)	NP_776759.1 [2]	27 kDa	0	83,843	0	2,4949	0	11	0	16	0	52,90%	0	100%
131,1	3-hydroxyacyl-CoA dehydrogenase type-2 [Bos taurus]	NP_776759.1	27 kDa	0	74,093	0	3,9956	0	9	0	14	0	52,90%	0	100%
131,2	PREDICTED: 3-hydroxyacyl-CoA dehydrogenase type-2 [Condylura cristata]	XP_004695060.1	27 kDa	0	9,6267	0	0,99421	0	5	0	6	0	30,70%	0	100%
132	Cluster of PREDICTED: prohibitin [Odobenus rosmarus divergens] (XP_004395188.1)	XP_004395188.1 [3]	30 kDa	0	58,326	0	2,6025	0	18	0	18	0	76,10%	0	100%
132,1	PREDICTED: prohibitin [Odobenus rosmarus divergens]	XP_004395188.1	30 kDa	0	28,874	0	2,9081	0	13	0	13	0	68,80%	0	100%
132,2	prohibitin [Ictidomys tridecemlineatus]	XP_005321751.1	30 kDa	0	18,438	0	3,3418	0	14	0	14	0	76,10%	0	100%
132,3	PREDICTED: prohibitin-like isoform X1 [Eptesicus fuscus]	XP_008136879.1	30 kDa	0	10,938	0	1,5576	0	9	0	9	0	46,30%	0	100%
133	Cluster of PREDICTED: 40S ribosomal protein SA-like [Saimiri boliviensis boliviensis] (XP_010348245.1)	XP_010348245.1 [2]	33 kDa	34,833	48,168	0,21309	0,46061	2	6	2	6	10,20%	24,70%	100%	100%
133,1	PREDICTED: 40S ribosomal protein SA-like [Saimiri boliviensis boliviensis]	XP_010348245.1	33 kDa	0	3,1261	0	0,46152	0	4	0	4	0	24,70%	0	99%
133,2	40S ribosomal protein SA-like [Physeter catodon]	XP_007122195.1	33 kDa	32,402	37,739	0,21309	0,4597	2	4	2	4	10,20%	18,30%	100%	100%
134	apolipoprotein R [Bos taurus]	XP_002693919.1	22 kDa	28,036	9,721	0,33454	0,52821	1	2	2	3	8,72%	15,40%	100%	100%
135	Cluster of ribosomal protein L15 [Bos taurus] (AAX08723.1)	AAX08723.1 [36]	24 kDa	25,488	0	0,68669	0	4	0	4	0	19,60%	0	100%	0
135,1	ribosomal protein L15 [Bos taurus]	AAX08723.1 (+35)	24 kDa	25,436	0	0,68669	0	4	0	4	0	19,60%	0	100%	0
136	IgG2a heavy chain constant region, partial [Bos taurus]	AAB37380.1	36 kDa	71,796	61,191	0,86219	2,1142	5	10	7	13	19,60%	48,20%	100%	100%
137	40S ribosomal protein S7 [Bos mutus]	ELR60909.1 (+23)	22 kDa	21,24	6,0756	0,5254	0,51277	2	3	3	3	17,80%	25,40%	100%	100%
138	Cluster of syntenin [Bos taurus] (ABQ12975.1)	ABQ12975.1 [9]	32 kDa	40,78	0	0,34954	0	5	0	5	0	19,10%	0	100%	0

138,1	syntenin [Bos taurus]	ABQ12975.1 (+6)	32 kDa	33,983	0	0,4805	0	4	0	4	0	19,10%	0	100%	0
138,2	syntenin-1 isoform X2 [Loxodonta africana]	XP_003408366.1 (+1)	32 kDa	6,6926	0	0,21859	0	2	0	2	0	10,80%	0	100%	0
139	hypothetical protein M91_00905, partial [Bos mutus]	ELR50059.1 (+33)	12 kDa	30,585	0	0,65264	0	2	0	2	0	25,90%	0	100%	0
140	Coagulation factor II (thrombin) [Bos taurus]	AAI05202.1 (+5)	70 kDa	13,593	40,099	0,046932	0,093731	1	2	1	2	2,24%	3,52%	100%	100%
141	hemopexin precursor [Bos taurus]	NP_001029784.1	52 kDa	8,4959	4,8605	0,13125	0,19825	1	2	2	3	5,01%	7,84%	100%	100%
142	Cluster of dolichyl-diphosphooligosaccharide--protein glycosyltransferase 48 kDa subunit precursor [Bos tauru	NP_001094543.1 [12]	49 kDa	13,593	34,023	0,14096	0,37346	2	5	2	6	5,47%	12,80%	100%	100%
142,1	dolichyl-diphosphooligosaccharide--protein glycosyltransferase 48 kDa subunit precursor [Bos taurus]	NP_001094543.1 (+9)	49 kDa	9,9996	13,894	0,14096	0,38066	2	4	2	5	5,47%	12,80%	98%	100%
142,2	dolichyl-diphosphooligosaccharide--protein glycosyltransferase 48 kDa subunit [Phascolarctos cinereus]	XP_020828816.1 (+1)	50 kDa	3,2652	19,729	0,0659	0,36626	1	4	1	5	3,29%	12,70%	41%	100%
143	TPA: ribosomal protein L35a-like [Bos taurus]	DAA23035.1 (+9)	13 kDa	2,5488	0	0,26786	0	1	0	1	0	8,85%	0	100%	0
144	Cluster of immunoglobulin light chain variable region, partial [Bos taurus] (AAB66575.1)	AAB66575.1	11 kDa	43,85	23,305	0,30662	0	2	2	2	2	12,70%	12,70%	100%	100%
145	Cluster of PREDICTED: myoferlin isoform X1 [Bos indicus] (XP_019808467.1)	XP_019808467.1 [8]	236 kDa	16,142	1,2151	0,042847	0,013566	8	1	8	1	4,04%	0,53%	100%	97%
145,1	PREDICTED: myoferlin isoform X1 [Bos indicus]	XP_019808467.1 (+3)	236 kDa	13,25	1,2054	0,086321	0,013566	6	1	6	1	4,04%	0,53%	100%	95%
145,2	PREDICTED: myoferlin isoform X1 [Echinops telfairi]	XP_004701123.1 (+1)	235 kDa	0,7696	0	0,014	0	1	0	1	0	0,58%	0,00%	98%	34%
145,3	PREDICTED: myoferlin isoform X1 [Condylura cristata]	XP_004680676.1 (+1)	235 kDa	1,4846	0	0,02822	0	2	0	2	0	1,36%	0	98%	0
146	IgG3 heavy chain constant region, partial [Bos taurus]	AAC48762.1	39 kDa	6,7967	0	0,39077	0	3	0	4	0	16,20%	0,00%	100%	100%
147	60S ribosomal protein L21 [Mus caroli]	XP_021029256.1	19 kDa	22,939	0	0,65539	0	2	0	3	0	20,60%	0	100%	0
148	Cluster of similar to 40S ribosomal protein S18, partial [Bos taurus] (BAC56379.1)	BAC56379.1 [6]	15 kDa	7,6463	0	1,2232	0	3	0	4	0	31,30%	0	100%	0
148,1	similar to 40S ribosomal protein S18, partial [Bos taurus]	BAC56379.1 (+5)	15 kDa	7,3186	0	1,2232	0	3	0	4	0	31,30%	0	100%	0
149	heterogeneous nuclear ribonucleoprotein A3 [Bos taurus]	NP_001192358.1 (+62)	40 kDa	28,886	0	0,083877	0	1	0	1	0	5,79%	0	100%	0
150	Cluster of transmembrane protein 43 [Bos taurus] (NP_001095950.1)	NP_001095950.1 [7]	45 kDa	25,488	18,227	0,43064	0,23413	4	3	5	3	19,50%	16,80%	100%	100%
150,1	transmembrane protein 43 [Bos taurus]	NP_001095950.1 (+6)	45 kDa	25,429	18,116	0,43064	0,23413	4	3	5	3	19,50%	16,80%	100%	100%
151	transketolase [Bos taurus]	ABS45051.1 (+5)	65 kDa	0	35,239	0	0,27568	0	5	0	5	0	10,40%	0	100%
152	Cluster of tropomyosin alpha-4 chain isoform X3 [Bos taurus] (XP_005208569.1)	XP_005208569.1 [192]	29 kDa	0	31,593	0	0,58508	0	9	0	9	0	35,90%	0	100%
152,1	tropomyosin alpha-3 chain isoform Tpm3.2cy [Homo sapiens]	NP_001036816.1 (+21)	29 kDa	0	22,478	0	1,1264	0	7	0	7	0	35,90%	0	100%
152,2	tropomyosin alpha isoform [Rattus norvegicus]	AAK54241.1 (+141)	29 kDa	0	4,1674	0	0,24293	0	2	0	2	0	12,90%	0	100%
152,3	tropomyosin alpha-4 chain isoform X3 [Bos taurus]	XP_005208569.1 (+27)	29 kDa	0	4,9088	0	0,38595	0	3	0	3	0	18,50%	0	100%
153	Cluster of similar to ribosomal protein L26, partial [Bos taurus] (BAC56435.1)	BAC56435.1 [81]	15 kDa	28,886	0	0,49613	0	2	0	2	0	19,40%	0	100%	0
153,1	similar to ribosomal protein L26, partial [Bos taurus]	BAC56435.1 (+80)	15 kDa	27,441	0	0,49613	0	2	0	2	0	19,40%	0	100%	0
154	TPA: electron transfer flavoprotein subunit alpha, mitochondrial precursor [Bos taurus]	DAA17530.1	35 kDa	0	71,692	0	1,2368	0	8	0	9	0	39,30%	0	100%
155	Cluster of splicing factor, proline- and glutamine-rich [Bos taurus] (NP_001193220.1)	NP_001193220.1 [34]	76 kDa	27,187	12,151	0,16258	0	3	2	3	2	7,66%	4,64%	100%	100%
155,1	splicing factor, proline- and glutamine-rich [Bos taurus]	NP_001193220.1 (+32)	76 kDa	12,283	5,4901	0,1359	0,086551	3	2	3	2	5,37%	3,25%	95%	76%
155,2	splicing factor, proline- and glutamine-rich isoform X1 [Papio anubis]	XP_021776543.1	56 kDa	14,867	6,6446	0,18927	0,11959	3	2	3	2	7,66%	4,64%	98%	82%
156	Cluster of acetyl-CoA acetyltransferase, mitochondrial precursor [Bos taurus] (NP_001039540.1)	NP_001039540.1 [5]	45 kDa	0	48,296	0	0,64847	0	8	0	10	0	24,20%	0	100%
156,1	acetyl-CoA acetyltransferase, mitochondrial precursor [Bos taurus]	NP_001039540.1 (+3)	45 kDa	0	38,443	0	0,87908	0	7	0	9	0	24,20%	0	100%
156,2	PREDICTED: acetyl-CoA acetyltransferase, mitochondrial [Galeopterus variegatus]	XP_008579591.1	45 kDa	0	9,7899	0	0,41787	0	4	0	5	0	14,80%	0	100%
157	Cluster of PREDICTED: ATP synthase subunit gamma, mitochondrial isoform X1 [Bos mutus] (XP_005895558.1)	XP_005895558.1	33 kDa	0	60,756	0	2,1087	0	11	0	12	0	53,00%	0	100%
157,1	PREDICTED: ATP synthase subunit gamma, mitochondrial isoform X1 [Bos mutus]	XP_005895558.1	33 kDa	0	60,278	0	2,1087	0	11	0	12	0	53,00%	0	100%
158	Cluster of Ribosomal protein L27a [Bos taurus] (AAI09684.1)	AAI09684.1 [50]	17 kDa	11,894	0	0,4546	0	2	0	2	0	16,20%	0	100%	0
158,1	Ribosomal protein L27a [Bos taurus]	AAI09684.1 (+49)	17 kDa	10,551	0	0,4546	0	2	0	2	0	16,20%	0	100%	0
159	cholesterol side-chain cleavage enzyme, mitochondrial precursor [Bos taurus]	NP_788817.1 (+1)	60 kDa	0	30,378	0	0,77961	0	11	0	11	0	24,80%	0	100%
160	TPA: splicing factor, arginine/serine-rich 3-like [Bos taurus]	DAA29303.1 (+28)	19 kDa	8,4959	0	0,38426	0	2	0	2	0	18,30%	0	100%	0
161	transmembrane emp24 protein transport domain containing 9 [Bos taurus]	AAX09080.1 (+100)	27 kDa	24,638	18,227	0,26212	0,57788	2	4	2	4	8,09%	11,90%	100%	100%
162	procollagen-lysine,2-oxoglutarate 5-dioxygenase 2 precursor [Bos taurus]	NP_001094619.1 (+31)	88 kDa	0	38,884	0	0,11439	0	3	0	3	0	5,51%	0	100%
163	Cluster of erlin-1 [Bos taurus] (NP_001157504.1)	NP_001157504.1 [23]	39 kDa	36,532	9,721	0,17786	0,27149	2	3	2	3	6,90%	10,90%	100%	100%
163,1	erlin-1 [Bos taurus]	NP_001157504.1 (+22)	39 kDa	30,78	8,4994	0,17786	0,27149	2	3	2	3	6,90%	10,90%	98%	100%
164	Cluster of annexin A1 [Bos taurus] (NP_786978.2)	NP_786978.2 [5]	39 kDa	0	74,122	0	0,90286	0	10	0	12	0	32,90%	0	100%
164,1	annexin A1 [Bos taurus]	NP_786978.2 (+2)	39 kDa	0	70,2	0	1,4243	0	9	0	11	0	32,90%	0	100%
164,2	annexin A1 [Equus caballus]	NP_001075336.1 (+1)	39 kDa	0	3,8642	0	0,38137	0	3	0	4	0	8,67%	0	99%
165	60S ribosomal protein L35 [Bos taurus]	NP_001029667.1 (+68)	15 kDa	11,894	0	0,88528	0	3	0	3	0	18,70%	0	100%	0
166	hypothetical protein A6R68_19806 [Neotoma lepida]	OBS77805.1 (+4)	10 kDa	21,24	14,581	0,34591	0,33759	1	1	1	1	9,57%	9,57%	98%	99%
167	Cluster of LOW QUALITY PROTEIN: membrane-associated progesterone receptor component 2 [Physeter cato	XP_007121455.1 [2]	26 kDa	38,232	23,087	0,27299	0,42547	2	3	2	3	13,40%	13,80%	100%	100%
167,1	LOW QUALITY PROTEIN: membrane-associated progesterone receptor component 2 [Physeter catodon]	XP_007121455.1 (+1)	26 kDa	38,03	23,032	0,27299	0,42547	2	3	2	3	13,40%	13,80%	100%	100%
168	immunoglobulin lambda light chain constant region 3 allotypic variant IGLC3c, partial [Bos taurus]	AEM05849.1	11 kDa	45,305	6,3214	2,8347	1,8867	4	2	4	2	65,10%	51,90%	100%	100%
169	Cluster of TPA: 60S ribosomal protein L19, partial [Bos taurus] (DAA27107.1)	DAA27107.1 [49]	8 kDa	43,329	0	3,0329	0	4	0	4	0	38,40%	0	100%	0
169,1	TPA: 60S ribosomal protein L19, partial [Bos taurus]	DAA27107.1 (+48)	8 kDa	38,681	0	3,0329	0	4	0	4	0	38,40%	0	100%	0
170	glutathione S-transferase P [Bos taurus]	NP_803482.1	24 kDa	0	57,111	0	1,1906	0	6	0	6	0	49,50%	0	100%
171	Cluster of biliverdin reductase A [Bos taurus] (NP_001091040.1)	NP_001091040.1 [6]	34 kDa	0	54,681	0	1,5329	0	9	0	10	0,00%	37,50%	36%	100%
171,1	biliverdin reductase A [Bos taurus]	NP_001091040.1 (+5)	34 kDa	0	54,062	0	1,5329	0	9	0	10	0	37,50%	0	100%
172	similar to ribosomal protein L12, partial [Bos taurus]	BAC56456.1 (+46)	16 kDa	31,428	0	1,1996	0	4	0	4	0	37,90%	0	100%	0
173	Cluster of Ywhaq protein, partial [Mus musculus] (AAH80802.1)	AAH80802.1 [3]	30 kDa	3,3984	49,885	0	1,549	1	9	1	10	5,28%	38,50%	100%	100%
173,1	Ywhaq protein, partial [Mus musculus]	AAH80802.1 (+2)	30 kDa	2,6273	34,809	0,11183	1,549	1	8	1	9	5,28%	38,50%	79%	100%
174	Cluster of 40S ribosomal protein S3a-like [Microcebus murinus] (XP_012633752.2)	XP_012633752.2	30 kDa	13,593	0	0,37709	0	5	0	5	0	14,40%	0	100%	0
174,1	40S ribosomal protein S3a-like [Microcebus murinus]	XP_012633752.2	30 kDa	10,444	0	0,37709	0	3	0	3	0	14,40%	0	100%	0
175	Cluster of Aconitase 2, mitochondrial [Bos taurus] (AAI02643.1)	AAI02643.1 [8]	85 kDa	0	23,087	0	0,07706	0	2	0	2	0	3,97%	0	100%
175,1	Aconitase 2, mitochondrial [Bos taurus]	AAI02643.1 (+7)	85 kDa	0	21,205	0	0,07706	0	2	0	2	0	3,97%	0	100%
176	TPA: ribosomal protein S29-like [Bos taurus]	DAA19427.1 (+11)	7 kDa	8,4959	0	1,3393	0	2	0	2	0	33,90%	0	100%	0
177	Cluster of 26S proteasome non-ATPase regulatory subunit 2 [Bos taurus] (NP_001094667.1)	NP_001094667.1	100 kDa	11,894	20,657	0,032907	0,032115	1	1	1	1	1,65%	1,65%	99%	100%
178	Cluster of nicotinamide nucleotide transhydrogenase, partial [Bos taurus] (AAA21440.1)	AAA21440.1 [16]	108 kDa	0	31,593	0	0,10591	0	4	0	4	0	5,02%	0	100%
178,1	nicotinamide nucleotide transhydrogenase, partial [Bos taurus]	AAA21440.1 (+14)	108 kDa	0	6,3251	0	0,12439	0	4	0	4	0	5,02%	0	100%
178,2	NAD(P) transhydrogenase, mitochondrial [Desmodus rotundus]	XP_024427705.1	114 kDa	0	20,622	0	0,087434	0	3	0	3	0	3,59%	0	99%
179	Cluster of carboxypeptidase D [Bos taurus] (XP_010814170.2)	XP_010814170.2 [2]	153 kDa	41,63	0	0,043739	0	3	0	3	0	3,56%	0	100%	0
179,1	carboxypeptidase D [Bos taurus]	XP_010814170.2	153 kDa	39,925	0	0,043609	0	2	0	2	0	3,41%	0	100%	0
179,2	carboxypeptidase D [Meriones unguiculatus]	XP_021510547.1	152 kDa	1,3211	0	0,043868	0	2	0	2	0	3,56%	0	98%	0
180	high mobility group protein B1 [Bos taurus]	NP_788785.1 (+64)	25 kDa	0	24,302	0	0,64449	0	4	0	4	0	21,90%	0	100%
181	Cluster of proteasome subunit alpha type-1 [Bos taurus] (NP_001030387.1)	NP_001030387.1 [4]	30 kDa	0	18,227	0	0,88174	0	5	0	6	0,00%	24,00%	96%	100%
181,1	proteasome subunit alpha type-1 [Bos taurus]	NP_001030387.1 (+3)	30 kDa	0	16,403	0	0,88174	0	5	0	6	0,00%	24,00%	96%	100%
182	Cluster of phosphate carrier protein, mitochondrial precursor [Bos taurus] (NP_777082.1)	NP_777082.1 [118]	40 kDa	0	14,581	0	0,081168	0	1	0	1	0	3,31%	0	100%
183	60S ribosomal protein L9 [Bos taurus]	NP_001019640.2 (+5)	22 kDa	18,691	0	0,53799	0	3	0	3	0	24,00%	0	100%	0
184	TPA: ribosomal protein L23a-like [Bos taurus]	DAA22605.1 (+90)	18 kDa	37,382	0	0,41361	0	2	0	2	0	10,80%	0	100%	0

185	cytochrome b-c1 complex subunit 2, mitochondrial precursor [Bos taurus]	NP_777055.1 (+2)	48 kDa	0	47,39	0	0,6861	0	7	0	8	0	23,40%	0	100%
186	translocon-associated protein subunit delta precursor [Bos taurus]	NP_001033592.1 (+11)	19 kDa	32,284	0	0,64601	0	3	0	3	0	25,00%	0	100%	0
187	Cluster of glucosidase 2 subunit beta precursor [Bos taurus] (NP_788835.1)	NP_788835.1 [9]	60 kDa	2,5488	30,378	0,11315	0,23321	2	4	2	4	5,25%	9,76%	100%	100%
187,1	glucosidase 2 subunit beta precursor [Bos taurus]	NP_788835.1 (+8)	60 kDa	2,5488	30,297	0,11315	0,23321	2	4	2	4	5,25%	9,76%	100%	100%
188	lysosome-associated membrane glycoprotein 2 isoform 2 precursor [Bos taurus]	NP_001029742.1 (+20)	45 kDa	23,789	25,518	0,074745	0,072947	1	1	1	1	2,95%	2,95%	99%	99%
189	Cluster of complement component 5 [Bos taurus] (AKE14289.1)	AKE14289.1 [4]	189 kDa	13,593	0	0,035144	0	2	0	2	0	1,91%	0	100%	0
189,1	complement component 5 [Bos taurus]	AKE14289.1 (+3)	189 kDa	12,733	0	0,035144	0	2	0	2	0	1,91%	0	100%	0
190	Cluster of endothelin-converting enzyme 1 isoform X1 [Bos taurus] (XP_015315502.1)	XP_015315502.1 [10]	91 kDa	16,142	1,2151	0,15264	0	3	1	4	1	8,19%	2,23%	100%	89%
190,1	endothelin-converting enzyme 1 isoform X1 [Bos taurus]	XP_015315502.1 (+9)	91 kDa	15,134	1,125	0,15264	0,035304	3	1	4	1	8,19%	2,23%	100%	71%
191	Cluster of ribose-phosphate pyrophosphokinase 3 [Bos taurus] (NP_001095953.1)	NP_001095953.1 [3]	36 kDa	7,6463	1,2151	0,26825	0,093859	3	1	4	1	14,50%	5,03%	100%	100%
191,1	ribose-phosphate pyrophosphokinase 2 [Dasypus novemcinctus]	XP_004459179.1	35 kDa	7,3905	1,0322	0,44306	0,093859	3	1	4	1	14,50%	5,03%	100%	99%
191,2	ribose-phosphate pyrophosphokinase 3 [Bos taurus]	NP_001095953.1 (+1)	36 kDa	0,24343	0	0,093433	0	1	0	1	0	3,65%	0	95%	0
192	Cluster of PREDICTED: phosphatidylethanolamine-binding protein 1 [Ovis aries] (XP_004017427.1)	XP_004017427.1 [2]	21 kDa	0	53,465	0	3,3449	0	7	0	10	0	57,80%	0	100%
192,1	PREDICTED: phosphatidylethanolamine-binding protein 1 [Ovis aries]	XP_004017427.1 (+1)	21 kDa	0	48,048	0	3,3449	0	7	0	10	0	57,80%	0	100%
193	Cluster of PREDICTED: LOW QUALITY PROTEIN: phosphoglycerate mutase 1-like [Ovis aries musimon] (XP_012	XP_012002737.1 [3]	29 kDa	0	48,605	0	0,95869	0	7	0	10	0	26,00%	0	100%
193,1	PREDICTED: LOW QUALITY PROTEIN: phosphoglycerate mutase 1-like [Ovis aries musimon]	XP_012002737.1	29 kDa	0	22,679	0	0,54054	0	3	0	4	0	19,30%	0	100%
193,2	phosphoglycerate mutase 1 [Pteropus alecto]	XP_006925978.1 (+1)	29 kDa	0	11,688	0	1,3768	0	5	0	8	0	26,00%	0	95%
194	Cluster of acidic ribosomal phosphoprotein PO, partial [Bos taurus] (AAB65436.1)	AAB65436.1 [74]	32 kDa	29,736	10,936	0,21779	0,33541	2	3	2	3	11,90%	12,90%	100%	100%
194,1	acidic ribosomal phosphoprotein PO, partial [Bos taurus]	AAB65436.1 (+73)	32 kDa	28,186	9,6224	0,21779	0,33541	2	3	2	3	11,90%	12,90%	100%	100%
195	cation-dependent mannose-6-phosphate receptor precursor [Bos taurus]	NP_786973.1 (+58)	31 kDa	13,593	1,2151	0,22611	0,10478	2	1	2	1	12,50%	5,02%	100%	96%
196	Cluster of vimentin [Bos taurus] (AAA53661.1)	AAA53661.1 [4]	54 kDa	2,5488	32,808	0,12757	0,42255	2	6	2	6	6,01%	22,50%	100%	100%
196,1	vimentin [Bos taurus]	AAA53661.1 (+3)	54 kDa	2,5335	32,568	0,12757	0,42255	2	6	2	6	6,01%	22,50%	100%	100%
197	Cluster of peroxiredoxin-4 isoform X1 [Bos taurus] (XP_005228408.1)	XP_005228408.1 [5]	31 kDa	0	40,392	0	1,3457	0	8	0	11	0,00%	46,40%	97%	100%
197,1	peroxiredoxin-4 isoform X1 [Bos taurus]	XP_005228408.1 (+2)	31 kDa	0	32,932	0	2,0295	0	8	0	11	0,00%	46,40%	95%	100%
197,2	peroxiredoxin-4 isoform X2 [Dasypus novemcinctus]	XP_004449879.1 (+1)	31 kDa	0	5,2335	0	0,66201	0	4	0	5	0,00%	12,20%	12%	95%
198	complement C1q subcomponent subunit B precursor [Bos taurus]	NP_001040064.1 (+3)	26 kDa	12,744	0	0,2705	0	2	0	2	0	14,20%	0	100%	0
199	PREDICTED: vacuolar protein sorting-associated protein 13A isoform X1 [Macaca mulatta]	XP_014973424.1 (+3)	387 kDa	2,5488	7,2907	0,0084822	0,0082782	1	1	1	1	0,23%	0,23%	97%	97%
200	Cluster of ATP synthase F(0) complex subunit B1, mitochondrial precursor [Bos taurus] (NP_001033590.1)	NP_001033590.1 [5]	29 kDa	0	40,099	0	0,88054	0	7	0	9	0	25,00%	0	100%
200,1	ATP synthase F(0) complex subunit B1, mitochondrial precursor [Bos taurus]	NP_001033590.1 (+3)	29 kDa	0	37,393	0	1,3768	0	6	0	8	0	25,00%	0	100%
200,2	ATP synthase F(0) complex subunit B1, mitochondrial [Carlito syrichta]	XP_008067114.1	29 kDa	0	2,7064	0	0,38425	0	2	0	3	0	11,80%	0	99%
201	Cluster of ATP synthase subunit O, mitochondrial precursor [Bos taurus] (NP_776669.1)	NP_776669.1 [5]	23 kDa	0	38,884	0	1,5292	0	5	0	7	0	26,30%	0	100%
201,1	ATP synthase subunit O, mitochondrial precursor [Bos taurus]	NP_776669.1 (+4)	23 kDa	0	38,804	0	1,5292	0	5	0	7	0	26,30%	0	100%
202	14-3-3 protein zeta/delta, partial [Bos mutus]	ELR62763.1 (+27)	26 kDa	0	36,994	0	1,905	0	7	0	9	0	37,00%	0	100%
203	Cluster of hCG1994130, isoform CRA_b [Homo sapiens] (EAW50256.1)	EAW50256.1 [6]	29 kDa	9,3455	3,6454	0,11669	0,2409	1	2	1	2	3,19%	9,56%	100%	100%
203,1	hCG1994130, isoform CRA_b [Homo sapiens]	EAW50256.1 (+5)	29 kDa	6,8327	3,6454	0,11669	0,2409	1	2	1	2	3,19%	9,56%	100%	100%
204	Cluster of vesicular integral-membrane protein VIP36 precursor [Bos taurus] (NP_001094779.1)	NP_001094779.1 [88]	40 kDa	16,142	6,0756	0,17166	0,16753	2	2	2	2	6,13%	3,90%	100%	100%
204,1	vesicular integral-membrane protein VIP36 precursor [Bos taurus]	NP_001094779.1 (+87)	40 kDa	16,097	6,0577	0,17166	0,16753	2	2	2	2	6,13%	3,90%	100%	100%
205	Cluster of procollagen galactosyltransferase 1 precursor [Bos taurus] (NP_001092425.1)	NP_001092425.1 [6]	72 kDa	6,7967	31,593	0,04627	0,092379	1	2	1	2	1,28%	2,89%	97%	100%
205,1	procollagen galactosyltransferase 1 precursor [Bos taurus]	NP_001092425.1 (+5)	72 kDa	6,7777	31,529	0,04627	0,092379	1	2	1	2	1,28%	2,89%	97%	100%
206	Cluster of 60S ribosomal protein L11 isoform X1 [Bos taurus] (XP_005203175.1)	XP_005203175.1 [38]	21 kDa	27,187	0	0,34225	0	2	0	2	0	8,60%	0	100%	0
206,1	60S ribosomal protein L11 isoform X1 [Bos taurus]	XP_005203175.1 (+37)	21 kDa	26,828	0	0,34225	0	2	0	2	0	8,60%	0	100%	0
207	protein HP-20 homolog precursor [Bos taurus]	NP_001040049.1 (+8)	21 kDa	16,142	0	0,16397	0	1	0	1	0	12,00%	0	99%	0
208	Cluster of poly(rC)-binding protein 1-like [Piliocolobus tephrosceles] (XP_023082141.1)	XP_023082141.1 [2]	40 kDa	0,84959	23,087	0,083639	0,2758	1	5	1	5	2,93%	18,90%	96%	100%
208,1	poly(rC)-binding protein 1-like [Piliocolobus tephrosceles]	XP_023082141.1	40 kDa	0,84959	11,685	0,083639	0,36925	1	4	1	4	2,93%	18,90%	96%	100%
208,2	poly(rC)-binding protein 2 isoform 3 [Cricetulus griseus]	ERE72154.1	37 kDa	0	7,9218	0	0,18235	0	2	0	2	0	8,88%	0	97%
209	Cluster of lung cancer oncogene 7 [Homo sapiens] (AAO21313.1)	AAO21313.1 [24]	38 kDa	0,84959	26,733	0	0,93856	1	7	1	8	2,59%	25,90%	92%	100%
209,1	lung cancer oncogene 7 [Homo sapiens]	AAO21313.1 (+23)	38 kDa	0,75719	23,94	0,088133	0,93856	1	7	1	8	2,59%	25,90%	71%	100%
210	PREDICTED: ubiquitin-40S ribosomal protein S27a-like [Elephantulus edwardii]	XP_006888789.1	18 kDa	6,7967	0	0,41361	0	2	0	2	0	17,90%	0	100%	0
211	TPA: ribosomal protein S23-like [Bos taurus]	DAA24498.1 (+24)	17 kDa	22,939	0	0,20485	0	1	0	1	0	7,28%	0	100%	0
212	Cluster of citrate synthase, mitochondrial isoform X1 [Bos taurus] (XP_010803417.1)	XP_010803417.1 [2]	53 kDa	0	38,884	0	0,35166	0	6	0	6	0	10,60%	0	100%
212,1	Citrate synthase, mitochondrial [Macaca mulatta]	EHH20859.1	52 kDa	0	21,255	0	0,3555	0	5	0	5	0	10,30%	0	100%
212,2	citrate synthase, mitochondrial isoform X1 [Bos taurus]	XP_010803417.1	53 kDa	0	17,629	0	0,34783	0	5	0	5	0	10,60%	0	100%
213	Cluster of secretory carrier-associated membrane protein 2 [Bos taurus] (NP_001095640.1)	NP_001095640.1 [9]	37 kDa	21,24	0	0,19045	0	2	0	2	0	10,40%	0	100%	0
213,1	secretory carrier-associated membrane protein 2 [Bos taurus]	NP_001095640.1 (+8)	37 kDa	21,232	0	0,19045	0	2	0	2	0	10,40%	0	100%	0
214	alpha-1-acid glycoprotein precursor [Bos taurus]	CAH59718.2 (+2)	23 kDa	9,3455	10,936	0,14541	0,30422	1	2	1	2	5,94%	15,30%	99%	100%
215	Cluster of inter-alpha-trypsin inhibitor heavy chain H2 precursor [Bos taurus] (NP_001091485.1)	NP_001091485.1 [7]	106 kDa	6,7967	0	0,080499	0	4	0	4	0	4,65%	0	100%	0
215,1	inter-alpha-trypsin inhibitor heavy chain H2 precursor [Bos taurus]	NP_001091485.1 (+5)	106 kDa	5,571	0	0,096101	0	3	0	3	0	4,65%	0	100%	0
215,2	ITIH2, partial [Cervus elaphus hippelaphus]	OWK03472.1	103 kDa	1,1528	0	0,064898	0	2	0	2	0	3,35%	0	96%	0
216	protein disulfide-isomerase A4 precursor [Bos taurus]	NP_001039344.1 (+3)	73 kDa	1,6992	4,8605	0,045626	0,1397	1	3	1	3	1,87%	5,29%	100%	100%
217	Cluster of immediate early response 3 interacting protein 1, isoform CRA_a [Mus musculus] (EDL09478.1)	EDL09478.1 [22]	11 kDa	11,894	6,0756	0,31309	0,076634	1	1	1	1	19,80%	5,47%	100%	99%
218	TPA: proteasome alpha 2 subunit-like [Bos taurus]	DAA33550.1 (+9)	26 kDa	0	20,657	0	0,8155	0	5	0	5	0	38,50%	0	100%
219	vesicle-trafficking protein SEC22b precursor [Bos taurus]	NP_001069811.1 (+14)	29 kDa	17,841	9,721	0,55386	0,38256	3	3	3	3	17,10%	20,30%	100%	100%
220	PREDICTED: stress-70 protein, mitochondrial [Bos indicus]	XP_019820560.1 (+46)	74 kDa	0	1,2151	0	0,043784	0	1	0	1	0	2,06%	0	100%
221	Glutathione peroxidase 1 [Bos taurus]	AAI49309.1 (+1)	23 kDa	0	29,163	0	0,72363	0	4	0	4	0,00%	35,10%	80%	100%
222	vitamin D binding protein [Bos taurus]	AFO63001.1 (+2)	53 kDa	0	30,378	0	0,34371	0	3	0	5	0	11,40%	0	100%
223	Cluster of UDP-glucose:glycoprotein glucosyltransferase 1 isoform X1 [Bos taurus] (XP_002685277.1)	XP_002685277.1 [9]	178 kDa	0	2,4302	0	0,036447	0	2	0	2	0,00%	1,93%	94%	100%
223,1	UDP-glucose:glycoprotein glucosyltransferase 1 isoform X1 [Bos taurus]	XP_002685277.1 (+8)	178 kDa	0	2,381	0	0,036447	0	2	0	2	0,00%	1,93%	94%	100%
224	isocitrate dehydrogenase [NAD] subunit alpha, mitochondrial precursor [Bos taurus]	NP_777069.1 (+5)	40 kDa	0	25,518	0	0,60694	0	6	0	6	0	22,40%	0	100%
225	carbonic anhydrase 2 [Bos taurus]	NP_848667.1	29 kDa	0	19,442	0	0,70645	0	5	0	5	0	33,50%	0	100%
226	Ig lambda variable, partial [Felis catus]	ATI97539.1	20 kDa	7,6463	1,2151	0,16769	0,16365	1	1	1	1	7,29%	7,29%	98%	95%
227	Cluster of cathepsin D precursor [Bos taurus] (NP_001159993.1)	NP_001159993.1 [7]	45 kDa	0,84959	4,8605	0	0,15128	1	2	1	2	0,00%	8,54%	87%	100%
227,1	cathepsin D precursor [Bos taurus]	NP_001159993.1 (+6)	45 kDa	0	3,1787	0	0,15128	0	2	0	2	0	8,54%	0	100%
228	Cluster of adenylate kinase 2, mitochondrial [Bos taurus] (NP_776314.1)	NP_776314.1	26 kDa	0	19,442	0	1,0647	0	6	0	6	0	25,60%	0	100%
228,1	adenylate kinase 2, mitochondrial [Bos taurus]	NP_776314.1	26 kDa	0	19,44	0	1,0647	0	6	0	6	0	25,60%	0	100%
229	chondroitin sulfate proteoglycan 4 precursor [Bos taurus]	NP_001179711.2 (+1)	251 kDa	0	13,366	0	0,052206	0	3	0	4	0	1,85%	0	100%
230	immmunoglobulin lambda light chain variable region, partial [Equus caballus]	AIY24844.1	23 kDa	0,84959	0	0,1447	0	1	0	1	0	9,42%	0	96%	0
231	PREDICTED: malectin isoform X2 [Ovis aries musimon]	XP_011967785.1	32 kDa	12,744	8,5059	0,1062	0,34471	1	3	1	3	4,59%	14,10%	100%	100%
232	40S ribosomal protein S10 [Heterocephalus glaber]	EHB06972.1	25 kDa	26,337	0	0,29036	0	2	0	2	0	11,10%	0	100%	0
233	complement factor B precursor [Bos taurus]	NP_001035616.1 (+1)	85 kDa	11,894	13,366	0,078854	0,076957	2	2	2	2	3,15%	3,15%	100%	100%

234	60S ribosomal protein L36 [Bos taurus]	NP_001071607.1 (+19)	12 kDa	16,142	0	0,64564	0	1	0	2	0	11,40%	0	100%	0
235	Cluster of Peroxiredoxin-1, partial [Bos mutus] (ELR57459.1)	ELR57459.1 [5]	23 kDa	0	31,301	0	2,9404	0	7	0	10	0,00%	29,20%	53%	100%
235,1	Peroxiredoxin-1, partial [Bos mutus]	ELR57459.1 (+4)	23 kDa	0	30,603	0	2,9404	0	7	0	10	0,00%	29,20%	53%	100%
236	Cluster of PREDICTED: cytochrome c1, heme protein, mitochondrial [Orcinus orca] (XP_004265315.1)	XP_004265315.1	35 kDa	0	23,087	0	0,42452	0	4	0	5	0	19,60%	0	100%
236,1	PREDICTED: cytochrome c1, heme protein, mitochondrial [Orcinus orca]	XP_004265315.1	35 kDa	0	10,613	0	0,42452	0	3	0	4	0	19,60%	0	100%
237	Cluster of 40S ribosomal protein S17 [Bos taurus] (NP_001092680.1)	NP_001092680.1 [22]	16 kDa	9,3455	0	0,48803	0	2	0	2	0	25,20%	0	100%	0
237,1	40S ribosomal protein S17 [Bos taurus]	NP_001092680.1 (+21)	16 kDa	9,3067	0	0,48803	0	2	0	2	0	25,20%	0	100%	0
238	Cluster of rab GDP dissociation inhibitor beta [Bos taurus] (NP_001028934.1)	NP_001028934.1 [70]	50 kDa	0	29,163	0	0,36534	0	5	0	5	0	14,60%	0	100%
238,1	rab GDP dissociation inhibitor beta [Bos taurus]	NP_001028934.1 (+69)	50 kDa	0	24,826	0	0,36534	0	5	0	5	0	14,60%	0	100%
239	alpha-1-antiproteinase precursor [Bos taurus]	NP_776307.1 (+4)	46 kDa	0	29,163	0	0,4062	0	4	0	5	0	14,90%	0	100%
240	CDC42 protein [Bos taurus]	ACJ06401.1 (+26)	21 kDa	0	12,151	0	0,5446	0	2	0	3	0	14,10%	0	100%
241	copper-transporting ATPase 1 isoform X1 [Bos taurus]	XP_024843970.1 (+1)	164 kDa	10,195	0	0,040414	0	1	0	2	0	1,39%	0	100%	0
242	Cluster of endoplasmic reticulum resident protein 29 precursor [Bos taurus] (NP_001069739.1)	NP_001069739.1 [4]	29 kDa	11,045	17,012	0,24684	0,54054	2	4	2	4	9,69%	26,00%	100%	100%
242,1	endoplasmic reticulum resident protein 29 precursor [Bos taurus]	NP_001069739.1 (+3)	29 kDa	11,008	17,003	0,24684	0,54054	2	4	2	4	9,69%	26,00%	100%	100%
243	PREDICTED: LOW QUALITY PROTEIN: serine/arginine-rich splicing factor 1 [Ailuropoda melanoleuca]	XP_019651287.1	27 kDa	11,045	0	0,12404	0	1	0	1	0	6,61%	0,00%	100%	34%
244	Cluster of eukaryotic translation elongation factor 1 gamma, partial [Bos taurus] (ABF57407.1)	ABF57407.1 [21]	50 kDa	2,5488	18,227	0,066196	0,20676	1	3	1	3	2,96%	8,66%	100%	100%
244,1	eukaryotic translation elongation factor 1 gamma, partial [Bos taurus]	ABF57407.1 (+20)	50 kDa	2,5488	18,22	0,066196	0,20676	1	3	1	3	2,96%	8,66%	100%	100%
245	malate dehydrogenase, cytoplasmic isoform MDH1 [Bos taurus]	NP_001029800.1 (+3)	36 kDa	0	17,012	0	0,53617	0	5	0	5	0	29,60%	0	100%
246	Cluster of PREDICTED: procollagen-lysine,2-oxoglutarate 5-dioxygenase 1 [Macaca fascicularis] (XP_00554482	XP_005544827.1 [5]	96 kDa	5,9471	19,442	0,034403	0,068292	1	2	1	2	1,89%	3,19%	99%	100%
246,1	PREDICTED: procollagen-lysine,2-oxoglutarate 5-dioxygenase 1 [Macaca fascicularis]	XP_005544827.1 (+4)	96 kDa	5,9471	19,37	0,034403	0,068292	1	2	1	2	1,89%	3,19%	99%	100%
247	Cluster of 6-phosphogluconate dehydrogenase, decarboxylating [Bos taurus] (NP_001137210.1)	NP_001137210.1 [5]	53 kDa	0,84959	17,012	0,062549	0,5152	1	7	1	7	3,52%	20,90%	97%	100%
247,1	6-phosphogluconate dehydrogenase, decarboxylating [Bos taurus]	NP_001137210.1 (+4)	53 kDa	0,84851	17,002	0,062549	0,5152	1	7	1	7	3,52%	20,90%	96%	100%
248	Cluster of TPA: mannose receptor, C type 2 [Bos taurus] (DAA18323.1)	DAA18323.1 [11]	167 kDa	16,992	0	0,039734	0	2	0	2	0	2,02%	0,00%	100%	72%
248,1	TPA: mannose receptor, C type 2 [Bos taurus]	DAA18323.1 (+10)	167 kDa	14,791	0	0,039734	0	2	0	2	0	2,02%	0,00%	100%	72%
249	Cluster of serpin A3-2 precursor [Bos taurus] (NP_001139773.1)	NP_001139773.1 [3]	46 kDa	1,7954	83,196	0	0,61029	1	5	1	7	2,19%	18,70%	29%	100%
249,1	serpin A3-2 precursor [Bos taurus]	NP_001139773.1 (+2)	46 kDa	1,761	81,998	0,072009	0,61029	1	5	1	7	2,19%	18,70%	29%	100%
250	alpha-2-macroglobulin receptor-associated protein precursor [Bos taurus]	NP_001073694.1 (+1)	42 kDa	7,6463	15,797	0,079367	0,45297	1	5	1	5	4,70%	13,80%	100%	100%
251	TPA: prostaglandin reductase 1 [Bos taurus]	DAA26494.1 (+1)	36 kDa	0	23,087	0	0,30062	0	2	0	3	0	11,20%	0	100%
252	transthyretin precursor [Ovis aries]	NP_001009800.1 (+2)	16 kDa	14,443	0	0,21686	0	1	0	1	0	8,84%	0	100%	0
253	PREDICTED: enhancer of rudimentary homolog [Bos indicus]	XP_019824677.1 (+4)	12 kDa	1,6992	0	0,28053	0	1	0	1	0	10,60%	0	100%	0
254	stomatin-like protein 2, mitochondrial [Bos taurus]	NP_001033157.1 (+3)	39 kDa	0	21,872	0	0,38137	0	4	0	4	0	19,10%	0	100%
255	PREDICTED: isocitrate dehydrogenase [NADP] cytoplasmic [Capra hircus]	XP_005676503.1 (+2)	47 kDa	0	21,872	0	0,30839	0	4	0	4	0	16,90%	0	100%
256	HMGB2 isoform 5, partial [Pan troglodytes]	PNI61900.1 (+6)	15 kDa	0	19,442	0	0,8018	0	3	0	3	0	23,90%	0	100%
257	inter-alpha-trypsin inhibitor heavy chain H4 precursor [Bos taurus]	NP_001015590.2 (+6)	102 kDa	2,5488	2,4302	0,032508	0,064471	1	2	1	2	2,29%	6,33%	96%	100%
258	Cluster of very long-chain specific acyl-CoA dehydrogenase, mitochondrial precursor [Bos taurus] (NP_776919	NP_776919.1 [8]	71 kDa	0	31,593	0	0,36883	0	6	0	8	0	14,40%	0	100%
258,1	very long-chain specific acyl-CoA dehydrogenase, mitochondrial precursor [Bos taurus]	NP_776919.1 (+7)	71 kDa	0	31,3	0	0,36883	0	5	0	7	0	14,40%	0	100%
259	Cluster of carboxymethylenebutenolidase homolog [Bos taurus] (NP_001179912.1)	NP_001179912.1 [7]	28 kDa	0	10,936	0	0,39468	0	2	0	3	0	11,40%	0	100%
259,1	carboxymethylenebutenolidase homolog [Bos taurus]	NP_001179912.1 (+6)	28 kDa	0	10,604	0	0,39468	0	2	0	3	0	11,40%	0	100%
260	Cluster of 40S ribosomal protein S27-like [Bos taurus] (NP_001035668.1)	NP_001035668.1 [99]	9 kDa	5,0975	0	0,62845	0	2	0	2	0	31,00%	0	100%	0
260,1	RPS27 [Bos taurus]	ABC84249.1 (+87)	9 kDa	0,75605	0	0,37657	0	1	0	1	0	15,50%	0	96%	0
260,2	40S ribosomal protein S27-like [Bos taurus]	NP_001035668.1 (+10)	9 kDa	4,2126	0	0,88033	0	2	0	2	0	31,00%	0	100%	0
261	Cluster of hypothetical protein CB1_029410002 [Camelus ferus] (EPY73167.1)	EPY73167.1	102 kDa	5,0975	0	0,032401	0	1	0	1	0	1,85%	0	100%	0
262	PREDICTED: thioredoxin-dependent peroxide reductase, mitochondrial [Condylura cristata]	XP_004680454.1	28 kDa	0	26,733	0	0,3929	0	3	0	3	0	14,40%	0	100%
263	Cluster of PREDICTED: phosphoribosyl pyrophosphate synthase-associated protein 1 isoform X1 [Monodelphis	XP_001368987.2 [11]	43 kDa	2,5488	0	0,16269	0	2	0	2	0	7,53%	0	100%	0
263,1	PREDICTED: phosphoribosyl pyrophosphate synthase-associated protein 1 isoform X1 [Monodelphis domes	XP_001368987.2 (+10)	43 kDa	2,5468	0	0,16269	0	2	0	2	0	7,53%	0	100%	0
264	TPA: FUS interacting protein (serine-arginine rich) 1-like [Bos taurus]	DAA18755.1 (+181)	31 kDa	3,3984	0	0,10736	0	1	0	1	0	6,11%	0	100%	0
265	hypothetical protein A6R68_18855 [Neotoma lepida]	OBS78770.1	13 kDa	2,5488	0	0,25627	0	1	0	1	0	9,24%	0	96%	0
266	ATP synthase subunit d, mitochondrial [Bos taurus]	NP_777149.1 (+2)	19 kDa	0	10,936	0	0,63502	0	3	0	3	0	26,70%	0	100%
267	importin subunit beta-1 [Bos taurus]	NP_001180082.1 (+46)	97 kDa	0	2,4302	0	0,03315	0	1	0	1	0	1,71%	0	100%
268	TPA: hypothetical protein LOC790886, partial [Bos taurus]	DAA21472.1 (+5)	15 kDa	8,4959	0	0,22511	0	1	0	1	0	9,16%	0	99%	0
269	Cluster of dihydropyrimidinase-like 2 [Sus scrofa] (AET99219.1)	AET99219.1 [8]	62 kDa	0	23,087	0	0,35537	0	5	0	6	0	14,00%	0	100%
269,1	dihydropyrimidinase-like 2 [Sus scrofa]	AET99219.1 (+7)	62 kDa	0	22,304	0	0,35537	0	5	0	6	0	14,00%	0	100%
270	Cluster of TPA: T-complex protein 1 subunit zeta [Bos taurus] (DAA15399.1)	DAA15399.1 [8]	54 kDa	0	21,872	0	0,12477	0	2	0	2	0	11,20%	0	100%
270,1	TPA: T-complex protein 1 subunit zeta [Bos taurus]	DAA15399.1 (+7)	54 kDa	0	19,328	0	0,12477	0	2	0	2	0	11,20%	0	100%
271	Cluster of ubiquitin-like modifier-activating enzyme 1 [Bos taurus] (NP_001095947.1)	NP_001095947.1 [3]	118 kDa	0	13,366	0	0,055363	0	2	0	2	0	2,74%	0	100%
271,1	ubiquitin-like modifier-activating enzyme 1 [Bos taurus]	NP_001095947.1 (+2)	118 kDa	0	13,327	0	0,055363	0	2	0	2	0	2,74%	0	100%
272	14-3-3 protein gamma [Mus musculus]	AAC14345.1 (+11)	28 kDa	0	27,423	0	1,1572	0	5	0	7	0	23,90%	0	100%
273	glucose-6-phosphate isomerase [Bos taurus]	NP_001035561.1 (+2)	63 kDa	0	29,163	0	0,28547	0	4	0	5	0	13,10%	0	100%
274	T-complex protein 1 subunit eta [Bos taurus]	NP_001039636.1 (+111)	59 kDa	0	31,593	0	0,17257	0	3	0	3	0	10,90%	0	100%
275	dolichyl-diphosphooligosaccharide--protein glycosyltransferase subunit DAD1 [Bos taurus]	NP_001029933.1 (+3)	12 kDa	8,4959	0	0,63208	0	2	0	2	0	17,70%	0	100%	0
276	Cluster of PSMA7 protein, partial [Bos taurus] (AAI51812.1)	AAI51812.1 [22]	27 kDa	0	24,302	0	0,59548	0	4	0	4	0	23,60%	0	100%
276,1	PSMA7 protein, partial [Bos taurus]	AAI51812.1 (+21)	27 kDa	0	24,298	0	0,59548	0	4	0	4	0	23,60%	0	100%
277	Cluster of PREDICTED: vinculin [Jaculus jaculus] (XP_004657881.1)	XP_004657881.1	120 kDa	0	6,0756	0	0,054268	0	2	0	2	0	2,18%	0	100%
277,1	PREDICTED: vinculin [Jaculus jaculus]	XP_004657881.1	120 kDa	0	5,0606	0	0,054268	0	2	0	2	0	2,18%	0	100%
278	Cluster of 40S ribosomal protein S14 isoform X1 [Bos taurus] (XP_024849610.1)	XP_024849610.1 [22]	17 kDa	0,84959	0	0,19933	0	1	0	1	0	13,30%	0	100%	0
279	TPA: EMI domain containing 1 [Bos taurus]	DAA20521.1 (+15)	48 kDa	11,045	0	0,069961	0	1	0	1	0	1,95%	0	100%	0
280	TPA: CKLF-like MARVEL transmembrane domain containing 6 [Bos taurus]	DAA17141.1 (+4)	20 kDa	16,142	0	0,16769	0	1	0	1	0	6,04%	0	100%	0
281	mannose-P-dolichol utilization defect 1 protein [Bos taurus]	NP_001068647.1 (+7)	27 kDa	4,2479	8,5059	0,1267	0,12366	1	1	1	1	4,07%	4,07%	96%	99%
282	HDCMA39P, partial [Homo sapiens]	AAF65181.1 (+40)	20 kDa	0	21,872	0	0,16459	0	1	0	1	0,00%	7,14%	78%	99%
283	Cluster of serpin A3-5 [Bos taurus] (XP_005222351.1)	XP_005222351.1 [2]	46 kDa	1,603	66,264	0	0,50271	1	6	1	7	2,18%	17,20%	25%	100%
283,1	serpin A3-5 [Bos taurus]	XP_005222351.1 (+1)	46 kDa	1,5818	65,455	0,071834	0,50271	1	5	1	6	2,18%	17,20%	25%	100%
284	Cluster of 40S ribosomal protein S25, partial [Bos mutus] (ELR55381.1)	ELR55381.1 [28]	14 kDa	6,7967	0	0,56122	0	2	0	2	0	15,20%	0	100%	0
284,1	40S ribosomal protein S25, partial [Bos mutus]	ELR55381.1 (+27)	14 kDa	6,7	0	0,56122	0	2	0	2	0	15,20%	0	100%	0
285	Cluster of TPA: ribosomal protein S24-like [Bos taurus] (DAA17032.1)	DAA17032.1 [71]	15 kDa	5,0975	0	0,49613	0	2	0	2	0	20,50%	0	100%	0
285,1	TPA: ribosomal protein S24-like [Bos taurus]	DAA17032.1 (+70)	15 kDa	5,0148	0	0,49613	0	2	0	2	0	20,50%	0	100%	0
286	very-long-chain 3-oxoacyl-CoA reductase [Bos taurus]	NP_001094777.1	35 kDa	0	12,151	0	0,30808	0	3	0	3	0	14,70%	0	100%
287	ADP-dependent glucokinase precursor [Bos taurus]	NP_001075907.1 (+1)	54 kDa	8,4959	8,5059	0,12648	0,12344	2	2	2	2	3,22%	7,44%	100%	100%
288	Cluster of PREDICTED: LOW QUALITY PROTEIN: T-complex protein 1 subunit theta isoform X1 [Bos indicus] (XI	XP_019813698.1 [2]	61 kDa	5,0975	18,227	0,054771	0,2318	1	4	1	4	2,70%	10,40%	100%	100%
288,1	PREDICTED: LOW QUALITY PROTEIN: T-complex protein 1 subunit theta isoform X1 [Bos indicus]	XP_019813698.1 (+1)	61 kDa	5,0975	17,99	0,054771	0,2318	1	4	1	4	2,70%	10,40%	100%	100%

289	Cluster of FDXR protein [Bos taurus] (AAI18191.1)	AAI18191.1 [7]	54 kDa	0	21,872	0	0,4165	0	5	0	6	0	12,20%	0	100%
289,1	FDXR protein [Bos taurus]	AAI18191.1 (+6)	54 kDa	0	21,871	0	0,4165	0	5	0	6	0	12,20%	0	100%
290	electron transfer flavoprotein subunit beta [Bos taurus]	NP_001033671.1 (+5)	28 kDa	0	10,936	0	0,5667	0	4	0	4	0	22,00%	0	100%
291	basigin precursor [Bos taurus]	NP_001068839.1 (+3)	30 kDa	4,2479	6,0756	0,11269	0,23219	1	2	1	2	6,64%	12,50%	99%	100%
292	vacuolar adenosine triphosphatase subunit D [Mus musculus]	AAC83085.1 (+59)	40 kDa	1,6992	0	0,082702	0	1	0	1	0	3,13%	0	100%	0
293	retinol-binding protein, partial [Bos taurus]	AAB28336.1 (+13)	15 kDa	9,3455	7,2907	0,22511	0,2197	1	1	1	1	8,33%	8,33%	99%	99%
294	Profilin-2, partial [Bos mutus]	ELR48672.1 (+107)	16 kDa	15,293	0	0,21373	0	1	0	1	0	9,72%	0	99%	0
295	collagen alpha-1(VI) chain precursor [Bos taurus]	NP_001137337.1 (+47)	109 kDa	0	6,0756	0	0,029637	0	1	0	1	0,00%	1,56%	45%	98%
296	ubiquitin carboxyl-terminal hydrolase isozyme L1 [Bos taurus]	NP_001039637.1 (+7)	28 kDa	0	8,5059	0	0,24498	0	2	0	2	0	13,50%	0	100%
297	enoyl-CoA hydratase precursor, partial [Bos taurus]	AAL09921.1 (+7)	28 kDa	0	21,34	0	0,39114	0	3	0	3	0	19,30%	0	100%
298	endopin 2B [Bos taurus]	AAR26722.1 (+2)	47 kDa	0	13,366	0	0,39731	0	4	0	5	0	15,10%	0	100%
299	fructose-bisphosphate aldolase C [Bos taurus]	NP_001091453.1 (+25)	39 kDa	0	23,535	0	0,61115	0	6	0	6	0	25,80%	0	100%
300	SMN protein, partial [Bos taurus]	AAI48904.1 (+10)	33 kDa	20,39	0	0,10181	0	1	0	1	0	6,98%	0	99%	0
301	F1-ATPase beta subunit=H(+)-transporting ATPase beta subunit {EC 3.6.1.34} [cattle, heart, Peptide Mitochon	AAB20007.1	2 kDa	0	21,872	0	10,227	0	1	0	2	0	100,00%	0	99%
302	Cluster of T-complex protein 1 subunit beta [Bos taurus] (NP_001029411.1)	NP_001029411.1 [21]	57 kDa	0	12,151	0	0,24559	0	4	0	4	0	10,80%	0	100%
302,1	T-complex protein 1 subunit beta [Bos taurus]	NP_001029411.1 (+20)	57 kDa	0	12,138	0	0,24559	0	4	0	4	0	10,80%	0	100%
303	Cluster of TPA: ferritin light chain-like [Bos taurus] (DAA17430.1)	DAA17430.1 [12]	20 kDa	13,593	0	0,17158	0	1	0	1	0	8,57%	0	100%	0
304	proteasome subunit beta type-1 precursor [Bos taurus]	NP_001033628.1 (+62)	26 kDa	0	15,797	0	0,42547	0	3	0	3	0	13,70%	0	100%
305	beta 2 glycoprotein I [cattle, Peptide, 326 aa]	AAB20668.1	36 kDa	0	14,581	0	0,18769	0	2	0	2	0	10,40%	0	100%
306	PREDICTED: ras-related protein Rab-14 isoform X1 [Ursus maritimus]	XP_008697044.1	25 kDa	8,4959	7,2907	0,1373	0,28618	1	2	1	2	13,60%	14,00%	100%	100%
307	gelsolin isoform b [Bos taurus]	NP_001029799.1 (+167)	81 kDa	0	4,8605	0	0,081531	0	2	0	2	0,00%	4,38%	47%	100%
308	PREDICTED: serglycin [Bison bison bison]	XP_010850505.1	17 kDa	7,6463	0	0,20485	0	1	0	1	0	14,70%	0	99%	0
309	Cluster of heterogeneous nuclear ribonucleoprotein K [Bos taurus] (NP_001029734.1)	NP_001029734.1 [20]	51 kDa	0	9,721	0	0,3617	0	4	0	5	0	12,50%	0	100%
309,1	heterogeneous nuclear ribonucleoprotein K [Bos taurus]	NP_001029734.1 (+19)	51 kDa	0	9,7172	0	0,3617	0	4	0	5	0	12,50%	0	100%
310	Cluster of transaldolase [Bos taurus] (NP_001030360.2)	NP_001030360.2 [5]	38 kDa	0	9,721	0	0,39431	0	4	0	4	0	11,90%	0	100%
310,1	transaldolase [Bos taurus]	NP_001030360.2 (+4)	38 kDa	0	9,302	0	0,39431	0	4	0	4	0	11,90%	0	100%
311	Cluster of TPA: ADP-ribosylation factor 3 [Bos taurus] (DAA29912.1)	DAA29912.1 [174]	16 kDa	8,4959	0	0,47636	0	2	0	2	0	22,10%	0	100%	0
311,1	TPA: ADP-ribosylation factor 3 [Bos taurus]	DAA29912.1 (+173)	16 kDa	7,4629	0	0,47636	0	2	0	2	0	22,10%	0	100%	0
312	complement component C8 gamma chain isoform X1 [Bos taurus]	XP_005213573.2 (+1)	22 kDa	5,9471	3,6454	0,3236	0,14698	2	1	2	1	13,90%	7,43%	100%	100%
313	Cluster of myosin-10 [Bos taurus] (NP_777259.1)	NP_777259.1 [341]	229 kDa	5,0975	0	0,014347	0	1	0	1	0	0,71%	0	100%	0
314	Rho GDP-dissociation inhibitor 1 [Fukomys damarensis]	KFO35713.1	48 kDa	0	4,8605	0	0,068117	0	1	0	1	0	5,83%	0	100%
315	proteasome (prosome, macropain) subunit, alpha type, 5 [Homo sapiens]	AAV38522.1 (+26)	26 kDa	0	4,8605	0	0,26399	0	2	0	2	0,00%	13,30%	30%	100%
316	complement component C9 precursor [Bos taurus]	NP_001030441.1 (+2)	62 kDa	4,2479	0	0,053481	0	1	0	1	0	2,19%	0,00%	100%	38%
317	Cluster of protein/nucleic acid deglycase DJ-1 [Bos taurus] (NP_001015572.1)	NP_001015572.1 [3]	20 kDa	0	9,721	0	0,8479	0	4	0	4	0	34,90%	0	100%
317,1	protein/nucleic acid deglycase DJ-1 [Bos taurus]	NP_001015572.1 (+2)	20 kDa	0	8,5679	0	0,8479	0	4	0	4	0	34,90%	0	100%
318	NADH dehydrogenase (ubiquinone) 1 alpha subcomplex, 9, 39kDa, partial [Bos taurus]	ABF57357.1 (+17)	42 kDa	0	12,151	0	0,16235	0	2	0	2	0	8,67%	0	100%
319	14-3-3 protein beta/alpha [Myotis brandtii]	EPQ10764.1	34 kDa	0	15,505	0	0,57854	0	3	0	5	0	14,80%	0	100%
320	probable glutathione peroxidase 8 [Bos taurus]	NP_001039553.1 (+3)	24 kDa	0,84959	3,6454	0,14057	0,13719	1	1	1	1	5,26%	5,26%	100%	100%
321	Cluster of apolipoprotein A-IV precursor [Bos taurus] (NP_001032557.1)	NP_001032557.1 [9]	43 kDa	3,3984	4,8605	0,077492	0,24469	1	3	1	3	3,16%	8,68%	95%	100%
321,1	apolipoprotein A-IV precursor [Bos taurus]	NP_001032557.1 (+8)	43 kDa	3,3984	4,792	0,077492	0,24469	1	3	1	3	3,16%	8,68%	95%	100%
322	Cluster of proteasome subunit beta type-5 [Bos taurus] (NP_001032701.1)	NP_001032701.1 [7]	29 kDa	0	10,936	0	0,54305	0	4	0	4	0	14,10%	0	100%
322,1	proteasome subunit beta type-5 [Bos taurus]	NP_001032701.1 (+6)	29 kDa	0	10,528	0	0,54305	0	4	0	4	0	14,10%	0	100%
323	PREDICTED: hydroxyacyl-coenzyme A dehydrogenase, mitochondrial [Bison bison bison]	XP_010856864.1	34 kDa	0	10,936	0	0,19866	0	2	0	2	0	6,69%	0	100%
324	tolloid-like protein 2 isoform X1 [Bos taurus]	XP_002698454.2 (+70)	114 kDa	1,6992	0	0,058412	0	2	0	2	0	2,16%	0	100%	0
325	proteolipid protein 2 [Bos taurus]	NP_976239.1 (+49)	17 kDa	5,9471	0	0,20485	0	1	0	1	0	8,55%	0	99%	0
326	Cluster of cathepsin B, partial [Bos taurus] (AAA30434.1)	AAA30434.1 [21]	14 kDa	0	19,442	0	0,3694	0	3	0	3	0	29,50%	0	100%
326,1	Cathepsin B [Macaca mulatta]	EHH28274.1 (+10)	38 kDa	0	8,4498	0	0,18064	0	2	0	2	0	7,67%	0	99%
326,2	cathepsin B, partial [Bos taurus]	AAA30434.1 (+9)	14 kDa	0	10,992	0	0,55817	0	2	0	2	0	29,50%	0	100%
327	Cluster of staphylococcal nuclease domain-containing protein 1 [Bos taurus] (NP_991353.1)	NP_991353.1 [85]	102 kDa	0	10,936	0	0,064184	0	2	0	2	0	3,19%	0	100%
327,1	staphylococcal nuclease domain-containing protein 1 [Bos taurus]	NP_991353.1 (+84)	102 kDa	0	10,916	0	0,064184	0	2	0	2	0	3,19%	0	100%
328	phosphoribosylaminoimidazole carboxylase [Bos taurus]	AAR05660.1 (+29)	47 kDa	0	12,151	0	0,14232	0	2	0	2	0	11,50%	0	100%
329	Ras-related protein Rap-1b, partial [Bos mutus]	ELR46625.1 (+92)	21 kDa	0	13,366	0	0,55144	0	2	0	3	0	7,53%	0	100%
330	eukaryotic translation initiation factor 3 subunit B [Bos taurus]	NP_001095826.1 (+36)	89 kDa	1,6992	0	0,03713	0	1	0	1	0	1,65%	0	100%	0
331	eukaryotic translation initiation factor 3 subunit C [Bos taurus]	NP_001029790.1 (+130)	105 kDa	6,7967	0	0,031301	0	1	0	1	0	1,32%	0	100%	0
332	Eukaryotic translation initiation factor 3 subunit I [Tupaia chinensis]	ELW48627.1	52 kDa	0,84959	0	0,064181	0	1	0	1	0	2,35%	0	100%	0
333	60S ribosomal protein L38 [Bos taurus]	NP_001106780.1 (+52)	8 kDa	1,6992	0	0,43108	0	1	0	1	0	14,30%	0	98%	0
334	anti-idiotype Ig lambda chain V region, partial [Bos taurus]	AAA68997.1	12 kDa	0,84959	0	0,28594	0	1	0	1	0	17,60%	0,00%	96%	42%
335	Cluster of polypyrimidine tract-binding protein 1 isoform 1 [Bos taurus] (NP_001289713.1)	NP_001289713.1 [3]	60 kDa	0	12,151	0	0,23606	0	3	0	4	0	10,80%	0	100%
335,1	polypyrimidine tract-binding protein 1 isoform 1 [Bos taurus]	NP_001289713.1 (+2)	60 kDa	0	8,5934	0	0,23606	0	3	0	4	0	10,80%	0	100%
336	3 beta-hydroxysteroid dehydrogenase/Delta 5-->4-isomerase [Bos taurus]	NP_776768.1 (+1)	42 kDa	0	10,936	0	0,24962	0	2	0	3	0,00%	9,38%	81%	100%
337	proteasome subunit alpha type 6, partial [Lynx pardinus]	AIA59720.1 (+48)	26 kDa	0	15,797	0	0,27142	0	2	0	2	0,00%	13,00%	77%	100%
338	ras-related protein Rab-2A [Bos taurus]	NP_001068822.1 (+3)	21 kDa	4,2479	3,6454	0,16217	0,15827	1	1	1	1	13,80%	13,80%	99%	100%
339	tissue alpha-L-fucosidase precursor [Bos taurus]	NP_001039500.1 (+2)	54 kDa	0	3,6454	0	0,059902	0	1	0	1	0	3,63%	0	100%
340	transmembrane emp24 domain-containing protein 7 precursor [Bos taurus]	NP_001098966.1 (+114)	25 kDa	1,6992	2,4302	0,13298	0,12978	1	1	1	1	4,44%	4,44%	95%	100%
341	TPA: histidine-rich glycoprotein [Bos taurus]	DAA33337.1 (+6)	61 kDa	1,6992	0	0,054569	0	1	0	1	0	2,05%	0	97%	0
342	immunoglobulin lambda light chain, partial [Macaca mulatta]	AER46313.1 (+1)	13 kDa	0,84959	1,2151	0,27532	0,2687	1	1	1	1	11,50%	11,50%	88%	96%
343	Cluster of 14-3-3 protein eta [Bos taurus] (NP_776917.2)	NP_776917.2 [16]	28 kDa	0	11,795	0	0,73457	0	3	0	5	0	15,40%	0	100%
343,1	14-3-3 protein eta [Bos taurus]	NP_776917.2 (+15)	28 kDa	0	8,8869	0	0,73457	0	3	0	5	0	15,40%	0	100%
344	syntaxin-6 [Bos taurus]	NP_001075900.1 (+117)	29 kDa	8,4959	0	0,38689	0	3	0	3	0	15,30%	0	100%	0
345	serine/arginine-rich splicing factor 7 [Bos taurus]	NP_001029449.1 (+117)	27 kDa	5,9471	0	0,26446	0	2	0	2	0	11,10%	0	100%	0
346	talin-1 [Bos taurus]	NP_001192357.3 (+15)	270 kDa	0	10,936	0	0,023916	0	2	0	2	0	0,98%	0	100%
347	syntaxin-7 [Bos taurus]	NP_001071332.1 (+5)	30 kDa	3,3984	0	0,11312	0	1	0	1	0	5,75%	0	100%	0
348	Cluster of endoplasmic reticulum resident protein 44 precursor [Bos taurus] (NP_001030204.1)	NP_001030204.1 [6]	47 kDa	0,84959	10,936	0	0,14338	1	2	1	2	3,45%	9,11%	100%	100%
348,1	endoplasmic reticulum resident protein 44 precursor [Bos taurus]	NP_001030204.1 (+5)	47 kDa	0,4224	8,4922	0,070971	0,14338	1	2	1	2	3,45%	9,11%	65%	100%
349	cytoplasmic dynein 1 intermediate chain 2 isoform X1 [Desmodus rotundus]	XP_024417285.1 (+6)	72 kDa	5,9471	1,2151	0,04627	0,045157	1	1	1	1	3,29%	3,29%	99%	99%
350	PREDICTED: Niemann-Pick C1 protein [Bos indicus]	XP_019842271.1	142 kDa	0	13,366	0	0,022663	0	1	0	1	0	0,94%	0	99%
351	Myosin light polypeptide 6 [Tupaia chinensis]	ELW72266.1	29 kDa	7,6463	0	0,11399	0	1	0	1	0	5,02%	0	97%	0
352	PREDICTED: LOW QUALITY PROTEIN: C4b-binding protein alpha chain-like isoform X1 [Ovis aries]	XP_014954512.1 (+1)	94 kDa	0,84959	6,0756	0,035015	0,034173	1	1	1	1	1,18%	1,18%	93%	97%
353	mitochondrial import receptor subunit TOM40 homolog [Bos taurus]	NP_001068906.1 (+5)	38 kDa	0	9,721	0	0,28207	0	3	0	3	0	8,03%	0	100%

354	chitinase domain-containing protein 1 precursor [Bos taurus]	NP_001015515.1 (+3)	45 kDa	0	1,2151	0	0,072947	0	1	0	1	0,00%	4,33%	65%	100%
355	immunoglobulin superfamily member 8 precursor [Bos taurus]	NP_001075908.1 (+2)	64 kDa	1,6992	0	0,051431	0	1	0	1	0	3,93%	0	100%	0
356	endonuclease G, mitochondrial precursor [Bos taurus]	NP_787017.1 (+3)	32 kDa	0	4,8605	0	0,10146	0	1	0	1	0	9,03%	0	100%
357	PREDICTED: inhibin alpha chain [Bos indicus]	XP_019833064.1 (+9)	39 kDa	0	1,2151	0	0,084008	0	1	0	1	0	4,17%	0	100%
358	nodal modulator 1 [Bos taurus]	XP_002698082.1 (+28)	134 kDa	0	4,8605	0	0,023984	0	1	0	1	0	0,82%	0	99%
359	Sfrs10 protein [Mus musculus]	AAH86795.1 (+56)	27 kDa	0,84959	0	0,12616	0	1	0	1	0	3,93%	0	98%	0
360	Cluster of chloride intracellular channel protein 1 [Bos taurus] (NP_001015608.1)	NP_001015608.1	27 kDa	0	7,2907	0	0,41143	0	3	0	3	0	13,30%	0	100%
360,1	chloride intracellular channel protein 1 [Bos taurus]	NP_001015608.1	27 kDa	0	7,1978	0	0,41143	0	3	0	3	0	13,30%	0	100%
361	glutathione S-transferase Mu 1 [Bos taurus]	NP_787019.1 (+2)	26 kDa	0	14,518	0	0,62067	0	4	0	4	0	17,90%	0	100%
362	heme-binding protein 1 [Bos taurus]	NP_001069453.1 (+7)	21 kDa	0	6,0756	0	0,33595	0	2	0	2	0	15,20%	0	100%
363	60S ribosomal protein L23 [Bos taurus]	NP_001030186.1 (+27)	15 kDa	3,3984	0	0,51759	0	2	0	2	0	27,10%	0	100%	0
364	TPA: sortilin 1 [Bos taurus]	DAA31548.1 (+20)	101 kDa	0,84959	0	0,032616	0	1	0	1	0	1,63%	0	100%	0
365	HABP2 [Cervus elaphus hippelaphus]	OWK07723.1	70 kDa	1,6992	0	0,047082	0	1	0	1	0	2,72%	0	98%	0
366	protein RER1 [Bos taurus]	NP_001098962.1 (+39)	23 kDa	7,6463	0	0,14759	0	1	0	1	0	9,69%	0	98%	0
367	PREDICTED: CAD protein-like [Pantholops hodgsonii]	XP_005960892.1	36 kDa	0,84959	0	0,093138	0	1	0	1	0	2,72%	0	96%	0
368	Cluster of TPA: UDP-Gal:betaGlcNAc beta 1,4- galactosyltransferase, polypeptide 4 [Bos taurus] (DAA33498.1)	DAA33498.1 [8]	40 kDa	3,3984	0	0,17522	0	2	0	2	0	6,14%	0,00%	100%	80%
368,1	TPA: UDP-Gal:betaGlcNAc beta 1,4- galactosyltransferase, polypeptide 4 [Bos taurus]	DAA33498.1 (+7)	40 kDa	2,94	0	0,17522	0	2	0	2	0	6,14%	0,00%	98%	54%
369	Cluster of isocitrate dehydrogenase [NADP], mitochondrial precursor [Bos taurus] (NP_786984.1)	NP_786984.1 [28]	51 kDa	0	7,2907	0	0,13161	0	2	0	2	0	6,64%	0	100%
369,1	isocitrate dehydrogenase [NADP], mitochondrial precursor [Bos taurus]	NP_786984.1 (+27)	51 kDa	0	7,2759	0	0,13161	0	2	0	2	0	6,64%	0	100%
370	Cluster of TPA: 3,2-trans-enoyl-CoA isomerase, mitochondrial [Bos taurus] (DAA15604.1)	DAA15604.1 [2]	33 kDa	0	13,366	0	0,32537	0	3	0	3	0	17,20%	0	100%
370,1	TPA: 3,2-trans-enoyl-CoA isomerase, mitochondrial [Bos taurus]	DAA15604.1 (+1)	33 kDa	0	13,361	0	0,32537	0	3	0	3	0	17,20%	0	100%
371	Cluster of Enhancer of mRNA-decapping protein 4 [Bos mutus] (ELR55807.1)	ELR55807.1 [84]	153 kDa	9,3455	0	0,043513	0	2	0	2	0	2,97%	0	100%	0
371,1	Enhancer of mRNA-decapping protein 4 [Bos mutus]	ELR55807.1 (+83)	153 kDa	9,3023	0	0,043513	0	2	0	2	0	2,97%	0	100%	0
372	Cluster of proteasome subunit beta type-2 [Bos taurus] (NP_001015615.1)	NP_001015615.1 [2]	23 kDa	0	2,4302	0	0,14404	0	1	0	1	0	5,47%	0	100%
373	medium-chain specific acyl-CoA dehydrogenase, mitochondrial precursor [Bos taurus]	NP_001068703.1 (+2)	47 kDa	0	3,6454	0	0,14446	0	2	0	2	0	10,20%	0	100%
374	trifunctional enzyme subunit beta, mitochondrial precursor [Bos taurus]	NP_776761.1 (+16)	51 kDa	0	3,6454	0	0,20194	0	3	0	3	0	8,21%	0	100%
375	saccharopine dehydrogenase-like oxidoreductase [Bos taurus]	NP_001029460.1 (+5)	47 kDa	0	10,936	0	0,14197	0	2	0	2	0	13,10%	0	100%
376	Proteasome subunit alpha type-3, partial [Bos mutus]	ELR52732.1	28 kDa	0	8,5059	0	0,56398	0	3	0	4	0	18,50%	0	100%
377	dihydrolipoyl dehydrogenase, mitochondrial [Bos taurus]	NP_001193099.1 (+50)	54 kDa	0	6,0756	0	0,12317	0	2	0	2	0	4,32%	0	100%
378	proteasome assembly chaperone 1 [Bos taurus]	NP_001069815.1	33 kDa	0	13,366	0	0,20796	0	2	0	2	0	10,40%	0	100%
379	26S proteasome regulatory subunit 10B [Bos taurus]	NP_001039705.1 (+31)	44 kDa	0	2,4302	0	0,073693	0	1	0	1	0,00%	3,60%	50%	100%
380	ORM1-like protein 3 [Bos taurus]	NP_001069835.1 (+29)	17 kDa	8,4959	0	0,1941	0	1	0	1	0	9,80%	0	99%	0
381	ORM1-like protein 1 [Bos taurus]	NP_001070601.1 (+107)	17 kDa	9,3455	0	0,19668	0	1	0	1	0	9,80%	0	99%	0
382	Cluster of hypoxia up-regulated protein 1 precursor [Bos taurus] (NP_001193839.1)	NP_001193839.1 [10]	112 kDa	0	1,2151	0	0,028835	0	1	0	1	0	1,20%	0	100%
383	proteasome activator complex subunit 1 [Bos taurus]	NP_001019680.2 (+57)	29 kDa	0	10,936	0	0,24191	0	2	0	2	0	10,40%	0	100%
384	secretory carrier-associated membrane protein 3 [Bos taurus]	NP_001030503.1 (+102)	38 kDa	3,3984	0	0,18168	0	2	0	2	0	9,80%	0	100%	0
385	mitochondrial dicarboxylate carrier [Bos taurus]	XP_003587518.1 (+8)	32 kDa	0	1,2151	0	0,10365	0	1	0	1	0	3,48%	0	100%
386	adenosine kinase [Bos taurus]	NP_001069534.1 (+5)	39 kDa	0	6,0756	0	0,0845	0	1	0	1	0	4,64%	0	100%
387	myosin regulatory light polypeptide 9 [Bos taurus]	NP_001015640.1 (+116)	20 kDa	7,6463	0	0,17059	0	1	0	1	0	5,81%	0	99%	0
388	Golgi membrane protein 1 [Bos taurus]	NP_001179392.1 (+2)	46 kDa	8,4959	0	0,073079	0	1	0	1	0	5,68%	0	99%	0
389	PREDICTED: protein kish-A [Bos indicus]	XP_019825036.1 (+68)	7 kDa	7,6463	0	0,53061	0	1	0	1	0	15,00%	0	99%	0
390	Cluster of PREDICTED: guanine nucleotide-binding protein subunit alpha-11 [Erinaceus europaeus] (XP_00752	XP_007524874.1	41 kDa	0,8231	5,962	0	0,16327	1	2	1	2	3,06%	8,91%	96%	100%
390,1	PREDICTED: guanine nucleotide-binding protein subunit alpha-11 [Erinaceus europaeus]	XP_007524874.1	41 kDa	0,3437	3,905	0,080448	0,16327	1	2	1	2	3,06%	8,91%	20%	98%
391	TPA: CD9 antigen, partial [Bos taurus]	DAA29220.1 (+11)	23 kDa	0,84959	0	0,14908	0	1	0	1	0	4,90%	0,00%	97%	24%
392	Cluster of aldehyde dehydrogenase, mitochondrial precursor [Bos taurus] (NP_001068835.1)	NP_001068835.1 [21]	57 kDa	0	6,0756	0	0,11764	0	2	0	2	0,00%	5,77%	10%	100%
392,1	aldehyde dehydrogenase, mitochondrial precursor [Bos taurus]	NP_001068835.1 (+20)	57 kDa	0	5,6655	0	0,11764	0	2	0	2	0	5,77%	0	100%
393	Cluster of TPA: NADH dehydrogenase [ubiquinone] iron-sulfur protein 3, mitochondrial precursor [Bos taurus]	DAA21757.1 [9]	29 kDa	0	8,5059	0	0,38425	0	3	0	3	0	17,90%	0	100%
393,1	TPA: NADH dehydrogenase [ubiquinone] iron-sulfur protein 3, mitochondrial precursor [Bos taurus]	DAA21757.1 (+8)	29 kDa	0	8,3758	0	0,38425	0	3	0	3	0	17,90%	0	100%
394	Cluster of adenylyl cyclase-associated protein 1 isoform X2 [Bos taurus] (XP_005204713.1)	XP_005204713.1 [2]	51 kDa	0	4,8605	0	0,20241	0	3	0	3	0	12,70%	0	100%
394,1	adenylyl cyclase-associated protein 1 isoform X2 [Bos taurus]	XP_005204713.1 (+1)	51 kDa	0	4,7656	0	0,20241	0	3	0	3	0	12,70%	0	100%
395	Cluster of glutathione peroxidase 7 precursor [Bos taurus] (NP_001094583.1)	NP_001094583.1 [3]	21 kDa	0	6,0756	0	0,34189	0	2	0	2	0	19,40%	0	100%
395,1	glutathione peroxidase 7 precursor [Bos taurus]	NP_001094583.1 (+2)	21 kDa	0	6,0414	0	0,34189	0	2	0	2	0	19,40%	0	100%
396	Cluster of NADH dehydrogenase [ubiquinone] 1 alpha subcomplex subunit 10, mitochondrial precursor [Bos ta	NP_788828.1 [11]	39 kDa	0	4,8605	0	0,17254	0	2	0	2	0	10,20%	0	100%
396,1	NADH dehydrogenase [ubiquinone] 1 alpha subcomplex subunit 10, mitochondrial precursor [Bos taurus]	NP_788828.1 (+10)	39 kDa	0	4,5841	0	0,17254	0	2	0	2	0	10,20%	0	100%
397	TPA: glutathione S-transferase mu 3, partial [Bos taurus]	DAA31493.1 (+5)	25 kDa	0	22,078	0	1,1037	0	5	0	6	0	27,80%	0	100%
398	ras-related protein Rab-6A [Bos taurus]	NP_001180044.1 (+30)	24 kDa	0	3,6454	0	0,2995	0	2	0	2	0,00%	12,50%	9%	100%
399	integrin alpha-V subunit, partial [Bos taurus]	AAG38595.1 (+18)	111 kDa	4,2479	0	0,029576	0	1	0	1	0	2,18%	0,00%	100%	100%
400	macrophage migration inhibitory factor, partial [Sus scrofa]	AAD50507.1 (+30)	12 kDa	5,0975	0	0,28872	0	1	0	1	0	9,91%	0	100%	0
401	inteferon-induced membrane protein Leu-13/9-27 [Bos taurus]	AAK58639.1 (+31)	14 kDa	10,195	0	0,24565	0	1	0	1	0	12,80%	0	99%	0
402	protein LYRIC [Bos taurus]	NP_001039503.1 (+12)	64 kDa	6,7967	0	0,051701	0	1	0	1	0	3,60%	0	99%	0
403	unnamed protein product [Homo sapiens]	CAA68390.1 (+13)	11 kDa	5,9471	2,4302	0,31984	0,31215	1	1	1	1	11,70%	11,70%	99%	99%
404	Cluster of PREDICTED: T-complex protein 1 subunit delta isoform X1 [Bos mutus] (XP_014331863.1)	XP_014331863.1 [5]	59 kDa	0	8,5059	0	0,23998	0	4	0	4	0	11,50%	0	100%
404,1	PREDICTED: T-complex protein 1 subunit delta isoform X1 [Bos mutus]	XP_014331863.1 (+4)	59 kDa	0	8,4367	0	0,23998	0	4	0	4	0	11,50%	0	100%
405	Cluster of dihydrolipoyllysine-residue succinyltransferase component of 2-oxoglutarate dehydrogenase compl	NP_001068750.1 [6]	49 kDa	0	6,0756	0	0,13692	0	2	0	2	0	6,59%	0	100%
405,1	dihydrolipoyllysine-residue succinyltransferase component of 2-oxoglutarate dehydrogenase complex, mito	NP_001068750.1 (+5)	49 kDa	0	6,0732	0	0,13692	0	2	0	2	0	6,59%	0	100%
406	Cluster of 3-hydroxyisobutyrate dehydrogenase, mitochondrial precursor [Bos taurus] (NP_001039571.1)	NP_001039571.1 [28]	35 kDa	0	6,0756	0	0,1927	0	2	0	2	0	8,04%	0	100%
406,1	3-hydroxyisobutyrate dehydrogenase, mitochondrial precursor [Bos taurus]	NP_001039571.1 (+27)	35 kDa	0	4,393	0	0,1927	0	2	0	2	0	8,04%	0	100%
407	Cluster of lupus La protein homolog [Bos taurus] (NP_788838.1)	NP_788838.1 [42]	47 kDa	0	6,0756	0	0,14446	0	2	0	2	0	6,19%	0	100%
407,1	lupus La protein homolog [Bos taurus]	NP_788838.1 (+41)	47 kDa	0	5,4412	0	0,14446	0	2	0	2	0	6,19%	0	99%
408	calcyclin-binding protein [Bos taurus]	NP_001029981.1 (+2)	26 kDa	0	8,5059	0	0,12473	0	1	0	1	0	8,70%	0	100%
409	actin, cytoplasmic 2-like [Aotus nancymaae]	XP_012301888.1	42 kDa	0	8,9918	0	0,25034	0	3	0	3	0	9,07%	0	100%
410	GTP-binding protein SAR1a [Myotis davidii]	ELK32383.1	53 kDa	0	4,8605	0	0,061304	0	1	0	1	0	2,32%	0	100%
411	ATPase, H+ transporting, lysosomal 31kDa, V1 subunit E1 [Bos taurus]	AAI02617.1 (+95)	26 kDa	8,4959	1,2151	0,12892	0,12582	1	1	1	1	7,08%	7,08%	99%	99%
412	small GTPase, partial [Sus scrofa]	AAS55918.1 (+6)	3 kDa	0	10,936	0	1,3028	0	1	0	1	0	42,90%	0	99%
413	transmembrane protein 14C [Bos taurus]	NP_001030514.1 (+3)	12 kDa	1,6992	0	0,29445	0	1	0	1	0	9,65%	0	98%	0
414	LOW QUALITY PROTEIN: heat shock 70 kDa protein 6 [Papio anubis]	XP_021781830.1	71 kDa	0,84959	4,8605	0,046709	0,045586	1	1	1	1	2,50%	2,50%	98%	98%
415	matrix Gla protein precursor [Bos taurus]	NP_777132.1	12 kDa	4,2479	0	0,28321	0	1	0	1	0	10,70%	0	97%	0
416	Cluster of voltage-dependent anion-selective channel protein 3 [Bos taurus] (NP_777154.1)	NP_777154.1 [32]	31 kDa	0	12,969	0	0,49901	0	4	0	4	0,00%	15,20%	16%	100%
416,1	voltage-dependent anion-selective channel protein 3 [Bos taurus]	NP_777154.1 (+31)	31 kDa	0	12,744	0	0,49901	0	4	0	4	0,00%	15,20%	16%	100%

417	Cluster of ras-related protein Rab-11A [Bos taurus] (NP_001033251.1)	NP_001033251.1 [47]	24 kDa	0	4,8605	0	0,2876	0	2	0	2	0,00%	11,10%	5%	100%
417,1	ras-related protein Rab-11A [Bos taurus]	NP_001033251.1 (+46)	24 kDa	0	4,1484	0	0,2876	0	2	0	2	0	11,10%	0	100%
418	Cluster of alcohol dehydrogenase [NADP(+)] [Bos taurus] (NP_001069981.1)	NP_001069981.1 [8]	37 kDa	0	5,5931	0	0,18587	0	2	0	2	0	5,54%	0	100%
418,1	alcohol dehydrogenase [NADP(+)] [Bos taurus]	NP_001069981.1 (+7)	37 kDa	0	5,2814	0	0,18587	0	2	0	2	0	5,54%	0	100%
419	UQCRC1 protein, partial [Bos taurus]	AAI04501.1 (+8)	53 kDa	0	8,5059	0	0,27089	0	3	0	4	0	6,69%	0	100%
420	Ras-related protein Rab-1A, partial [Bos mutus]	ELR57927.1 (+42)	27 kDa	0	6,0756	0	0,26279	0	2	0	2	0,00%	10,20%	9%	100%
421	proteasome 26S non-ATPase subunit 7, partial [Bos taurus]	ABM21562.1 (+66)	36 kDa	0	9,721	0	0,30062	0	2	0	3	0	9,27%	0	100%
422	adenine phosphoribosyltransferase [Bos taurus]	NP_001020505.1	20 kDa	0	2,4302	0	0,36789	0	2	0	2	0	14,40%	0	100%
423	succinate--CoA ligase [ADP/GDP-forming] subunit alpha, mitochondrial precursor [Bos taurus]	NP_001030254.1 (+19)	36 kDa	0	3,6454	0	0,090046	0	1	0	1	0	4,62%	0	100%
424	TPA: FK506 binding protein 11 precursor [Bos taurus]	DAA29904.1 (+13)	22 kDa	1,6992	3,6454	0,15137	0,14773	1	1	1	1	8,91%	8,91%	98%	100%
425	transmembrane protein 35A [Bos taurus]	NP_001014940.1 (+2)	19 kDa	3,3984	0	0,17671	0	1	0	1	0	16,90%	0	99%	0
426	manganous superoxide dismutase, partial [Bos taurus]	AAA30655.1 (+15)	26 kDa	0	7,2907	0	0,12862	0	1	0	1	0	6,06%	0	99%
427	PREDICTED: erythrocyte band 7 integral membrane protein [Hipposideros armiger]	XP_019503777.1	27 kDa	2,5488	0	0,1225	0	1	0	1	0	3,21%	0	98%	0
428	Cluster of ribosome-binding protein 1 isoform X1 [Bos taurus] (XP_024856794.1)	XP_024856794.1 [5]	167 kDa	0	7,2907	0	0,038883	0	2	0	2	0	2,58%	0	100%
428,1	ribosome-binding protein 1 isoform X1 [Bos taurus]	XP_024856794.1 (+4)	167 kDa	0	5,9838	0	0,038883	0	2	0	2	0	2,58%	0	100%
429	serine hydroxymethyltransferase, mitochondrial precursor [Bos taurus]	NP_001029454.1 (+2)	56 kDa	0	4,8605	0	0,11984	0	2	0	2	0	8,73%	0	100%
430	ras-related protein Rab-7a isoform X1 [Bos taurus]	XP_005223227.1 (+33)	24 kDa	0,84959	4,8605	0,1433	0,13985	1	1	1	1	6,76%	6,76%	100%	100%
431	TPA: 6-phosphogluconolactonase [Bos taurus]	DAA28196.1 (+2)	28 kDa	0	1,2151	0	0,11906	0	1	0	1	0	6,20%	0	100%
432	DNA-(apurinic or apyrimidinic site) lyase [Bos taurus]	NP_788782.2 (+6)	36 kDa	0	2,4302	0	0,091766	0	1	0	1	0	5,35%	0	100%
433	TPA: dipeptidyl peptidase 7, partial [Bos taurus]	DAA33780.1 (+5)	37 kDa	0	7,2907	0	0,087317	0	1	0	1	0	2,99%	0	100%
434	protein NipSnap homolog 2 [Bos taurus]	NP_001070479.1 (+30)	33 kDa	0	6,0756	0	0,097676	0	1	0	1	0	3,15%	0	100%
435	PREDICTED: histone H2B 1/2-like [Bos indicus]	XP_019823312.1	10 kDa	5,9471	0	0,8678	0	1	0	2	0	25,60%	0	100%	0
436	Cluster of PREDICTED: ras-related C3 botulinum toxin substrate 1 [Mandrillus leucophaeus] (XP_011852055.1)	XP_011852055.1	41 kDa	2,5488	6,5124	0	0,16656	1	2	1	2	2,73%	6,01%	100%	100%
436,1	PREDICTED: ras-related C3 botulinum toxin substrate 1 [Mandrillus leucophaeus]	XP_011852055.1	41 kDa	2,3554	6,1437	0,082013	0,16656	1	2	1	2	2,73%	6,01%	91%	95%
437	PREDICTED: alpha-actinin-4 [Balaenoptera acutorostrata scammoni]	XP_007180080.1	101 kDa	0	2,4302	0	0,031796	0	1	0	1	0	1,36%	0	100%
438	protein ERGIC-53 precursor [Bos taurus]	NP_001092413.1 (+111)	58 kDa	2,5488	0	0,057103	0	1	0	1	0	2,32%	0,00%	99%	45%
439	COP9 signalosome complex subunit 3 [Bos taurus]	NP_001092374.1 (+73)	48 kDa	0	7,2907	0	0,067957	0	1	0	1	0	2,84%	0	99%
440	PREDICTED: protein transport protein Sec61 subunit alpha isoform 1 isoform X1 [Propithecus coquereli]	XP_012514421.1 (+1)	43 kDa	1,6992	0	0,076686	0	1	0	1	0	2,75%	0	99%	0
441	Cluster of TPA: 3-hydroxyisobutyryl-CoA hydrolase, mitochondrial precursor, partial [Bos taurus] (DAA32869.1	DAA32869.1 [29]	40 kDa	0	7,2907	0	0,080487	0	1	0	1	0	3,06%	0	100%
442	prolyl 4-hydroxylase subunit alpha-1 isoform X1 [Bos taurus]	XP_005226443.1 (+3)	61 kDa	0	7,2907	0	0,16788	0	2	0	2	0	10,70%	0	100%
443	proliferating cell nuclear antigen [Bos taurus]	NP_001029666.1 (+26)	29 kDa	0	2,4302	0	0,11389	0	1	0	1	0	4,98%	0	100%
444	complement factor I precursor [Bos taurus]	NP_001033185.1 (+3)	69 kDa	0	2,4302	0	0,095915	0	2	0	2	0,00%	4,53%	80%	100%
445	TPA: DnaJ (Hsp40) homolog, subfamily C, member 13 [Bos taurus]	DAA33060.1 (+146)	254 kDa	5,0975	0	0,012915	0	1	0	1	0	0,67%	0	100%	0
446	transmembrane protein 109 precursor [Bos taurus]	NP_001039935.1 (+2)	26 kDa	2,5488	4,8605	0,12836	0,12527	1	1	1	1	6,17%	6,17%	99%	99%
447	protein O-fucosyltransferase 2b [Bos taurus]	AAQ02334.1 (+41)	53 kDa	0	8,5059	0	0,061435	0	1	0	1	0	3,05%	0	99%
448	GTP-binding protein PTD004 [Bos taurus]	ABG67106.1 (+133)	14 kDa	0	9,721	0	0,23392	0	1	0	1	0	11,30%	0	99%
449	glucose-6-phosphate dehydrogenase, partial [Bos taurus]	AAW81980.1 (+11)	25 kDa	0	2,4302	0	0,13277	0	1	0	1	0	7,51%	0	98%
450	PREDICTED: beta-galactosidase isoform X1 [Ceratotherium simum simum]	XP_004419523.1 (+1)	75 kDa	0	1,2151	0	0,043323	0	1	0	1	0	3,02%	0	98%
451	LOW QUALITY PROTEIN: telomerase reverse transcriptase [Aotus nancymaae]	XP_021531079.1	142 kDa	3,3984	0	0,023112	0	1	0	1	0	0,70%	0	95%	0
452	Cluster of heterogeneous nuclear ribonucleoprotein H2 [Bos taurus] (NP_001069476.1)	NP_001069476.1 [364]	49 kDa	0	6,0756	0	0,13595	0	2	0	2	0,00%	7,35%	79%	100%
452,1	heterogeneous nuclear ribonucleoprotein H2 [Bos taurus]	NP_001069476.1 (+363)	49 kDa	0	6,0479	0	0,13595	0	2	0	2	0,00%	7,35%	78%	100%
453	Cluster of TPA: short-chain specific acyl-CoA dehydrogenase, mitochondrial precursor [Bos taurus] (DAA20605	DAA20605.1 [82]	44 kDa	0	8,5059	0	0,23604	0	3	0	3	0	10,40%	0	100%
453,1	TPA: short-chain specific acyl-CoA dehydrogenase, mitochondrial precursor [Bos taurus]	DAA20605.1 (+81)	44 kDa	0	8,1965	0	0,23604	0	3	0	3	0	10,40%	0	100%
454	Cluster of purine nucleoside phosphorylase [Bos taurus] (AAX46392.1)	AAX46392.1 [6]	32 kDa	0	7,2907	0	0,21492	0	2	0	2	0	12,50%	0	100%
454,1	purine nucleoside phosphorylase [Bos taurus]	AAX46392.1 (+5)	32 kDa	0	7,2849	0	0,21492	0	2	0	2	0	12,50%	0	100%
455	Cluster of elongation factor Tu, mitochondrial precursor [Bos taurus] (NP_776632.1)	NP_776632.1 [8]	49 kDa	0	1,2151	0	0,065634	0	1	0	1	0	3,10%	0	100%
456	Cluster of peptidyl-prolyl cis-trans isomerase FKBP3 [Bos taurus] (NP_001033201.1)	NP_001033201.1 [31]	25 kDa	0	1,2151	0	0,13037	0	1	0	1	0	7,59%	0	100%
457	Cluster of l-arginine:glycine amidinotransferase, partial [Bos taurus] (AAT39889.1)	AAT39889.1 [112]	38 kDa	0	6,0756	0	0,086531	0	1	0	1	0	5,21%	0	100%
458	Phosphoglycerate dehydrogenase [Bos taurus]	AAI05480.1 (+6)	56 kDa	0	7,2907	0	0,11837	0	2	0	2	0	5,25%	0	100%
459	PREDICTED: peroxiredoxin-6 [Mustela putorius furo]	XP_004761988.1 (+1)	25 kDa	0	6,0756	0	0,28063	0	2	0	2	0	17,90%	0	100%
460	F-actin-capping protein subunit alpha-1 [Bos taurus]	NP_001076949.1 (+82)	33 kDa	0	3,6454	0	0,20871	0	2	0	2	0	8,74%	0	100%
461	unnamed protein product [Mus musculus]	BAE29663.1 (+41)	29 kDa	0	2,4302	0	0,11125	0	1	0	1	0	6,13%	0	100%
462	transgelin-2 [Bos taurus]	NP_001013617.1 (+43)	22 kDa	0	2,4302	0	0,31582	0	2	0	2	0	14,60%	0	100%
463	platelet-activating factor acetylhydrolase IB subunit gamma [Bos taurus]	NP_777090.1 (+25)	26 kDa	0	6,0756	0	0,27015	0	2	0	2	0	7,33%	0	100%
464	TPA: palmitoyl-protein thioesterase 1 precursor [Bos taurus]	DAA30952.1 (+10)	34 kDa	0	6,0756	0	0,09573	0	1	0	1	0	5,88%	0	100%
465	nascent polypeptide-associated complex subunit alpha isoform X1 [Microcebus murinus]	XP_012609620.1 (+2)	200 kDa	0	4,8605	0	0,016028	0	1	0	1	0	0,75%	0	100%
466	60S ribosomal protein L10-like [Dasypus novemcinctus]	XP_023439699.1	11 kDa	3,3984	0	0,30662	0	1	0	1	0	13,10%	0	100%	0
467	pyruvate carboxylase, mitochondrial isoform X1 [Microcebus murinus]	XP_012613044.1 (+3)	130 kDa	0	1,2151	0	0,024811	0	1	0	1	0	1,19%	0	100%
468	integrin beta 3 subunit [Bos taurus]	AFS60671.1 (+11)	87 kDa	2,5488	0	0,038187	0	1	0	1	0	3,19%	0	99%	0
469	NADH dehydrogenase [ubiquinone] 1 alpha subcomplex subunit 9, mitochondrial isoform X1 [Heterocephalus	XP_004869427.1 (+3)	45 kDa	0	6,0756	0	0,071677	0	1	0	1	0	4,71%	0	99%
470	PREDICTED: LOW QUALITY PROTEIN: heat shock 70 kDa protein 6-like [Rhinopithecus roxellana]	XP_010373900.1 (+1)	71 kDa	0	2,4302	0	0,045514	0	1	0	1	0	2,49%	0	98%
471	immunoglobulin light chain variable region, partial [Homo sapiens]	AMB38639.1	12 kDa	0,84959	2,4302	0,29445	0,28737	1	1	1	1	12,50%	12,50%	97%	92%
472	TPA: glutathione S-transferase alpha 5 [Bos taurus]	DAA16542.1 (+1)	25 kDa	0,83066	3,5642	0,13298	0,12978	1	1	1	1	4,05%	4,05%	6%	96%
473	hypothetical protein A6R68_15895, partial [Neotoma lepida]	OBS73567.1	6 kDa	2,5488	0	0,6056	0	1	0	1	0	17,90%	0	95%	0
474	Cluster of Alpha-centractin [Pteropus alecto] (ELK05000.1)	ELK05000.1	77 kDa	0	6,0756	0	0,13043	0	3	0	3	0	6,24%	0	100%
474,1	Alpha-centractin [Pteropus alecto]	ELK05000.1	77 kDa	0	5,8361	0	0,13043	0	3	0	3	0	6,24%	0	100%
475	Cluster of fumarate hydratase, mitochondrial [Bos taurus] (NP_001069271.1)	NP_001069271.1 [30]	55 kDa	0	2,4302	0	0,12213	0	2	0	2	0	6,27%	0	100%
475,1	fumarate hydratase, mitochondrial [Bos taurus]	NP_001069271.1 (+29)	55 kDa	0	2,2634	0	0,12213	0	2	0	2	0	6,27%	0	100%
476	Cluster of spliceosome RNA helicase DDX39B [Bos taurus] (NP_001028801.1)	NP_001028801.1 [94]	49 kDa	0	2,4302	0	0,066238	0	1	0	1	0	2,34%	0	100%
477	Cluster of unnamed protein product [Homo sapiens] (BAG63220.1)	BAG63220.1	57 kDa	0	4,8605	0	0,056717	0	1	0	1	0	3,27%	0	100%
478	Cluster of fibronectin precursor [Bos taurus] (NP_001157250.1)	NP_001157250.1 [76]	262 kDa	0,84959	0	0,012521	0	1	0	1	0	0,80%	0	100%	0
479	septin-2 [Bos taurus]	NP_001039557.1 (+146)	42 kDa	0	6,0756	0	0,16281	0	2	0	2	0	9,14%	0	100%
480	NADH dehydrogenase [ubiquinone] 1 beta subcomplex subunit 9 [Bos taurus]	NP_786977.1 (+1)	22 kDa	0	6,0756	0	0,32649	0	2	0	2	0	14,50%	0	100%
481	mitotic checkpoint protein BUB3 [Mus musculus]	AAD38038.1 (+65)	37 kDa	0	4,8605	0	0,1841	0	2	0	2	0	7,98%	0	100%
482	mitochondrial carrier homolog 2 [Bos taurus]	BAA95942.1 (+8)	33 kDa	0	2,4302	0	0,098009	0	1	0	1	0	4,62%	0	100%
483	protein HP-25 homolog 2 precursor [Bos taurus]	NP_001039544.1 (+2)	23 kDa	0	7,2907	0	0,30908	0	2	0	2	0	13,00%	0	100%
484	ubiquitin thioesterase OTUB1 [Bos taurus]	NP_001029891.1 (+7)	31 kDa	0	3,6454	0	0,1044	0	1	0	1	0	7,01%	0	100%
485	G protein beta 1 subunit [Rattus norvegicus]	AAC72249.1 (+31)	37 kDa	0	3,6454	0	0,087053	0	1	0	1	0	5,29%	0	100%
486	adenosylhomocysteinase [Bos taurus]	NP_001029487.1 (+13)	48 kDa	0	1,2151	0	0,068117	0	1	0	1	0	3,47%	0	100%

487	TPA: peptidyl-prolyl cis-trans isomerase FKBP4 [Bos taurus]	DAA29159.1 (+5)	52 kDa	0	4,8605	0	0,062911	0	1	0	1	0	7,19%	0	100%
488	TPA: armadillo repeat containing 10 isoform 2 [Bos taurus]	DAA30669.1 (+12)	31 kDa	0	6,0756	0	0,10555	0	1	0	1	0	5,65%	0	100%
489	alpha-1B-glycoprotein precursor [Bos taurus]	NP_001039708.1	54 kDa	0	2,4302	0	0,060531	0	1	0	1	0,00%	2,98%	45%	100%
490	ECH1 protein, partial [Bos taurus]	AAI02085.1 (+10)	38 kDa	0	7,2907	0	0,086791	0	1	0	1	0	4,61%	0	99%
491	protein phosphatase 1 regulatory subunit 7 [Bos taurus]	NP_001029410.1 (+169)	41 kDa	0	4,8605	0	0,078513	0	1	0	1	0	5,00%	0	99%
492	septin-11 [Bos taurus]	NP_001075916.1 (+75)	49 kDa	0	4,8605	0	0,066238	0	1	0	1	0	3,06%	0	99%
493	prenylcysteine oxidase 1 precursor [Bos taurus]	NP_001098944.1 (+6)	57 kDa	0	3,6454	0	0,057054	0	1	0	1	0	7,48%	0	99%
494	60S ribosomal protein L21 [Tupaia chinensis]	ELW66634.1 (+1)	15 kDa	1,6992	0	0,23587	0	1	0	1	0	12,00%	0	99%	0
495	C-reactive protein precursor [Bos taurus]	NP_001137569.1 (+2)	25 kDa	0	1,2151	0	0,12978	0	1	0	1	0	5,36%	0	98%
496	interleukin enhancer-binding factor 2 [Bos taurus]	NP_001033276.1 (+39)	43 kDa	0	1,2151	0	0,07543	0	1	0	1	0	2,82%	0	97%
497	eukaryotic translation elongation factor 1 delta, partial [Bos taurus]	ABQ12948.1 (+20)	33 kDa	0,84959	0	0,10181	0	1	0	1	0	8,03%	0	95%	0
498	carbonic anhydrase 14 precursor [Bos taurus]	NP_001179134.1 (+3)	38 kDa	0	3,6454	0	0,086014	0	1	0	1	0	3,57%	0	95%
499	Cluster of PREDICTED: enoyl-CoA hydratase, mitochondrial [Jaculus jaculus] (XP_012806921.1)	XP_012806921.1 [2]	31 kDa	0	12,683	0	0,21942	0	3	0	3	0	11,80%	0	100%
499,1	PREDICTED: enoyl-CoA hydratase, mitochondrial [Jaculus jaculus]	XP_012806921.1	31 kDa	0	6,3451	0	0,22067	0	2	0	2	0	11,80%	0	100%
499,2	PREDICTED: enoyl-CoA hydratase, mitochondrial isoform X1 [Nannospalax galili]	XP_017651109.1	32 kDa	0	6,3379	0	0,21817	0	2	0	2	0	11,70%	0	100%
500	dihydropteridine reductase [Odocoileus virginianus texanus]	XP_020766344.1	24 kDa	0	3,6454	0	0,47392	0	3	0	3	0	17,80%	0	100%
501	Aminolevulinate, delta-, dehydratase [Bos taurus]	AAI12597.1 (+11)	36 kDa	0	6,0756	0	0,18891	0	2	0	2	0	16,40%	0	100%
502	NADH dehydrogenase 24 kDa subunit (AA 6-217), partial [Bos taurus]	CAA32848.1 (+120)	24 kDa	0	3,6454	0	0,2995	0	2	0	2	0	10,80%	0	100%
503	complement C3-like, partial [Ictidomys tridecemlineatus]	XP_021577305.1	38 kDa	2,6874	1,1754	0,1828	0,085503	2	1	2	1	10,10%	6,65%	100%	95%
504	TPA: aldo-keto reductase family 7, member A2, partial [Bos taurus]	DAA32096.1 (+2)	29 kDa	0	4,8605	0	0,11479	0	1	0	1	0	5,73%	0	100%
505	carboxymethylenebutenolidase homolog [Mus musculus]	NP_853619.1 (+2)	28 kDa	0	2,4302	0	0,1176	0	1	0	1	0	6,94%	0	100%
506	Peroxisomal D3,D2-enoyl-CoA isomerase [Bos taurus]	AAI02907.1 (+14)	24 kDa	0	4,8605	0	0,13985	0	1	0	1	0	7,83%	0	99%
507	eukaryotic translation initiation factor 2 subunit 1, partial [Macaca fascicularis]	AAV66398.1 (+49)	26 kDa	0	4,8605	0	0,12748	0	1	0	1	0	5,33%	0	99%
508	PREDICTED: protein SET [Ovis aries musimon]	XP_014962407.1 (+1)	34 kDa	0	4,8605	0	0,09637	0	1	0	1	0	11,00%	0	99%
509	PREDICTED: LOW QUALITY PROTEIN: N(G),N(G)-dimethylarginine dimethylaminohydrolase 2 isoform X1 [Ovis	XP_011956318.1	30 kDa	0	3,6454	0	0,1104	0	1	0	1	0	4,93%	0	99%
510	proteasome subunit beta type (predicted) [Plecturocebus moloch]	ACA57936.1	29 kDa	0	1,2151	0	0,11389	0	1	0	1	0	5,75%	0	99%
511	PREDICTED: adenylate kinase 2, mitochondrial isoform X2 [Ornithorhynchus anatinus]	XP_007669942.1 (+1)	21 kDa	0	2,4302	0	0,16002	0	1	0	1	0	13,50%	0	98%
512	MICOS complex subunit MIC19 [Bos taurus]	NP_001030552.1 (+205)	26 kDa	0	4,8605	0	0,12582	0	1	0	1	0	5,29%	0	96%
513	Cluster of solute carrier family 25 (mitochondrial carrier; oxoglutarate carrier), member 11, partial [Bos taurus	ABQ12913.1 [9]	34 kDa	0	4,8605	0	0,31823	0	3	0	3	0	9,65%	0	100%
513,1	solute carrier family 25 (mitochondrial carrier; oxoglutarate carrier), member 11, partial [Bos taurus]	ABQ12913.1 (+8)	34 kDa	0	4,8035	0	0,31823	0	3	0	3	0	9,65%	0	100%
514	Cluster of succinate--CoA ligase [GDP-forming] subunit beta, mitochondrial precursor [Bos taurus] (NP_00102	NP_001029811.1 [9]	47 kDa	0	4,8605	0	0,1441	0	2	0	2	0	9,49%	0	100%
514,1	succinate--CoA ligase [GDP-forming] subunit beta, mitochondrial precursor [Bos taurus]	NP_001029811.1 (+8)	47 kDa	0	4,8124	0	0,1441	0	2	0	2	0	9,49%	0	100%
515	Cluster of pyrroline-5-carboxylate reductase 1, mitochondrial [Bos taurus] (NP_001014957.1)	NP_001014957.1 [96]	33 kDa	0	3,6454	0	0,20501	0	2	0	2	0	8,44%	0	100%
515,1	pyrroline-5-carboxylate reductase 1, mitochondrial [Bos taurus]	NP_001014957.1 (+95)	33 kDa	0	3,5955	0	0,20501	0	2	0	2	0	8,44%	0	100%
516	Cluster of NADH dehydrogenase (ubiquinone) Fe-S protein 8, 23kDa (NADH-coenzyme Q reductase) [Bos tauru	AAI09907.1 [14]	24 kDa	0	3,6454	0	0,13785	0	1	0	1	0	5,66%	0	100%
517	hypoxanthine phosphoribosyltransferase, partial [Bos taurus]	AAG09236.1 (+154)	21 kDa	0	1,2151	0	0,1574	0	1	0	1	0	6,99%	0	100%
518	TMED4 protein, partial [Bos taurus]	AAI23581.1 (+122)	28 kDa	0,84959	0	0,12199	0	1	0	1	0	7,88%	0	100%	0
519	protein ABHD11 [Bos taurus]	NP_001029544.1 (+7)	34 kDa	0	2,4302	0	0,097346	0	1	0	1	0	4,62%	0	100%
520	PREDICTED: 26S proteasome non-ATPase regulatory subunit 5 [Marmota marmota marmota]	XP_015353163.1 (+2)	56 kDa	0	3,6454	0	0,05774	0	1	0	1	0	1,79%	0	100%
521	LOW QUALITY PROTEIN: transmembrane emp24 domain-containing protein 2 [Neophocaena asiaeorientalis a	XP_024614408.1	23 kDa	0	3,6454	0	0,1455	0	1	0	1	0	6,50%	0	100%
522	ER membrane protein complex subunit 2 [Bos taurus]	NP_001073796.1 (+8)	35 kDa	0	1,2151	0	0,093859	0	1	0	1	0	4,04%	0	100%
523	PREDICTED: LOW QUALITY PROTEIN: non-POU domain-containing octamer-binding protein-like [Panthera tigr	XP_015394475.1	53 kDa	0	2,4302	0	0,061566	0	1	0	1	0	2,40%	0	100%
524	glyceraldehyde-3-phosphate dehydrogenase, partial [Heterocephalus glaber]	BAJ24843.1	24 kDa	8,976	14,29	0,30688	1,8612	2	7	2	8	8,00%	48,90%	46%	99%
525	alpha-soluble NSF attachment protein [Bos taurus]	NP_001179416.1 (+113)	33 kDa	3,3984	0	0,10077	0	1	0	1	0	4,41%	0	99%	0
526	retinoid-inducible serine carboxypeptidase precursor [Bos taurus]	NP_001039374.1 (+3)	51 kDa	0	1,2151	0	0,063746	0	1	0	1	0	3,12%	0	99%
527	sepiapterin reductase isoform 2 [Bos taurus]	ABI79460.1 (+6)	29 kDa	0	4,8605	0	0,11299	0	1	0	1	0	12,00%	0	99%
528	unnamed protein product [Mus musculus]	BAC37223.1	36 kDa	1,6992	2,4302	0,091977	0,089765	1	1	1	1	3,37%	3,37%	96%	99%
529	PREDICTED: LOW QUALITY PROTEIN: protein NipSnap homolog 1 [Balaenoptera acutorostrata scammoni]	XP_007170789.1	33 kDa	0	3,6454	0	0,098009	0	1	0	1	0	3,17%	0	99%
530	transmembrane emp24 domain-containing protein 1 precursor [Bos taurus]	NP_001033652.1 (+22)	25 kDa	0	4,8605	0	0,13096	0	1	0	1	0	3,96%	0	99%
531	PREDICTED: histone H2B type 1-H [Bos mutus]	XP_005887000.1	14 kDa	0,84959	0	0,25192	0	1	0	1	0	17,60%	0	99%	0
532	PREDICTED: LOW QUALITY PROTEIN: ATPase family AAA domain-containing protein 1 [Rhinopithecus roxellan	XP_010377929.1	41 kDa	0	1,2151	0	0,08004	0	1	0	1	0	4,43%	0	98%
533	leucine-rich repeat-containing protein 59 [Bos taurus]	NP_001029750.1 (+83)	35 kDa	0	1,2151	0	0,093554	0	1	0	1	0	3,92%	0	98%
534	Cathepsin Z [Myotis brandtii]	EPQ19839.1 (+6)	34 kDa	0	2,4302	0	0,096049	0	1	0	1	0	3,99%	0	98%
535	cytochrome P450c17 [Ursus thibetanus japonicus]	BAH82843.1 (+45)	57 kDa	0	4,8605	0	0,056495	0	1	0	1	0	3,15%	0	97%
536	acyl-protein thioesterase 1 [Bos taurus]	NP_001029860.1 (+159)	25 kDa	0	3,6454	0	0,134	0	1	0	1	0	6,09%	0	97%
537	PREDICTED: mammalian ependymin-related protein 1 [Bos indicus]	XP_019814267.1 (+93)	26 kDa	0	3,6454	0	0,12419	0	1	0	1	0	4,66%	0	97%
538	protein canopy homolog 4 precursor [Bos taurus]	NP_001029425.1 (+1)	27 kDa	0	2,4302	0	0,12005	0	1	0	1	0	14,20%	0	97%
539	clathrin interactor 1 [Bos taurus]	NP_001098887.1 (+43)	71 kDa	0,84959	0	0,046932	0	1	0	1	0	3,11%	0	96%	0
540	TPA: IGK protein-like [Bos taurus]	DAA24658.1 (+2)	14 kDa	1,6992	0	0,2477	0	1	0	1	0	7,14%	0	96%	0
541	PREDICTED: calcium-binding mitochondrial carrier protein Aralar1 [Ornithorhynchus anatinus]	XP_007669417.1	65 kDa	0	1,2151	0	0,049933	0	1	0	1	0,00%	2,72%	9%	95%
542	pigment epithelium-derived factor precursor [Bos taurus]	NP_776565.1 (+2)	46 kDa	0	2,4302	0	0,14555	0	2	0	2	0	8,89%	0	100%
543	IDH3B protein, partial [Bos taurus]	AAI04503.1 (+175)	42 kDa	0	1,2151	0	0,076838	0	1	0	1	0	4,95%	0	100%
544	cytochrome oxidase subunit II (mitochondrion) [Bos taurus]	AAA31644.1 (+108)	26 kDa	0	2,4302	0	0,12637	0	1	0	1	0	7,05%	0	100%
545	protein NipSnap homolog 3A [Bos taurus]	NP_001073767.1 (+2)	29 kDa	0	1,2151	0	0,11479	0	1	0	1	0	4,05%	0	100%
546	RAB14 protein [Mus musculus]	CAI92129.1 (+1)	24 kDa	0,84959	2,4302	0,14124	0,13785	1	1	1	1	14,00%	14,40%	99%	100%
547	phenylalanine--tRNA ligase alpha subunit [Bos taurus]	NP_001094577.1 (+5)	57 kDa	0	1,2151	0	0,056274	0	1	0	1	0	2,76%	0	100%
548	Hydroxysteroid (17-beta) dehydrogenase 4 [Bos taurus]	AAI22585.1 (+2)	80 kDa	0	1,2151	0	0,040577	0	1	0	1	0	3,67%	0	100%
549	hsc70-interacting protein [Bos taurus]	NP_001095396.1 (+57)	41 kDa	0	1,2151	0	0,078513	0	1	0	1	0	3,79%	0	100%
550	valacyclovir hydrolase [Bos taurus]	NP_001039383.1 (+2)	32 kDa	0	2,4302	0	0,10075	0	1	0	1	0	4,81%	0	100%
551	branched chain aminotransferase 2, mitochondrial, partial [Bos taurus]	ABG67061.1 (+14)	44 kDa	0	1,2151	0	0,073318	0	1	0	1	0	2,06%	0	99%
552	protein transport protein Sec24C [Bos taurus]	NP_001192584.1 (+42)	118 kDa	1,6992	0	0,027951	0	1	0	1	0	1,19%	0	99%	0
553	N-acetyl galactosaminidase, alpha [Mus musculus]	AAH21631.1 (+45)	47 kDa	0	3,6454	0	0,068768	0	1	0	1	0	4,58%	0	99%
554	Atp2a2, partial [Bos taurus]	AAW29825.1 (+72)	50 kDa	0	1,2151	0	0,064749	0	1	0	1	0	3,52%	0	98%
555	acid ceramidase precursor [Bos taurus]	NP_001068927.1 (+5)	45 kDa	0	2,4302	0	0,072216	0	1	0	1	0	2,53%	0	98%
556	GPI-anchor transamidase [Fukomys damarensis]	KFO23258.1 (+3)	43 kDa	0	3,6454	0	0,074842	0	1	0	1	0	3,18%	0	98%
557	proteasome activator complex subunit 2 [Bos taurus]	NP_001014889.1 (+2)	27 kDa	0	2,4302	0	0,11956	0	1	0	1	0	6,69%	0	98%
558	platelet-activating factor acetylhydrolase IB subunit beta isoform c [Mus musculus]	NP_001344169.1	26 kDa	0	2,4302	0	0,12637	0	1	0	1	0	3,88%	0	98%
559	DNA repair and recombination protein RAD54B isoform X2 [Microcebus murinus]	XP_012628306.1	103 kDa	0,84959	0	0,03191	0	1	0	1	0	1,54%	0	97%	0
560	PREDICTED: 60S ribosomal protein L32 [Erinaceus europaeus]	XP_007517145.1	11 kDa	0,84959	0	0,30982	0	1	0	1	0	18,30%	0	96%	0

561	glutathione S-transferase alpha I [Oryctolagus cuniculus]	NP_001164568.1	26 kDa	0	1,2151	0	0,12805	0	1	0	1	0,00%	10,80%	56%	96%
562	leucine-rich alpha-2-glycoprotein precursor [Bos taurus]	NP_001039642.1 (+4)	38 kDa	0	2,4302	0	0,084999	0	1	0	1	0	3,47%	0	96%
563	Cluster of LOW QUALITY PROTEIN: thioredoxin-like protein 1 [Loxodonta africana] (XP_003406335.1)	XP_003406335.1	32 kDa	0	1,2151	0	0,1011	0	1	0	1	0	5,19%	0	100%
564	propionyl-CoA carboxylase beta chain, mitochondrial precursor [Bos taurus]	NP_001033637.1 (+39)	58 kDa	0	2,4302	0	0,11415	0	2	0	2	0	6,12%	0	100%
565	succinyl-CoA:3-ketoacid coenzyme A transferase 1, mitochondrial [Bos taurus]	NP_001069538.1 (+11)	56 kDa	0	2,4302	0	0,11812	0	2	0	2	0	6,54%	0	100%
566	PREDICTED: inorganic pyrophosphatase 2, mitochondrial [Condylura cristata]	XP_004686519.1	37 kDa	0	2,4302	0	0,087053	0	1	0	1	0	4,80%	0	100%
567	CMP-N-acetylneuraminate-beta-galactosamide-alpha-2, 3-sialyltransferase 4, partial [Bos mutus]	ELR58858.1	39 kDa	0	1,2151	0	0,082801	0	1	0	1	0	4,55%	0	100%
568	PREDICTED: serine hydroxymethyltransferase, mitochondrial [Ceratotherium simum simum]	XP_004429349.1	57 kDa	0	2,4302	0	0,056717	0	1	0	1	0	6,24%	0	100%
569	beta-hexosaminidase subunit alpha precursor [Bos taurus]	NP_001068632.1 (+11)	60 kDa	0	1,2151	0	0,053653	0	1	0	1	0	3,21%	0	100%
570	protein FAM3A isoform X4 [Phascolarctos cinereus]	XP_020831307.1 (+2)	33 kDa	0	1,2151	0	0,099704	0	1	0	1	0	4,03%	0	99%
571	heparin cofactor 2 isoform X1 [Bos taurus]	XP_005218256.1 (+1)	63 kDa	1,6992	0	0,052811	0	1	0	1	0	2,30%	0	99%	0
572	PREDICTED: serine/arginine-rich splicing factor 8 [Myotis brandtii]	XP_005856796.1	18 kDa	0,84959	0	0,18675	0	1	0	1	0	11,00%	0	99%	0
573	galactokinase 1, partial [Bos taurus]	ABF57297.1 (+4)	31 kDa	0	2,4302	0	0,1044	0	1	0	1	0	6,21%	0	99%
574	TPA: serine/threonine-protein phosphatase PP1-beta catalytic subunit, partial [Bos taurus]	DAA24418.1 (+214)	35 kDa	0	2,4302	0	0,092652	0	1	0	1	0	5,50%	0	99%
575	protein O-glucosyltransferase 1 precursor [Bos taurus]	NP_001014903.1 (+127)	46 kDa	0	2,4302	0	0,070622	0	1	0	1	0	3,57%	0	99%
576	ribonuclease inhibitor [Bos taurus]	NP_001030396.1 (+1)	49 kDa	0	2,4302	0	0,066391	0	1	0	1	0	4,61%	0	99%
577	small glutamine-rich tetratricopeptide repeat-containing protein alpha [Bos taurus]	NP_001033119.1 (+13)	34 kDa	0	2,4302	0	0,095413	0	1	0	1	0	5,43%	0	99%
578	ATPase family AAA domain-containing protein 3 [Bos taurus]	NP_001098932.1 (+132)	66 kDa	0	1,2151	0	0,048915	0	1	0	1	0	2,39%	0	99%
579	alpha-2-HS-glycoprotein precursor [Bos taurus]	NP_776409.1 (+10)	38 kDa	0	1,2151	0	0,084749	0	1	0	1	0	4,74%	0	99%
580	PREDICTED: LOW QUALITY PROTEIN: synaptic vesicle membrane protein VAT-1 homolog isoform X1 [Ovis arie	XP_012041537.2	54 kDa	0	1,2151	0	0,060404	0	1	0	1	0	3,61%	0	98%
581	mitochondrial diablo [Sus scrofa]	ACJ24820.1 (+1)	27 kDa	0	2,4302	0	0,12261	0	1	0	1	0	4,22%	0	97%
582	PREDICTED: enoyl-CoA hydratase, mitochondrial [Rhinolophus sinicus]	XP_019583885.1	30 kDa	0	1,2151	0	0,11082	0	1	0	1	0	6,18%	0	95%
583	inorganic pyrophosphatase 2, mitochondrial [Bos taurus]	NP_001069864.1 (+29)	37 kDa	0	1,2151	0	0,088118	0	1	0	1	0	4,89%	0	100%
584	Protein FAM3C, partial [Bos mutus]	ELR57312.1 (+1)	24 kDa	0	1,2151	0	0,13526	0	1	0	1	0	7,62%	0	100%
585	heterochromatin protein 1-binding protein 3 [Bos taurus]	NP_001068904.1 (+106)	61 kDa	0,84959	0	0,053872	0	1	0	1	0	2,34%	0	99%	0
586	PREDICTED: LOW QUALITY PROTEIN: interferon-induced transmembrane protein 3-like [Callithrix jacchus]	XP_008992126.1	15 kDa	0,84959	0	0,23217	0	1	0	1	0	12,00%	0	99%	0
587	peptidyl-prolyl cis-trans isomerase C precursor [Bos taurus]	NP_001070378.1	23 kDa	0	1,353	0	0,31074	0	2	0	2	0	12,70%	0	99%
588	Dicarbonyl/L-xylulose reductase [Bos taurus]	AAI42211.1 (+12)	26 kDa	0	1,2151	0	0,12805	0	1	0	1	0	6,56%	0	99%
589	isocitrate dehydrogenase 3 (NAD+) gamma isoform a precursor [Bos taurus]	AAX46424.1 (+18)	43 kDa	0	1,2151	0	0,075827	0	1	0	1	0	3,57%	0	99%
590	pyruvate dehydrogenase protein X component precursor [Bos taurus]	NP_001069219.1 (+11)	54 kDa	0	1,2151	0	0,060152	0	1	0	1	0	2,79%	0	99%
591	coatomer subunit delta [Bos taurus]	NP_001181942.1 (+115)	57 kDa	0	1,2151	0	0,056495	0	1	0	1	0	2,35%	0	99%
592	TPA: COMM domain-containing protein 9 [Bos taurus]	DAA21833.1 (+28)	22 kDa	0	1,2151	0	0,15161	0	1	0	1	0	8,08%	0	99%
593	angiotensinogen precursor [Bos taurus]	NP_001107554.1 (+9)	45 kDa	0	1,2151	0	0,071499	0	1	0	1	0	4,25%	0	98%
594	SUMO-activating enzyme subunit 1 [Bos taurus]	NP_001075180.1 (+4)	38 kDa	0	1,2151	0	0,084999	0	1	0	1	0	5,78%	0	98%
595	U2 small nuclear ribonucleoprotein A' [Bos taurus]	NP_001092418.1 (+63)	28 kDa	0	1,2151	0	0,11572	0	1	0	1	0	11,00%	0	98%
596	Pentatricopeptide repeat-containing protein 2 [Heterocephalus glaber]	EHB03324.1 (+3)	39 kDa	0	1,2151	0	0,083521	0	1	0	1	0	2,38%	0	96%
597	dehydrogenase/reductase SDR family member 7B [Bos taurus]	NP_001030420.1 (+16)	35 kDa	0	1,2151	0	0,093251	0	1	0	1	0	4,92%	0	96%
598	PREDICTED: nucleotide exchange factor SIL1 [Erinaceus europaeus]	XP_007517663.1	49 kDa	0	1,2151	0	0,065485	0	1	0	1	0	2,96%	0	95%
599	Coatomer protein complex, subunit epsilon [Bos taurus]	AAI09964.1 (+19)	34 kDa	0	1,2151	0	0,094474	0	1	0	1	0	3,90%	0	95%
600	PREDICTED: EF-hand calcium-binding domain-containing protein 3 [Nomascus leucogenys]	XP_003270847.2	51 kDa	0,84959	0	0,064602	0	1	0	1	0	5,79%	0	95%	0
601	LOW QUALITY PROTEIN: complement factor H-related protein 4 [Macaca nemestrina]	XP_011744782.1	78 kDa	0	1,2151	0	0,041098	0	1	0	1	0	6,05%	0	95%

END OF FILE
