# Supplementary material for: Protein Cargo of Extracellular Vesicles From Bovine Follicular Fluid and Analysis of Their Origin From Different Ovarian Cells
Source: Front Vet Sci. 2020 Nov 4;7:584948. doi: 10.3389/fvets.2020.584948 (PMC7672127; doi:10.3389/fvets.2020.584948)
Supplement: Supplementary Table 2 — List of proteins identified in bovine ffEVs and GC. [file Table_2.pdf]

Supplementary data Table S2.

List of proteins identified in bovine follicular fluid extracellular vesicles (ff-EVs) and granulosa cells (GC)

ff-EV dominant proteins		
**Bt acc**	**Description (exo-specific proteins)**	**Official symblol**
DAA19706.1	adaptor-related protein complex 2, alpha 1 subunit [Bos taurus]	AP2A1
NP_001068593.1	AP-2 complex subunit beta [Bos taurus]	AP2B1
DAA29912.1	ADP-ribosylation factor 3 [Bos taurus]	ARF3
NP_776930.1	V-type proton ATPase subunit d 1 [Bos taurus]	ATP6V0D1
XP_024843970.1	copper-transporting ATPase 1 isoform X1 [Bos taurus]	ATP7A
NP_001040064.1	complement C1q subcomponent subunit B precursor [Bos taurus]	C1QB
NP_001040049.1	protein HP-20 homolog precursor [Bos taurus] MGC137014	C1QTNF4
AKE14289.1	complement component 5 [Bos taurus]	C5
NP_001178322.1	CAD protein [Bos taurus]	CAD
XP_024832523.1	calmodulin-binding transcription activator 1 isoform X6 [Bos taurus]	CAMTA1
DAA13541.1	CD81 antigen, partial [Bos taurus]	CD81
DAA21472.1	hypothetical protein LOC790886, partial [Bos taurus]	CFH
NP_001098887.1	clathrin interactor 1 [Bos taurus]	CLINT1
XP_005210119.1	clathrin light chain A isoform X3 [Bos taurus]	CLTA
NP_776327.1	clusterin preproprotein [Bos taurus]	CLU
DAA17141.1	CKLF-like MARVEL transmembrane domain containing 6 [Bos taurus]	CMTM6
XP_010814170.2	luster of carboxypeptidase D [Bos taurus]	CPD
NP_001029933.1	dolichyl-diphosphooligosaccharide--protein glycosyltransferase subunit DAD1 [Bos tauru	DAD1
DAA33060.1	DnaJ homolog, subfamily C, member 13 [Bos taurus]	DNAJC13
XP_002694948.2	enhancer of mRNA-decapping protein 4 isoform X1 [Bos taurus]	EDC4
ABQ12948.1	eukaryotic translation elongation factor 1 delta, partial [Bos taurus]	EEF1D
XP_024835965.1	EF-hand calcium-binding domain-containing protein 3 isoform X1 [Bos taurus]	EFCAB3
NP_001015628.1	eukaryotic translation initiation factor 3 subunit I [Bos taurus]	EIF3A
NP_001095826.1	eukaryotic translation initiation factor 3 subunit B [Bos taurus]	EIF3B
NP_001029790.1	eukaryotic translation initiation factor 3 subunit C [Bos taurus]	EIF3CL
DAA20521.1	EMI domain containing 1 [Bos taurus]	EMID1
NP_001029508.1	enhancer of rudimentary homolog [Bos taurus]	ERH
NP_001157250.1	fibronectin precursor [Bos taurus]	FN1
DAA17430.1	ferritin light chain-like [Bos taurus]	FTL
DAA18755.1	FUS interacting protein 1-like [Bos taurus]	FUSIP1
NP_001179392.1	Golgi membrane protein 1 [Bos taurus]	GOLM1
NP_777234.1	histone H2A.Z [Bos taurus]	H2AZ1
NP_001032546.1	histone H2B type 1 [Bos taurus]	H2BC19
NP_001092854.1	histone H2B type 2-E [Bos taurus]	H2BC21
NP_001014868.1	hyaluronan-binding protein 2 precursor [Bos taurus]	HABP2
NP_001192358.1	heterogeneous nuclear ribonucleoprotein A3 [Bos taurus]	HNRNPA3
NP_001070388.2	heterogeneous nuclear ribonucleoprotein U [Bos taurus]	HNRNPU
NP_001068904.1	heterochromatin protein 1-binding protein 3 [Bos taurus]	HP1BP3
DAA33337.1	histidine-rich glycoprotein [Bos taurus]	HRG
AAK58639.1	inteferon-induced membrane protein Leu-13/9-27 [Bos taurus]	IFITM1
NP_001071609.1	interferon-induced transmembrane protein 3 [Bos taurus]	IFITM3
AAI14802.1	Immunoglobulin light chain, lambda gene cluster [Bos taurus]	IGL@
NP_001075908.1	immunoglobulin superfamily member 8 precursor [Bos taurus]	IGSF8
AFS60671.1	integrin beta 3 subunit [Bos taurus]	ITGB3
NP_001091485.1	inter-alpha-trypsin inhibitor heavy chain H2 precursor [Bos taurus]	ITIH2

DAA24658.1	IGK protein-like [Bos taurus]	LOC100299426
DAA29303.1	splicing factor, arginine/serine-rich 3-like [Bos taurus]	LOC786295
NP_777132.1	matrix Gla protein precursor [Bos taurus]	MGP
NP_001028780.1	macrophage migration inhibitory factor [Bos taurus]	MIF
NP_001039503.1	protein LYRIC [Bos taurus]	MTDH
NP_001030394.1	major vault protein [Bos taurus]	MVP
NP_777259.1	myosin-10 [Bos taurus]	MYH10
NP_001015640.1	myosin regulatory light polypeptide 9 [Bos taurus]	MYL12A
XP_005206559.1	myosin light polypeptide 6 isoform X1 [Bos taurus]	MYL6
NP_001179416.1	alpha-soluble NSF attachment protein [Bos taurus]	NAPA
NP_001070601.1	ORM1-like protein 1 [Bos taurus]	ORMDL1
NP_001069835.1	ORM1-like protein 3 [Bos taurus]	ORMDL3
XP_005201869.1	profilin-2 isoform X1 [Bos taurus]	PFN2
NP_976239.1	proteolipid protein 2 [Bos taurus]	PLP2
NP_001070325.1	phosphoribosyl pyrophosphate synthase-associated protein 1 [Bos taurus]	PRPSAP1
DAA19427.1	ribosomal protein S29-like [Bos taurus]	PS29
NP_001179884.1	DNA repair and recombination protein RAD54B [Bos taurus]	RAD54B
NP_001098962.1	protein RER1 [Bos taurus]	RER1
NP_777185.1	60S ribosomal protein L10 [Bos taurus]	RPL10
DAA29048.1	ribosomal protein L10-like [Bos taurus]	RPL10L
XP_005203175.1	60S ribosomal protein L11 isoform X1 [Bos taurus]	RPL11
BAC56456.1	similar to ribosomal protein L12, partial [Bos taurus]	RPL12
NP_001015543.1	60S ribosomal protein L13 [Bos taurus]	RPL13
NP_001070466.1	60S ribosomal protein L13a [Bos taurus]	RPL13A
NP_001029846.1	60S ribosomal protein L14 [Bos taurus]	RPL14
AAX08723.1	ribosomal protein L15 [Bos taurus]	RPL15
BAC56477.1	similar to ribosomal protein L17, partial [Bos taurus]	RPL17
ABD77172.1	ribosomal protein L18, partial [Bos taurus]	RPL18
NP_001028791.1	60S ribosomal protein L18a [Bos taurus]	RPL18A
DAA27107.1	60S ribosomal protein L19, partial [Bos taurus]	RPL19
NP_001178341.1	60S ribosomal protein L21 [Bos taurus]	RPL21
NP_001030186.1	60S ribosomal protein L23 [Bos taurus]	RPL23
DAA22605.1	ribosomal protein L23a-like [Bos taurus]	RPL23A
BAC56435.1	similar to ribosomal protein L26, partial [Bos taurus]	RPL26
NP_001029223.1	60S ribosomal protein L27 [Bos taurus]	RPL27
AAI09684.1	Ribosomal protein L27a [Bos taurus]	RPL27A
BAC56497.1	similar to ribosomal protein L30, partial [Bos taurus]	RPL30
NP_001029955.1	60S ribosomal protein L32 [Bos taurus]	RPL32
NP_001029667.1	60S ribosomal protein L35 [Bos taurus]	RPL35
DAA23035.1	ribosomal protein L35a-like [Bos taurus]	RPL35A
NP_001071607.1	60S ribosomal protein L36 [Bos taurus]	RPL36
NP_001106780.1	60S ribosomal protein L38 [Bos taurus]	RPL38
NP_001014894.1	60S ribosomal protein L4 [Bos taurus]	RPL4
NP_001026926.1	60S ribosomal protein L6 [Bos taurus]	RPL6
NP_001035610.1	60S ribosomal protein L7a [Bos taurus]	RPL7A
NP_001029797.1	60S ribosomal protein L8 [Bos taurus]	RPL8
NP_001019640.2	60S ribosomal protein L9 [Bos taurus]	RPL9
NP_001029888.1	40S ribosomal protein S10 [Bos taurus]	RPS10
NP_001019739.1	40S ribosomal protein S11 [Bos taurus]	RPS11
NP_001020513.1	40S ribosomal protein S13 [Bos taurus]	RPS13
XP_024849610.1	40S ribosomal protein S14 isoform X1 [Bos taurus]	RPS14

NP_001092680.1	40S ribosomal protein S17 [Bos taurus]	RPS17
BAC56379.1	similar to40S ribosomal protein S18, partial [Bos taurus]	RPS18
DAA24498.1	ribosomal protein S23-like [Bos taurus]	RPS23
DAA17032.1	ribosomal protein S24-like [Bos taurus]	RPS24
NP_001020486.1	40S ribosomal protein S25 [Bos taurus]	RPS25
NP_001015561.1	40S ribosomal protein S26 [Bos taurus]	RPS26
NP_777203.1	ubiquitin-40S ribosomal protein S27a [Bos taurus]	RPS27A
NP_001035668.1	40S ribosomal protein S27-like [Bos taurus]	RPS27L
NP_001029210.1	40S ribosomal protein S3a [Bos taurus]	RPS3A
DAA26959.1	40S ribosomal protein S6, partial [Bos taurus]	RPS6
NP_001020488.1	40S ribosomal protein S8 [Bos taurus]	RPS8
NP_001094622.1	40S ribosomal protein S9 [Bos taurus]	RPS9
NP_001095640.1	secretory carrier-associated membrane protein 2 [Bos taurus]	SCAMP2
NP_001030503.1	secretory carrier-associated membrane protein 3 [Bos taurus]	SCAMP3
ABQ12975.1	syntenin [Bos taurus]	SDCBP
NP_001192584.1	protein transport protein Sec24C [Bos taurus]	SEC24C
NP_001035594.1	protein transport protein Sec61 subunit alpha isoform 1 [Bos taurus]	SEC61A1
NP_001039557.1	septin-2 [Bos taurus]	SEPT2
XP_005218256.1	heparin cofactor 2 isoform X1 [Bos taurus]	SERPIND1
AAI48904.1	SMN protein, partial [Bos taurus]	SMN
DAA31548.1	sortilin 1 [Bos taurus]	SORT1
NP_001020497.1	serglycin precursor [Bos taurus]	SRGN
NP_001029449.1	serine/arginine-rich splicing factor 7 [Bos taurus]	SRSF7
NP_001029872.1	serine/arginine-rich splicing factor 3 [Bos taurus]	SRSF8
NP_001033592.1	translocon-associated protein subunit delta precursor [Bos taurus]	SSR4
NP_001098943.1	erythrocyte band 7 integral membrane protein [Bos taurus]	STOM
NP_001075900.1	syntaxin-6 [Bos taurus]	STX6
NP_001071332.1	syntaxin-7 [Bos taurus]	STX7
NP_001039707.1	telomerase reverse transcriptase [Bos taurus]	TERT
NP_001193506.1	transferrin receptor protein 1 [Bos taurus]	TFRC
XP_002698454.2	tolloid-like protein 2 isoform X1 [Bos taurus]	TLL2
AAI23581.1	TMED4 protein, partial [Bos taurus]	TMED4
NP_001030514.1	transmembrane protein 14C [Bos taurus]	TMEM14C
NP_001107983.1	protein kish-A precursor [Bos taurus]	TMEM167A
NP_001014940.1	transmembrane protein 35A [Bos taurus]	TMEM35A
NP_001029948.1	transformer-2 protein homolog beta [Bos taurus]	TRA2B
NP_776392.1	transthyretin precursor [Bos taurus]	TTR

ff-EV /GC common proteins

**Bt Acc**	**Dscription (common proteins GC-exo)**	Officila symbol
NP_001039708.1	alpha-1B-glycoprotein precursor [Bos taurus]	A1BG
NP_001103265.1	alpha-2-macroglobulin precursor [Bos taurus]	A2M
DAA24846.1	TPA: actin, gamma-enteric smooth muscle [Bos taurus]	ACTL7A
NP_001075907.1	ADP-dependent glucokinase precursor [Bos taurus]	ADPGK
NP_001012537.1	aldose reductase [Bos taurus]	AKR1B1
NP_851335.1	serum albumin precursor [Bos taurus]	ALB
NP_001068835.1	aldehyde dehydrogenase, mitochondrial precursor [Bos taurus]	ALDH1L2
AAI02517.1	Annexin A2 [Bos taurus]	ANXA2
NP_001001440.2	annexin A4 [Bos taurus]	ANXA4
DAA28951.1	TPA: annexin A5 [Bos taurus]	ANXA5
NP_001096694.1	annexin A6 [Bos taurus]	ANXA6

XP_024856500.1	adipocyte plasma membrane-associated protein isoform X1 [Bos taurus]	APMAP
NP_776667.2	apolipoprotein A-I preproprotein [Bos taurus]	APOA1
NP_001032557.1	apolipoprotein A-IV precursor [Bos taurus]	APOA4
AAI34730.1	Apolipoprotein E [Bos taurus]	APOE
NP_001070266.1	sodium/potassium-transporting ATPase subunit alpha-1 [Bos taurus]	ATP1A1
AAI02617.1	ATPase, H+ transporting, lysosomal 31kDa, V1 subunit E1 [Bos taurus]	ATP6V1E1
DAA33498.1	TPA: UDP-Gal:betaGlcNAc beta 1,4- galactosyltransferase, polypeptide 4 [Bos taurus]	B4GALT4
NP_001091040.1	biliverdin reductase A [Bos taurus]	BLVRA
NP_001068839.1	basigin precursor [Bos taurus]	BSG
AKE14287.1	complement component 3 [Bos taurus]	C3
XP_005223750.2	complement C4-like isoform X2 [Bos taurus]	C4A
XP_005213573.2	complement component C8 gamma chain isoform X1 [Bos taurus]	C8G
NP_001030441.1	complement component C9 precursor [Bos taurus]	C9
NP_776425.1	calreticulin precursor [Bos taurus]	CALR
NP_001099082.1	calnexin precursor [Bos taurus]	CANX
NP_001028781.1	T-complex protein 1 subunit theta [Bos taurus]	CCT8
DAA29220.1	TPA: CD9 antigen, partial [Bos taurus]	CD9
NP_001033185.1	complement factor I precursor [Bos taurus]	CFI
NP_783630.2	conglutinin precursor [Bos taurus] CGN1	CGN1
NP_001015515.1	chitinase domain-containing protein 1 precursor [Bos taurus]	CHID1
XP_002687704.3	cytoskeleton-associated protein 4 [Bos taurus]	CKAP4
NP_776448.1	clathrin heavy chain 1 [Bos taurus]	CLTC
NP_001137337.1	collagen alpha-1 chain precursor [Bos taurus]	COL6A1
NP_001092425.1	procollagen galactosyltransferase 1 precursor [Bos taurus]	COLGALT1
XP_024840765.1	ceruloplasmin isoform X1 [Bos taurus]	CP
NP_001159993.1	cathepsin D precursor [Bos taurus]	CTSD
NP_001096720.1	NADH-cytochrome b5 reductase 3 [Bos taurus]	CYB5R3
NP_001029776.1	dolichyl-diphosphooligosaccharide--protein glycosyltransferase subunit 2 precursor [Bos	RPN2
NP_001076074.1	dolichyl-diphosphooligosaccharide--protein glycosyltransferase subunit 1 precursor [Bos	RPN1
NP_001094543.1	dolichyl-diphosphooligosaccharide--protein glycosyltransferase 48 kDa subunit precursoı	DDOST
XP_024830888.1	cytoplasmic dynein 1 intermediate chain 2 isoform X1 [Bos taurus]	DYNC1I2
XP_015315502.1	endothelin-converting enzyme 1 isoform X1 [Bos taurus]	ECE1
AAI05316.1	EEF1A1 protein [Bos taurus]	EEF1A2
ABF57407.1	eukaryotic translation elongation factor 1 gamma, partial [Bos taurus]	EEF1G
NP_001068589.1	elongation factor 2 [Bos taurus]	EEF2
NP_001069922.1	ELAV-like protein 1 [Bos taurus]	ELAVL1
NP_001035616.1	complement factor B precursor [Bos taurus]	ERCC3
NP_001157504.1	erlin-1 [Bos taurus]	ERLIN1
NP_001069739.1	endoplasmic reticulum resident protein 29 precursor [Bos taurus]	ERP29
NP_001030204.1	endoplasmic reticulum resident protein 44 precursor [Bos taurus]	ERP44
AAI05202.1	Coagulation factor II [Bos taurus]	F2
AAI42073.1	Fibrinogen alpha chain [Bos taurus]	FGA
NP_001136389.1	fibrinogen beta chain precursor [Bos taurus]	FGB
XP_005217490.1	fibrinogen gamma-B chain isoform X1 [Bos taurus]	FGG
DAA29904.1	TPA: FK506 binding protein 11 precursor [Bos taurus]	FKBP11
XP_005226989.1	neutral alpha-glucosidase AB isoform X1 [Bos taurus]	GANAB
AAB47507.1	glyceraldehyde-phosphate-dehydrogenase [Bos taurus]	GAPDH
NP_001029206.1	glyceraldehyde-3-phosphate dehydrogenase [Bos taurus]	GAPDH
NP_776493.1	gap junction alpha-1 protein [Bos taurus]	GJA1
NP_776747.1	guanine nucleotide-binding protein subunit alpha-11 [Bos taurus]	GNA11
NP_001039680.1	vesicle transport protein GOT1B [Bos taurus]	GOLT1B

NP_777231.1	aspartate aminotransferase, mitochondrial [Bos taurus]	GOT2
AAI49309.1	Glutathione peroxidase 1 [Bos taurus]	GPX1
NP_001039553.1	probable glutathione peroxidase 8 [Bos taurus]	GPX8
NP_001029799.1	gelsolin isoform b [Bos taurus]	GSN
NP_001071617.1	glutathione S-transferase A1 [Bos taurus]	GSTA1
NP_803481.1	glutathione S-transferase A2 [Bos taurus]	GSTA2
DAA16542.1	TPA: glutathione S-transferase alpha 5 [Bos taurus]	GSTA5
XP_014332511.1	PREDICTED: histone H2A.V [Bos mutus]	H2AFV
NP_001070890.2	hemoglobin subunit alpha [Bos taurus]	HBA2
NP_776342.1	hemoglobin subunit beta [Bos taurus]	HBB
NP_001094536.1	histone H1.3 [Bos taurus]	HIST1H1D
NP_001069476.1	heterogeneous nuclear ribonucleoprotein H2 [Bos taurus]	HNRNPH2
NP_001029784.1	hemopexin precursor [Bos taurus]	HPX
NP_001095835.1	estradiol 17-beta-dehydrogenase 1 [Bos taurus]	HSD17B1
NP_776768.1	3 beta-hydroxysteroid dehydrogenase/Delta 5-->4-isomerase [Bos taurus]	HSD3B2
BAK63243.1	heat shock protein HSP 90-alpha [Pan troglodytes]	HSP90AA1
NP_001073105.1	heat shock protein HSP 90-beta [Bos taurus]	HSP90AB1
NP_777125.1	endoplasmin precursor [Bos taurus]	HSP90B1
NP_001068616.1	endoplasmic reticulum chaperone BiP precursor [Bos taurus]	HSPA5
XP_002685896	heat shock 70 kDa protein 6 [Bos taurus]	HSPA6
NP_776770.2	heat shock cognate 71 kDa protein [Bos taurus]	HSPA8
NP_001020740.1	heat shock protein beta-1 [Bos taurus]	HSPB1
NP_001106791.1	immediate early response 3-interacting protein 1 precursor [Bos taurus]	IER3IP1
AAB37380.1	IgG2a heavy chain constant region, partial [Bos taurus]	IGHD
AAC48762.1	IgG3 heavy chain constant region, partial [Bos taurus]	IGHG3
AQT27056.1	immunoglobulin gamma heavy chain [Bos taurus]	nd
AAB37381.2	IgG1 heavy chain constant region, partial [Bos taurus]	nd
AAP55674.1	immunoglobulin heavy chain constant region, partial [Bos taurus]	IGHM
AEM45004.1	immunoglobulin kappa light chain constant region, partial [Bos taurus]	IGKC
AAA68997.1	anti-idiotype Ig lambda chain V region, partial [Bos taurus]	IGL
AEM05849.1	immunoglobulin lambda light chain constant region 3 allotypic variant IGLC3c, partial [Bo	IGLC3
NP_001077269.1	immunoglobulin lambda-like polypeptide 1 precursor [Bos taurus]	IGLL1
XP_024844915.1	immunoglobulin lambda-1 light chain-like [Bos taurus]	IGLL1
NP_001159971.1	integrin alpha-2 precursor [Bos taurus]	ITGA2
AAG38595.1	integrin alpha-V subunit, partial [Bos taurus]	ITGAV
NP_001103451.1	integrin alpha-6 precursor [Bos taurus]	ITGA6
ABH07895.1	integrin beta 1 [Bos taurus]	ITGB1
NP_001015590.2	inter-alpha-trypsin inhibitor heavy chain H4 precursor [Bos taurus]	ITIH4
NP_001029742.1	lysosome-associated membrane glycoprotein 2 isoform 2 precursor [Bos taurus]	LAMP2
NP_001092413.1	protein ERGIC-53 precursor [Bos taurus]	LMAN1
NP_001094779.1	vesicular integral-membrane protein VIP36 precursor [Bos taurus]	LMAN2
XP_024833611.1	immunoglobulin lambda-1 light chain-like isoform X2 [Bos taurus]	LOC100847119
XP_024833287.1	immunoglobulin lambda-like polypeptide 5 [Bos taurus]	LOC109570963
XP_024844915.1	immunoglobulin lambda-1 light chain-like [Bos taurus]	LOC112441460
XP_002693918.1	zona pellucida sperm-binding protein 3 receptor [Bos taurus]	LOC506707
XP_002693919.1	apolipoprotein R [Bos taurus]	LOC515150
NP_001073694.1	alpha-2-macroglobulin receptor-associated protein precursor [Bos taurus]	LRPAP1
NP_786973.1	cation-dependent mannose-6-phosphate receptor precursor [Bos taurus]	M6PR
XP_005225065.1	malate dehydrogenase, mitochondrial isoform X1 [Bos taurus]	MDH2
CAA72406.1	bP47 protein, partial [Bos taurus]	MFGE8
NP_001092453.1	malectin precursor [Bos taurus]	MLEC

NP_001068647.1	mannose-P-dolichol utilization defect 1 protein [Bos taurus]	MPDU1
DAA18323.1	TPA: mannose receptor, C type 2 [Bos taurus]	MRC2
NP_777140.1	60S ribosomal protein L3 [Bos taurus]	MRPL3
XP_024841355.1	myoferlin isoform X1 [Bos taurus]	MYOF
CAH59718.2	alpha-1-acid glycoprotein precursor [Bos taurus]	ORM1
NP_001015565.1	poly-binding protein 1 [Bos taurus]	PCBP1
NP_776758.2	protein disulfide-isomerase A3 precursor [Bos taurus]	PDIA3
NP_001039344.1	protein disulfide-isomerase A4 precursor [Bos taurus]	PDIA4
NP_001193274.1	protein disulfide-isomerase A6 precursor [Bos taurus]	PDIA6
NP_001137210.1	6-phosphogluconate dehydrogenase, decarboxylating [Bos taurus]	PGD
NP_001029471.1	phosphoglycerate kinase 1 [Bos taurus]	PGK1
XP_005887269.1	PREDICTED: membrane-associated progesterone receptor component 1 isoform X1 [Bos	PGRMC1
XP_007121455.1	LOW QUALITY PROTEIN: membrane-associated progesterone receptor component 2 [Phy	PGRMC2
NP_776376.1	plasminogen precursor [Bos taurus]	PLG
XP_005544827.1	PREDICTED: procollagen-lysine,2-oxoglutarate 5-dioxygenase 1 [Macaca fascicularis]	PLOD1
DAA25310.1	TPA: peptidyl-prolyl cis-trans isomerase B [Bos taurus]	PPIB
ELR57459.1	Peroxiredoxin-1, partial [Bos mutus]	PRDX1
XP_005228408.1	peroxiredoxin-4 isoform X1 [Bos taurus]	PRDX4
NP_788835.1	glucosidase 2 subunit beta precursor [Bos taurus]	PRKCSH
NP_001095953.1	ribose-phosphate pyrophosphokinase 3 [Bos taurus]	PRPS1L1
NP_001107199.1	cationic trypsin precursor [Bos taurus]	PRSS1
Q29463.1	PRSS2 protein [Bos taurus] anionic tryprisn precursor	PRSS2
NP_001030387.1	proteasome subunit alpha type-1 [Bos taurus]	PSMA1
NP_001015566.1	proteasome subunit alpha type-5 [Bos taurus]	PSMA5
NP_001039427.1	proteasome subunit alpha type-6 [Bos taurus]	PSMA6
NP_001039705.1	26S proteasome regulatory subunit 10B [Bos taurus]	PSMC6
NP_001094667.1	26S proteasome non-ATPase regulatory subunit 2 [Bos taurus]	PSMD2
XP_010801437.1	prostaglandin F2 receptor negative regulator [Bos taurus]	PTGFRN
NP_001033251.1	ras-related protein Rab-11A [Bos taurus]	RAB11A
NM_001130754.1	Bos taurus RAB14, member RAS oncogene family , mRNA	RAB14
NP_001069701.1	ras-related protein Rab-1B [Bos taurus]	RAB1A
NP_001068822.1	ras-related protein Rab-2A [Bos taurus]	RAB2A
XP_024834273	Ras-related protein Rab-4B isoform X1 [Bos taurus]	RAB4B
NP_001180044.1	ras-related protein Rab-6A [Bos taurus]	RAB6A
XP_005223227.1	ras-related protein Rab-7a isoform X1 [Bos taurus]	RAB7A
XP_024840414.1	ras-related C3 botulinum toxin substrate 1 isoform X1 [Bos taurus]	RAC1
DAA27596.1	TPA: guanine nucleotide-binding protein subunit beta-2-like 1 [Bos taurus] GNB2L1	RACK1
NP_001029877.1	GTP-binding nuclear protein Ran [Bos taurus]	RAN
AAB28336.1	retinol-binding protein, partial [Bos taurus]	RBP4
AAX46329.1	ribosomal protein L5 [Bos taurus]	RPL5
NP_001014928.1	60S ribosomal protein L7 [Bos taurus]	RPL7
AAB65436.1	acidic ribosomal phosphoprotein PO, partial [Bos taurus]	RPLP0
EAW50256.1	hCG1994130, isoform CRA_b [Homo sapiens]	RPS15A
DAA30928.1	PA: ribosomal protein S16-like [Bos taurus]	RPS16
NP_001028785.1	40S ribosomal protein S2 [Bos taurus]	RPS2
NP_001029219.1	40S ribosomal protein S3 [Bos taurus]	RPS3
NP_001030522.1	40S ribosomal protein S4 [Bos taurus]	RPS4Y1
NP_001015531.1	40S ribosomal protein S5 [Bos taurus]	RPS5
NP_001092344.1	40S ribosomal protein S7 [Bos taurus]	RPS7
XP_005222426.1	40S ribosomal protein SA isoform X1 [Bos taurus]	RPSA
NP_001107197.1	protein S100-A8 [Bos taurus]	S100A8

NP_001069811.1	vesicle-trafficking protein SEC22b precursor [Bos taurus]	SEC22B
NP_001139773.1	serpin A3-2 precursor [Bos taurus]	SERPINA3
XP_005222351.1	serpin A3-5 [Bos taurus]	SERPINA3
NP_777094.1	glia-derived nexin precursor [Bos taurus]	SERPINE2
NP_001039528.1	serpin H1 precursor [Bos taurus]	SERPINH1
NP_001193220.1	splicing factor, proline- and glutamine-rich [Bos taurus]	SFPQ
NP_001076867.1	serine/arginine-rich splicing factor 9 [Bos taurus]	SFRS9
NP_001094664.1	calcium-binding mitochondrial carrier protein Aralar1 [Bos taurus]	SLC25A12
ABG67048.1	solute carrier family 29 , member 1, partial [Bos taurus]	SLC29A1
NP_001069198.1	receptor for retinol uptake STRA6 [Bos taurus]	STRA6
NP_803450.2	serotransferrin precursor [Bos taurus]	TF
NP_001035639.1	transmembrane emp24 domain-containing protein 10 precursor [Bos taurus]	TMED10
AAX09080.1	transmembrane emp24 protein transport domain containing 9 [Bos taurus]	TMED9
NP_001095950.1	transmembrane protein 43 [Bos taurus]	TMEM43
NP_001098966.1	transmembrane emp24 domain-containing protein 7 precursor [Bos taurus]	TMED7
NP_001039935.1	transmembrane protein 109 precursor [Bos taurus]	TMEM109
NP_001029376.1	tubulin alpha-1C chain [Bos taurus]	TUBA1C
XP_002697652.1	tubulin beta-2A chain isoform X1 [Bos taurus]	TUBB2A
XP_002685277.1	UDP-glucose:glycoprotein glucosyltransferase 1 isoform X1 [Bos taurus]	UGGT1
NP_001029466.1	transitional endoplasmic reticulum ATPase [Bos taurus]	VCP
XP_005209392.1	voltage-dependent anion-selective channel protein 1 isoform X1 [Bos taurus]	VDAC1
NP_776911.2	voltage-dependent anion-selective channel protein 2 [Bos taurus]	VDAC2
NP_777154.1	voltage-dependent anion-selective channel protein 3 [Bos taurus]	VDAC3
AAA53661.1	vimentin [Bos taurus]	VIM
AAA83135.1	immunoglobulin lambda light chain variable region [Bos taurus]	Vl1b
XP_024851955.1	vacuolar protein sorting-associated protein 13A isoform X1 [Bos taurus]	VPS13A
NP_001030222.1	vitronectin precursor [Bos taurus]	VTN
NP_001071595.1	14-3-3 protein theta [Bos taurus]	YWHAQ
AAB66575.1	immunoglobulin light chain variable region [Bos taurus]	nd
AAB66566.1	immunoglobulin light chain variable region [Bos taurus]	nd

GC dominant proteins

Bt acc	**Description (GC specific proteins)**	Official symbol
NP_001029544.1	protein ABHD11 [Bos taurus]	ABHD11
NP_001068703.1	medium-chain specific acyl-CoA dehydrogenase, mitochondrial precursor [Bos taurus]	ACADM
AAI51812.1	short-chain specific acyl-CoA dehydrogenase, mitochondrial precursor [Bos taurus] (DAA2	ACADS
NP_776919.1	very long-chain specific acyl-CoA dehydrogenase, mitochondrial precursor [Bos taurus] (N	ACADVL
NP_001039540.1	acetyl-CoA acetyltransferase, mitochondrial precursor [Bos taurus] (NP_001039540.1)	ACAT1
AAI02907.1	Peroxisomal D3,D2-enoyl-CoA isomerase [Bos taurus]	ACBP
AAI02643.1	Aconitase 2, mitochondrial [Bos taurus]	ACO2
NP_776404.2	actin, cytoplasmic 1 [Bos taurus]	ACTB
NP_001091521.1	alpha-actinin-4 [Bos taurus]	ACTN4
NP_001180177.1	alpha-centractin [Bos taurus]	ACTR1A
NP_001069534.1	adenosine kinase [Bos taurus]	ADK
NP_776314.1	adenylate kinase 2, mitochondrial [Bos taurus]	AK2
NP_001107554.1	angiotensinogen precursor [Bos taurus]	AGT
NP_001029487.1	adenosylhomocysteinase [Bos taurus]	AHCY
NP_776409.1	alpha-2-HS-glycoprotein precursor [Bos taurus]	AHSG
NP_776314.1	adenylate kinase 2, mitochondrial [Bos taurus] (NP_776314.1)	AK2
NP_001069981.1	alcohol dehydrogenase [NADP(+)] [Bos taurus] (NP_001069981.1)	AKR1A1
DAA32096.1	TPA: aldo-keto reductase family 7, member A2, partial [Bos taurus]	AKR7A2

AAI12597.1	Aminolevulinate, delta-, dehydratase [Bos taurus]	ALAD
NP_001014957.1	pyrroline-5-carboxylate reductase 1, mitochondrial [Bos taurus] (NP_001014957.1)	ALDH4A1
NP_001095385.1	fructose-bisphosphate aldolase A [Bos taurus] (NP_001095385.1)	ALDOA
NP_001091453.1	fructose-bisphosphate aldolase C [Bos taurus]	ALDOC
NP_786978.2	annexin A1 [Bos taurus] (NP_786978.2)	ANXA1
NP_788782.2	DNA-(apurinic or apyrimidinic site) lyase [Bos taurus]	APEX1
NP_001020505.1	adenine phosphoribosyltransferase [Bos taurus]	APRT
NP_001181942.1	coatomer subunit delta [Bos taurus]	ARCN1
NP_788823.1	rho GDP-dissociation inhibitor 1 [Bos taurus]	ARHGDIA
DAA30669.1	TPA: armadillo repeat containing 10 isoform 2 [Bos taurus]	ARMC10
NP_001068927.1	acid ceramidase precursor [Bos taurus]	ASAH1
NP_001192510.1	ATPase family AAA domain-containing protein 1 [Bos taurus]	ATAD1
NP_001098932.1	ATPase family AAA domain-containing protein 3 [Bos taurus]	ATAD3A
AAW29825.1	Atp2a2, partial [Bos taurus]	ATP2A2
NP_777109.1	ATP synthase subunit alpha, mitochondrial precursor [Bos taurus] (NP_777109.1)	ATP5F1A
NP_786990.1	ATP synthase subunit beta, mitochondrial precursor [Bos taurus] (NP_786990.1)	ATP5F1B
XP_005214178.1	ATP synthase subunit gamma, mitochondrial isoform X1 [Bos taurus]	ATP5F1C
NP_001033590.1	ATP synthase F(0) complex subunit B1, mitochondrial precursor [Bos taurus] (NP_001033	ATP5PB
NP_777149.1	ATP synthase subunit d, mitochondrial [Bos taurus]	ATP5PD
NP_776669.1	ATP synthase subunit O, mitochondrial precursor [Bos taurus] (NP_776669.1)	ATP5PO
ABG67061.1	branched chain aminotransferase 2, mitochondrial, partial [Bos taurus]	BCAT2
NP_001039383.1	valacyclovir hydrolase [Bos taurus]	BPHL
NP_001069645.1	mitotic checkpoint protein BUB3 [Bos taurus]	BUB3
NP_001179134.1	carbonic anhydrase 14 precursor [Bos taurus]	CA14
NP_848667.1	carbonic anhydrase 2 [Bos taurus]	CA2
NP_001029981.1	calcyclin-binding protein [Bos taurus]	CACYBP
XP_005204713.1	adenylyl cyclase-associated protein 1 isoform X2 [Bos taurus] (XP_005204713.1)	CAP1
NP_001076949.1	F-actin-capping protein subunit alpha-1 [Bos taurus]	CAPZA1
NP_776857.1	thioredoxin-dependent peroxide reductase, mitochondrial precursor [Bos taurus]	CAT
NP_001029411.1	T-complex protein 1 subunit beta [Bos taurus] (NP_001029411.1)	CCT2
NP_001033283.1	T-complex protein 1 subunit delta [Bos taurus]	CCT4
NP_001029767.2	T-complex protein 1 subunit epsilon [Bos taurus]	CCT5
AAI12597.1	T-complex protein 1 subunit zeta [Bos taurus] (DAA15399.1)	CCT6A
NP_001039636.1	T-complex protein 1 subunit eta [Bos taurus]	CCT7
ACJ06401.1	CDC42 protein [Bos taurus]	CDC42
XP_015330796.2	complement factor H-related protein 5 isoform X2 [Bos taurus]	CFHR5
NP_001030552.1	MICOS complex subunit MIC19 [Bos taurus]	CHCHD3
NP_001015608.1	chloride intracellular channel protein 1 [Bos taurus] (NP_001015608.1)	CLIC1
NP_001179912.1	carboxymethylenebutenolidase homolog [Bos taurus]	CMBL
NP_001179912.1	carboxymethylenebutenolidase homolog [Bos taurus] (NP_001179912.1)	CMBL
NP_001029425.1	protein canopy homolog 4 precursor [Bos taurus]	CNPY4
DAA21833.1	TPA: COMM domain-containing protein 9 [Bos taurus]	COMMD9
AAI09964.1	Coatomer protein complex, subunit epsilon [Bos taurus]	COPE
NP_001092374.1	COP9 signalosome complex subunit 3 [Bos taurus]	COPS3
AAA31644.1	cytochrome oxidase subunit II (mitochondrion) [Bos taurus]	COX2
NP_001137569.1	C-reactive protein precursor [Bos taurus]	CRP
XP_010803417.1	citrate synthase, mitochondrial isoform X1 [Bos taurus] (XP_010803417.1)	CS
NP_001179711.2	chondroitin sulfate proteoglycan 4 precursor [Bos taurus]	CSPG4
NP_001071303.1	cathepsin Z precursor [Bos taurus]	CTSZ
NP_001033179.1	cytochrome c1, heme protein, mitochondrial [Bos taurus]	CYC1
NP_788817.1	cholesterol side-chain cleavage enzyme, mitochondrial precursor [Bos taurus]	CYP11A1

DAA14766.1	TPA: steroid 17-alpha-hydroxylase/17,20 lyase [Bos taurus]	CYP17A1
AAI42211.1	Dicarbonyl/L-xylulose reductase [Bos taurus]	DCXR
NP_001029876.1	N(G),N(G)-dimethylarginine dimethylaminohydrolase 2 [Bos taurus]	DDAH2
NP_001028801.1	Cluster of spliceosome RNA helicase DDX39B [Bos taurus] (NP_001028801.1)	DDX39B
NP_001030420.1	dehydrogenase/reductase SDR family member 7B [Bos taurus]	DHRS7B
NP_001039347.1	diablo homolog, mitochondrial [Bos taurus]	DIABLO
NP_001193099.1	dihydrolipoyl dehydrogenase, mitochondrial [Bos taurus]	DLD
NP_001068750.1	dihydrolipoyllysine-residue succinyltransferase component of 2-oxoglutarate dehydroger	DLST
DAA33780.1	TPA: dipeptidyl peptidase 7, partial [Bos taurus]	DPP7
NP_001069468.1	dihydropyrimidinase-related protein 2 [Bos taurus]	DPYSL2
AAI02085.1	ECH1 protein, partial [Bos taurus]	ECH1
AAL09921.1	enoyl-CoA hydratase precursor, partial [Bos taurus]	ECH1
NP_001020377.2	enoyl-CoA hydratase, mitochondrial precursor [Bos taurus]	ECHS1
AAI18191.1	3,2-trans-enoyl-CoA isomerase, mitochondrial [Bos taurus] (DAA15604.1)	ECI1
NP_787007.1	eukaryotic translation initiation factor 2 subunit 1 [Bos taurus]	EIF2S1
NP_001073796.1	ER membrane protein complex subunit 2 [Bos taurus]	EMC2
NP_787017.1	endonuclease G, mitochondrial precursor [Bos taurus]	ENDOG
AAD33073.1	alpha enolase [Bos taurus] (AAD33073.1)	ENO1
NP_001095758.1	mammalian ependymin-related protein 1 precursor [Bos taurus]	EPDR1
AAI46272.1	electron transfer flavoprotein subunit alpha, mitochondrial precursor [Bos taurus]	ETFA
NP_001033671.1	electron transfer flavoprotein subunit beta [Bos taurus]	ETFB
NP_001069777.1	protein FAM3A [Bos taurus]	FAM3A
NP_001092617.1	protein FAM3C precursor [Bos taurus]	FAM3C
NP_001094577.1	phenylalanine--tRNA ligase alpha subunit [Bos taurus]	FARSA
AAI18191.1	FDXR protein [Bos taurus] (AAI18191.1)	FDXR
NP_001069271.1	fumarate hydratase, mitochondrial [Bos taurus] (NP_001069271.1)	FH
NP_001033201.1	Cluster of peptidyl-prolyl cis-trans isomerase FKBP3 [Bos taurus] (NP_001033201.1)	FKBP3
DAA29159.1	TPA: peptidyl-prolyl cis-trans isomerase FKBP4 [Bos taurus]	FKBP4
NP_001193443.1	filamin-A [Bos taurus] (NP_001193443.1)	FLNA
NP_001039500.1	tissue alpha-L-fucosidase precursor [Bos taurus]	FUCA1
AAW81980.1	glucose-6-phosphate dehydrogenase, partial [Bos taurus]	G6PD
ABF57297.1	galactokinase 1, partial [Bos taurus]	GALK1
AAT39889.1	Cluster of l-arginine:glycine amidinotransferase, partial [Bos taurus] (AAT39889.1)	GATM
NP_001028934.1	rab GDP dissociation inhibitor beta [Bos taurus] (NP_001028934.1)	GDI2
NP_001030215.1	beta-galactosidase precursor [Bos taurus]	GLB1
P62871	G protein beta 1 subunit [Rattus norvegicus]	GNB1
NP_001035561.1	glucose-6-phosphate isomerase [Bos taurus]	GPI
NP_001094583.1	glutathione peroxidase 7 precursor [Bos taurus] (NP_001094583.1)	GPX7
NP_787019.1	glutathione S-transferase Mu 1 [Bos taurus]	GSTM1
AAX46392.1	glutathione S-transferase mu 3, partial [Bos taurus]	GSTM3
NP_803482.1	glutathione S-transferase P [Bos taurus]	GSTP1
NP_776759.1	3-hydroxyacyl-CoA dehydrogenase type-2 [Bos taurus] (NP_776759.1)	HSD17B10
NP_001039799.1	hydroxyacyl-coenzyme A dehydrogenase, mitochondrial [Bos taurus]	HADH
NP_776761.1	trifunctional enzyme subunit beta, mitochondrial precursor [Bos taurus]	HADHB
NP_001069453.1	heme-binding protein 1 [Bos taurus]	HEBP1
NP_001068632.1	beta-hexosaminidase subunit alpha precursor [Bos taurus]	HEXA
NP_001039571.1	3-hydroxyisobutyrate dehydrogenase, mitochondrial precursor [Bos taurus] (NP_001039!	HIBADH
DAA32869.1	Cluster of TPA: 3-hydroxyisobutyryl-CoA hydrolase, mitochondrial precursor, partial [Bos	HIBCH
NP_788785.1	high mobility group protein B1 [Bos taurus]	HMGB1
NP_001032705.1	high mobility group protein B2 [Bos taurus]	HMGB2
NP_001029734.1	heterogeneous nuclear ribonucleoprotein K [Bos taurus] (NP_001029734.1)	HNRNPK

AAG09236.1	hypoxanthine phosphoribosyltransferase, partial [Bos taurus]	HPRT
NP_001094777.1	very-long-chain 3-oxoacyl-CoA reductase [Bos taurus]	HSD17B12
AAI22585.1	Hydroxysteroid (17-beta) dehydrogenase 4 [Bos taurus]	HSD17B4
NP_001029696.1	stress-70 protein, mitochondrial precursor [Bos taurus]	HSPA9
NP_001160080.1	60 kDa heat shock protein, mitochondrial [Bos taurus] (NP_001160080.1)	HSPD1
NP_001193839.1	Cluster of hypoxia up-regulated protein 1 precursor [Bos taurus] (NP_001193839.1)	HYOU1
NP_851355.2	isocitrate dehydrogenase [NADP] cytoplasmic [Bos taurus]	IDH1
NP_786984.1	isocitrate dehydrogenase [NADP], mitochondrial precursor [Bos taurus] (NP_786984.1)	IDH2
NP_777069.1	isocitrate dehydrogenase [NAD] subunit alpha, mitochondrial precursor [Bos taurus]	IDH3A
AAI04503.1	IDH3B protein, partial [Bos taurus]	IDH3B
AAX46424.1	isocitrate dehydrogenase 3 (NAD+) gamma isoform a precursor [Bos taurus]	IDH3G
NP_001033276.1	interleukin enhancer-binding factor 2 [Bos taurus]	ILF2
NP_776519.2	inhibin alpha chain precursor [Bos taurus]	INHA
NP_001180082.1	importin subunit beta-1 [Bos taurus]	KPNB1
NP_776525.2	L-lactate dehydrogenase B chain isoform LDHB [Bos taurus]	LDHB
NP_001039642.1	leucine-rich alpha-2-glycoprotein precursor [Bos taurus]	LRG1
NP_001029750.1	leucine-rich repeat-containing protein 59 [Bos taurus]	LRRC59
NP_001029860.1	acyl-protein thioesterase 1 [Bos taurus]	LYPLA1
NP_001029800.1	malate dehydrogenase, cytoplasmic isoform MDH1 [Bos taurus]	MDH1
NP_001039544.1	protein HP-25 homolog 2 precursor [Bos taurus]	MGC137211
BAA95942.1	mitochondrial carrier homolog 2 [Bos taurus]	MTCH2
NP_001014916.1	nascent polypeptide-associated complex subunit alpha [Bos taurus]	NACA
NP_001039814.1	alpha-N-acetylgalactosaminidase precursor [Bos taurus]	NAGA
ABF57357.1	NADH dehydrogenase (ubiquinone) 1 alpha subcomplex, 9, 39kDa, partial [Bos taurus]	NDUFA1
NP_788828.1	NADH dehydrogenase [ubiquinone] 1 alpha subcomplex subunit 10, mitochondrial precu	NDUFA10
NP_991386.1	NADH dehydrogenase [ubiquinone] 1 alpha subcomplex subunit 9, mitochondrial precurs	NDUFA9
NP_786977.1	NADH dehydrogenase [ubiquinone] 1 beta subcomplex subunit 9 [Bos taurus]	NDUFB9
AAL09921.1	NADH dehydrogenase [ubiquinone] iron-sulfur protein 3, mitochondrial precursor [Bos ta	NDUFS3
AAI09907.1	Cluster of NADH dehydrogenase (ubiquinone) Fe-S protein 8, 23kDa (NADH-coenzyme Q	NDUFS8
AAI05480.1	NADH dehydrogenase 24 kDa subunit (AA 6-217), partial [Bos taurus]	NDUFV2
NP_001069824.1	protein NipSnap homolog 1 [Bos taurus]	NIPSNAP1
NP_001070479.1	protein NipSnap homolog 2 [Bos taurus]	NIPSNAP2
NP_001073767.1	protein NipSnap homolog 3A [Bos taurus]	NIPSNAP3A
AAA21440.1	nicotinamide nucleotide transhydrogenase, partial [Bos taurus] (AAA21440.1)	NNT
XP_002698082.1	nodal modulator 1 [Bos taurus]	NOMO1
NP_001040019.1	non-POU domain-containing octamer-binding protein [Bos taurus]	NONO
NP_777183.1	NPC intracellular cholesterol transporter 1 precursor [Bos taurus]	NPC1
NP_001029412.1	ornithine aminotransferase, mitochondrial precursor [Bos taurus] (NP_001029412.1)	OAT
ABG67106.1	GTP-binding protein PTD004 [Bos taurus]	OLA1
NP_001029891.1	ubiquitin thioesterase OTUB1 [Bos taurus]	OTUB1
NP_001069538.1	succinyl-CoA:3-ketoacid coenzyme A transferase 1, mitochondrial [Bos taurus]	OXCT1
XP_005226443.1	prolyl 4-hydroxylase subunit alpha-1 isoform X1 [Bos taurus]	P4HA1
AAI46272.1	P4HB protein [Bos taurus] (AAI46272.1)	P4HB
NP_777089.1	platelet-activating factor acetylhydrolase IB subunit beta [Bos taurus]	PAFAH1B2
NP_777090.1	platelet-activating factor acetylhydrolase IB subunit gamma [Bos taurus]	PAFAH1B3
AAR05660.1	phosphoribosylaminoimidazole carboxylase [Bos taurus]	PAICS
NP_001015572.1	protein/nucleic acid deglycase DJ-1 [Bos taurus] (NP_001015572.1)	PARK7
XP_005227045	pyruvate carboxylase, mitochondrial isoform X2 [Bos taurus]	PC
NP_001033637.1	propionyl-CoA carboxylase beta chain, mitochondrial precursor [Bos taurus]	PCCB
NP_001029666.1	proliferating cell nuclear antigen [Bos taurus]	PCNA
NP_001098944.1	prenylcysteine oxidase 1 precursor [Bos taurus]	PCYOX1

NP_001069219.1	pyruvate dehydrogenase protein X component precursor [Bos taurus]	PDHX
NP_001028795.1	phosphatidylethanolamine-binding protein 1 [Bos taurus]	PEBP1
NP_001029226.1	phosphoglycerate mutase 1 [Bos taurus]	PGAM1
DAA28196.1	TPA: 6-phosphogluconolactonase [Bos taurus]	PGLS
NP_001029744.1	prohibitin [Bos taurus]	PHB
NP_001039663.1	prohibitin-2 [Bos taurus]	PHB2
AAI05480.1	Phosphoglycerate dehydrogenase [Bos taurus]	PHGDH
NP_001071348.1	GPI-anchor transamidase precursor [Bos taurus]	PIGK
NP_001094619.1	procollagen-lysine,2-oxoglutarate 5-dioxygenase 2 precursor [Bos taurus]	PLOD2
AAX46392.1	purine nucleoside phosphorylase [Bos taurus] (AAX46392.1)	PNP
AAQ02334.1	protein O-fucosyltransferase 2b [Bos taurus]	POFUT2
NP_001014903.1	protein O-glucosyltransferase 1 precursor [Bos taurus]	POGLUT1
NP_001069864.1	inorganic pyrophosphatase 2, mitochondrial [Bos taurus]	PPA2
XP_005207716.1	inorganic pyrophosphatase 2, mitochondrial isoform X1 [Bos taurus]	PPA2
NP_001070378.1	peptidyl-prolyl cis-trans isomerase C precursor [Bos taurus]	PPIC
DAA24418.1	TPA: serine/threonine-protein phosphatase PP1-beta catalytic subunit, partial [Bos tauru	PPP1CB
NP_001029410.1	protein phosphatase 1 regulatory subunit 7 [Bos taurus]	PPP1R7
DAA30952.1	TPA: palmitoyl-protein thioesterase 1 precursor [Bos taurus]	PPT2
NP_777188.1	peroxiredoxin-2 [Bos taurus]	PRDX2
NP_777068.1	peroxiredoxin-6 [Bos taurus]	PRDX6
AAB20007.1	F1-ATPase beta subunit=H(+)-transporting ATPase beta subunit {EC 3.6.1.34} [cattle, hear	PRK09280
NP_001029834.1	proteasome alpha 2 subunit-like [Bos taurus]	PSMA2
NP_001029407.1	proteasome subunit alpha type-3 [Bos taurus]	PSMA3
NP_001029553.1	proteasome subunit alpha type-4 [Bos taurus]	PSMA4
AAI51812.1	PSMA7 protein, partial [Bos taurus] (AAI51812.1)	PSMA7
NP_001033628.1	proteasome subunit beta type-1 precursor [Bos taurus]	PSMB1
NP_001015615.1	Cluster of proteasome subunit beta type-2 [Bos taurus] (NP_001015615.1)	PSMB2
NP_001029438.1	proteasome subunit beta type-4 [Bos taurus]	PSMB4
NP_001032701.1	proteasome subunit beta type-5 [Bos taurus] (NP_001032701.1)	PSMB5
DAA26442.1	TPA: 26S proteasome non-ATPase regulatory subunit 5 [Bos taurus]	PSMD5
ABM21562.1	proteasome 26S non-ATPase subunit 7, partial [Bos taurus]	PSMD7
NP_001019680.2	proteasome activator complex subunit 1 [Bos taurus]	PSME1
NP_001014889.1	proteasome activator complex subunit 2 [Bos taurus]	PSME2
NP_001069815.1	proteasome assembly chaperone 1 [Bos taurus]	PSMG1
NP_001289713.1	polypyrimidine tract-binding protein 1 isoform 1 [Bos taurus] (NP_001289713.1)	PTBP1
NP_001030375.1	pentatricopeptide repeat-containing protein 2, mitochondrial [Bos taurus]	PTCD2
AAR05660.1	prostaglandin reductase 1 [Bos taurus]	PTGR1
NP_001192656.1	pyruvate kinase PKM [Bos taurus] (NP_001192656.1)	PYCR1
NP_001069960.1	dihydropteridine reductase [Bos taurus]	QDPR
NP_001029915.1	ras-related protein Rab-5C [Bos taurus]	RAB5B
NP_787018.1	ras-related protein Rap-1b precursor [Bos taurus]	RAP1B
NP_001030396.1	ribonuclease inhibitor [Bos taurus]	RNH1
XP_024856794.1	ribosome-binding protein 1 isoform X1 [Bos taurus] (XP_024856794.1)	RRBP1
NP_001075180.1	SUMO-activating enzyme subunit 1 [Bos taurus]	SAE1
NP_001029693.1	GTP-binding protein SAR1a [Bos taurus]	SAR1A
NP_001029460.1	saccharopine dehydrogenase-like oxidoreductase [Bos taurus]	SCCPDH
NP_001039374.1	retinoid-inducible serine carboxypeptidase precursor [Bos taurus]	SCPEP1
NP_001075916.1	septin-11 [Bos taurus]	SEPTIN11
NP_001039557.1	septin-2 [Bos taurus]	SEPTIN2
NP_776307.1	alpha-1-antiproteinase precursor [Bos taurus]	SERPINA1
AAR26722.1	endopin 2B [Bos taurus]	SERPINA3-7

NP_776565.1	pigment epithelium-derived factor precursor [Bos taurus]	SERPINF1
XP_010819218.1	LOW QUALITY PROTEIN: protein SET-like [Bos taurus]	SET
NP_001033119.1	small glutamine-rich tetratricopeptide repeat-containing protein alpha [Bos taurus]	SGTA
NP_001029454.1	serine hydroxymethyltransferase, mitochondrial precursor [Bos taurus]	SHMT2
NP_001069276.1	nucleotide exchange factor SIL1 precursor [Bos taurus]	SIL1
XP_003587518.1	mitochondrial dicarboxylate carrier [Bos taurus]	SLC25A10
ABQ12913.1	solute carrier family 25 (mitochondrial carrier; oxoglutarate carrier), member 11, partial [	SLC25A11
NP_777082.1	Cluster of phosphate carrier protein, mitochondrial precursor [Bos taurus] (NP_777082.1	SLC25A3
NP_777085.1	ADP/ATP translocase 3 [Bos taurus] (NP_777085.1)	SLC25A6
NP_991353.1	staphylococcal nuclease domain-containing protein 1 [Bos taurus] (NP_991353.1)	SND1
NP_001092418.1	U2 small nuclear ribonucleoprotein A' [Bos taurus]	SNRPA1
AAA30655.1	manganous superoxide dismutase, partial [Bos taurus]	SOD2
ABI79460.1	sepiapterin reductase isoform 2 [Bos taurus]	SPR
NP_788838.1	lupus La protein homolog [Bos taurus] (NP_788838.1)	SSB
NP_001095396.1	hsc70-interacting protein [Bos taurus]	ST13
NP_991375.1	CMP-N-acetylneuraminate-beta-galactosamide-alpha-2,3-sialyltransferase 4 precursor [B	ST3GAL4
NP_001033157.1	stomatin-like protein 2, mitochondrial [Bos taurus]	STOML2
NP_001030254.1	succinate--CoA ligase [ADP/GDP-forming] subunit alpha, mitochondrial precursor [Bos ta	SUCLG1
NP_001029811.1	succinate--CoA ligase [GDP-forming] subunit beta, mitochondrial precursor [Bos taurus] (	SUCLG2
AAB20668.1	beta 2 glycoprotein I [cattle, Peptide, 326 aa]	SVEP1
NP_001013617.1	transgelin-2 [Bos taurus]	TAGLN2
NP_001030360.2	transaldolase [Bos taurus] (NP_001030360.2)	TALDO1
ABS45051.1	transketolase [Bos taurus]	TKT
NP_001192357.3	talin-1 [Bos taurus]	TLN1
NP_001033652.1	transmembrane emp24 domain-containing protein 1 precursor [Bos taurus]	TMED1
NP_001153290.1	transmembrane emp24 domain-containing protein 2 precursor [Bos taurus]	TMED2
NP_001068906.1	mitochondrial import receptor subunit TOM40 homolog [Bos taurus]	TOMM40
NP_001013607.1	triosephosphate isomerase [Bos taurus] (NP_001013607.1)	TPI1
XP_005208569.1	tropomyosin alpha-4 chain isoform X3 [Bos taurus] (XP_005208569.1)	TPM4
NP_776632.1	Cluster of elongation factor Tu, mitochondrial precursor [Bos taurus] (NP_776632.1)	TUFM
DAA15792.1	CTPA: thioredoxin-like 1 [Bos taurus]	TXNL1
AAA30434.1	cathepsin B, partial [Bos taurus] (AAA30434.1)	TYRP1
NP_001095947.1	ubiquitin-like modifier-activating enzyme 1 [Bos taurus] (NP_001095947.1)	UBA1
NP_001039637.1	ubiquitin carboxyl-terminal hydrolase isozyme L1 [Bos taurus]	UCHL1
AAI04501.1	UQCRC1 protein, partial [Bos taurus]	UQCRC1
NP_777055.1	cytochrome b-c1 complex subunit 2, mitochondrial precursor [Bos taurus]	UQCRC2
NP_001179194.2	synaptic vesicle membrane protein VAT-1 homolog [Bos taurus]	VAT1
NP_001178299.1	vinculin [Bos taurus]	VCL
NP_777219.2	14-3-3 protein beta/alpha [Bos taurus]	YWHAB
NP_776916.1	14-3-3 protein epsilon [Bos taurus]	YWHAE
NP_777218.2	14-3-3 protein gamma [Bos taurus]	YWHAG
NP_776917.2	14-3-3 protein eta [Bos taurus] (NP_776917.2)	YWHAH
NP_777239.1	14-3-3 protein zeta/delta [Bos taurus]	YWHAZ
