# Supplementary material for: Protein Cargo of Extracellular Vesicles From Bovine Follicular Fluid and Analysis of Their Origin From Different Ovarian Cells
Source: Front Vet Sci. 2020 Nov 4;7:584948. doi: 10.3389/fvets.2020.584948 (PMC7672127; doi:10.3389/fvets.2020.584948)
Supplement: Supplementary Table 3 — Gene Ontology terms in ffEV-unique, common ffEV-GC, and GC-unique protein lists. [file Table_3.pdf]

Supplementary data S3.

FunRich analysisi of GO terms (charts) in proteins identified in GC, ff-EV and common to both GC and ff-EV

	% of proteins		
**Cellular component**	**GC**	**GC-EV**	**EV**
Exosomes	55,60165975	70,8333333	52,1008403
Cytoplasm	57,67634855	58,3333333	45,3781513
Cytosol	17,42738589	20,2380952	44,5378151
Nucleolus	14,93775934	23,2142857	42,0168067
Centrosome	21,99170124	20,2380952	37,8151261
Ribosome	1,659751037	8,33333333	34,4537815
Nucleus	39,00414938	41,6666667	30,2521008
Lysosome	61,82572614	63,0952381	27,7310924
Plasma membrane	15,76763485	30,3571429	19,3277311
Extracellular	19,50207469	31,547619	17,6470588
Mitochondrion	42,73858921	33,3333333	16,8067227
Cytosolic large ribosomal subu	0	1,78571429	15,9663866
Endoplasmic reticulum	14,52282158	30,3571429	15,9663866
Golgi aparatus	5,394190871	17,8571429	14,2857143
Cytosolic small ribosomal subu	0	5,35714286	13,4453782
Extracellular region	5,809128631	15,4761905	8,40336134
Membrane	4,979253112	15,4761905	8,40336134
Ribonucleoprotein complex	1,659751037	5,95238095	7,56302521
Cytoskeleton	8,298755187	15,4761905	7,56302521
Endosome	0,829875519	7,14285714	5,88235294
Integral to membrane	2,904564315	9,52380952	5,04201681
Extracellular space	3,319502075	10,7142857	5,04201681
Small ribosomal subunit	0	0,5952381	5,04201681
Integral to plasma membrane	2,489626556	2,97619048	4,20168067
Perinuclear region of cytoplasn	2,489626556	3,57142857	3,36134454
Eukaryotic translation initiatior	0	0	2,5210084
Early endosome	0,414937759	1,78571429	2,5210084
Microsome	1,244813278	1,78571429	2,5210084
Cell surface	2,489626556	5,95238095	2,5210084
Extracellular matrix	0,414937759	3,57142857	2,5210084
Membrane fraction	1,659751037	4,16666667	2,5210084
Intracellular membrane-bound	1,244813278	1,19047619	2,5210084
Myosin complex	0	0	1,68067227
Proteinaceous extracellular ma	0	0	1,68067227
Endocytic vesicle membrane	0	0	1,68067227
Nucleoplasm	3,319502075	2,38095238	1,68067227
Platelet alpha granule lumen	0,414937759	2,97619048	1,68067227
Endoplasmic reticulum membr	0,829875519	4,16666667	1,68067227
Cytoplasmic membrane-bound	0	0,5952381	1,68067227
Actin cytoskeleton	1,659751037	1,19047619	1,68067227
Coated pit	0,414937759	0,5952381	1,68067227
Clathrin-coated vesicle	0	0,5952381	1,68067227
Integral to Golgi membrane	0	0	0,84033613
Adherens junction	0	0	0,84033613

Basolateral plasma membrane	0	0	0,84033613
CRD-mediated mRNA stability (	0	0	0,84033613
SPOTS complex	0	0	0,84033613
Midbody	0	0	0,84033613
Recycling endosome membran	0	0	0,84033613
Trans-Golgi network membran	0	0	0,84033613
Vacuolar proton-transporting \	0	0	0,84033613
Intracellular vesicle (None)	0	0	0,84033613
Trans-Golgi network transport	0	0	0,84033613
Eukaryotic translation elongati	0	0	0,84033613
Polysomal ribosome	0	0	0,84033613
Sec61 translocon complex	0	0	0,84033613
Cytosolic ribosome	0	0	0,84033613
Unconventional myosin compl	0	0	0,84033613
Large ribosomal subunit	0	0	0,84033613
Complement component C1 cc	0	0	0,84033613
AP-2 adaptor complex	0	0	0,84033613
COPII vesicle coat	0	0	0,84033613
Clathrin coat of trans-Golgi net	0	0	0,84033613
Telomerase holoenzyme comp	0	0	0,84033613
Intracellular ferritin complex	0	0	0,84033613
Cell cortex	0	0	0,84033613
Synaptic vesicle	0	0	0,84033613
Membrane raft	0	0	0,84033613
Cell junction	0,414937759	0	0,84033613
Secretory granule	0,414937759	0	0,84033613
Stress fiber	0,414937759	0	0,84033613
Fibrinogen complex	0	1,78571429	0,84033613
Late endosome	0	2,38095238	0,84033613
Oligosaccharyltransferase com	0	1,78571429	0,84033613
Nuclear membrane	0,414937759	1,19047619	0,84033613
Integrin complex	0	1,19047619	0,84033613
Perinuclear region	0	1,19047619	0,84033613
Zymogen granule	0,829875519	2,38095238	0,84033613
ER-Golgi intermediate compart	0,829875519	5,35714286	0,84033613
Cytoplasmic vesicle	1,244813278	2,97619048	0,84033613
Microtubule	2,489626556	3,57142857	0,84033613
Clathrin coat	0	0,5952381	0,84033613
Trans-Golgi network	0	0,5952381	0,84033613
Nuclear matrix	0,414937759	0,5952381	0,84033613
Heterogeneous nuclear ribonu	0,829875519	0,5952381	0,84033613
Platelet alpha granule membra	0	0,5952381	0,84033613
Alphav-beta3 integrin-vitronec	0	0,5952381	0,84033613
Cleavage furrow	0	0,5952381	0,84033613
Spherical high-density lipoprot	0	0,5952381	0,84033613
PCNA-p21 complex	0,414937759	0	0
WASH complex	0,414937759	0	0
Coated vesicle	0,414937759	0	0
Extracellular vesicular exosome	0,414937759	0	0
Lipopolysaccharide receptor cc	0,414937759	0	0

I band	0,414937759	0	0
Integral to membrane of meml	0,414937759	0	0
Cornified envelope	0,414937759	0	0
Nascent polypeptide-associate	0,414937759	0	0
Dynactin complex	0,414937759	0	0
Microtubule associated comple	0,414937759	0	0
Transcription export complex	0,414937759	0	0
Small nuclear ribonucleoprotei	0,414937759	0	0
Angiogenin-PRI complex	0,414937759	0	0
Chromosome	0,414937759	0	0
Heterotrimeric G-protein comp	0,414937759	0	0
Transcriptional repressor comp	0,414937759	0	0
Connexon complex	0	0,5952381	0
Integral to endoplasmic reticul	0,829875519	0,5952381	0
Sodium:potassium-exchanging	0	0,5952381	0
Caspase complex	0	0,5952381	0
Apical membrane	0,414937759	0,5952381	0
Intermediate filament cytoskel	0,414937759	0,5952381	0
Spindle	0	0,5952381	0
Proteasome regulatory particle	0,414937759	0,5952381	0
Platelet dense granule membra	0	0,5952381	0
Neuronal cell body	0	0,5952381	0
NuA4 histone acetyltransferase	0,414937759	0	0
U2 snRNP	0,414937759	0	0
Apical part of cell	0,414937759	0	0
Filopodium	0,414937759	0	0
Beta-catenin destruction comp	0,414937759	0	0
Mitochondrial outer membrane	0,414937759	0	0
Mitochondrial inner membrane	0,414937759	0	0
Kinetochore	0,414937759	0	0
Sarcoplasmic reticulum membr	0,414937759	0	0
F-actin capping protein comple	0,414937759	0	0
Mitochondrial intermembrane	0,414937759	0	0
Pseudopodium	0,414937759	0	0
Cell-cell adherens junction	0,414937759	0	0
Brush border	0,414937759	0	0
Internal side of plasma membr	0,414937759	0	0
Eukaryotic translation initiatior	0,414937759	0	0
Muscle thin filament tropomyc	0,414937759	0	0
DNA replication factor C compl	0,414937759	0	0
Mitochondrial inner membrane	2,489626556	0	0
Microtubule cytoskeleton	1,244813278	0	0
Cyclin-dependent protein kinas	0,414937759	0	0
GPI-anchor transamidase comp	0,414937759	0	0
Inhibin-betaglycan-ActRII comp	0,414937759	0	0
MLL5-L complex	0,829875519	0	0
Mitochondrial respiratory chai	0,829875519	0	0
Spliceosomal complex	0,829875519	0	0
Mitochondrial respiratory chai	2,904564315	0	0
Peroxisome	2,489626556	0	0

Condensed chromosome	0,829875519	0	0
Nuclear replication fork	0,414937759	0	0
U4 snRNP	0,414937759	0	0
Cell-substrate junction	0,414937759	0	0
Costamere	0,414937759	0	0
Mitochondrial envelope	0,414937759	0	0
Proteasome regulatory particle	0,414937759	0	0
Peroxisomal matrix	0,829875519	0	0
U6 snRNP	0,414937759	0	0
Inhibin A complex	0,414937759	0	0
Protein complex	1,659751037	1,78571429	0
Soluble fraction	1,244813278	1,78571429	0
Proteasome complex	3,734439834	2,38095238	0
Mitochondrial outer membrane	0	1,78571429	0
External side of plasma membr	0	2,38095238	0
Polysome	0,414937759	1,19047619	0
Stored secretory granule	0	1,19047619	0
Mitochondrial nucleoid	2,489626556	1,19047619	0
Mitochondrial matrix	8,298755187	1,19047619	0
Endocytic vesicle	0	1,19047619	0
Extrinsic to external side of pla	0	1,19047619	0
Hemoglobin complex	0	1,78571429	0
Very-low-density lipoprotein pa	0,414937759	1,19047619	0
High-density lipoprotein particl	0	1,19047619	0
Golgi membrane	0	2,38095238	0
Platelet alpha granule	0	2,38095238	0
Endoplasmic reticulum lumen	0	4,76190476	0
Intracellular	1,659751037	5,35714286	0
Mitochondrial membrane	2,074688797	0,5952381	0
Basal plasma membrane	0	0,5952381	0
Pore complex	0	0,5952381	0
Sarcoplasmic reticulum	0,414937759	0,5952381	0
Golgi-associated vesicle memb	0	0,5952381	0
Mitochondrial large ribosomal	0	0,5952381	0
Lysosomal membrane	0	0,5952381	0
Apical plasma membrane	0	0,5952381	0
Intermediate-density lipoprote	0	0,5952381	0
Cell projection	1,244813278	0,5952381	0
Chylomicron	0	0,5952381	0
Holo TFIIH complex	0	0,5952381	0
Nucleosome	0	0,5952381	0
Ruffle	0	0,5952381	0
Chromatin	0	0,5952381	0
Proteasome core complex	0,414937759	0,5952381	0
Sarcolemma	0	0,5952381	0
Collagen type VI	0	0,5952381	0
Weibel-Palade body	0	0,5952381	0
Proton-transporting two-secto	0	0,5952381	0
Intrinsic to endosome membra	0	0,5952381	0
Dendrite	0	0,5952381	0

Others	1,244813278	0,5952381	0
Basal part of cell	0	0,5952381	0
Intermediate filament	0	0,5952381	0
Paraspeckles	0,414937759	0,5952381	0
Ruffle membrane	0	1,19047619	0
Inclusion body	0	0,5952381	0
Nuclear speck	0	0,5952381	0
Low-density lipoprotein particl	0	0,5952381	0
Nuclear pore	0,414937759	0,5952381	0
Recycling endosome	0	0,5952381	0
Nuclear envelope	0,829875519	0,5952381	0
Neuromuscular junction	0	0,5952381	0
Focal adhesion	0,829875519	0,5952381	0

	% of proteins		
**Molecular function**	**GC**	**GC-EV**	**EV**
Catalytic activity	17,07317073	4,73372781	0,81967213
Molecular function unknown	10,16260163	11,8343195	12,295082
Oxidoreductase activity	7,723577236	1,77514793	0
Ubiquitin-specific protease acti	6,097560976	2,95857988	0,81967213
Transporter activity	4,471544715	8,28402367	4,91803279
Chaperone activity	4,471544715	7,10059172	0,81967213
Hydrolase activity	4,06504065	0,59171598	0
Receptor signaling complex sca	2,845528455	0,59171598	2,45901639
Ligase activity	2,845528455	0	0,81967213
Isomerase activity	2,43902439	1,77514793	0
RNA binding	2,032520325	2,36686391	5,73770492
Auxiliary transport protein acti	2,032520325	1,18343195	4,09836066
Transcription regulator activity	2,032520325	0	1,63934426
GTPase activity	2,032520325	5,91715976	1,63934426
Structural constituent of cytosl	1,62601626	1,77514793	0,81967213
Acyltransferase activity	1,62601626	0	0
Translation regulator activity	1,219512195	1,77514793	1,63934426
Peroxidase activity	1,219512195	1,77514793	0
Glutathione transferase activity	1,219512195	1,77514793	0
Cytoskeletal protein binding	1,219512195	1,18343195	0
ATPase activity	0,81300813	1,77514793	2,45901639
Protease inhibitor activity	0,81300813	2,36686391	1,63934426
Serine-type peptidase activity	0,81300813	1,18343195	0,81967213
DNA binding	0,81300813	1,18343195	0,81967213
Transferase activity, transferrir	0,81300813	0	0
Transaminase activity	0,81300813	0,59171598	0
Ribonucleoprotein	0,81300813	0	0
Protein serine/threonine phos	0,81300813	0	0
Peptide hormone	0,81300813	0	0
Lyase activity	0,81300813	0	0
Cysteine-type peptidase activit	0,81300813	0	0
Cell adhesion molecule activity	0,81300813	1,77514793	0
Structural molecule activity	0,406504065	0,59171598	2,45901639
Complement activity	0,406504065	2,95857988	2,45901639

Receptor activity	0,406504065	4,14201183	1,63934426
Protein binding	0,406504065	2,36686391	1,63934426
Complement binding	0,406504065	0	1,63934426
Calcium ion binding	0,406504065	2,95857988	1,63934426
Cytokine activity	0,406504065	0	0,81967213
Carboxypeptidase activity	0,406504065	0	0,81967213
Transmembrane receptor activ	0,406504065	0	0
Transferase activity	0,406504065	1,18343195	0
Transcription factor activity	0,406504065	0	0
Superoxide dismutase activity	0,406504065	0	0
Sialyltransferase activity	0,406504065	0	0
Receptor binding	0,406504065	0	0
Protein transporter activity	0,406504065	0	0
Phosphorylase activity	0,406504065	0,59171598	0
Phospholipase activity	0,406504065	0	0
MHC class II receptor activity	0,406504065	0	0
MHC class I receptor activity	0,406504065	0	0
Methyltransferase activity	0,406504065	0	0
Intracellular ligand-gated ion cl	0,406504065	0	0
Hydro-lyase activity	0,406504065	0	0
Heat shock protein activity	0,406504065	2,36686391	0
Fucosyltransferase activity	0,406504065	0	0
DNA repair protein	0,406504065	0,59171598	0
Deoxyribonuclease activity	0,406504065	0	0
Defense/immunity protein acti	0,406504065	0,59171598	0
Cytoskeletal anchoring activity	0,406504065	0	0
CoA-transferase activity	0,406504065	0	0
Caspase activator activity	0,406504065	0	0
Amidinotransferase activity	0,406504065	0	0
Structural constituent of riboso	0	7,10059172	32,7868852
Extracellular matrix structural c	0	1,18343195	3,27868852
Storage protein	0	0	0,81967213
RNA-directed DNA polymerase	0	0	0,81967213
Protein translocase activity	0	0	0,81967213
Polysaccharide binding	0	0	0,81967213
Nucleocytoplasmic transporter	0	0	0,81967213
Metallopeptidase activity	0	0,59171598	0,81967213
Metal ion binding	0	0	0,81967213
Ion channel activity	0	0	0,81967213
Intracellular transporter activit	0	0	0,81967213
Immunoglobulin binding	0	0	0,81967213
Guanyl-nucleotide exchange fa	0	0	0,81967213
Enzyme regulator activity	0	0	0,81967213
Chemokine activity	0	0	0,81967213
Channel regulator activity	0	0	0,81967213
Binding	0	0	0,81967213
Antigen binding	0	0	0,81967213
Voltage-gated ion channel activ	0	1,77514793	0
Steroid hormone receptor activ	0	0,59171598	0
Peptidase activity	0	1,77514793	0

Motor activity	0	0,59171598	0
Glucosidase activity	0	0,59171598	0
Galactosyltransferase activity	0	1,18343195	0
Aspartic-type signal peptidase a	0	0,59171598	0

	% of proteins		
**Biological process**	**GC**	**GC-EV**	**EV**
Protein metabolism	41,80327869	14,6341463	30,7692308
Transport	14,75409836	6,91056911	11,8343195
Cell communication	10,6557377	12,195122	16,5680473
Signal transduction	10,6557377	13,4146341	15,9763314
Regulation of nucleobase, nucl	9,016393443	7,31707317	4,14201183
Biological_process unknown	8,196721311	7,31707317	8,28402367
Immune response	6,557377049	0,81300813	4,73372781
Cell growth and/or maintenanc	6,557377049	4,87804878	6,50887574
Metabolism	2,459016393	42,2764228	14,7928994
Energy pathways	2,459016393	42,6829268	14,7928994
Apoptosis	1,639344262	0,81300813	0
Cell cycle	0,819672131	0	0
Development	0	0,40650407	0
Regulation of cell cycle	0	0,81300813	0
Proteolysis and peptidolysis	0	0,40650407	0
Xenobiotic metabolism	0	0,40650407	0
DNA repair	0	0,40650407	0
Purine salvage	0	0,40650407	0
Complement activation	0	0,40650407	0
Regulation of immune respons	0	0,40650407	0
Cell activation	0	0	0,59171598
Regulation of gene expression,	0	0	0,59171598
Cell proliferation	0	0,40650407	0,59171598
Cell differentiation	0	0	0,59171598
Protein folding	0	0,81300813	1,18343195
Lipid metabolism	0	0	0,59171598
Anti-apoptosis	0	0,40650407	0,59171598
Immune cell migration	0	0	0,59171598
Cell adhesion	0	0	0,59171598
Carbohydrate metabolism	0	0	0,59171598

	% of proteins		
**Biological pathway**	**GC**	**GC-EV**	**EV**
Developmental Biology	3,488372093	15,2380952	58,1395349
Gene Expression	3,488372093	15,2380952	56,9767442
Metabolism of proteins	5,23255814	25,7142857	55,8139535
Translation	0,581395349	12,3809524	53,4883721
Metabolism	39,53488372	26,6666667	52,3255814
L13a-mediated translational sil	0,581395349	10,4761905	52,3255814
GTP hydrolysis and joining of th	0,581395349	10,4761905	52,3255814
Cap-dependent Translation Init	0,581395349	10,4761905	52,3255814
Integrin family cell surface inte	16,27906977	32,3809524	19,7674419
Beta1 integrin cell surface inter	15,69767442	31,4285714	19,7674419

Proteoglycan syndecan-mediat	15,69767442	33,3333333	18,6046512
Syndecan-1-mediated signaling	15,11627907	31,4285714	18,6046512
TRAIL signaling pathway	16,27906977	30,4761905	17,4418605
Sphingosine 1-phosphate (S1P)	15,69767442	29,5238095	17,4418605
PAR1-mediated thrombin signa	15,69767442	30,4761905	17,4418605
Thrombin/protease-activated r	15,69767442	30,4761905	17,4418605
Signaling events mediated by V	15,11627907	31,4285714	17,4418605
VEGF and VEGFR signaling netw	15,11627907	32,3809524	17,4418605
Cytokine Signaling in Immune s	2,325581395	0	3,48837209
Nef Mediated CD4 Down-regul	0	0	2,3255814
Nef-mediates down modulatio	0	0	2,3255814
Nef Mediated CD8 Down-regul	0	0	2,3255814
Metabolism of nucleotides	3,488372093	0,95238095	1,1627907
p75 NTR receptor-mediated sig	1,162790698	0,95238095	1,1627907
DNA Repair	1,162790698	0,95238095	1,1627907
IRS-mediated signalling	0,581395349	0	1,1627907
Neurotransmitter Receptor Bin	0,581395349	0	1,1627907
Signaling by FGFR	0,581395349	0	1,1627907
Classical antibody-mediated co	0,581395349	0	1,1627907
Creation of C4 and C2 activator	0,581395349	0	1,1627907
NOD1/2 Signaling Pathway	0	0	1,1627907
Trafficking of GluR2-containing	0	0	1,1627907
Toll Receptor Cascades	0	0	1,1627907
mRNA 3'-end processing	0	0	1,1627907
Circadian Clock	0	0	1,1627907
MyD88 cascade initiated on pla	0	0	1,1627907
IRAK2 mediated activation of T	0	0	1,1627907
Toll Like Receptor 9 (TLR9) Casc	0	0	1,1627907
Activated TLR4 signalling	0	0	1,1627907
PECAM1 interactions	0	0	1,1627907
Toll Like Receptor TLR6:TLR2 C	0	0	1,1627907
MyD88-independent cascade in	0	0	1,1627907
TRAF6 mediated induction of T	0	0	1,1627907
TRAF6 mediated induction of N	0	0	1,1627907
NFkB and MAP kinases activati	0	0	1,1627907
Deadenylation-dependent mRN	0	0	1,1627907
p75NTR signals via NF-kB	0	0	1,1627907
Trafficking of AMPA receptors	0	0	1,1627907
ER to Golgi Transport	0	0	1,1627907
pyrimidine ribonucleotides de r	0	0	1,1627907
TRIF mediated TLR3 signaling	0	0	1,1627907
RNA Polymerase II Transcriptio	0	0	1,1627907
Neurofascin interactions	0	0	1,1627907
mTORC1-mediated signalling	0	0	1,1627907
mRNA Splicing - Minor Pathway	0	0	1,1627907
COPII (Coat Protein 2) Mediate	0	0	1,1627907
Post-Elongation Processing of I	0	0	1,1627907
RIG-I/MDA5 mediated inductio	0	0	1,1627907
IRAK2 mediated activation of T	0	0	1,1627907
Post-Elongation Processing of t	0	0	1,1627907

Toll Like Receptor 5 (TLR5) Cas	0	0	1,1627907
mRNA Decay by 5' to 3' Exoribo	0	0	1,1627907
Glutamate Binding, Activation	0	0	1,1627907
MyD88:Mal cascade initiated o	0	0	1,1627907
TRAF6 Mediated Induction of p	0	0	1,1627907
uridine-5'-phosphate biosynthe	0	0	1,1627907
Toll Like Receptor 7/8 (TLR7/8)	0	0	1,1627907
MyD88 dependent cascade init	0	0	1,1627907
Toll Like Receptor 2 (TLR2) Cas	0	0	1,1627907
Toll Like Receptor 10 (TLR10) C	0	0	1,1627907
Toll Like Receptor 4 (TLR4) Cas	0	0	1,1627907
Disinhibition of SNARE formati	0	0	1,1627907
Toll Like Receptor TLR1:TLR2 C	0	0	1,1627907
Negative regulators of RIG-I/M	0	0	1,1627907
Toll Like Receptor 3 (TLR3) Cas	0	0	1,1627907
RNA Polymerase II Transcriptio	0	0,95238095	1,1627907
Insulin receptor recycling	0	0,95238095	1,1627907
Beta5 beta6 beta7 and beta8 in	0	0,95238095	1,1627907
Validated transcriptional target	0	0,95238095	1,1627907
Regulation of the Fanconi aner	0	0	1,1627907
PI3K Cascade	0	0	1,1627907
S6K1 signalling	0	0	1,1627907
Spry regulation of FGF signaling	0	0	1,1627907
PKB-mediated events	0	0	1,1627907
Cleavage of Growing Transcript	0	0	1,1627907
Endosomal Sorting Complex Re	0	0	1,1627907
IL4-mediated signaling events	0	0	1,1627907
Signaling events mediated by P	0	0	1,1627907
NF-kB is activated and signals s	0	0	1,1627907
p75NTR recruits signalling com	0	0	1,1627907
Pyrimidine metabolism	0	0	1,1627907
Cyclin D associated events in G	0	0	1,1627907
Transport of Mature mRNA der	0	0	1,1627907
Telomere Extension By Telome	0	0	1,1627907
APC/C:Cdc20 mediated degrad	0	0	1,1627907
Fanconi Anemia pathway	0	0	1,1627907
NRIF signals cell death from the	0	0	1,1627907
Association of licensing factors	0	0	1,1627907
mTOR signalling	0	0	1,1627907
G1 Phase	0	0	1,1627907
Transport of Mature Transcript	0	0	1,1627907
S6K1-mediated signalling	0	0	1,1627907
Negative regulation of FGFR sig	0	0	1,1627907
Pyrimidine biosynthesis	0	0	1,1627907
Respiratory electron transport	6,976744186	0	0
Respiratory electron transport,	6,976744186	0	0
Citric acid cycle (TCA cycle)	6,976744186	0,95238095	0
Fatty acid, triacylglycerol, and l	6,395348837	0	0
Mitochondrial Fatty Acid Beta-	4,651162791	0	0
mitochondrial fatty acid beta-c	3,488372093	0	0

Folding of actin by CCT/TriC	3,488372093	0,95238095	0
Trk receptor signaling mediate	3,488372093	0,95238095	0
fatty acid beta-oxidation I	2,906976744	0	0
isoleucine degradation I	2,906976744	0	0
valine degradation I	2,906976744	0	0
Branched-chain amino acid cat	2,906976744	0	0
Beta oxidation of hexanoyl-CoA	2,325581395	0	0
Beta oxidation of decanoyl-CoA	2,325581395	0	0
Integration of energy metaboli	2,325581395	0	0
Beta oxidation of octanoyl-CoA	2,325581395	0	0
sucrose degradation	1,744186047	0	0
superoxide radicals degradatio	1,744186047	0	0
colanic acid building blocks bio	1,162790698	0	0
2-ketoglutarate dehydrogenase	1,162790698	0	0
Endogenous sterols	1,162790698	0	0
eNOS activation and regulation	1,162790698	0,95238095	0
Nongenotropic Androgen signa	1,162790698	0,95238095	0
Intrinsic Pathway for Apoptosis	1,162790698	0,95238095	0
pentose phosphate pathway (c	1,162790698	0,95238095	0
TCR signaling in naïve CD	1,162790698	0,95238095	0
Pyruvate metabolism	1,162790698	0,95238095	0
Aurora B signaling	1,162790698	0,95238095	0
Activation of G protein gated P	0,581395349	0	0
Resolution of D-loop structures	0,581395349	0	0
RAF/MAP kinase cascade	0,581395349	0	0
adenosine nucleotides degrada	0,581395349	0	0
RNA Polymerase III Transcriptio	0,581395349	0	0
Activation of BH3-only proteins	0,581395349	0	0
Release of apoptotic factors fro	0,581395349	0	0
glutaryl-CoA degradation	0,581395349	0	0
Double-Strand Break Repair	0,581395349	0	0
Activation of BAD and transloca	0,581395349	0	0
Homologous Recombination Re	0,581395349	0	0
guanosine nucleotides degrada	0,581395349	0	0
NADPH regeneration	0,581395349	0	0
Synthesis of bile acids and bile	0,581395349	0	0
Phenylalanine and tyrosine cat	0,581395349	0	0
Creatine metabolism	0,581395349	0	0
Inactivation of APC/C via direct	0,581395349	0	0
Activation of Kainate Receptors	0,581395349	0	0
GABA B receptor activation	0,581395349	0	0
Proline catabolism	0,581395349	0	0
Recycling of eIF2:GDP	0,581395349	0	0
purine ribonucleosides degrada	0,581395349	0	0
purine nucleotides degradation	0,581395349	0	0
tryptophan degradation III (euk	0,581395349	0	0
Activation of GABAB receptors	0,581395349	0	0
Destabilization of mRNA by But	0,581395349	0	0
SMAC-mediated dissociation of	0,581395349	0	0
citrulline biosynthesis	0,581395349	0	0

Inwardly rectifying K+ channels	0,581395349	0	0
Beta oxidation of myristoyl-CoA	0,581395349	0	0
Galactose catabolism	0,581395349	0	0
G-protein beta:gamma signallir	0,581395349	0	0
Hormone-sensitive lipase (HSL)	0,581395349	0	0
SMAC-mediated apoptotic resp	0,581395349	0	0
RNA Polymerase III Transcriptic	0,581395349	0	0
RAF phosphorylates MEK	0,581395349	0	0
RNA Polymerase III Abortive Ar	0,581395349	0	0
RAF activation	0,581395349	0	0
MEK activation	0,581395349	0	0
SMAC binds to IAPs	0,581395349	0	0
G alpha (12/13) signalling even	0,581395349	0,95238095	0
HIF-2-alpha transcription facto	0,581395349	0,95238095	0
Cell-extracellular matrix intera	0,581395349	0,95238095	0
FOXA1 transcription factor net	0,581395349	0,95238095	0
Signaling events mediated by t	0,581395349	0,95238095	0
Nucleotide Excision Repair	0,581395349	0,95238095	0
SEMA3A-Plexin repulsion signa	0,581395349	0,95238095	0
Netrin-mediated signaling ever	0,581395349	0,95238095	0
Global Genomic NER (GG-NER)	0,581395349	0,95238095	0
Pregnenolone biosynthesis	0,581395349	0,95238095	0
Hedgehog signaling events me	0,581395349	0	0
CRMPs in Sema3A signaling	0,581395349	0	0
CD28 co-stimulation	0,581395349	0,95238095	0
RNA Polymerase I, RNA Polyme	0,581395349	0,95238095	0
Striated Muscle Contraction	0,581395349	0,95238095	0
Costimulation by the CD28 far	0,581395349	0,95238095	0
Interleukin-3, 5 and GM-CSF sig	0,581395349	0	0
CD28 dependent Vav1 pathwa	0,581395349	0,95238095	0
heme biosynthesis II	0,581395349	0	0
tetrapyrrole biosynthesis II	0,581395349	0	0
folate polyglutamylation	0,581395349	0	0
pregnenolone biosynthesis	0,581395349	0	0
folate transformations	0,581395349	0	0
tetrahydrobiopterin biosynthes	0,581395349	0	0
tetrahydrobiopterin biosynthes	0,581395349	0	0
cysteine biosynthesis III (mamr	0,581395349	0	0
Attachment of GPI anchor to u	0,581395349	0	0
Gap-filling DNA repair synthesi	0,581395349	0	0
Telomere C-strand (Lagging Str	0,581395349	0	0
NADE modulates death signalli	0,581395349	0	0
Displacement of DNA glycosyla	0,581395349	0	0
Resolution of AP sites via the si	0,581395349	0	0
Post-translational modification	0,581395349	0	0
mineralocorticoid biosynthesis	0	0,95238095	0
estrogen biosynthesis	0	0,95238095	0
BCR signaling pathway	0	0,95238095	0
Sumoylation by RanBP2 regula	0	0,95238095	0
NRAGE signals death through J	0	0,95238095	0

IL23-mediated signaling events	0	0,95238095	0
Formation of HIV-1 elongation	0	0,95238095	0
RNA Polymerase II HIV-1 Prom	0	0,95238095	0
Wnt	0	0,95238095	0
Activation of Rac	0	0,95238095	0
Post-chaperonin tubulin foldin	0	0,95238095	0
Gamma-carboxylation, transpc	0	0,95238095	0
E-cadherin signaling in keratinc	0	0,95238095	0
Regulatory RNA pathways	0	0,95238095	0
Gamma-carboxylation of prote	0	0,95238095	0
Validated nuclear estrogen rec	0	0,95238095	0
ABC-family proteins mediated	0	0,95238095	0
DSCAM interactions	0	0,95238095	0
Sema4D mediated inhibition of	0	0,95238095	0
Aurora A signaling	0	0,95238095	0
CD40/CD40L signaling	0	0,95238095	0
Canonical NF-kappaB pathway	0	0,95238095	0
RNA Polymerase II Pre-transcri	0	0,95238095	0
Formation of the Early Elongati	0	0,95238095	0
RNA Polymerase II Transcriptio	0	0,95238095	0
Metal ion SLC transporters	0	0,95238095	0
Type I hemidesmosome assem	0	0,95238095	0
Alpha6 beta4 integrin-ligand in	0	0,95238095	0
Alternative complement activa	0	0,95238095	0
Other semaphorin interactions	0	0,95238095	0
Signaling events mediated by H	0	0,95238095	0
Dual incision reaction in GG-NE	0	0,95238095	0
RNA Pol II CTD phosphorylation	0	0,95238095	0
heme degradation	0	0,95238095	0
mRNA Capping	0	0,95238095	0
acetone degradation I (to meth	0	0,95238095	0
glutamate degradation II	0	0,95238095	0
a4b7 Integrin signaling	0	0,95238095	0
TCR signaling in naïve CD	0	0,95238095	0
MicroRNA (miRNA) Biogenesis	0	0,95238095	0
The NLRP3 inflammasome	0	0,95238095	0
EPHA2 forward signaling	0	0,95238095	0
GPVI-mediated activation casca	0	0,95238095	0
Formation of RNA Pol II elonga	0	0,95238095	0
Inflammasomes	0	0,95238095	0
