# Supplementary material for: Protein Cargo of Extracellular Vesicles From Bovine Follicular Fluid and Analysis of Their Origin From Different Ovarian Cells
Source: Front Vet Sci. 2020 Nov 4;7:584948. doi: 10.3389/fvets.2020.584948 (PMC7672127; doi:10.3389/fvets.2020.584948)
Supplement: Supplementary Table 4 — Enrichment Analysis of GO terms of ffEV proteins using DAVID. [file Table_4.pdf]

Supplementary Table S4. Enrichment Analysis of GO terms of ffEV proteins using DAVID

KEGG_PATHWAY	Count	%	*PValue*	Genes	Fold Enrichment
bta03010:Ribosome	57	18.6	*2.25E-54*	RPL18, RPL17, RPL19, RPL14, RPL13, RPL15, RPS27L, RPS2, RPS3, RPS3A, RPLP0, RPL10, RPL11, RPL12, RPS27A, RPL35A, MRPL3, RPS18, RPS16, RPS17, RPS14, RPS4Y1, RPS13, RPS10, RPS11, RPL27A, RPL35, RPS15A, RPL36, RPL38, RPL10L, RPS25, RPS26, RPL30, RPL32, RPL7, RPL6, RPL9, RPL8, RPL5, RPL7A, RPL4, RPS23, RPS24, RPSA, RPL26, RPL27, RPS9, RPL23A, RPS6, RPS5, RPS8, RPS7, RPL23, RPL18A, RPL13A, RPL21	15.6
bta04141:Protein processing in endoplasmic reticulum	26	8.5	*2.41E-12*	HSP90AB1, GANAB, PDIA3, PDIA6, PDIA4, LMAN2, PRKCSH, CALR, LMAN1, CANX, DAD1, RPN1, HSPA6, RPN2, HSPA5, SEC24C, DDOST, HSPA8, SEC61A1, HSP90AA1, CKAP4, ERP29, HSP90B1, VCP, SSR4, UGGT1	5.6
bta04145:Phagosome	22	7.2	*1.17E-09*	CGN1, RAB7A, STX7, TUBB2A, C3, MRC2, ITGA2, ITGB3, CALR, ITGB1, M6PR, CANX, LAMP2, TFRC, ATP6V1E1, ITGAV, RAC1, SEC22B, ATP6V0D1, DYNC1I2, TUBA1C, SEC61A1	5.1
bta04610:Complement and coagulation cascades	15	4.9	*7.74E-09*	A2M, C9, C4A, C3, C5, PLG, C8G, C1QB, FGG, FGA, FGB, F2, CFH, SERPIND1, CFI	7.4
bta04961:Endocrine and other factor-regulated calcium reabsorption	6	2	*0.0051*	AP2B1, CLTA, AP2A1, RAB11A, ATP1A1, CLTC	5.2
bta04721:Synaptic vesicle cycle	7	2.3	*0.0068*	AP2B1, CLTA, AP2A1, ATP6V1E1, NAPA, ATP6V0D1, CLTC	4.1
bta04512:ECM-receptor interaction	8	2.6	*0.0089*	ITGA6, ITGAV, ITGA2, COL6A1, VTN, ITGB3, ITGB1, FN1	3.4
bta05020:Prion diseases	5	1.6	*0.0102*	C1QB, C9, C5, HSPA5, C8G	5.7
bta04918:Thyroid hormone synthesis	7	2.3	*0.0105*	GPX1, HSP90B1, ATP1A1, GPX8, HSPA5, PDIA4, CANX	3.7
bta04612:Antigen processing and presentation	7	2.3	*0.0155*	HSP90AB1, HSP90AA1, PDIA3, HSPA6, CALR, CANX, HSPA8	3.4
bta03050:Proteasome	5	1.6	*0.0349*	PSMA1, PSMC6, PSMA6, PSMA5, PSMD2	4.0
bta00510:N-Glycan biosynthesis	5	1.6	*0.0426*	GANAB, DAD1, RPN1, RPN2, DDOST	3.7

GOTERM_MOLECULAR FUNCTION_DIRECT	Count		*PValue*	Genes	Fold Enrichment
GO:0003735~structural constituent of ribosome	56	18.2	*7.31E-48*	RPL18, RPL17, RPL19, RPL14, RPL13, RPL15, RPS27L, RPS2, RPS3, RPS3A, RPLP0, RPL10, RPL11, RPL12, RPS27A, RPL35A, MRPL3, RPS18, RPS16, RPS17, RPS14, RPS4Y1, RPS13, RPS10, RPS11, RPL27A, RPL35, RPS15A, RPL36, RPL38, RPL10L, RPS26, RPL30, RPL32, RPL7, RPL6, RPL9, RPL8, RPL5, RPL4, RPS23, RPS24, RPSA, RPL26, RPL27, RPS9, RPL23A, RPS6, RPS5, RPS8, RPS7, SLC25A12, RPL23, RPL18A, RPL13A, RPL21	13.9
GO:0044822~poly(A) RNA binding	57	18.6	2.86E-17	RPL14, PDIA3, RPL13, RPL15, PDIA4, CANX, PRDX1, GOT2, RPS3A, RPLP0, RPN1, RPL11, RPL12, RPS27A, HIST1H1D, HSP90AA1, MRPL3, EEF2, HNRNPU, RPS18, RPS16, HSPB1, RPS11, MDH2, GANAB, MTDH, RPS15A, RPL36, SERPINH1, HNRNPA3, RPS25, RACK1, RPL30, RPL32, RPL7, RPL8, RPL7A, HSPA8, RPS23, RPS24, ERH, CKAP4, RPL26, RPS9, RPL23A, RPS6, RPS8, ANXA2, RPS7, HNRNPH2, RPL23, SRSF7, RPL18A, PPIB, VCP, TFRC, SFPQ	3.6
GO:0019843~rRNA binding	10	3.3	*5.02E-09*	RPS18, RPL9, RPL8, RPS9, RPS4Y1, RPL11, RPL23A, RPL12, RPS11, RPS5	16.5
GO:0098641~cadherin binding involved in cell-cell adhesion	12	3.9	*3.13E-08*	RACK1, PCBP1, RPL15, EEF2, RPL23A, RPL7A, RPS2, EEF1D, PRDX1, CLINT1, HSPA8, ANXA2	9.7
GO:0003729~mRNA binding	11	3.6	*0.00002*	RPS26, EIF3A, RPL7, RPL13A, TRA2B, RPL35, RPS13, RPS2, CALR, RPS5, RPS3	5.7
GO:0051082~unfolded protein binding	8	2.6	*0.00003*	HSP90AB1, HSP90B1, HSP90AA1, CCT8, CALR, UGGT1, SERPINH1, HSPA8	8.4
GO:0005537~mannose binding	5	1.6	*0.00018*	CGN1, BSG, LMAN2, LMAN1, M6PR	16.5
GO:0004579~dolichyl-diphosphooligosaccharide-protein glycotransferase activity	4	1.3	*0.00034*	DAD1, RPN1, RPN2, DDOST	26.4
GO:0003924~GTPase activity	12	3.9	*0.00046*	RAB2A, RAB7A, RAN, TUBB2A, GNA11, EEF1A2, RAC1, RAB14, RAB11A, EEF2, RAB1A, TUBA1C	3.6
GO:0005525~GTP binding	17	5.5	*0.00086*	RAB2A, RAB7A, SEPT2, TUBB2A, RAN, EEF1A2, GNA11, RAB4B, EEF2, RAB1A, ANXA6, ARF3, RAC1, RAB14, RAB11A, RAB6A, TUBA1C	2.6
GO:0004867~serine-type endopeptidase inhibitor activity	8	2.6	*0.0011*	A2M, SERPINE2, ITIH4, SERPINA3, HRG, ITIH2, SERPIND1, SERPINH1	4.9
GO:0015485~cholesterol binding	5	1.6	*0.002*	APOA4, ANXA6, APOA1, APOE, ERLIN1	9.1

GOTERM_CELLULAR COMPONENT	Count		*PValue*	Genes	Fold Enrichment

GO:0070062~extracellular exosome	152	49.5	*5.32E-56*	A2M, SEPT2, RPL14, S100A8, TUBB2A, GNA11, RPL15, PGD, VTN, ALDH1L2, RAB1A, GOT2, APOA4, TTR, APOA1, H2AFV, PLOD1, RPLP0, PGRMC1, RPL11, RPL12, CFI, RPS27A, BSG, SCAMP3, SCAMP2, ERP29, EEF2, MFGE8, PSMA1, C1QB, RPS18, IGSF8, CHID1, RPS16, PSMA6, PSMA5, F2, RAB14, HSPB1, SDCBP, RPS13, RPS11, CPD, UGGT1, MDH2, MVP, MYL6, RBP4, RAB7A, STX7, NAPA, LMAN2, LMAN1, CALR, SERPINH1, RPS25, RPS26, PFN2, FGG, EIF3B, ECE1, RPL7, FGB, ITGAV, RAC1, TMED10, RPL7A, FN1, CKAP4, RPS9, RPL23A, VDAC2, RPS5, RPS8, RPS7, VDAC1, LAMP2, PPIB, VCP, SRSF7, MYH10, CYB5R3, PDIA3, PRDX4, PDIA6, GJA1, CAD, RPS2, CANX, PRDX1, RPS3, TMEM109, DNAJC13, RPS3A, PCBP1, PSMD2, ITIH4, ITIH2, ATP6V0D1, GOLM1, HSP90AA1, C8G, STOM, CD81, EEF1G, TF, GANAB, C9, C3, C5, VIM, RPS15A, MIF, ANXA6, RACK1, CD9, TMED7, TMED4, RPL30, MPDU1, DAD1, HSPA6, RAB11A, COL6A1, HRG, APMAP, GAPDH, MYOF, HSPA8, RAB2A, PLP2, RPL26, ATP1A1, TMED9, ANXA5, ANXA4, ANXA2, BLVRA, A1BG, ERP44, HSP90B1, TFRC, RPL23, HPX, ATP6V1E1, YWHAQ, CP, SERPIND1, PGK1, SSR4, CMTM6	3.8
GO:0022625~cytosolic large ribosomal subunit	30	9.8	*4.58E-34*	RPL18, RPL19, RPL14, RPL13, RPL15, RPL35, RPL27A, RPL36, RPL38, RPL10L, RPL30, RPL32, RPL7, RPL6, RPL9, RPLP0, RPL8, RPL10, RPL11, RPL5, RPL12, RPL7A, RPL4, RPL35A, RPL26, RPL27, RPL23A, RPL23, RPL18A, RPL13A	25.8
GO:0022627~cytosolic small ribosomal subunit	23	7.5	*2.36E-28*	RPSA, RPS9, RPS15A, RPS27L, RPS2, RPS6, RPS5, RPS8, RPS3, RPS7, RPS25, RPS26, RPS18, RPS16, RPS17, RPS3A, RPS4Y1, RPS13, RPS10, RPS11, RPS23, RPS27A, RPS24	31.2
GO:0016020~membrane	71	23.1	*8.05E-26*	RPL18, RPL14, RPL13, GNA11, RPL15, EDC4, CAD, RPS2, PGRMC1, RPLP0, PCBP1, PGRMC2, PSMD2, RPL11, CFI, RPL12, CLINT1, RPS27A, ERP29, MFGE8, HNRNPU, RPS18, CHID1, RPS16, CD81, RPS13, EEF1G, RPS11, CPD, MVP, MYL6, GANAB, ADPGK, RPS15A, RPL36, ITGB1, COLGALT1, RPL10L, ANXA6, CD9, RPS26, EIF3A, RPL30, ECE1, RPL7, RPL32, RPL8, RAC1, RAB11A, RPL7A, APMAP, RPS23, RPS24, RPSA, ERH, RPL26, RPS9, ELAVL1, ANXA5, RPS6, RPS5, ANXA4, RPS8, RPS7, ANXA2, VDAC1, HNRNPH2, RPL23, PPIB, RPL18A, YWHAQ	4.2
GO:0072562~blood microparticle	25	8.1	*8.82E-24*	TF, A2M, C9, C3, VTN, C8G, APOA4, A1BG, STOM, C1QB, FGG, APOA1, TFRC, HPX, FGB, APOE, F2, ITIH4, HSPA6, SDCBP, ITIH2, HRG, CP, HSPA8, FN1	18.1
GO:0005925~focal adhesion	40	13	*2.53E-21*	RPL18, PDIA3, VIM, GJA1, RPS2, CALR, ITGB1, RPS3, ANXA6, CD9, RPL30, RPL7, GSN, RPS3A, ITGAV, RPLP0, RPL8, RAC1, RPL12, RPL7A, HSPA8, BSG, MRC2, ITGA2, RPS9, ANXA5, RPS5, RPS8, RPS7, HSP90B1, RPS18, ITGA6, RPL23, RPS16, PPIB, CD81, YWHAQ, HSPB1, RPS13, RPS11	6.8
GO:0042470~melanosome	20	6.5	*2.00E-17*	HSP90AB1, BSG, HSP90AA1, PDIA3, RAN, ERP29, PDIA4, CLTC, ITGB1, PRDX1, RAB1A, ANXA2, ANXA6, HSP90B1, PPIB, RAC1, CTSD, TMED10, HSPA5, HSPA8	15.3
GO:0031012~extracellular matrix	19	6.2	*3.69E-12*	MYL6, VIM, CLU, RPS15A, MGP, EEF2, MFGE8, RPS5, PRDX1, RPS3, RPS7, HSP90B1, RPS18, RPL30, SERPINE2, RPS16, RPL11, RPS11, HSPA8	8.9
GO:0043209~myelin sheath	18	5.9	*8.78E-10*	HSP90AA1, SEPT2, PDIA3, EEF1A2, ATP1A1, NAPA, CLTC, VDAC2, PRDX1, CANX, VDAC1, MIF, SLC25A12, GOT2, VCP, MDH2, HSPA8, RPS27A	6.9
GO:0005788~endoplasmic reticulum lumen	11	3.6	*2.00E-08*	ERP44, HSP90B1, PDIA3, PPIB, TMEM43, ERP29, HSPA5, PDIA4, CALR, SERPINH1, COLGALT1	11.9
GO:0005840~ribosome	12	3.9	*2.90E-08*	RPL18, RPS25, RPS26, RPL6, RPL13, RPS17, RPL21, RPLP0, RPS14, RPL27A, RPS15A, RPL10	9.9
GO:0030529~intracellular ribonucleoprotein complex	11	3.6	*1.72E-07*	HNRNPH2, RPS3A, RPLP0, PCBP1, RPS9, GAPDH, HNRNPU, RPS8, HSPA8, RPS3, MVP	9.6
GO:0005783~endoplasmic reticulum	28	9.1	*5.08E-07*	CYB5R3, PDIA3, ADPGK, CLU, PRDX4, GJA1, PDIA6, PDIA4, PRKCSH, SERPINH1, PLOD1, EMID1, PGRMC1, MPDU1, APMAP, GOLT1B, CKAP4, ERP29, ERLIN1, LRPAP1, STOM, ERP44, HSP90B1, VCP, EEF1G, ORMDL3, EEF1D, UGGT1	3.1
GO:0005793~endoplasmic reticulum-Golgi intermediate compartment	9	2.9	*5.42E-07*	TMED7, ERP44, SEC22B, TMED10, PDIA6, LMAN2, LMAN1, SERPINH1, FN1	12.2
GO:0005913~cell-cell adherens junction	12	3.9	*9.52E-07*	RACK1, PCBP1, RPL15, EEF2, RPL23A, RPL7A, RPS2, EEF1D, PRDX1, CLINT1, HSPA8, ANXA2	7.1
GO:0048471~perinuclear region of cytoplasm	23	7.5	*1.32E-06*	STX6, TF, MTDH, STX7, CLU, CALR, RPS6, ANXA4, M6PR, ATP7A, RACK1, STOM, ANXA6, HSP90B1, ECE1, TFRC, PPIB, VCP, ARF3, RAB11A, SORT1, CLINT1, MVP	3.4
GO:0005730~nucleolus	29	9.4	*7.00E-06*	RPL18, SEPT2, MTDH, RPL36, RPS3, RPS25, RPS26, EIF3A, RPL7, RPS3A, PGRMC1, RPL8, RPL11, RPL5, RPL12, RPL7A, RPS23, TERT, HSPA8, RPS27A, RPS9, RPL23A, RPS6, RPS7, RPL23, RPS13, RPS10, RPS11, EEF1D	2.6
GO:0005789~endoplasmic reticulum membrane	21	6.8	*0.000014*	CYB5R3, PLP2, IER3IP1, MTDH, ORMDL1, ERLIN1, LMAN2, LMAN1, TMED9, CANX, RAB1A, TMED7, ERP44, TMED4, HSP90B1, PGRMC1, SEC22B, TMED10, SSR4, FKBP11, DDOST	3.1
GO:0005765~lysosomal membrane	14	4.6	*0.000028*	RAB2A, STX7, GNA11, M6PR, ANXA2, ANXA6, LAMP2, ECE1, DNAJC13, ATP6V1E1, RAB14, COL6A1, CP, ATP6V0D1	4.2
GO:0005844~polysome	6	2	*0.000096*	RPL10L, PSMA6, EEF2, CALR, RPS6, RPS3	12.5
GO:0009897~external side of plasma membrane	12	3.9	*0.00015*	CD9, FGG, ECE1, ITGA6, TFRC, FGB, ITGAV, ITGA2, MFGE8, ITGB3, ANXA5, CALR	4.1
GO:0005577~fibrinogen complex	4	1.3	*0.0002*	FGG, FGA, FGB, FN1	31

GO:0005615~extracellular space	34	11.1	*0.0003*	TF, RBP4, A2M, S100A8, C3, CLU, C5, PRSS1, LMAN2, CALR, SERPINH1, PRDX1, MIF, CD9, TTR, SERPINE2, ALB, PRSS2, SERPINA3, IGLL1, CFI, GOLM1, RPS27A, SRGN, C4A, MFGE8, ANXA2, ORM1, LAMP2, CHID1, HSPB1, CTSD, SERPIND1, CPD	1.9
GO:0008305~integrin complex	5	1.6	*0.0003*	ITGA6, ITGAV, ITGA2, ITGB3, ITGB1	14.3
GO:0008250~oligosaccharyltransferase complex	4	1.3	*0.0004*	DAD1, RPN1, RPN2, DDOST	24.1
GO:0005770~late endosome	8	2.6	*0.0005*	ATP7A, TF, RAB7A, STX7, CHID1, GJA1, M6PR, HSPA8	5.5
GO:0005811~lipid particle	6	2	*0.0019*	CYB5R3, RAB7A, VCP, CKAP4, GAPDH, ANXA2	6.64
GO:0009986~cell surface	15	4.9	*0.0034*	SEPT2, PDIA3, ERP29, LMAN2, PDIA4, ITGB1, ANXA4, ANXA2, MIF, GOT2, APOA4, ERP44, SORT1, PTGFRN, APMAP	2.4
GO:0005802~trans-Golgi network	8	2.6	*0.0037*	ATP7A, CHID1, RAC1, RAB14, RAB11A, CPD, CLINT1, M6PR	4
GO:0036464~cytoplasmic ribonucleoprotein granule	4	1.3	*0.0055*	RPLP0, RAC1, RPS6, HNRNPU	10.8

**GOTERM_BIOLOGICAL PROCESS_DIRECT**	**Count**		***PValue***	**Genes**	**Fold Enrichment**
GO:0006412~translation	49	16	*1.43E-42*	RPL18, RPL17, RPL19, RPL14, RPL13, RPS27L, RPS2, RPS3, RPS3A, RPL10, RPL11, RPL12, RPS27A, EEF2, RPS18, RPS16, RPS17, RPS14, RPS4Y1, RPS13, RPS11, RPL35, RPL27A, RPS15A, RPL36, RPL38, RPL10L, RPS26, RPL30, RPL32, RPL6, RPL5, RPL4, RPL7A, RPS23, RPS24, RPSA, RPS9, RPL27, RPL23A, RPS6, RPS5, RPS8, RPS7, SLC25A12, RPL18A, RPL23, RPL13A, RPL21	14.6
GO:0002181~cytoplasmic translation	9	2.9	*1.50E-08*	RPL35A, RPL7, RPL6, RPLP0, RPL9, RPL8, RPL15, RPL26, RPL36	18.4
GO:0098609~cell-cell adhesion	12	3.9	*6.04E-08*	RACK1, PCBP1, RPL15, EEF2, RPL23A, RPL7A, RPS2, EEF1D, PRDX1, CLINT1, HSPA8, ANXA2	9.2
GO:0000027~ribosomal large subunit assembly	7	2.3	*1.58E-06*	RPL10L, RPL6, RPL10, RPL5, RPL11, RPL23A, RPL12	17.9
GO:0042730~fibrinolysis	6	2	*4.66E-06*	FGG, FGB, F2, HRG, PLG, ANXA2	21.9
GO:0006457~protein folding	12	3.9	*7.33E-06*	HSP90AB1, ERP44, HSP90B1, HSP90AA1, PDIA3, ERP29, CCT8, PDIA6, PDIA4, CALR, FKBP11, CANX	5.7
GO:0033627~cell adhesion mediated by integrin	5	1.6	*0.000061*	ITGA6, ITGAV, ITGA2, VTN, ITGB1	21.3
GO:0010951~negative regulation of endopeptidase activity	8	2.6	*0.00014*	A2M, C3, ITIH4, SERPINA3, VTN, HRG, ITIH2, SERPINH1	6.9
GO:0000028~ribosomal small subunit assembly	5	1.6	*0.00016*	RPSA, RPS17, RPS27L, RPS10, RPS5	17.1
GO:0006958~complement activation, classical pathway	5	1.6	*0.00021*	C1QB, C9, C4A, C3, C5	16
GO:0006957~complement activation, alternative pathway	4	1.3	*0.00024*	C9, C3, C5, CFH	29.2
