# Supplementary material for: Protein Cargo of Extracellular Vesicles From Bovine Follicular Fluid and Analysis of Their Origin From Different Ovarian Cells
Source: Front Vet Sci. 2020 Nov 4;7:584948. doi: 10.3389/fvets.2020.584948 (PMC7672127; doi:10.3389/fvets.2020.584948)
Supplement: Supplementary Table 5 — Gene expression analysis of ffEV proteins in follicular cells. [file Table_5.pdf]

# Supplementary data Table S5

**Differential analysis of expression of the genes coding for the proteins identified in EV from FF, in bovine follicular cells**

Correction post-hoc : Benjamini-Hochberg, Tukey test pair-by-pir

| Genes | p-value | Significance | TH | GR | CC | Oo |
|---|---|---|---|---|---|---|
| A2M | 0,000 | yes | 65,466 (b) | 3,266 (a) | 2,098 (a) | 0,260 (a) |
| ACTL7A | 0,000 | yes | 0,438 (b) | 0,519 (b) | 0,512 (b) | 0,218 (a) |
| ADPGK | 0,000 | yes | 31,081 (b) | 54,929 (c) | 169,217 (d) | 2,840 (a) |
| AKR1B1 | 0,000 | yes | 247,502 (a) | 464,639 (ab) | 700,606 (b) | 1424 (c) |
| ALB | 0,008 | yes | 0,501 (b) | 0,581 (b) | 0,482 (ab) | 0,325 (a) |
| ALDH1L2 | 0,000 | yes | 2,378 (a) | 2,247 (a) | 8,040 (b) | 9,843 (c) |
| ANXA2 | 0,000 | yes | 436,571 (b) | 195,417 (a) | 85,542 (a) | 59,117 (a) |
| ANXA4 | 0,000 | yes | 70,638 (c) | 46,905 (b) | 74,670 (c) | 3,633 (a) |
| ANXA5 | 0,000 | yes | 169,587 (b) | 64,802 (a) | 68,781 (a) | 24,884 (a) |
| ANXA6 | 0,003 | yes | 261,764 (b) | 183,841 (ab) | 269,382 (b) | 31,290 (a) |
| AP2A1 | 0,155 | no | 2,503 (a) | 1,941 (a) | 2,148 (a) | 0,802 (a) |
| AP2B1 | 0,006 | yes | 1,095 (a) | 1,112 (a) | 1,324 (ab) | 1,658 (b) |
| APMAP | 0,000 | yes | 8,963 (b) | 12,230 (b) | 23,661 (c) | 1,428 (a) |
| APOA1 | 0,000 | yes | 2400 (b) | 644,318 (a) | 658,087 (a) | 4,217 (a) |
| APOA4 | 0,000 | yes | 0,513 (a) | 0,648 (a) | 0,573 (a) | 1,900 (b) |
| APOE | 0,000 | yes | 565,884 (b) | 447,998 (b) | 297,803 (b) | 1,740 (a) |
| ATP1A1 | 0,051 | no | 15,736 (a) | 16,332 (a) | 30,850 (a) | 22,919 (a) |
| ATP6V0D1 | 0,001 | yes | 278,325 (c) | 155,328 (ab) | 130,638 (a) | 236,164 (bc) |
| ATP6V1E1 | 0,038 | yes | 84,153 (a) | 129,148 (ab) | 150,812 (ab) | 174,107 (b) |
| ATP7A | 0,000 | yes | 0,889 (a) | 3,509 (b) | 3,238 (b) | 0,667 (a) |
| B4GALT4 | 0,001 | yes | 12,760 (ab) | 24,661 (bc) | 29,095 (c) | 5,509 (a) |
| BLVRA | 0,000 | yes | 25,940 (a) | 75,982 (b) | 130,174 (c) | 1,068 (a) |
| BSG | 0,219 | no | 30,547 (a) | 10,957 (a) | 15,889 (a) | 1,855 (a) |
| C1QB | 0,000 | yes | 45,613 (b) | 5,565 (a) | 4,895 (a) | 0,242 (a) |
| C1QB | 0,000 | yes | 45,613 (b) | 5,565 (a) | 4,895 (a) | 0,242 (a) |
| C3 | 0,000 | yes | 0,580 (b) | 0,714 (b) | 1,800 (c) | 0,210 (a) |
| C5 | 0,000 | yes | 0,787 (a) | 0,604 (a) | 0,494 (a) | 14,175 (b) |
| C8G | 0,000 | yes | 11,498 (b) | 9,517 (b) | 13,900 (b) | 1,867 (a) |
| C9 | 0,000 | yes | 0,561 (c) | 0,468 (bc) | 0,439 (b) | 0,228 (a) |
| CAD | 0,000 | yes | 80,696 (b) | 100,093 (b) | 162,020 (c) | 4,871 (a) |
| CALR | 0,001 | yes | 896,312 (bc) | 801,220 (ab) | 1283 (c) | 420,413 (a) |
| CAMTA1 | 0,000 | yes | 2,151 (a) | 2,482 (a) | 4,958 (b) | 21,723 (c) |
| CANX | 0,003 | yes | 234,081 (ab) | 233,852 (ab) | 318,600 (b) | 153,760 (a) |
| CCT8 | 0,000 | yes | 154,748 (a) | 169,511 (a) | 187,311 (a) | 540,190 (b) |
| CD81 | 0,231 | no | 66,963 (a) | 13,256 (a) | 18,177 (a) | 22,398 (a) |
| CD9 | 0,000 | yes | 62,285 (b) | 25,507 (a) | 16,167 (a) | 14,785 (a) |
| CFH | 0,000 | yes | 129,535 (b) | 4,948 (a) | 0,507 (a) | 0,225 (a) |
| CFI | 0,024 | yes | 4,435 (b) | 0,943 (a) | 0,637 (a) | 1,173 (ab) |
| CGN1 | 0,000 | yes | 0,456 (a) | 0,577 (a) | 0,527 (a) | 1,246 (b) |
| CHID1 | 0,000 | yes | 259,347 (b) | 219,999 (b) | 244,356 (b) | 52,224 (a) |
| CKAP4 | 0,025 | yes | 531,340 (ab) | 674,865 (ab) | 792,857 (b) | 383,658 (a) |
| CLINT1 | 0,000 | yes | 7,523 (a) | 7,613 (a) | 6,886 (a) | 33,098 (b) |
| CLTA | 0,000 | yes | 537,790 (a) | 542,142 (a) | 578,354 (a) | 940,794 (b) |

| | | | | | | |
|---|---|---|---|---|---|---|
| CLTC | 0,231 | no | 141,299 (a) | 106,379 (a) | 144,720 (a) | 114,141 (a) |
| CLU | 0,000 | yes | 712,028 (b) | 685,353 (b) | 1487 (c) | 127,758 (a) |
| CMTM6 | 0,000 | yes | 83,799 (a) | 82,246 (a) | 137,289 (b) | 82,557 (a) |
| COL6A1 | 0,000 | yes | 1077 (b) | 111,859 (a) | 155,817 (a) | 0,829 (a) |
| COLGALT1 | 0,000 | yes | 123,636 (b) | 234,158 (c) | 516,348 (d) | 15,317 (a) |
| CP | 0,000 | yes | 5,395 (b) | 0,641 (a) | 0,427 (a) | 0,207 (a) |
| CPD | 0,000 | yes | 5,081 (a) | 5,403 (a) | 12,515 (b) | 2,506 (a) |
| CYB5R3 | 0,000 | yes | 396,668 (b) | 55,144 (a) | 53,989 (a) | 47,980 (a) |
| DAD1 | 0,046 | yes | 282,690 (ab) | 244,960 (ab) | 213,699 (a) | 340,241 (b) |
| DDOST | 0,082 | no | 2,079 (a) | 1,453 (a) | 1,508 (a) | 0,500 (a) |
| DNAJC13 | 0,000 | yes | 3,563 (a) | 3,147 (a) | 3,375 (a) | 8,638 (b) |
| DYNC1I2 | 0,000 | yes | 33,596 (a) | 25,131 (a) | 23,837 (a) | 210,386 (b) |
| ECE1 | 0,000 | yes | 2,163 (b) | 2,378 (bc) | 3,327 (c) | 0,545 (a) |
| EDC4 | 0,000 | yes | 22,821 (a) | 25,287 (ab) | 44,086 (b) | 109,688 (c) |
| EEF1A2 | 0,122 | no | 0,728 (a) | 0,814 (a) | 0,605 (a) | 0,565 (a) |
| EEF1D | 0,000 | yes | 1577 (b) | 1338 (b) | 1610 (b) | 820,184 (a) |
| EEF1G | 0,001 | yes | 708,335 (b) | 475,693 (b) | 549,173 (b) | 78,092 (a) |
| EEF2 | 0,000 | yes | 2116 (c) | 1618 (b) | 2097 (c) | 484,727 (a) |
| EFCAB3 | 0,080 | no | 0,429 (a) | 0,509 (a) | 0,457 (a) | 0,358 (a) |
| EIF3A | 0,000 | yes | 43,606 (c) | 34,786 (b) | 45,213 (c) | 20,937 (a) |
| EIF3B | 0,000 | yes | 21,354 (a) | 22,124 (a) | 38,355 (b) | 63,883 (c) |
| ELAVL1 | 0,000 | yes | 32,655 (a) | 29,482 (a) | 28,791 (a) | 74,488 (b) |
| EMID1 | 0,038 | yes | 4,950 (ab) | 6,090 (ab) | 20,681 (b) | 0,874 (a) |
| ERCC3 | 0,000 | yes | 9,436 (a) | 8,788 (a) | 12,013 (a) | 62,505 (b) |
| ERH | 0,158 | no | 0,659 (a) | 0,842 (a) | 0,763 (a) | 1,020 (a) |
| ERP29 | 0,000 | yes | 306,508 (b) | 266,916 (b) | 305,135 (b) | 64,291 (a) |
| ERP44 | 0,000 | yes | 37,015 (a) | 49,605 (ab) | 63,439 (b) | 173,210 (c) |
| F2 | 0,000 | yes | 1,363 (a) | 2,371 (a) | 13,662 (b) | 1,404 (a) |
| FGA | 0,023 | yes | 0,393 (ab) | 0,491 (b) | 0,443 (ab) | 0,328 (a) |
| FGB | 0,000 | yes | 0,422 (a) | 0,600 (a) | 0,463 (a) | 2,508 (b) |
| FGG | 0,015 | yes | 0,420 (ab) | 0,543 (b) | 0,483 (b) | 0,292 (a) |
| FKBP11 | 0,000 | yes | 85,254 (c) | 24,875 (ab) | 43,580 (b) | 0,687 (a) |
| FN1 | 0,000 | yes | 274,609 (b) | 20,090 (a) | 3,175 (a) | 13,348 (a) |
| FTL | 0,084 | no | 505,777 (a) | 558,583 (a) | 729,822 (a) | 683,643 (a) |
| FUSIP1 | 0,000 | yes | 21,942 (a) | 36,640 (b) | 49,717 (c) | 31,339 (ab) |
| GANAB | 0,000 | yes | 114,726 (b) | 127,953 (b) | 178,353 (c) | 18,864 (a) |
| GAPDH | 0,000 | yes | 0,410 (b) | 0,464 (b) | 0,451 (b) | 0,220 (a) |
| GJA1 | 0,000 | yes | 272,764 (b) | 667,534 (c) | 709,514 (c) | 7,815 (a) |
| GNA11 | 0,000 | yes | 37,665 (a) | 27,050 (a) | 41,322 (a) | 182,958 (b) |
| GOLM1 | 0,000 | yes | 6,426 (c) | 3,784 (b) | 6,551 (c) | 1,196 (a) |
| GOLT1B | 0,000 | yes | 7,369 (a) | 7,021 (a) | 9,807 (a) | 62,216 (b) |
| GOT2 | 0,090 | no | 37,523 (a) | 43,653 (a) | 36,774 (a) | 56,476 (a) |
| GPX1 | 0,000 | yes | 333,816 (a) | 1332 (b) | 1473 (b) | 27,717 (a) |
| GPX8 | 0,000 | yes | 95,414 (c) | 73,916 (b) | 61,125 (b) | 10,885 (a) |
| GSN | 0,000 | yes | 332,871 (c) | 158,396 (b) | 126,453 (b) | 1,918 (a) |
| GSTA1 | 0,000 | yes | 908,988 (b) | 1177 (b) | 995,209 (b) | 7,019 (a) |
| GSTA2 | 0,006 | yes | 15,251 (ab) | 27,389 (b) | 16,591 (ab) | 0,230 (a) |
| GSTA5 | 0,000 | yes | 6,637 (a) | 64,183 (b) | 19,054 (a) | 0,243 (a) |
| H2AFV | 0,019 | yes | 201,409 (a) | 247,096 (ab) | 308,403 (b) | 197,541 (a) |
| HABP2 | 0,001 | yes | 0,554 (b) | 0,630 (b) | 0,633 (b) | 0,288 (a) |

| | | | | | | |
|---|---|---|---|---|---|---|
| HBB | 0,000 | yes | 0,586 (b) | 0,601 (b) | 0,435 (b) | 0,218 (a) |
| HIST1H1D | 0,005 | yes | 0,668 (ab) | 1,400 (b) | 0,888 (ab) | 0,192 (a) |
| HNRNPU | 0,000 | yes | 52,947 (a) | 61,658 (a) | 75,802 (a) | 121,070 (b) |
| HP1BP3 | 0,000 | yes | 138,357 (a) | 109,912 (a) | 122,303 (a) | 187,258 (b) |
| HPX | 0,001 | yes | 4,670 (b) | 4,781 (b) | 4,629 (b) | 2,915 (a) |
| HRG | 0,000 | yes | 0,451 (a) | 0,525 (a) | 0,478 (a) | 3,343 (b) |
| HSD17B1 | 0,000 | yes | 317,131 (b) | 873,825 (c) | 2188 (d) | 38,566 (a) |
| HSP90AA1 | 0,753 | no | 574,655 (a) | 542,217 (a) | 565,913 (a) | 515,250 (a) |
| HSP90AB1 | 0,330 | no | 19,811 (a) | 10,058 (a) | 12,435 (a) | 3,375 (a) |
| HSP90B1 | 0,005 | yes | 598,959 (a) | 553,362 (a) | 1116 (b) | 782,259 (ab) |
| HSPA6 | 0,004 | yes | 2,446 (ab) | 1,761 (a) | 1,431 (a) | 3,833 (b) |
| HSPA8 | 0,175 | no | 347,739 (a) | 482,409 (a) | 436,868 (a) | 292,496 (a) |
| HSPB1 | 0,000 | yes | 1885 (b) | 2636 (c) | 3211 (d) | 76,411 (a) |
| IER3IP1 | 0,000 | yes | 98,819 (a) | 194,777 (b) | 175,915 (b) | 182,993 (b) |
| IFITM3 | 0,000 | yes | 961,399 (c) | 497,004 (b) | 488,267 (b) | 4,043 (a) |
| IGLL1 | 0,024 | yes | 3,574 (b) | 0,704 (ab) | 0,629 (a) | 0,407 (a) |
| IGSF8 | 0,000 | yes | 3,976 (b) | 4,317 (b) | 10,195 (c) | 0,559 (a) |
| ITGA2 | 0,000 | yes | 0,557 (a) | 1,759 (b) | 3,653 (c) | 0,533 (a) |
| ITGA6 | 0,000 | yes | 1,866 (a) | 5,936 (b) | 6,641 (b) | 1,496 (a) |
| ITGB1 | 0,001 | yes | 269,576 (c) | 164,010 (a) | 199,859 (ab) | 264,385 (bc) |
| ITIH2 | 0,000 | yes | 0,428 (b) | 0,471 (b) | 0,419 (b) | 0,223 (a) |
| ITIH4 | 0,000 | yes | 3,998 (c) | 1,506 (ab) | 2,213 (b) | 0,502 (a) |
| LAMP2 | 0,077 | no | 55,452 (a) | 43,795 (a) | 55,110 (a) | 34,565 (a) |
| LMAN1 | 0,004 | yes | 5,513 (ab) | 9,436 (bc) | 11,057 (c) | 4,985 (a) |
| LMAN2 | 0,000 | yes | 303,308 (b) | 301,451 (b) | 382,024 (b) | 146,726 (a) |
| LRPAP1 | 0,415 | no | 6,223 (a) | 4,895 (a) | 5,831 (a) | 1,432 (a) |
| M6PR | 0,000 | yes | 24,630 (a) | 28,837 (ab) | 32,502 (b) | 43,498 (c) |
| MDH2 | 0,157 | no | 323,949 (a) | 575,067 (a) | 551,418 (a) | 616,439 (a) |
| MFGE8 | 0,000 | yes | 476,621 (a) | 2380 (b) | 2650 (b) | 70,336 (a) |
| MGP | 0,000 | yes | 2716 (b) | 132,536 (a) | 3,404 (a) | 4,264 (a) |
| MIF | 0,000 | yes | 30,195 (a) | 44,401 (a) | 261,681 (b) | 6,639 (a) |
| MLEC | 0,000 | yes | 7,252 (a) | 9,927 (a) | 11,466 (a) | 46,329 (b) |
| MPDU1 | 0,000 | yes | 82,155 (a) | 80,046 (a) | 84,427 (a) | 148,672 (b) |
| MRC2 | 0,042 | yes | 1,602 (ab) | 1,524 (ab) | 6,204 (b) | 0,284 (a) |
| MRPL3 | 0,004 | yes | 90,816 (ab) | 118,458 (bc) | 138,773 (c) | 77,906 (a) |
| MTDH | 0,224 | no | 54,303 (a) | 44,885 (a) | 56,727 (a) | 60,411 (a) |
| MVP | 0,000 | yes | 116,815 (a) | 75,853 (a) | 103,480 (a) | 1130 (b) |
| MYH10 | 0,000 | yes | 1,761 (a) | 0,828 (a) | 1,015 (a) | 8,456 (b) |
| MYL6 | 0,000 | yes | 1672 (c) | 1002 (b) | 1187 (b) | 347,027 (a) |
| MYOF | 0,000 | yes | 102,653 (b) | 134,738 (b) | 144,418 (b) | 6,644 (a) |
| NAPA | 0,149 | no | 86,794 (a) | 76,062 (a) | 93,063 (a) | 113,665 (a) |
| ORMDL1 | 0,000 | yes | 15,678 (a) | 25,856 (b) | 23,791 (b) | 10,867 (a) |
| ORMDL3 | 0,000 | yes | 0,688 (a) | 1,360 (b) | 1,203 (ab) | 2,570 (c) |
| PCBP1 | 0,000 | yes | 213,480 (a) | 206,754 (a) | 248,605 (a) | 839,252 (b) |
| PDIA3 | 0,002 | yes | 44,094 (b) | 34,423 (b) | 51,793 (b) | 5,441 (a) |
| PDIA6 | 0,003 | yes | 517,072 (a) | 619,400 (a) | 889,052 (b) | 476,990 (a) |
| PFN2 | 0,000 | yes | 90,319 (a) | 209,230 (bc) | 260,574 (c) | 159,932 (b) |
| PGD | 0,000 | yes | 151,406 (a) | 175,328 (a) | 299,991 (b) | 605,070 (c) |
| PGK1 | 0,000 | yes | 185,235 (ab) | 344,460 (b) | 853,946 (c) | 35,570 (a) |
| PGRMC1 | 0,000 | yes | 153,160 (b) | 82,756 (a) | 75,694 (a) | 239,794 (c) |

| | | | | | | |
|---|---|---|---|---|---|---|
| PGRMC2 | 0,001 | yes | 39,492 (b) | 30,839 (b) | 38,654 (b) | 18,926 (a) |
| PLG | 0,000 | yes | 0,549 (a) | 0,579 (a) | 0,822 (a) | 12,338 (b) |
| PLOD1 | 0,000 | yes | 6,995 (b) | 4,076 (ab) | 21,899 (c) | 0,290 (a) |
| PLP2 | 0,000 | yes | 136,650 (c) | 66,602 (b) | 84,781 (b) | 15,653 (a) |
| PPIB | 0,324 | no | 23,151 (a) | 5,531 (a) | 5,531 (a) | 0,945 (a) |
| PRDX1 | 0,024 | yes | 67,441 (a) | 57,425 (a) | 77,819 (ab) | 134,025 (b) |
| PRDX4 | 0,000 | yes | 273,562 (b) | 211,787 (b) | 381,624 (c) | 122,201 (a) |
| PRKCSH | 0,000 | yes | 326,512 (b) | 356,616 (bc) | 470,777 (c) | 12,251 (a) |
| PRPS1L1 | 0,018 | yes | 1,393 (a) | 1,802 (ab) | 2,235 (b) | 1,510 (a) |
| PRPSAP1 | 0,000 | yes | 125,197 (a) | 150,464 (a) | 126,897 (a) | 252,120 (b) |
| PRSS2 | 0,000 | yes | 0,511 (a) | 0,617 (a) | 4,122 (a) | 225,048 (b) |
| PSMA1 | 0,000 | yes | 89,056 (b) | 114,491 (bc) | 147,765 (c) | 2,637 (a) |
| PSMA5 | 0,000 | yes | 134,528 (a) | 175,392 (ab) | 245,707 (b) | 786,865 (c) |
| PSMA6 | 0,000 | yes | 193,638 (a) | 195,733 (a) | 242,935 (a) | 668,885 (b) |
| PSMC6 | 0,000 | yes | 61,594 (ab) | 75,399 (b) | 98,308 (c) | 41,088 (a) |
| PSMD2 | 0,050 | yes | 134,278 (a) | 170,857 (ab) | 225,465 (b) | 211,138 (ab) |
| PTGFRN | 0,000 | yes | 1,378 (a) | 3,498 (b) | 7,341 (c) | 0,643 (a) |
| RAB11A | 0,000 | yes | 6,941 (a) | 6,448 (a) | 5,338 (a) | 96,074 (b) |
| RAB14 | 0,032 | yes | 4,643 (b) | 4,097 (ab) | 3,685 (ab) | 2,710 (a) |
| RAB1A | 0,000 | yes | 46,298 (a) | 44,354 (a) | 49,147 (a) | 91,009 (b) |
| RAB2A | 0,000 | yes | 55,362 (a) | 46,986 (a) | 57,884 (a) | 167,073 (b) |
| RAB7A | 0,000 | yes | 33,844 (a) | 31,352 (a) | 38,252 (a) | 300,091 (b) |
| RAC1 | 0,000 | yes | 2,937 (b) | 1,556 (a) | 1,506 (a) | 3,284 (b) |
| RAD54B | 0,002 | yes | 2,555 (a) | 2,642 (a) | 3,068 (a) | 5,050 (b) |
| RAN | 0,122 | no | 50,301 (a) | 35,253 (a) | 33,829 (a) | 12,914 (a) |
| RBP4 | 0,000 | yes | 13,593 (a) | 9,249 (a) | 12,691 (a) | 49,015 (b) |
| RER1 | 0,139 | no | 20,003 (a) | 11,429 (a) | 10,931 (a) | 13,392 (a) |
| RPL10 | 0,000 | yes | 2559 (b) | 2685 (b) | 2614 (b) | 644,347 (a) |
| RPL10L | 0,000 | yes | 0,460 (b) | 0,553 (b) | 0,483 (b) | 0,209 (a) |
| RPL11 | 0,000 | yes | 2670 (b) | 2634 (b) | 2531 (b) | 593,396 (a) |
| RPL12 | 0,000 | yes | 1210 (b) | 1367 (b) | 1290 (b) | 295,309 (a) |
| RPL13 | 0,000 | yes | 2379 (b) | 2295 (b) | 2350 (b) | 713,350 (a) |
| RPL13A | 0,000 | yes | 2442 (b) | 2392 (b) | 2361 (b) | 458,256 (a) |
| RPL14 | 0,000 | yes | 2792 (c) | 2553 (bc) | 2368 (b) | 233,364 (a) |
| RPL15 | 0,044 | yes | 882,297 (ab) | 991,427 (ab) | 1089 (b) | 243,050 (a) |
| RPL17 | 0,000 | yes | 1344 (b) | 1424 (b) | 1301 (b) | 382,774 (a) |
| RPL18 | 0,000 | yes | 2879 (b) | 2837 (b) | 2719 (b) | 1118 (a) |
| RPL18A | 0,000 | yes | 2178 (b) | 2601 (c) | 2381 (bc) | 567,648 (a) |
| RPL19 | 0,000 | yes | 1912 (b) | 1922 (b) | 1895 (b) | 504,929 (a) |
| RPL21 | 0,000 | yes | 554,604 (b) | 409,258 (b) | 431,272 (b) | 140,487 (a) |
| RPL23 | 0,000 | yes | 2815 (c) | 2125 (b) | 2410 (bc) | 365,475 (a) |
| RPL23A | 0,000 | yes | 1339 (b) | 1409 (b) | 1429 (b) | 282,608 (a) |
| RPL26 | 0,000 | yes | 1550 (b) | 1607 (b) | 1480 (b) | 545,540 (a) |
| RPL27 | 0,000 | yes | 1916 (c) | 1627 (b) | 1579 (b) | 708,412 (a) |
| RPL27A | 0,000 | yes | 3033 (b) | 2734 (b) | 2890 (b) | 593,585 (a) |
| RPL30 | 0,000 | yes | 1086 (c) | 780,479 (b) | 886,705 (b) | 199,974 (a) |
| RPL32 | 0,000 | yes | 1745 (b) | 1756 (b) | 1686 (b) | 136,826 (a) |
| RPL35 | 0,000 | yes | 148,289 (c) | 92,043 (b) | 105,628 (b) | 32,842 (a) |
| RPL35A | 0,000 | yes | 2263 (b) | 2481 (b) | 2288 (b) | 632,189 (a) |
| RPL36 | 0,000 | yes | 1602 (b) | 1550 (b) | 1602 (b) | 411,472 (a) |

| | | | | | | |
|---|---|---|---|---|---|---|
| RPL38 | 0,000 | yes | 1620 (b) | 1475 (b) | 1583 (b) | 979,255 (a) |
| RPL4 | 0,000 | yes | 1890 (b) | 1796 (b) | 1721 (b) | 599,179 (a) |
| RPL5 | 0,000 | yes | 2149 (b) | 2246 (b) | 2164 (b) | 370,717 (a) |
| RPL6 | 0,000 | yes | 2050 (c) | 1694 (b) | 1625 (b) | 431,818 (a) |
| RPL7 | 0,000 | yes | 213,479 (c) | 161,733 (bc) | 143,225 (b) | 56,268 (a) |
| RPL7A | 0,000 | yes | 3117 (b) | 3246 (b) | 3173 (b) | 1241 (a) |
| RPL8 | 0,000 | yes | 2489 (b) | 2476 (b) | 2421 (b) | 538,350 (a) |
| RPL9 | 0,000 | yes | 1674 (c) | 1387 (b) | 1526 (bc) | 128,213 (a) |
| RPLP0 | 0,000 | yes | 3025 (b) | 3502 (b) | 3142 (b) | 1143 (a) |
| RPS10 | 0,000 | yes | 2952 (b) | 2832 (b) | 2852 (b) | 815,658 (a) |
| RPS11 | 0,000 | yes | 2125 (b) | 1979 (b) | 2261 (b) | 377,167 (a) |
| RPS13 | 0,000 | yes | 1996 (b) | 1980 (b) | 1976 (b) | 499,605 (a) |
| RPS14 | 0,000 | yes | 1343 (b) | 1212 (b) | 1202 (b) | 100,824 (a) |
| RPS15A | 0,000 | yes | 2270 (b) | 1893 (b) | 1906 (b) | 456,480 (a) |
| RPS16 | 0,000 | yes | 2683 (b) | 2475 (b) | 2588 (b) | 578,020 (a) |
| RPS17 | 0,000 | yes | 2529 (b) | 2319 (b) | 2660 (b) | 563,045 (a) |
| RPS18 | 0,000 | yes | 3190 (b) | 3252 (b) | 3257 (b) | 727,279 (a) |
| RPS2 | 0,000 | yes | 1908 (b) | 1840 (b) | 1829 (b) | 517,127 (a) |
| RPS23 | 0,000 | yes | 1329 (c) | 1111 (b) | 1100 (b) | 287,828 (a) |
| RPS24 | 0,000 | yes | 1316 (c) | 794,930 (b) | 811,355 (b) | 256,968 (a) |
| RPS25 | 0,000 | yes | 1597 (c) | 1270 (b) | 1304 (b) | 274,674 (a) |
| RPS26 | 0,000 | yes | 1009 (c) | 714,952 (b) | 808,067 (b) | 135,648 (a) |
| RPS27A | 0,000 | yes | 4158 (b) | 4284 (b) | 4010 (b) | 1262 (a) |
| RPS27L | 0,079 | no | 114,162 (a) | 103,461 (a) | 132,083 (a) | 95,221 (a) |
| RPS3 | 0,000 | yes | 3818 (b) | 4001 (b) | 3831 (b) | 1162 (a) |
| RPS3A | 0,000 | yes | 2749 (b) | 2508 (b) | 2412 (b) | 559,620 (a) |
| RPS4Y1 | 0,000 | yes | 855,385 (b) | 719,949 (b) | 784,123 (b) | 351,499 (a) |
| RPS5 | 0,000 | yes | 2171 (b) | 1872 (b) | 2255 (b) | 780,122 (a) |
| RPS6 | 0,000 | yes | 543,388 (b) | 450,370 (b) | 495,301 (b) | 94,467 (a) |
| RPS7 | 0,000 | yes | 1805 (b) | 1753 (b) | 1675 (b) | 594,807 (a) |
| RPS8 | 0,000 | yes | 2599 (b) | 3032 (b) | 2637 (b) | 826,898 (a) |
| RPS9 | 0,132 | no | 365,521 (a) | 192,061 (a) | 207,010 (a) | 10,501 (a) |
| RPSA | 0,000 | yes | 1971 (b) | 2173 (b) | 1925 (b) | 777,342 (a) |
| S100A8 | 0,000 | yes | 0,867 (bc) | 1,271 (c) | 0,584 (ab) | 0,244 (a) |
| SCAMP2 | 0,000 | yes | 67,556 (b) | 36,957 (a) | 48,975 (a) | 108,271 (c) |
| SCAMP3 | 0,000 | yes | 123,981 (a) | 126,553 (a) | 154,982 (a) | 385,571 (b) |
| SDCBP | 0,001 | yes | 14,724 (ab) | 10,393 (a) | 8,247 (a) | 21,309 (b) |
| SEC24C | 0,027 | yes | 0,959 (ab) | 1,085 (ab) | 1,218 (b) | 0,879 (a) |
| SEC61A1 | 0,000 | yes | 13,201 (a) | 12,393 (a) | 11,575 (a) | 24,784 (b) |
| SERPIND1 | 0,000 | yes | 0,476 (b) | 0,471 (b) | 0,435 (b) | 0,211 (a) |
| SERPINE2 | 0,000 | yes | 16,348 (a) | 113,613 (b) | 124,110 (b) | 6,378 (a) |
| SERPINH1 | 0,000 | yes | 188,408 (bc) | 136,620 (b) | 222,703 (c) | 1,088 (a) |
| SFPQ | 0,112 | no | 31,915 (a) | 45,463 (a) | 44,523 (a) | 33,772 (a) |
| SFRS9 | 0,002 | yes | 104,472 (a) | 118,705 (a) | 128,252 (a) | 185,919 (b) |
| SLC25A12 | 0,000 | yes | 7,972 (b) | 3,793 (a) | 3,578 (a) | 7,481 (b) |
| SLC29A1 | 0,132 | no | 36,285 (a) | 34,479 (a) | 25,852 (a) | 14,404 (a) |
| SORT1 | 0,000 | yes | 0,602 (a) | 1,302 (b) | 2,723 (c) | 2,634 (c) |
| SRGN | 0,000 | yes | 211,211 (a) | 1962 (b) | 1836 (b) | 30,715 (a) |
| SRSF7 | 0,000 | yes | 82,531 (a) | 107,994 (a) | 111,871 (a) | 173,258 (b) |
| SSR4 | 0,012 | yes | 242,531 (b) | 140,969 (ab) | 144,026 (ab) | 11,748 (a) |

| | | | | | | |
|---|---|---|---|---|---|---|
| STOM | 0,000 | yes | 17,297 (c) | 5,242 (a) | 5,770 (a) | 10,913 (b) |
| STRA6 | 0,000 | yes | 90,346 (a) | 1164 (b) | 2488 (c) | 23,725 (a) |
| STX6 | 0,000 | yes | 11,682 (a) | 16,320 (a) | 13,840 (a) | 87,541 (b) |
| STX7 | 0,000 | yes | 23,188 (a) | 32,050 (a) | 33,949 (a) | 90,033 (b) |
| TERT | 0,003 | yes | 0,420 (b) | 0,494 (b) | 0,464 (b) | 0,284 (a) |
| TF | 0,000 | yes | 20,355 (c) | 2,374 (a) | 0,843 (a) | 10,709 (b) |
| TFRC | 0,000 | yes | 1,731 (a) | 3,302 (b) | 4,608 (c) | 6,180 (d) |
| TLL2 | 0,000 | yes | 1,053 (a) | 14,776 (b) | 37,259 (c) | 0,419 (a) |
| TMED10 | 0,000 | yes | 28,987 (b) | 26,326 (b) | 25,493 (b) | 9,477 (a) |
| TMED4 | 0,000 | yes | 105,228 (a) | 168,937 (b) | 252,961 (c) | 113,434 (ab) |
| TMED7 | 0,000 | yes | 16,119 (a) | 12,712 (a) | 14,272 (a) | 52,639 (b) |
| TMEM109 | 0,000 | yes | 57,243 (a) | 24,254 (a) | 32,658 (a) | 377,463 (b) |
| TMEM14C | 0,000 | yes | 105,826 (a) | 71,034 (a) | 73,712 (a) | 342,313 (b) |
| TMEM167A | 0,000 | yes | 8,256 (a) | 12,082 (a) | 12,039 (a) | 60,437 (b) |
| TRA2B | 0,000 | yes | 30,341 (b) | 42,842 (b) | 48,274 (b) | 3,487 (a) |
| TTR | 0,011 | yes | 2,191 (ab) | 1,356 (ab) | 0,792 (a) | 2,928 (b) |
| TUBA1C | 0,000 | yes | 122,269 (a) | 278,316 (a) | 282,515 (a) | 2021 (b) |
| UGGT1 | 0,002 | yes | 1,551 (ab) | 2,302 (bc) | 2,387 (c) | 1,204 (a) |
| VCP | 0,002 | yes | 90,626 (a) | 104,015 (a) | 174,951 (b) | 63,306 (a) |
| VDAC1 | 0,526 | no | 276,849 (a) | 256,657 (a) | 304,975 (a) | 258,025 (a) |
| VDAC2 | 0,000 | yes | 47,805 (a) | 51,748 (a) | 93,724 (b) | 32,639 (a) |
| VIM | 0,000 | yes | 730,037 (b) | 913,989 (b) | 1235 (c) | 7,346 (a) |
| VTN | 0,001 | yes | 0,381 (b) | 0,470 (b) | 0,462 (b) | 0,208 (a) |
| YWHAQ | 0,252 | no | 283,443 (a) | 347,164 (a) | 353,980 (a) | 310,860 (a) |
